# Synthesis of Novel Bioactive Lipophilic Hydroxyalkyl Esters and Diesters Based on Hydroxyphenylacetic Acids

**DOI:** 10.3390/molecules30153087

**Published:** 2025-07-23

**Authors:** Andrea Fochetti, Noemi Villanova, Andrea Lombardi, Veronica Lelli, Yuri Gazzilli, Anna Maria Timperio, Giancarlo Fabrizi, Roberta Bernini

**Affiliations:** 1 Department of Agriculture and Forest Sciences (DAFNE), University of Tuscia, Via San Camillo de Lellis, 01100 Viterbo, Italy; noemi.villanova@unitus.it (N.V.); andrea.lombardi@unitus.it (A.L.); 2 Bioricerche S.r.l., Loc. Ferro di Cavallo, 58034 Castell’Azzara, Italy; 3 Department of Ecological and Biological Sciences (DEB), University of Tuscia, 01100 Viterbo, Italy; v.lelli@unitus.it (V.L.); timperio@unitus.it (A.M.T.); 4 Department of Chemistry and Technology of Drugs, Sapienza University of Rome, P.le Aldo Moro 5, 00185 Rome, Italy; yuri.gazzilli@uniroma1.it (Y.G.); giancarlo.fabrizi@uniroma1.it (G.F.)

**Keywords:** hydroxyphenylacetic acids, hydroxyalkyl esters, butyl diarylacetates, lipophilicity, antioxidant activity, DPPH assay, ABTS assay, antibacterial activity

## Abstract

Novel lipophilic hydroxyalkyl esters were synthetized by Fischer esterification in good to excellent yields (60–96%) from a panel of hydroxyphenylacetic acids and increasing chain length (2 to 8 carbon atoms) α,ω-diols. The in vitro antioxidant activity of these compounds was evaluated by DPPH and ABTS assays. Hydroxybutyl esters and hydroxyphenylacetic acids were used as starting materials for the synthesis of novel lipophilic diesters (butyl diarylacetates) using Mitsunobu reaction. The final products were isolated in moderate to good yields (40–78%), and their structure–antioxidant activity relationships are discussed. Compounds bearing the catechol moiety on one of the two aromatic rings and high lipophilicity proved to be the strongest antioxidants and were selected for testing as antibacterials against *Staphylococcus aureus* and *Escherichia coli*, obtaining preliminary and promising results.

## 1. Introduction

Polyphenols are a wide family of naturally occurring compounds produced by plants, bacteria, and fungi, and they play a crucial role in the physiology of these organisms [1]. They are renowned for their multiple biological properties, including antioxidant, anti-inflammatory, anticancer, and antimicrobial activities [2,3,4]. Considering the beneficial effects on human health, polyphenols are employed to prevent various diseases, including cancer, cardiovascular diseases, diabetes mellitus, atherosclerosis, and neurological and cardiovascular disorders [5,6]. Beyond their pharmacological uses, these compounds have also been investigated for applications in food, cosmetics, packaging, and textiles [7,8,9,10,11].

Polyphenols include phenolic acids, such as hydroxybenzoic acids, hydroxycinnamic acids, and hydroxyphenylacetic acids. Hydroxybenzoic acids and hydroxycinnamic acids have been widely studied for their numerous biological properties and applications [12,13,14], while hydroxyphenylacetic acids have received little attention so far, and only a few biological activities have been evaluated. As an example, 3,4-dihydroxyphenylacetic acid has been tested for antiproliferative activity in prostate and colon cancer cells [15,16], as well as against oxidative-stress-induced cytotoxicity in human neuroblastoma SH-SY5Y cells, showing a neuroprotective effect [17].

The general limiting aspects to the application of polyphenols are the poor pharmacokinetic properties and low bioavailability, and the importance of increasing the lipophilic character of polyphenols has already been emphasized throughout the years [18,19]. To achieve this aim, chemical reactions, such as halogenation, esterification, etherification, or amidation, have been proposed to introduce a lipophilic unit into these compounds without modifying the phenolic moiety responsible for the biological properties [20,21,22,23]. As an example, the selective esterification of the alcoholic moiety reported for tyrosol and hydroxytyrosol afforded the corresponding lipophilic esters, which exhibited antimicrobial and antioxidant activities [24,25,26,27,28]. Methyl, butyl, and hexanoyl esters of 3,4-dihydroxyphenylacetic acid, showing enhanced solubility in oil systems, were obtained by esterification and transesterification reactions [29].

The incorporation of a lipophilic unit between two phenolic moieties has also emerged as a useful strategy to increase both the lipophilicity and the biological activities of phenolic compounds. For this reason, some studies have focused on synthesizing new derivatives from two phenolic monomers, linked by alkyl chains of varying lengths. Branched alkyl esters and amides of gallic acid represent a first example [30]. More recently, caffeic and sinapic acid derivatives with diols of different carbon chain lengths [31], diesters consisting of hydroxytyrosol units linked by carbon chains [32], and 3,4-dihydroxyphenylacetic acid ester and amide derivatives and conjugates have been reported [33]. Most of these syntheses require more steps, including protection and deprotection of the phenolic groups.

To the best of our knowledge, among hydroxyphenylacetic acids analogues, only 2-hydroxyethyl 2-(4-hydroxyphenyl) acetate [34] butane-1,4-diyl bis(2-(3,4-dihydroxyphenyl)acetate) [33] and butane-1,4-diyl bis(2-(4-hydroxyphenyl)acetate) have recently been described, with the latter being intermediate for the synthesis of flame-retardant benzoxazine derivatives [35].

Given the lack of existing literature, this work focused on the synthesis of novel hydroxyalkyl esters from hydroxyphenylacetic acids and aliphatic α,ω-diols with chain lengths ranging from 2 to 8 carbon atoms, whose antioxidant activity was evaluated by in vitro assays. Hydroxybutyl esters were used as starting materials for the synthesis of butyl diarylacetates in combination with hydroxyphenylacetic acids to investigate their antioxidant properties and carry out a structure–antioxidant activity relationship analysis. After careful study of the synthetic procedures reported in the literature for obtaining lipophilic phenolic diesters [30,31,32,33,34,35], attention was focused on the two-step procedure used for hydroxyalkyl esters and bis-aryl esters based on sinapic and caffeic acids, involving simple reactions, such as Fischer esterification and Mitsunobu reaction, without protection or deprotection of the phenolic groups [31]. Additionally, preliminary study of the antibacterial activity of selected butyl diarylacetates was performed against *Staphylococcus aureus* and *Escherichia coli*, as examples of Gram-positive and Gram-negative bacteria.

## 2. Results and Discussions

### 2.1. Synthetic Procedures

**Scheme 1:**
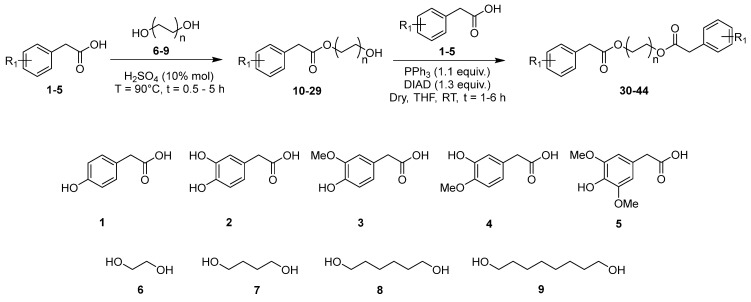
Two-step synthesis of butyl diarylacetates **30**–**44** from hydroxyphenylacetic acids **1**–**5** and diols **6**–**9**.

The synthesis of alkyl diarylacetates was carried out in two steps, adapting a procedure described in the literature for sinapic and caffeic acids [31] (Scheme 1). The first step involved Fischer esterification of 4-hydroxyphenylacetic acid **1**; 3,4-dihydroxyphenylacetic acid **2**; 4-hydroxy-3-methoxyphenylacetic acid **3**; 3-hydroxy-4-methoxyphenylacetic acid **4**; 4-hydroxy-3,5-dimethoxyphenylacetic acid **5** with 1,2-ethandiol **6**; 1,4-butanediol **7**; 1,6-hexanediol **8**, and 1,8-octanediol **9** to obtain the corresponding hydroxyalkyl esters **10**–**29**. The second step was a Mitsunobu reaction between selected hydroxybutyl esters **11**,**15**,**19**,**23**,**27** and hydroxyphenylacetic acids **1**–**5**, affording the corresponding butyl diarylacetates **30**–**44**.

### 2.2. Synthesis of Hydroxyalkyl Esters ***10***–***29***

**Scheme 2:**
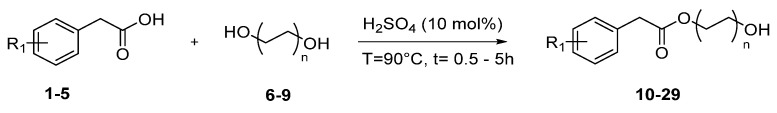
Synthesis of hydroxyalkyl esters **10**–**29** via Fischer esterification.

As intermediates for the synthesis of the designed diesters, hydroxyalkyl esters **10**–**29** were synthesized by treating hydroxyphenylacetic acids **1**–**5** with diols **6**–**9** (molar ratio = 1/30) in presence of catalytic amounts of sulphuric acid for 0.5–5 h at T = 90 °C (Scheme 2). In all reactions, the diol was used as both reagent and solvent. After the work-up and the chromatographic purification of the crude on a silica gel column, the corresponding hydroxyalkyl esters were obtained as pure samples in good to excellent yields (60–96%). The reported data in Table 1 evidence that the yields were lower when the acids reacted with diol **9**, which has a C-8 chain (entries 4, 8, 16, and 20), likely because of difficulties in purification from the latter. Ester **17** was not completely separated from diol **9** after the chromatographic column; however, a pure sample was obtained through a semi-preparative RP-HPLC system (see Section 3).

The logP values of hydroxyalkyl esters **10**–**29** are included in Table 1. As expected, for each series of esters, they were positive and significantly increased with the alkyl chain length. As an example, from 2-hydroxyethyl 2-(4-hydroxyphenyl)acetate **10** to 8-hydroxyoctyl 2-(4-hydroxyphenyl)acetate **13**, the logP increased from 0.89 to 3.12 (entries 1–4).

### 2.3. Antioxidant Activity of Hydroxyphenylacetic Acids ***1***–***5*** and of Hydroxyalkyl Esters ***10***–***29*** by DPPH and ABTS Assays

The antioxidant activities of all products were carried out using two in vitro assays, 2,2-diphenyl-1-picrylhydrazyl (DPPH) and 2,2′-azino-bis(3-ethylbenzothiazoline-6-sulfonic acid) (ABTS), commonly used for phenolic compounds [36,37,38]. The DPPH data are expressed as the concentration of the sample required to inhibit 50% of the radical (IC_50_, μM), and the ABTS data as the Trolox equivalent antioxidant capacity (TEAC, μM). 

**Figure 1:**
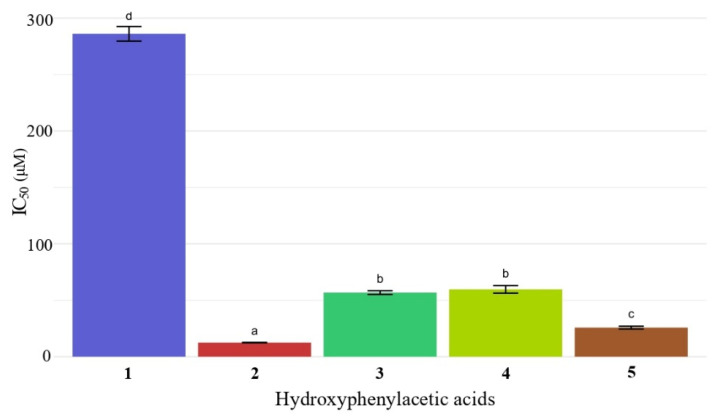
DPPH assay of hydroxyphenylacetic acids **1**–**5**. The IC_50_ values (μM) are expressed as the mean ± standard deviation (n = 3). The analysis of variance (ANOVA) revealed a highly significant difference among the groups. Tukey’s post hoc test was applied to compare IC_50_ values across compounds and identified significant differences between the following pairs. The letters above each bar represent the results of Tukey’s test (*p* < 0.05). 4-Hydroxyphenylacetic acid **1** (d) exhibited significant differences from **2** (a, *p* < 0.0000001), **3** (b, *p* < 0.0000001), **4** (b, *p* < 0.0000001), and **5** (c, *p* < 0.0000001); 3,4-dihydroxyphenylacetic acid **2** (a) was significantly different from **3** (b, *p* = 0.0000003), **4** (b, *p* = 0.0000001), and **5** (c, *p* = 0.0082). No significant difference was observed between hydroxyphenylacetic acids **3** and **4** (b, *p* = 0.4841). 3,5-Dimethoxy-4-hydroxyphenylacetic acid **5** (c) demonstrated significant differences from **3** (b, *p* = 0.0000095) and **4** (b, *p* = 0.0000023).

**Figure 2:**
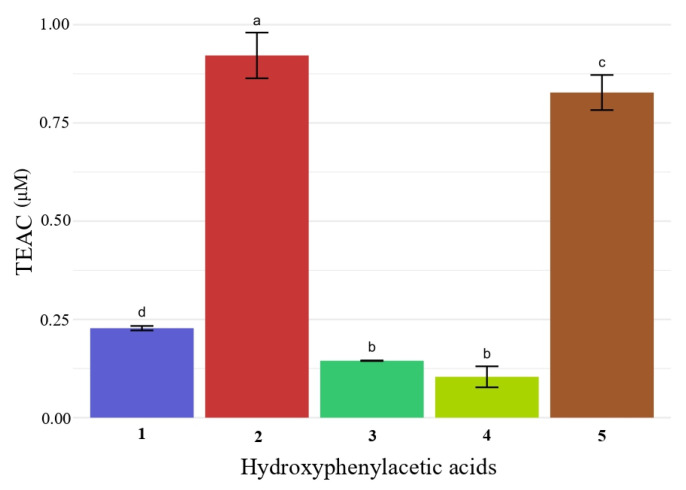
ABTS assay of hydroxyphenylacetic acids **1**–**5**. TEAC values (μM) are expressed as the mean ± standard deviation (n = 3). The analysis of variance (ANOVA) revealed a highly significant difference among the groups. Tukey’s post hoc test was applied to compare the TEAC values across compounds and identified significant differences between the following pairs. The letters above each bar represent the results of Tukey’s test (*p* < 0.05). Hydroxyphenylacetic acid **1** (d) was significantly different from **2** (a, *p* < 0.0000001), **3** (b, *p* = 0.0273), **4** (b, *p* = 0.0038), and **5** (c, *p* < 0.0000001). 3,4-dihydroxyphenylacetic acid **2** (a) was significantly different from **3** (b, *p* < 0.0000001), **4** (b, *p* < 0.0000001), and **5** (c, *p* = 0.0014). No significant difference was observed between hydroxyphenylacetic acids **3** and **4** (b, *p* = 0.6842). 3,5-dimethoxy-4-hydroxyphenylacetic acid **5** (c) was significantly different from **3** (b, *p* < 0.0000001) and **4** (b, *p* < 0.0000001).

Firstly, the antioxidant activity of hydroxyphenylacetic acids **1**–**5** (Table 2, entries 1, 6, 11, 16, and 21) was evaluated. The DPPH and ABTS values were also statistically analyzed using the post hoc Tukey test (Figure 1 and Figure 2).

Both assays demonstrated that 4-hydroxyphenylacetic acid **1** displayed no significant antioxidant activity, with the highest IC_50_ value and a low TEAC value (>200 and <0.05 μM, respectively, entry 1), while hydroxyphenylacetic acids **2**–**4** exhibited antioxidant activity correlated with the substitution pattern on the aromatic ring. In particular, 3,4-dihydroxyphenylacetic acid **2** exhibited the highest antioxidant activity (IC_50_ = 12.5 ± 0.2 μM, TEAC = 0.92 ± 0.05 μM, entry 6), followed by 3,5-dimethoxy-4-hydroxyphenylacetic acid **5** (IC_50_ = 25.8 ± 1.2 μM, TEAC = 0.82 ± 0.04 μM, entry 21), 4-hydroxy-3-methoxyphenylacetic acid **3** (IC_50_ = 56.8 ± 1.6 μM, TEAC = 0.14 ± 0.01 μM, entry 11), and 3-hydroxy-4-methoxyphenylacetic acid **4** (IC_50_ = 59.7 ± 3.3 μM, TEAC = 0.10 ± 0.02 μM, entry 16).

These experimental results are consistent with the available data in the literature [39]. 4-Hydroxyphenylacetic acid **1**, which only possesses a hydroxyl group on the aromatic ring, showed no significant antioxidant activity. On the contrary, the scavenging activity of acids **2**–**5** was attributed to the presence of a hydroxyl group in the aromatic moiety of the starting acid, which can act as an electron-donating group [40], in combination with another substituent (hydroxyl or methoxy group). Replacement of this substituent with other functional groups strongly affects the antioxidant activity, and steric effects can have a big impact on the radical scavenging activity [41,42]. However, the difference between regioisomers **3** and **4** was not statistically significant due to the hydroxy and methoxy groups on the aromatic ring. Data obtained from both assays appeared consistent, where compounds **1**, **2**, and **5** were not comparable, and compounds **3** and **4** showed similarity.

Subsequently, DPPH and ABTS assays were performed on novel hydroxyalkyl esters **10**–**29** (Table 2, entries 2–5, 7–10, 12–15, 17–20, and 22–25). As expected, and demonstrated by the IC_50_ (μM) and TEAC (μM) values, the introduction of the hydroxyalkyl chain did not modify the scale of the antioxidant activity previously discussed for hydroxyphenylacetic acids **1**–**5**. Hydroxyalkyl esters **10**–**13** derived from 4-hydroxyphenylacetic acid **1** did not show antioxidant activity (entries 2–5); esters **14**–**17** and **26**–**29**, obtained from 3,4-dihydroxyphenylacetic acid **2** and 3,5-dimethoxy-4-hydroxyphenylacetic acid **5**, were the best antioxidants (entries 7–10 and 22–25); **18**–**21** and **22**–**25** derived from 4-hydroxy-3-methoxyphenylacetic acid **3** and 3-hydroxy-4-methoxyphenylacetic acid **4** showed similar antioxidant activity (entries 12–15 and 17–20). Regarding the effect of the hydroxyalkyl chain length, the experimental data did not follow a regular trend, and no data in the literature were available for these compounds. Hydroxyalkyl esters **14**–**17** and **26**–**29** showed similar antioxidant activity compared with 3,4-dihydroxyphenylacetic acid **2** and 3,5-dimethoxy-4-hydroxyphenylacetic acid **5** (entries 7–10 and 22–25). On the contrary, hydroxyalkyl esters **18**–**21** and **22**–**25** exhibited increasing antioxidant activity compared with 4-hydroxy-3-methoxyphenylacetic acid **3** and 3-hydroxy-4-methoxyphenylacetic acid **4** (entries 12–15 and 17–20).

**Figure 3:**
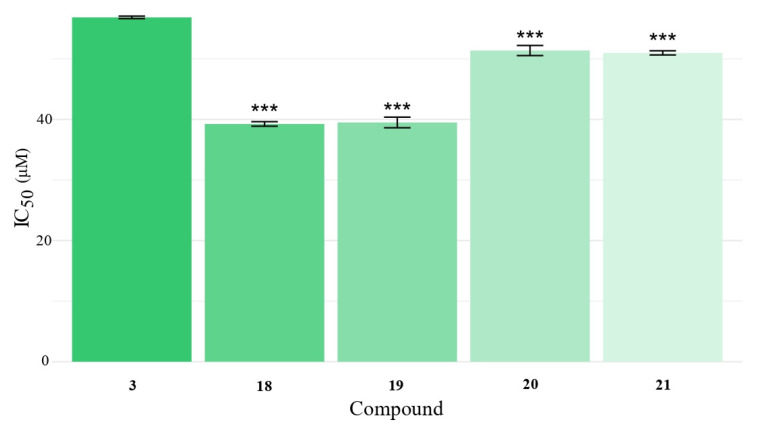
DPPH assay of hydroxyalkyl esters **18**–**21** compared with 4-hydroxy-3-methoxyphenylacetic acid **3**. IC_50_ values (μM) are expressed as the mean ± standard deviation (n = 3). The ANOVA revealed a highly significant difference among groups, and Dunnett’s multiple comparison test was applied to compare **18**–**21** with **3** (*** *p* < 0.001). All tested hydroxyalkyl esters **18**–**21** were significantly different from **3**, exhibiting higher antioxidant activity.

**Figure 4:**
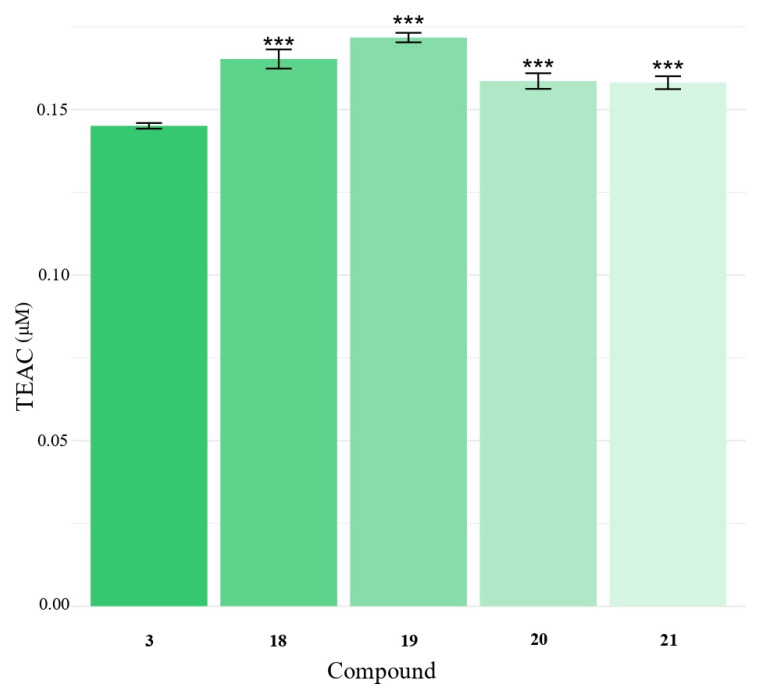
ABTS assay of hydroxyalkyl esters **18**–**21** compared with 4-hydroxy-3-methoxyphenylacetic acid **3**. TEAC values (μM) are expressed as the mean ± standard deviation (n = 3). Dunnett’s post hoc test was applied to compare the samples with **3**. The analysis revealed significant differences in the TEAC values between hydroxyalkyl esters **18**–**21** and **3**. The results show that **18**–**21** exhibited significantly lower TEAC values compared with **3**, exhibiting higher antioxidant activity (*** *p* < 0.001).

The DPPH and ABTS data for each series of compounds were statistically analyzed by the post hoc Dunnett test. For example, Figure 3 and Figure 4 report the results for hydroxyalkyl esters **18**–**21** compared with 4-hydroxy-3-methoxyphenylacetic acid **3**.

### 2.4. Synthesis of Butyl Diarylacetates ***30**–**44***

**Scheme 3:**
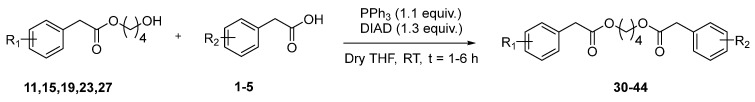
Synthesis of butyl diarylacetates **30**–**44** with Mitsunobu reaction.

In this study, we decided to carry out the synthesis of butyl diarylacetates as examples of alkyl diarylacetates. The choice was justified in consideration of the reaction yields, lipophilicity, and the straightforward purification procedure. Specifically, 4-hydroxybutyl 2-(4-hydroxyphenyl)acetate **11**, 4-hydroxybutyl 2-(3,4-dihydroxyphenyl)acetate **15**, 4-hydroxybutyl 2-(4-hydroxy-3-methoxyphenyl)acetate **19**, 4-hydroxybutyl 2-(3-hydroxy-4-methoxyphenyl)acetate **23**, and 4-hydroxybutyl 2-(4-hydroxy-3,5-dimethoxyphenyl)acetate **27** were esterified with hydroxyphenylacetic acids **1**–**5** under Mitsunobu conditions. The reactions were carried out using a slight excess of triphenyl phosphine (PPh_3_) and diisopropyl azodicarboxylate (DIAD) in tetrahydrofuran (THF) at room temperature for 1–6 h (Scheme 3). The reaction conditions are detailed in Table 3.

4-Hydroxybutyl 2-(4-hydroxyphenyl)acetate **11**, in combination with hydroxyphenylacetic acids **1**–**5**, afforded the corresponding diesters **30**–**34** in 53–60% yields (entries 1–5). Unfortunately, the isolation of diester **35** failed (entry 6). 4-Hydroxybutyl 2-(3,4-dihydroxyphenyl)acetate **15** reacted with hydroxyphenylacetic acids **3**–**5** to obtain diesters **36**–**38** in 45–78% yields (entries 7–9). 4-Hydroxybutyl 2-(4-hydroxy-3-methoxyphenyl)acetate **19** reacted with **3**–**5** producing **39**–**41** in 48–60% yields (entries 10–12); 4-hydroxybutyl 2-(3-hydroxy-4-methoxyphenyl)acetate **23** was combined with **4** and **5** to obtain **42** and **43** in 65% and 40% yields, respectively (entries 13,14). Finally, 4-hydroxybutyl 2-(4-hydroxy-3,5-dimethoxyphenyl)acetate **27** in combination with **5** afforded diester **44** in a 71% yield (entry 15). As shown in Table 3, the logP values range from 2.49 (entry 9) to 3.13 (entry 1), indicating a high lipophilicity that varied slightly depending on the substitution on the aromatic rings.

### 2.5. Antioxidant Activity of Butyl Diarylacetates by DPPH and ABTS Assays

Butyl diarylacetates **30**–**34** and **36**–**44** were tested for their antioxidant activity by DPPH and ABTS assays. The IC_50_ and TEAC values are reported in Table 4. All diesters showed antioxidant activity, the effectiveness of which varied according to the substitution pattern on the aromatic rings. The only compound with a poor radical-reducing ability was **30**, having a hydroxy group on both aromatic rings (IC_50_ > 200 μM, TEAC < 0.05 μM, entry 1). These data are in accordance with the high IC_50_ and low TEAC values of 4-hydroxyphenylacetic acid **1** (Table 2, entry 1) and esters **10**–**13** (Table 2, entries 2–5). However, when **1** was esterified with hydroxyphenylacetic acids **2**–**5**, the corresponding diesters **31**–**34** showed antioxidant activity, which increased significantly from **32** (IC_50_ = 60.8 ± 1.0 μM, TEAC = 0.08 ± 0.02 μM, entry 3) and **33** (IC_50_ = 43.1 ± 4.5 μM, TEAC = 0.09 ± 0.03 μM, entry 4) to **34** (IC_50_ = 26.9 ± 0.3 μM, TEAC = 0.38 ± 0.01 μM, entry 5) and **31** (IC_50_ = 19.5 ± 0.7 μM, TEAC = 0.94 ± 0.01 μM, entry 2), evidencing the relevant roles of the guaiacyl, syringyl, and catechol moieties [41,42]. A similar effect was observed with **37**, having a catecholic moiety on the first aromatic ring and a guaiacyl group on the other aromatic ring, which exerted excellent antioxidant activity with the lowest IC_50_ value of all diesters (12.2 ± 0.1 μM, entry 7) and a high TEAC value (0.71 ± 0.03 μM, entry 7). Similar activity was also observed for diesters **36** (IC_50_ = 12.6 ± 0.1 μM, TEAC = 0.71 ± 0.03 μM, entry 6) and **38** (IC_50_ = 17.5 ± 0.8 μM, TEAC = 0.49 ± 0.04 μM, entry 8). Also, the syringyl group conferred antioxidant activity to the diesters, as highlighted by the antioxidant activity of **34** (IC_50_ = 26.9 ± 0.3 μM, TEAC = 0.38 ± 0.01 μM, entry 5), **38** (IC_50_ = 17.5 ± 0.8 μM, TEAC = 0.49 ± 0.04 μM, entry 9), **41** (IC_50_ = 27.3 ± 0.8 μM, TEAC = 0.56 ± 0.02 μM, entry 11), and **43** (IC_50_ = 26.4 ± 1.54 μM, TEAC = 0.68 ± 0.01 μM, entry 13). Diester **44**, having the syringyl group on both aromatic rings, showed a high antioxidant activity with an IC_50_ = 14.3 ± 1.8 μM and TEAC = 0.20 ± 0.01 μM (entry 14). Finally, the guaiacyl present on both aromatic rings conferred discrete antioxidant activity to diesters **39** (IC_50_ = 38.2 ± 0.4 μM, TEAC = 0.13 ± 0.06 μM, entry 9), **40** (IC_50_ = 35.3 ± 0.1 μM, TEAC = 0.13 ± 0.08 μM, entry 10), and **42** (IC_50_ = 31.3 ± 2.8 μM, TEAC = 0.11 ± 0.01 μM, entry 12).

**Figure 5:**
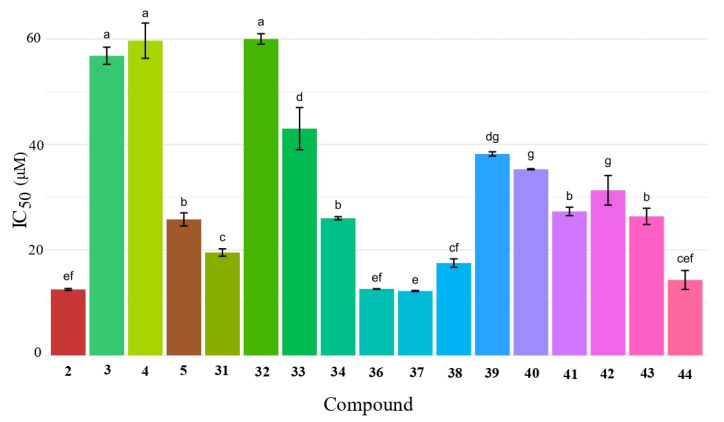
DPPH data of butyl diarylacetates **31**–**34** and **36**–**44** compared with hydroxyphenylacetic acids **2**–**5** evaluated by analysis of variance (ANOVA) and Tukey’s post hoc test. IC_50_ values (μM) are expressed as the mean ± standard deviation (n = 3). The ANOVA revealed statistically significant differences among groups (*p* < 0.001). Tukey’s test provided multiple pairwise comparisons of the means, identifying similarities and significant differences among compounds (*p* < 0.05). **3**, **4**, **32** (a) have significantly higher values than **2** (ef, *p* < 0.0001). **33** (d, diff = 29.33, *p* = 0.0000000), **39** (dg, diff = 25.97, *p* = 0.0000000), **40** (g, diff = 22.76, *p* = 0.0000000), and **42** (g, diff = 20.72, *p* = 0.0000000) differ significantly from other groups, confirming higher mean values. Samples **31** (c), **36** (ef), **37** (e), and **44** (cef) have lower mean values than samples with higher levels. Comparison between **36** (ef) and **3** (a) shows a difference of -45.27 with *p* < 0.0001, and **44** (cef) vs. **3** (a) has a difference of -42.98 (*p* < 0.0001). Most of the comparisons between different samples show very low *p*-values, indicating strong evidence of differences between the groups. Only a few comparisons, such as **38** vs. **2** (*p* = 0.073) and **44** vs. **31** (*p* = 0.177), do not reach the significance threshold (*p* < 0.05). Some comparisons, such as **36** vs. **2** (ef, diff = 0.03, *p* = 1.0), **37** (e) vs. **2** (ef) (diff = -0.33, *p* = 1.0), and **5** (b) vs. **34** (b, *p* = 1.0000000), **41** (b, diff = 0.69, *p* = 1.0000000), **43** (b, diff = -0.92336295, *p* = 0.9999995) are not significant, suggesting that these samples are not distinguishable each other.

**Figure 6:**
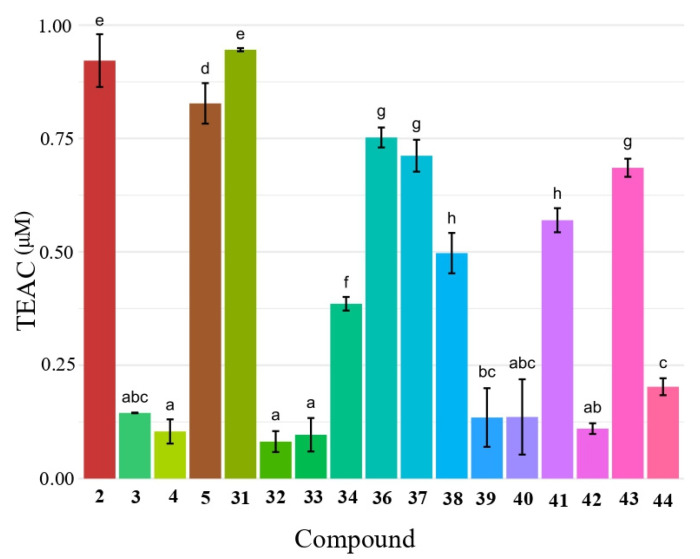
ABTS data of butyl diarylacetates **31**–**34** and **36**–**44,** compared with hydroxyphenylacetic acids **2**–**5,** evaluated by ANOVA followed by Tukey’s post hoc test for pairwise comparisons among the sample groups. TEAC values (μM) are expressed as the mean ± standard deviation (n = 3). Tukey’s test provides the *p*-values for each pairwise comparison to indicate where differences are statistically significant. Groups with different letters differ significantly from each other (*p* < 0.05), while groups with the same letter show no statistical differences. Hydroxyphenylacetic acid **2** is significantly different from other samples (*p* < 0.0001), except for **31** (e, *p* = 1). **3** (abc) is not significantly different from **4** (a, *p* = 0.667), **33** (a, *p* = 0.548), **40** (abc, *p* = 1), **42** (ab, *p* = 0.999). **4** (a) is significantly different from **44** (c, *p* = 0.0022). **36**, **37**, **43** (g) are not significantly different from each other (**37**–**36**: g, *p* = 0.999; **43**–**36**: g, *p* = 0.293; **43**–**37**: g, *p* = 0.918). **38** (h) is significantly different from almost all except for **41** (h, *p* = 0.871). **44** (c) is significantly different from **4** (a, *p* = 0.0022).

The DPPH and ABTS values of butyl diarylacetates **31**–**34** and **36**–**44** were also statistically analyzed and compared with hydroxyphenylacetic acids **2**–**5** using the post hoc Tukey test (Figure 5 and Figure 6). Data on compound **30** are not included for high DPPH and low ABTS values.

### 2.6. Qualitative Evaluation of the Bactericidal Properties of Hydroxyphenylacetic Acids ***1**–**5*** and Butyl Diarylacetates ***31***, ***36***, ***37***, and ***38*** Against Staphylococcus aureus and Escherichia coli

*Staphylococcus aureus* (*S. aureus*) is a Gram-positive bacterium that, commonly, is part of the normal human microbiota, residing primarily on the skin and mucous membranes, especially in the nasal area. While it typically does not cause infections on intact skin, if it enters the bloodstream or internal tissues, for example, through broken skin or a medical procedure, it can lead to various serious diseases, including infections of the heart valves (endocarditis), pneumonia, and bacteremia (bloodstream infection) [43]. Most strains of *S. aureus* are sensitive to commonly used antibiotics, and infections can be effectively treated, but some of them are more resistant and require different types of antibiotics [44]. *Escherichia coli* (*E. coli*) is a Gram-negative bacterium known to be a part of normal intestinal microbiota. When found outside of the intestinal tract, it can be the cause of intestinal and extraintestinal illness in humans, such as urinary tract infections, pneumonia, bacteremia, and peritonitis [44]. Treatment against *E. coli* is dependent on the strain, as well as the illness, varying from rehydration and administration of antimotility agents for mild diseases to antibiotics for severe infections [45,46].

In the literature, it was reported that 4-hydroxyphenylacetic acid **1** and 3,4-dihydroxyphenylacetic acid **2**, either from a commercial supply or extracted from natural sources, showed low to moderate inhibition against *S. aureus* and *E. coli* [47,48]. Structure–activity studies revealed enhanced antibacterial activity by increasing the lipophilicity of the phenolic acids, probably due to an increased membrane permeability [49,50]. Despite these data in the literature, ethyl ester of 4-hydroxyphenylacetic acid **1** has not shown significant improvements in efficacy against *S. aureus* and *E. coli* compared to its precursor [51].

In this study, butyl diarylacetates **31**, **36**, **37**, and **38**, having a catecholic moiety in their structure and a high lipophilicity (logP values ranging from 2.49 to 2.74), were selected for a preliminary evaluation of the bactericidal activity against *S. aureus* and *E. coli*, as examples of Gram-positive and Gram-negative bacteria. As reported in Table 5, with the exception of 4-hydroxyphenylacetic acid **1**, which was ineffective against *S. aureus* (entry 1), 3,4-dihydroxyphenylacetic acid **2**, 4-hydroxy-3-methoxyphenylacetic acid **3**, and 3-hydroxy-4-methoxyphenylacetic acid **4** showed antibacterial activity with an MBC of 10 mmol/L (entries 2–4) and 4-hydroxy-3,5-dimethoxyphenylacetic acid **5** with an MBC of 5 mmol/L (entry 5). All diesters **31**, **36**, **37**, and **38** showed antibacterial activity against *S. aureus*, with an MBC of 5.0 mmol/L for **31**, **36**, and **38** (entries 6,7, 9) and 10 mmol/l for **37** (entry 8). Against *E. coli*, only 3,4-dihydroxyphenylacetic acid **2** and 3-hydroxy-4-methoxyphenylacetic acid **4** were effective with an MBC of 10 mmol/L (entries 2 and 4), while all diesters **31**, **36**, **37**, and **38** displayed antibacterial activity with an MBC of 10 mmol/L (entries 6–9). Even if the antibacterial activity of the tested diesters against *S. aureus* and *E. coli* was lower than for gentamicin sulfate, which was used as a positive control (entry 10), the preliminary data of synthetized butyl diarylacetates **31**, **36**, **37**, and **38** seem promising for future applications.

## 3. Material and Methods

### 3.1. Reagents and Instruments

In this study, all reagents and solvents were purchased from Sigma Aldrich (Merck Group, Milan, Italy) or VWR International (Avantor, Milan, Italy) and used without further purification. Reaction products were purified by flash chromatography using silica gel (40–63 mm) as the stationary phase, eluting with a mixture of dichloromethane/methanol (from 98/2 to 90/10% *v*/*v*). ^1^H and ^13^C NMR were recorded with a 400 MHz Bruker Avance III spectrometer (Bruker Group, Fällanden, Switzerland). Splitting patterns are designed as s (singlet), d (doublet), t (triplet), q (quartet), m (multiplet), or bs (broad singlet). HRMS analysis was performed with a UHPLC system coupled with a Q-Exactive (Thermo, Waltham, MA, USA) mass spectrometer in the full MS mode (2 μ scans). The FT-IR spectra were recorded using a Jasco Europe Srl (Milan, Italy) FT/IR-6800 spectrometer equipped with an ATR (attenuated total reflectance) accessory. Spectra were collected in the range 4000–650 cm^−1^. Melting points were determined with a Falc Instruments MPD-03 apparatus (Treviglio, Italy). Antioxidant activities were determined using a UV-Vis spectrometer Shimadzu UV-2600 (Shimadzu Corporation, Duisburg, Germany).

### 3.2. Synthesis of Hydroxyalkyl Esters ***10***–***29*** by Fischer Esterification

In a round-bottomed flask equipped with a magnetic stirring bar, the hydroxyphenylacetic acid (3 mmol, 1 equiv.) was dissolved in appropriate diol (90 mmol, 30 equiv.) and the solution was heated to 90 °C. After 10 min, H_2_SO_4_ (29.4 mg, 16 μL, 0.3 mmol, 0.1 equiv.) was added and the mixture stirred until the disappearance of the starting material (0.5 to 5 h), as monitored by thin layer chromatography on silica gel (eluent from 70/30 to 50/50% *v*/*v* petroleum ether/ethyl acetate). After that, the reaction was diluted with ethyl acetate (150 mL) and washed with brine (2 × 20 mL). The organic layer was dried over Na_2_SO_4_, filtered, and concentrated under reduced pressure. The residue was purified by chromatography on silica gel (40–63 μm), eluting with a CH_2_Cl_2_/MeOH mixture to obtain the corresponding hydroxyalkyl esters **10**–**29**.

The simultaneous presence of the hydroxyl group and the ester group was confirmed by both IR measurements (bands above 3300 and 1700 cm^−1^, respectively, -OH and C=O stretching) and NMR experiments, while the introduction of the alkyl chain was observed mainly with NMR. The ^1^H NMR experiments, indeed, showed, for all hydroxyalkyl esters, two signals at about 4.1 ppm and 3.5 ppm, indicating methylene groups next to oxygen atoms that were not present in the starting hydroxyphenylacetic acids. The number of different methylene groups was also easily detected, considering the clear aliphatic region in the ^13^C experiments (see Supplementary Materials).

*2-Hydroxyethyl 2-(4-hydroxyphenyl) acetate* **10**. White solid, mp = 72–73 °C. FT-IR (neat): 3379, 3176, 1699, 1219, 1171, 531 cm^−1^; ^1^H NMR (400.13 MHz) (CD_3_OD) δ = 7.11 (d, *J* = 8.6 Hz, 2H, Ph-H), 6.74 (d, *J* = 8.6 Hz, 2H, Ph-H), 4.87 (bs, 2H, -OH), 4.17–4.14 (m, 2H, -CH_2_-O), 3.75–3.72 (m, 2H, -CH_2_-O), 3.58 (s, 2H, -COCH_2_-); ^13^C NMR (100.6 MHz) (CD_3_OD) δ = 172.8 (C), 156.1 (C), 130.0 (CH), 124.9 (C), 114.9 (CH), 65.8 (CH_2_), 59.6 (CH_2_), 39.6 (CH_2_). HRMS: *m*/*z* [M + H]+ calcd. for C_10_H_13_O_4_: 197.0814; found: 197.0805.
*4-Hydroxybutyl 2-(4-hydroxyphenyl)acetate* **11**. Pale yellow oil. FT-IR (neat): 3323, 2948, 1708, 1514, 1223, 1033 cm^−1^; ^1^H NMR (400.13 MHz) (CD_3_OD) δ = 7.09 (d, *J* = 8.6 Hz, 2H, Ph-H), 6.74 (d, *J* = 8.6 Hz, 2H, Ph-H), 4.88 (bs, 2H, -OH), 4.11 (t, *J* = 6.5 Hz, 2H, -CH_2_-O), 3.56 (t, *J* = 6.4 Hz, 2H, -CH_2_-O), 3.53 (s, 2H, -COCH_2_-), 1.73–1.66 (m, 2H, CH_2_-CH_2_-CH_2_), 1.60–1.53 (m, 2H, CH_2_-CH_2_-CH_2_); ^13^C NMR (100.6 MHz) (CD_3_OD) δ = 172.7 (C), 156.2 (C), 129.9 (CH), 125.0 (C), 114.9 (CH), 64.3 (CH_2_), 61.0 (CH_2_), 39.8 (CH_2_), 28.6 (CH_2_), 24.9 (CH_2_); HRMS: *m*/*z* [M + H]+ calcd. for C_12_H_17_O_4_: 225.1127; found: 225.1116.
*6-Hydroxyhexyl 2-(4-hydroxyphenyl)acetate* **12**. White solid, mp = 67–68 °C. FT-IR (neat): 3440, 3141, 1713, 1220, 1170, 987 cm^−1^; ^1^H NMR (400.13 MHz) (CD_3_OD) δ = 7.09 (d, *J* = 8.6 Hz, 2H, Ph-H), 6.74 (d, *J* = 8.6 Hz, 2H, Ph-H), 4.88 (s, 2H, -OH), 4.09 (t, *J* = 6.6 Hz, 2H, -CH_2_-O), 3.56–3.53 (m, 4H), 1.67–1.60 (m, 2H, R-CH_2_-R), 1.56–1.49 (m, 2H, CH_2_-CH_2_-CH_2_), 1.41–1.31 (m, 4H, CH_2_-CH_2_-CH_2_); ^13^C NMR (100.6 MHz) (CD_3_OD) δ = 172.8 (C), 156.2 (C), 129.9 (CH), 125.1 (C), 114.9 (CH), 64.4 (CH_2_), 61.4 (CH_2_), 39.9 (CH_2_), 32.1 (CH_2_), 28.3 (CH_2_), 25.4 (CH_2_), 25.1 (CH_2_); HRMS: *m*/*z* [M + H]+ calcd. for C_14_H_21_O_4_: 253.1440; found: 253.1428.
*8-Hydroxyoctyl 2-(4-hydroxyphenyl)acetate* **13**. White wax. FT-IR (neat): 3325, 2927, 2853, 1728, 1173, 1028 cm^−1^; ^1^H NMR (400.13 MHz) (CD_3_OD) δ = 7.09 (d, *J* = 8.4 Hz, 2H, Ph-H), 6.74 (d, *J* = 8.4 Hz, 2H, Ph-H), 4.88 (s, 2H, -OH), 4.08 (t, *J* = 6.6 Hz, 2H, -CH_2_-O), 3.56 (t, *J* = 6.6 Hz, 2H, -CH_2_-O), 3.52 (s, 2H, -COCH_2_-), 1.63–1.60 (m, 2H, CH_2_-CH_2_-CH_2_), 1.57–1.50 (m, 2H, CH_2_-CH_2_-CH_2_), 1.36–1.27 (m, 8H, CH_2_-CH_2_-CH_2_); ^13^C NMR (100.6 MHz) (CD_3_OD) δ = 172.8 (C), 156.2 (C), 129.9 (CH), 125.1 (C), 114.9 (CH), 64.5 (CH_2_), 61.6 (CH_2_), 39.9 (CH_2_), 32.2 (CH_2_), 29.1 (CH_2_), 28.9 (CH_2_), 28.3 (CH_2_), 25.5 (CH_2_), 25.4 (CH_2_); HRMS: *m*/*z* [M + H]+ calcd. for C_16_H_25_O_4_: 281.1753; found: 281.1738.
*2-Hydroxyethyl 2-(3,4-dihydroxyphenyl)acetate* **14**. Pale pink solid, mp = 80–81 °C. FT-IR (neat): 3420, 3221, 1698, 1447, 1115 cm^−1^; ^1^H NMR (400.13 MHz) (CD_3_OD) δ = 6.74 (d, *J* = 2.1 Hz, 1H, Ph-H), 6.71 (d, *J* = 8.1 Hz, 1H, Ph-H), 6.60 (dd, *J*_1_ = 8.1 Hz, *J*_2_ = 2.1 Hz, 1H, Ph-H), 4.88 (s, 3H, -OH), 4.17–4.14 (m, 2H, -CH_2_-O), 3.75–3.73 (m, 2H, -CH_2_-O), 3.52 (s, 2H, -COCH_2_-); ^13^C NMR (100.6 MHz) (CD_3_OD) δ = 172.8 (C), 144.9 (C), 144.1 (C), 125.5 (C), 120.3 (CH), 116.0 (CH), 114.9 (CH), 65.8 (CH_2_), 59.6 (CH_2_), 39.8 (CH_2_); HRMS: *m*/*z* [M + H]+ calcd. for C_10_H_13_O_5_: 213.0763; found: 213.0753.
*4-Hydroxybutyl 2-(3,4-dihydroxyphenyl)acetate* **15**. Yellow oil. FT-IR (neat): 3329, 2947, 2362, 1708, 1519, 1285 cm^−1^; ^1^H NMR (400.13 MHz) (CD_3_OD) δ = 6.73–6.70 (m, 2H, Ph-H), 6.59 (dd, *J*_1_ = 8.0 Hz, *J*_2_ = 2.1 Hz, 1H, Ph-H), 4.88 (s, 3H, -OH), 4.1 (t, *J* = 6.5 Hz, 2H, -CH_2_-O), 3.56 (t, *J* = 6.5 Hz, 2H, -CH_2_-O), 3.47 (s, 2H, -COCH_2_-), 1.74–1.67 (m, 2H, CH_2_-CH_2_-CH_2_), 1.60–1.53 (m, 2H, CH_2_-CH_2_-CH_2_); ^13^C NMR (100.6 MHz) (CD_3_OD) δ = 172.7 (C), 144.9 (C), 144.0 (C), 125.6 (C), 120.2 (CH), 115.9 (CH), 114.9 (CH), 64.3 (CH_2_), 61.0 (CH_2_), 40.1 (CH_2_), 28.6 (CH_2_), 24.9 (CH_2_); HRMS: *m*/*z* [M + H]+ calcd. for C_12_H_17_O_5_: 241.1076; found: 241.1065.
*6-Hydroxyhexyl 2-(3,4-dihydroxyphenyl)acetate* **16**. Orange oil. FT-IR (neat): 3363, 2935, 1709, 1519, 1284 cm^−1^; ^1^H NMR (400.13 MHz) (CD_3_OD) δ = 6.72–6.70 (m, 2H, Ph-H), 6.60–6.58 (m, 1H, Ph-H), 4.87 (s, 3H, -OH), 4.09 (t, *J* = 6.5 Hz, 2H, -CH_2_-O), 3.54 (t, *J* = 6.5 Hz, 2H, -CH_2_-O), 3.46 (s, 2H, COCH_2_-), 1.64–1.61 (m, 2H, CH_2_-CH_2_-CH_2_), 1.53–1.51 (m, 2H, CH_2_-CH_2_-CH_2_), 1.37–1.31 (m, 4H, CH_2_-CH_2_-CH_2_); ^13^C NMR (100.6 MHz) (CD_3_OD) δ = 172.8 (C), 144.9 (C), 144.0 (C), 125.7 (C), 120.2 (CH), 115.9 (CH), 114.9 (CH), 64.4 (CH_2_), 61.5 (CH_2_), 40.2 (CH_2_), 32.1 (CH_2_), 28.3 (CH_2_), 25.4 (CH_2_), 25.1 (CH_2_); HRMS: *m*/*z* [M + H]+ calcd. for C_14_H_21_O_5_: 269.1389; found: 269.1375.
*8-Hydroxyoctyl 2-(3,4-dihydroxyphenyl)acetate* **17**. Purified with semi-preparative RP-HPLC, Teknokroma column Mediterranea SEA18 5mm (25 × 0.78 cm) using CH3CN/H_2_O 50/50 *v*/*v* as mobile phase with flow rate 4 mL/min. Pale yellow wax. FT-IR (neat): 3387, 2924, 2852, 1707, 1354, 1194 cm^−1^; ^1^H NMR (400.13 MHz) (CD_3_OD) δ = 6.73–6.70 (m, 2H, Ph-H), 6.59 (dd, *J*_1_ = 8.0 Hz, *J*_2_ = 2.0 Hz, 1H, Ph-H), 4.86 (bs, 3H, -OH), 4.08 (t, *J* = 6.5 Hz, 2H, -CH_2_-O), 3.56 (t, *J* = 6.5 Hz, 2H, -CH_2_-O), 3.46 (s, 2H, COCH_2_-), 1.63–1.50 (m, 4H, CH_2_-CH_2_-CH_2_), 1.32 (m, 8H, CH_2_-CH_2_-CH_2_); ^13^C NMR (100.6 MHz) (CD_3_OD) δ = 172.8 (C), 144.9 (C), 144.0 (C), 125.7 (C), 120.2 (CH), 115.9 (CH), 114.9 (CH), 64.5 (CH_2_), 61.6 (CH_2_), 40.2 (CH_2_), 32.2 (CH_2_), 29.0 (CH_2_), 28.9 (CH_2_), 28.3 (CH_2_), 25.5 (CH_2_), 25.4 (CH_2_); HRMS: *m*/*z* [M + H]+ calcd. for C_16_H_25_O_5_: 297.1702; found: 297.1687.
*2-Hydroxyethyl 2-(4-hydroxy-3-methoxyphenyl)acetate* **18**. Orange oil. FT-IR (neat): 3420, 2942, 1719, 1516, 1275, 1154 cm^−1^; ^1^H NMR (400.13 MHz) (CD_3_OD) δ = 6.89 (d, *J* = 1.6 Hz, 1H, Ph-H), 6.76–6.71 (m, 2H, Ph-H), 4.87 (s, 2H, -OH), 4.18–4.16 (m, 2H, -CH_2_-O), 3.85 (s, 3H, -OCH_3_), 3.76–3.73 (m, 2H, -CH_2_-O), 3.59 (s, 2H, COCH_2_-); ^13^C NMR (100.6 MHz) (CD_3_OD) δ = 172.7 (C), 147.5 (C), 145.3 (C), 125.5 (C), 121.6 (CH), 114.7 (CH), 112.6 (CH), 65.9 (CH_2_), 59.6 (CH_2_), 55.0 (CH_3_), 39.9 (CH_2_); HRMS: *m*/*z* [M + H]+ calcd. for C_11_H_15_O_5_: 227.0919; found: 227.0908.
*4-Hydroxybutyl 2-(4-hydroxy-3-methoxyphenyl)acetate* **19**. Colorless oil. FT-IR (neat): 3423, 2940, 1719, 1516, 1273, 1153 cm^−1^; ^1^H NMR (400.13 MHz) (CD_3_OD) δ = 6.87 (d, *J* = 1.9 Hz, 1H, Ph-H), 6.76–6.69 (m, 2H, Ph-H), 4.87 (s, 2H, -OH), 4.12 (t, *J* = 6.4 Hz, 2H, -CH_2_-O), 3.85 (s, 3H, -OCH_3_), 3.56 (t, *J* = 6.4 Hz, 2H, -CH_2_-O), 3.55 (s, 2H, COCH_2_-), 1.74–1.67 (m, 2H, CH_2_-CH_2_-CH_2_), 1.61–1.53 (m, 2H, CH_2_-CH_2_-CH_2_); ^13^C NMR (100.6 MHz) (CD_3_OD) δ = 172.7 (C), 147.5 (C), 145.3 (C), 125.6 (C), 121.5 (CH), 114.8 (CH), 112.5 (CH), 64.4 (CH_2_), 61.0 (CH_2_), 55.0 (CH_3_), 40.2 (CH_2_), 28.6 (CH_2_), 24.9 (CH_2_); HRMS: *m*/*z* [M + H]+ calcd. for C_13_H_19_O_5_: 255.1232; found: 255.1222.
*6-Hydroxyhexyl 2-(4-hydroxy-3-methoxyphenyl)acetate* **20**. Colorless oil. FT-IR (neat): 3356, 2934, 1715, 1514, 1271, 1149 cm^−1^; ^1^H NMR (400.13 MHz) (CD_3_OD) δ = 6.86 (d, *J* = 1.9 Hz, 1H, Ph-H), 6.76–6.69 (m, 2H, Ph-H), 4.87 (s, 2H, -OH), 4.10 (t, *J* = 6.5 Hz, 2H, -CH_2_-O), 3.85 (s, 3H, -OCH_3_), 3.56–3.52 (m, 4H), 1.67–1.60 (m, 2H, CH_2_-CH_2_-CH_2_), 1.55–1.48 (m, 2H, CH_2_-CH_2_-CH_2_), 1.40–1.31 (m, 4H, CH_2_-CH_2_-CH_2_); ^13^C NMR (100.6 MHz) (CD_3_OD) δ = 172.7 (C), 147.5 (C), 145.3 (C), 125.6 (C), 121.5 (CH), 114.8 (CH), 112.5 (CH), 64.5 (CH_2_), 61.4 (CH_2_), 55.0 (CH_3_), 40.3 (CH_2_), 32.1 (CH_2_), 28.3 (CH_2_), 25.4 (CH_2_), 25.1 (CH_2_); HRMS: *m*/*z* [M + H]+ calcd. for C_15_H_23_O_5_: 283.1545; found: 283.1532.
*8-Hydroxyoctyl 2-(4-hydroxy-3-methoxyphenyl)acetate* **21**. Pale yellow oil. FT-IR (neat): 3391, 2929, 2854, 1724, 1516, 1275, 1152 cm^−1^; ^1^H NMR (400.13 MHz) (CD_3_OD) δ = 6.86 (d, *J* = 1.4 Hz, 1H, Ph-H), 6.76–6.69 (m, 2H, Ph-H), 4.85 (s, 2H, -OH), 4.09 (t, *J* = 6.5 Hz, 2H, -CH_2_-O), 3.85 (s, 3H, -OCH_3_), 3.55 (t, *J* = 6.7 Hz, 2H, -CH_2_-O), 3.53 (s, 2H, COCH_2_-), 1.63–1.58 (m, 2H, CH_2_-CH_2_-CH_2_), 1.55–1.50 (m, 2H, CH_2_-CH_2_-CH_2_), 1.31 (bs, 8H, CH_2_-CH_2_-CH_2_); ^13^C NMR (100.6 MHz) (CD_3_OD) δ = 172.7 (C), 147.5 (C), 145.3 (C), 125.6 (C), 121.5 (CH), 114.8 (CH), 112.5 (CH), 64.5 (CH_2_), 61.6 (CH_2_), 55.0 (CH_3_), 40.3 (CH_2_), 32.2 (CH_2_), 29.1 (CH_2_), 28.9 (CH_2_), 28.3 (CH_2_), 25.5 (CH_2_), 25.4 (CH_2_); HRMS: *m*/*z* [M + H]+ calcd. for C_17_H_27_O_5_: 311.1858; found: 311.1841.
*2-Hydroxyethyl 2-(3-hydroxy-4-methoxyphenyl)acetate* **22**. Pale pink solid, mp = 63–64 °C. FT-IR (neat): 3387, 3246, 1697, 1509, 1216 cm^−1^; ^1^H NMR (400.13 MHz) (CD_3_OD) δ = 6.86 (d, *J* = 8.2 Hz, 1H, Ph-H), 6.77 (bs, 1H, Ph-H), 6.72 (dd, *J*_1_ = 8.2 Hz, *J*_2_ = 1.6 Hz, 1H, Ph-H), 4.89 (s, 2H, -OH), 4.16 (t, *J* = 4.9 Hz, 2H, -CH_2_-O), 3.84 (s, 3H, -OCH_3_), 3.74 (t, *J* = 4.9 Hz, 2H, -CH_2_-O), 3.55 (s, 2H, COCH_2_-); ^13^C NMR (100.6 MHz) (CD_3_OD) δ = 172.5 (C), 146.8 (C), 146.1 (C), 127.0 (C), 120.2 (CH), 116.0 (CH), 111.4 (CH), 65.9 (CH_2_), 59.6 (CH_2_), 55.0 (CH_3_), 39.8 (CH_2_); HRMS: *m*/*z* [M + H]+ calcd. for C_11_H_15_O_5_: 227.0919; found: 227.0908.
*4-Hydroxybutyl 2-(3-hydroxy-4-methoxyphenyl)acetate* **23**. Light yellow oil. FT-IR (neat): 3397, 2939, 1715, 1511, 1274 cm^−1^; ^1^H NMR (400.13 MHz) (CD_3_OD) δ = 6.86 (d, *J* = 8.1 Hz, 1H, Ph-H), 6.76 (s, 1H, Ph-H), 6.71 (d, *J* = 8.1 Hz, 1H, Ph-H), 4.87 (s, 2H, -OH), 4.12 (t, *J* = 6.3 Hz, 2H, -CH_2_-O), 3.84 (s, 3H, -OCH_3_), 3.56 (t, *J* = 6.3 Hz. 2H, -CH_2_-O), 3.51 (s, 2H, COCH_2_-), 1.74–1.67 (m, 2H, CH_2_-CH_2_-CH_2_), 1.60–1.53 (m, 2H, CH_2_-CH_2_-CH_2_); ^13^C NMR (100.6 MHz) (CD_3_OD) δ = 172.5 (C), 146.8 (C), 146.2 (C), 127.1 (C), 120.1 (CH), 115.9 (CH), 111.4 (CH), 64.4 (CH_2_), 61.0 (CH_2_), 55.0 (CH_3_), 40.1 (CH_2_), 28.6 (CH_2_), 24.9 (CH_2_); HRMS: *m*/*z* [M + H]+ calcd. for C_13_H_19_O_5_: 255.1232; found: 255.1221.
*6-Hydroxyhexyl 2-(3-hydroxy-4-methoxyphenyl)acetate* **24**. Pale yellow oil. FT-IR (neat): 3402, 2933, 2857, 1717, 1511, 1273 cm^−1^; ^1^H NMR (400.13 MHz) (CD_3_OD) δ = 6.86 (d, *J* = 8.2 Hz, 1H, Ph-H), 6.76 (bs, 1H, Ph-H), 6.71 (d, *J* = 8.2 Hz, 1H, Ph-H), 4.87 (s, 2H, -OH), 4.09 (t, *J* = 6.4 Hz, 2H, -CH_2_-O), 3.84 (s, 3H, -OCH_3_), 3.54 (t, *J* = 6.4 Hz, 2H, -CH_2_-O), 3.50 (s, 2H, COCH_2_-), 1.65–1.62 (m, 2H, CH_2_-CH_2_-CH_2_), 1.53–1.51 (m, 2H, CH_2_-CH_2_-CH_2_), 1.36 (bs, 4H, CH_2_-CH_2_-CH_2_); ^13^C NMR (100.6 MHz) (CD_3_OD) δ = 172.5, 146.8, 146.2, 127.1, 120.1 (CH), 115.9 (CH), 111.3 (CH), 64.4 (CH_2_), 61.4 (CH_2_), 55.0 (CH_3_), 40.2 (CH_2_), 32.1 (CH_2_), 28.3 (CH_2_), 25.4 (CH_2_), 25.1 (CH_2_); HRMS: *m*/*z* [M + H]+ calcd. for C_15_H_23_O_5_: 283.1545; found: 283.1532.
*8-Hydroxyoctyl 2-(3-hydroxy-4-methoxyphenyl)acetate* **25**. Light yellow oil. FT-IR (neat): 3400, 2929, 2855, 1725, 1512, 1275 cm^−1^; ^1^H NMR (400.13 MHz) (CD_3_OD) δ = 6.86 (d, *J* = 8.2 Hz, 1H, Ph-H), 6.76 (d, *J* = 2.0 Hz, 1H, Ph-H), 6.71 (dd, *J*_1_ = 8.2 Hz, *J*_2_ = 2.0 Hz, 1H, Ph-H), 4.85 (s, 2H, -OH), 4.09 (t, *J* = 6.5 Hz, 2H, -CH_2_-O), 3.84 (s, 3H, -OCH_3_), 3.55 (t, *J* = 6.5 Hz, 2H, -CH_2_-O), 3.50 (s, 2H, COCH_2_-), 1.63–1.60 (m, 2H, CH_2_-CH_2_-CH_2_), 1.55–1.50 (m, 2H, CH_2_-CH_2_-CH_2_), 1.32 (bs, 8H, CH_2_-CH_2_-CH_2_); ^13^C NMR (100.6 MHz) (CD_3_OD) δ = 172.6 (C), 146.8 (C), 146.2 (C), 127.1 (C), 120.1 (CH), 115.9 (CH), 111.4 (CH), 64.5 (CH_2_), 61.6 (CH_2_), 55.0 (CH_3_), 40.2 (CH_2_), 32.2 (CH_2_), 29.0 (CH_2_), 28.8 (CH_2_), 28.3 (CH_2_), 25.5 (CH_2_), 25.4 (CH_2_); HRMS: *m*/*z* [M + H]+ calcd. for C_17_H_27_O_5_: 311.1858; found: 311.1841.
*2-Hydroxyethyl 2-(4-hydroxy-3,5-dimethoxyphenyl)acetate* **26**. Pale yellow solid, mp = 72–73 °C. FT-IR (neat): 3482, 3358, 2938, 1697, 1515, 1447 cm^−1^; ^1^H NMR (400.13 MHz) (CD_3_OD) δ = 6.59 (s, 2H, Ph-H), 4.87 (s, 2H, -OH), 4.19–4.16 (m, 2H), 3.84 (s, 6H, -OCH_3_), 3.76–3.74 (m, 2H), 3.60 (s, 2H, COCH_2_-); ^13^C NMR (100.6 MHz) (CD_3_OD) δ = 172.6 (C), 147.8 (C), 134.3 (C), 124.7 (C), 106.3 (CH), 65.9 (CH_2_), 59.6 (CH_2_), 55.3 (CH_3_), 40.3 (CH_2_); HRMS: *m*/*z* [M + H]+ calcd. for C_12_H_17_O_6_: 257.1025; found: 257.1011.
*4-Hydroxybutyl 2-(4-hydroxy-3,5-dimethoxyphenyl)acetate* **27**. Pale yellow solid, mp = 79–80 °C. FT-IR (neat): 3482, 3098, 2960, 1731, 1113 cm^−1^; ^1^H NMR (400.13 MHz) (CD_3_OD) δ = 6.57 (s, 2H, Ph-H), 4.87 (s, 2H, -OH), 4.14 (t, *J* = 6.3 Hz, 2H, -CH_2_-O), 3.84 (s, 6H, -OCH_3_), 3.58–3.56 (m, 4H), 1.75–1.68 (m, 2H, CH_2_-CH_2_-CH_2_), 1.61–1.54 (m, 2H, CH_2_-CH_2_-CH_2_); ^13^C NMR (100.6 MHz) (CD_3_OD) δ = 172.5 (C), 147.8 (C), 134.3 (C), 124.8 (C), 106.2 (CH), 64.4 (CH_2_), 61.0 (CH_2_), 55.3 (CH_3_), 40.6 (CH_2_), 28.6 (CH_2_), 24.9 (CH_2_); HRMS: *m*/*z* [M + H]+ calcd. for C_14_H_21_O_6_: 285.1338; found: 285.1326.
*6-Hydroxyhexyl 2-(4-hydroxy-3,5-dimethoxyphenyl)acetate* **28**. Yellow solid, mp = 77–78 °C. FT-IR (neat): 3477, 2936, 1726, 1115 cm^−1^; ^1^H NMR (400.13 MHz) (CD_3_OD) δ = 6.57 (s, 2H, Ph-H), 4.87 (s, 2H, -OH), 4.11 (t, *J* = 6.5 Hz, 2H, -CH_2_-O), 3.84 (s, 6H, -OCH_3_), 3.55 (s, 2H, COCH_2_-), 3.54 (t, *J* = 6.6 Hz, 2H, -CH_2_-O), 1.68–1.61 (m, 2H, CH_2_-CH_2_-CH_2_), 1.55–1.48 (m, 2H, CH_2_-CH_2_-CH_2_), 1.40–1.31 (m, 4H, CH_2_-CH_2_-CH_2_); ^13^C NMR (100.6 MHz) (CD_3_OD) δ = 172.6 (C), 147.8 (C), 134.3 (C), 124.9 (C), 106.2 (CH), 64.5 (CH_2_), 61.4 (CH_2_), 55.4 (CH_3_), 40.7 (CH_2_), 32.1 (CH_2_), 28.3 (CH_2_), 25.4 (CH_2_), 25.1 (CH_2_); HRMS: *m*/*z* [M + H]+ calcd. for C_16_H_25_O_6_: 313.1651; found: 313.1635.
*8-Hydroxyoctyl 2-(4-hydroxy-3,5-dimethoxyphenyl)acetate* **29**. Yellow solid, mp = 72–73 °C. FT-IR (neat): 3479, 2934, 1729, 1116 cm^−1^; ^1^H NMR (400.13 MHz) (CD_3_OD) δ = 6.57 (s, 2H, Ph-H), 4.87 (s, 2H, -OH), 4.10 (t, *J* = 6.5 Hz, 2H, -CH_2_-O), 3.84 (s, 6H, -OCH_3_), 3.57–3.53 (m, 4H), 1.64–1.60 (m, 2H, CH_2_-CH_2_-CH_2_), 1.55–1.50 (m, 2H, CH_2_-CH_2_-CH_2_), 1.32 (bs, 8H, CH_2_-CH_2_-CH_2_); ^13^C NMR (100.6 MHz) (CD_3_OD) δ = 172.6 (C), 147.8 (C), 134.3 (C), 124.9 (C), 106.2 (CH), 64.5 (CH_2_), 61.6 (CH_2_), 55.4 (CH_3_), 40.7 (CH_2_), 32.2 (CH_2_), 29.1 (CH_2_), 28.9 (CH_2_), 28.3 (CH_2_), 25.6 (CH_2_), 25.4 (CH_2_); HRMS: *m*/*z* [M + H]+ calcd. for C_18_H_29_O_6_: 341.1964; found: 341.1951.

### 3.3. Synthesis of Butyl Diarylacetates ***30***–***34*** and ***36***–***44*** by Mitsunobu Reaction

In a 10 mL two-necked, round-bottomed flask equipped with a magnetic stirring bar, the hydroxybutyl ester (0.45 mmol, 1 equiv.), hydroxyphenylacetic acid (0.45 mmol, 1 equiv.), and triphenylphosphine (PPh_3_, 129.8 mg, 0.495 mmol, 1.1 equiv.) were dissolved in tetrahydrofuran (THF, 1 mL) under inert atmosphere (N_2_). Then, a solution of diisopropyl azodicarboxylate (DIAD, 115.1 μL, 118.3 mg, 0.585 mmol, 1.3 equiv.) in THF (1 mL) was added dropwise. After two hours, the solvent was evaporated under reduced pressure, and the residue was purified by chromatography on silica gel (40–63 μm), eluting with a petroleum ether/ethyl acetate mixture to afford the expected butyl diarylacetates.

The IR spectra of the butyl diarylacetates maintained similar features to those of the hydroxyalkylaryl acetates, and the main differences in the chemical characterization were derived from NMR experiments. The ^1^H NMR experiments showed an increased number of aromatic protons, as well as pattern substitution, on the phenolic rings. The ^13^C NMR experiments showed the newly formed ester moiety as two close peaks at about 172 ppm, with the obvious exception of compounds showing symmetry (see Supplementary Materials for all spectra).

*Butane-1,4-diyl bis(2-(4-hydroxyphenyl)acetate)* **30**. White solid, mp = 120–121 °C; spectroscopic data are in accordance with those reported in the literature [35].
*4-(2-(3,4-Dihydroxyphenyl)acetoxy)butyl 2-(4-hydroxyphenyl)acetate* **31**. Pale brown solid, mp = 124–125 °C. FT-IR (neat): 3394, 2964, 1706, 1610, 1517, 1171 cm^−1^; ^1^H NMR (400.13 MHz) (CD_3_OD) δ = 7.09 (d, *J* = 8.4 Hz, 2H, Ph-H), 6.75–6.70 (m, 4H, Ph-H), 6.58 (dd, *J*_1_ = 8.1 Hz, *J*_2_ = 1.9 Hz, 1H, Ph-H), 4.87 (s, 3H, -OH), 4.07 (m, 4H, -CH_2_-O), 3.52 (s, 2H, COCH_2_-), 3.46 (s, 2H, COCH_2_-), 1.64 (m, 4H, CH_2_-CH_2_-CH_2_); ^13^C NMR (100.6 MHz) (CD_3_OD) δ = 172.71 (C), 172.66 (C), 156.1 (C), 144.9 (C), 144.1 (C), 129.9 (CH), 125.6 (C), 125.0 (C), 120.2 (CH), 115.9 (CH), 114.93 (CH), 114.89 (CH), 63.97 (CH_2_), 63.93 (CH_2_), 40.1 (CH_2_), 39.8 (CH_2_), 24.88 (CH_2_), 24.86 (CH_2_); HRMS: *m*/*z* [M + H]+ calcd. for C_20_H_23_O_7_: 375.1444; found: 375.1431.
*4-(2-(4-Hydroxy-3-methoxyphenyl)acetoxy)butyl 2-(4-hydroxyphenyl)acetate* **32**. Yellow oil. FT-IR (neat): 3402, 2959, 1709, 1514, 1149 cm^−1^; ^1^H NMR (400.13 MHz) (CDCl_3_) δ = 7.12 (d, *J* = 8.6 Hz, 2H, Ph-H), 6.87 (d, *J* = 8.0 Hz, 1H, Ph-H), 6.82–6.74 (m, 4H, Ph-H), 5.79 (bs, 1H, -OH), 5.69 (s, 1H, -OH), 4.12–4.09 (m, 4H, -CH_2_-O), 3.87 (s, 3H, -OCH_3_), 3.55 (s, 2H, COCH_2_-), 3.54 (s, 2H, COCH_2_-), 1.68–1.65 (m, 4H, CH_2_-CH_2_-CH_2_); ^13^C NMR (100.6 MHz) (CDCl_3_) δ = 172.3 (C), 172.2 (C), 155.0 (C), 146.5 (C), 144.7 (C), 130.4 (CH), 125.83 (C), 125.77 (C), 122.1 (CH), 115.5 (CH), 114.5 (CH), 111.8 (CH), 64.4 (CH_2_), 64.3 (CH_2_), 55.9 (CH_3_), 41.0 (CH_2_), 40.5 (CH_2_), 25.2 (2× CH_2_); HRMS: *m*/*z* [M + H]+ calcd. for C_21_H_25_O_7_: 389.1600; found: 389.1580.
*4-(2-(3-Hydroxy-4-methoxyphenyl)acetoxy)butyl 2-(4-hydroxyphenyl)acetate* **33**. Yellow wax. FT-IR (neat): 3394, 2960, 1710, 1512, 1130, 1027 cm^−1^; ^1^H NMR (400.13 MHz) (CDCl_3_) δ = 7.15–7.13 (m, 2H, Ph-H), 6.88 (d, *J* = 2.0 Hz, 1H, Ph-H), 6.83–6.76 (m, 4H, Ph-H), 5.74 (m, 1H, -OH), 5.56 (m, 1H, -OH), 4.12–4.06 (m, 4H, -CH_2_-O), 3.89 (s, 3H, -OCH_3_), 3.55 (s, 2H, COCH_2_-), 3.52 (s, 2H, COCH_2_-), 1.66–1.65 (m, 4H, CH_2_-CH_2_-CH_2_); ^13^C NMR (100.6 MHz) (CDCl_3_) δ = 172.1 (C), 171.9 (C), 154.9 (C), 145.8 (C), 145.5 (C), 130.4 (CH), 127.2 (C), 126.0 (C), 120.9 (CH), 115.52 (CH), 115.49 (CH), 110.8 (CH), 64.35 (CH_2_), 64.32 (CH_2_), 56.0 (CH_3_), 40.8 (CH_2_), 40.6 (CH_2_), 25.22 (CH_2_), 25.17 (CH_2_); HRMS: *m*/*z* [M + H]+ calcd. for C_21_H_25_O_7_: 389.1600; found: 389.1577.
*4-(2-(4-Hydroxy-3,5-dimethoxyphenyl)acetoxy)butyl 2-(4-hydroxyphenyl)acetate* **34**. Colorless oil. FT-IR (neat): 3360, 2939, 1726, 1714, 1117 cm^−1^; ^1^H NMR (400.13 MHz) (CDCl_3_) δ = 7.12 (d, *J* = 8.5 Hz, 2H, Ph-H), 6.76 (d, *J* = 8.5 Hz, 2H, Ph-H), 6.53 (s, 2H, Ph-H), 5.69 (bs, 1H, -OH), 5.54 (s, 1H, -OH), 4.10 (m, 4H, -CH_2_-O), 3.88 (s, 6H, -OCH_3_), 3.54 (m, 4H, CH_2_-CH_2_-CH_2_); ^13^C NMR (100.6 MHz) (CDCl_3_) δ = 172.1 (C), 171.9 (C), 155.0 (C), 147.0 (C), 133.9 (C), 130.4 (CH), 125.8 (C), 125.0 (C), 115.5 (CH), 106.1 (CH), 64.4 (CH_2_), 64.3 (CH_2_), 56.3 (CH_3_), 41.5 (CH_2_), 40.5 (CH_2_), 25.2 (2x CH_2_); HRMS: *m*/*z* [M + H]+ calcd. for C_22_H_27_O_8_: 419.1706; found: 419.1681.
*4-(2-(3,4-Dihydroxyphenyl)acetoxy)butyl 2-(4-hydroxy-3-methoxyphenyl)acetate* **36**. Yellow wax. FT-IR (neat): 3339, 2939, 1713, 1515, 1148 cm^−1^; ^1^H NMR (400.13 MHz) (CDCl_3_) δ = 6.86 (d, *J* = 8.0 Hz, 1H, Ph-H), 6.80–6.75 (m, 3H, Ph-H), 6.65–6.64 (m, 2H, Ph-H), 6.16 (bs, 1H, -OH), 5.84 (bs, 1H, -OH), 4.13–4.08 (m, 4H, -CH_2_-O), 3.84 (s, 3H, -OCH_3_), 3.56 (s, 2H, COCH_2_-), 3.47 (s, 2H, COCH_2_-), 1.68–1.66 (m, 4H, CH_2_-CH_2_-CH_2_); ^13^C NMR (100.6 MHz) (CDCl_3_) δ = 172.8 (C), 172.6 (C), 146.6 (C), 144.7 (C), 143.8 (C), 143.4 (C), 126.1 (C), 125.6 (C), 122.1 (CH), 121.6 (CH), 116.2 (CH), 115.3 (CH), 114.6 (CH), 111.9 (CH), 64.8 (CH_2_), 64.5 (CH_2_), 55.9 (CH_3_), 41.1 (CH_2_), 40.8 (CH_2_), 25.2 (CH_2_), 25.0 (CH_2_); HRMS: *m*/*z* [M + H]+ calcd. for C_21_H_25_O_8_: 405.1549; found: 405.1529.
*2-(2-(3,4-Dihydroxyphenyl)acetoxy)ethyl 2-(3-hydroxy-4-methoxyphenyl)acetate* **37**. Orange wax. FT-IR (neat): 3391, 1706, 1510, 1131, 762 cm^−1^; ^1^H NMR (400.13 MHz) (CDCl_3_) δ = 6.87 (d, *J* = 1.9 Hz, 1H, Ph-H), 6.81–6.76 (m, 3H, Ph-H), 6.65 (dd, *J*_1_ = 8.0 Hz, *J*_2_ = 2.0 Hz, 1H, Ph-H), 6.61 (bs, 1H, Ph-H), 6.07 (bs, 1H, -OH), 5.91 (bs, 1H, -OH), 4.11–4.09 (m, 4H, -CH_2_-O), 3.86 (s, 3H, -OCH_3_), 3.54 (s, 2H, COCH_2_-), 3.49 (s, 2H, COCH_2_-), 1.68–1.66 (m, 4H, CH_2_-CH_2_-CH_2_); ^13^C NMR (100.6 MHz) (CDCl_3_) δ = 172.7 (C), 172.6 (C), 145.9 (C), 145.5 (C), 143.8 (C), 143.4 (C), 126.9 (C), 126.1 (C), 121.6 (CH), 121.0 (CH), 116.2 (CH), 115.6 (CH), 115.3 (CH), 110.9 (CH), 64.8 (CH_2_), 64.6 (CH_2_), 56.0 (CH_3_), 40.9 (CH_2_), 40.8 (CH_2_), 25.3 (CH_2_), 25.0 (CH_2_); HRMS: *m*/*z* [M + H]+ calcd. for C_21_H_25_O_8_: 405.1549; found: 405.1530.
*4-(2-(3,4-Dihydroxyphenyl)acetoxy)butyl 2-(4-hydroxy-3,5-dimethoxyphenyl)acetate* **38**. White solid, mp = 74–75 °C. FT-IR (neat): 3512, 3425, 1717, 1699, 1513, 1105 cm^−1^; ^1^H NMR (400.13 MHz) (CDCl_3_) δ = 6.78–6.60 (m, 3H, Ph-H), 6.52 (s, 2H, Ph-H), 6.01 (bs, 1H), 5.63 (bs, 1H), 4.13 (t, *J* = 6.2 Hz, 2H, -CH_2_-O), 4.09 (t, *J* = 5.9 Hz, 2H, -CH_2_-O), 3.85 (s, 6H, -OCH_3_), 3.56 (s, 2H, COCH_2_-), 3.47 (s, 2H, COCH_2_-), 1.68–1.67 (m, 4H, CH_2_-CH_2_-CH_2_); ^13^C NMR (100.6 MHz) (CDCl3) δ = 172.6 (C), 172.3 (C), 147.0 (C), 143.8 (C), 143.4 (C), 133.9 (C), 126.1 (C), 124.8 (C), 121.6 (CH), 116.1 (CH), 115.1 (CH), 106.1 (CH), 64.8 (CH_2_), 64.4 (CH_2_), 56.3 (CH_3_), 41.6 (CH_2_), 40.9 (CH_2_), 25.3 (CH_2_), 25.1 (CH_2_); HRMS: *m*/*z* [M + H]+ calcd. for C_22_H_27_O_9_: 435.1655; found: 435.1641.
*Butane-1,4-diyl bis(2-(4-hydroxy-3-methoxyphenyl)acetate)* **39**. White solid, mp = 118–119 °C. FT-IR (neat): 3460, 1720, 1512, 1138, 1036 cm^−1^; ^1^H NMR (400.13 MHz) (CDCl_3_) δ = 6.88–6.76 (m, 6 H, Ph-H), 5.67 (bs, 1H, -OH), 4.12–4.09 (m, 4H, -CH_2_-O), 3.88 (s, 6H, -OCH_3_), 3.55 (s, 4H, COCH_2_-), 1.69–1.66 (m, 4H, CH_2_-CH_2_-CH_2_); ^13^C NMR (100.6 MHz) (CDCl3) δ = 171.9 (C), 146.5 (C), 144.8 (C), 125.8 (C), 122.1 (CH), 114.4 (CH), 111.8 (CH), 64.3 (CH_2_), 55.9 (CH_2_), 41. (CH_2_), 25.2 (CH_2_); HRMS: *m*/*z* [M + H]+ calcd. for C_22_H_27_O_8_: 419.1706; found: 419.1683.
*4-(2-(4-Hydroxy-3-methoxyphenyl)acetoxy)butyl 2-(3-hydroxy-4-methoxyphenyl)acetate* **40**. Yellow wax. FT-IR (neat): 3458, 1720, 1512, 1131, 1027 cm^−1^; ^1^H NMR (400.13 MHz) (CDCl_3_) δ = 6.88–6.75 (m, 6H, Ph-H), 5.78–5.72 (m, 2H, -OH), 4.11–4.10 (m, 4H, -CH_2_-O), 3.88 (s, 3H, -OCH_3_), 3.87 (s, 3H, -OCH_3_), 3.55 (s, 2H, COCH_2_-), 3.52 (s, 2H, COCH_2_-), 1.69–1.66 (m, 4H, CH_2_-CH_2_-CH_2_); ^13^C NMR (100.6 MHz) (CDCl_3_) δ = 172.0 (C), 171.8 (C), 146.5 (C), 145.8 (C), 145.6 (C), 144.8 (C), 127.2 (C), 125.8 (C), 122.1 (CH), 120.8 (CH), 115.5 (CH), 114.4 (CH), 111.8 (CH), 110.8 (CH), 64.33 (CH_2_), 64.26 (CH_2_), 56.0 (CH_3_), 55.9 (CH_3_), 41.0 (CH_2_) 40.8 (CH_2_), 25.23 (CH_2_), 25.21 (CH_2_); HRMS: *m*/*z* [M + H]+ calcd. for C_22_H_27_O_8_: 419.1706; found: 419.1684.
*4-(2-(4-Hydroxy-3,5-dimethoxyphenyl)acetoxy)butyl 2-(4-hydroxy-3-methoxyphenyl)acetate* **41**. Yellow wax. FT-IR (neat): 3417, 1721, 1613, 1516, 1115 cm^−1^; ^1^H NMR (400.13 MHz) (CDCl_3_) δ = 6.86 (d, *J* = 8.0 Hz, 1H, Ph-H), 6.81 (d, *J* = 1.7 Hz, 1H, Ph-H), 6.76 dd, (*J*_1_ = 8.0 Hz, *J*_2_ = 1.7 Hz, 1H, Ph-H), 6.52 (s, 2H, Ph-H), 5.67 (bs, 1H, -OH), 5.54 (bs, 1H, -OH), 4.11–4.10 (m, 4H, -CH_2_-O), 3.88 (s, 9H, -OCH_3_), 3.54 (s, 2H, COCH_2_-), 3.53 (s, 2H, COCH_2_-), 1.69–1.67 (m, 4H, CH_2_-CH_2_-CH_2_); ^13^C NMR (100.6 MHz) (CDCl_3_) δ = 171.9 (C), 171.7 (C), 147.0 (C), 146.5 (C), 144.8 (C), 133.9 (C), 125.7 (C), 124.5 (C), 122.1 (CH), 114.4 (CH), 111.8 (CH), 106.1 (CH), 64.3 (CH_2_), 64.2 (CH_2_), 56.3 (CH_3_), 55.9 (CH_3_), 41.4 (CH_2_), 41.0 (CH_2_), 25.23 (CH_2_), 25.20 (CH_2_); HRMS: *m*/*z* [M + H]+ calcd. for C_23_H_29_O_9_: 449.112; found: 449.1788.
*Butane-1,4-diyl bis(2-(3-hydroxy-4-methoxyphenyl)acetate)* **42**. Yellow wax. FT-IR (neat): 3391, 1736, 1589, 1510, 1126 cm^−1^; ^1^H NMR (400.13 MHz) (CDCl_3_) δ = 6.87 (1.9 Hz, 2H, Ph-H), 6.82–6.75 (m, 4H, Ph-H), 5.80 (bs, 2H, -OH), 4.11–4.08 (m, 4H, -CH_2_-O), 3.87 (s, 6H, -OCH_3_), 3.53 (s, 4H, COCH_2_-), 1.68–1.66 (m, 4H, CH_2_-CH_2_-CH_2_); ^13^C NMR (100.6 MHz) (CDCl_3_) δ = 171.8 (C), 145.8 (C), 145.6 (C), 127.2 (C), 120.8 (CH), 115.6 (CH), 110.8 (CH), 64.3 (CH_2_), 56.0 (CH_3_), 40.8 (CH_2_), 25.2 (CH_2_); HRMS: *m*/*z* [M + H]+ calcd. for C_22_H_27_O_8_: 419.1706; found: 419.1683.
*4-(2-(4-Hydroxy-3,5-dimethoxyphenyl)acetoxy)butyl 2-(3-hydroxy-4-methoxyphenyl)acetate* **43**. Pale yellow solid, mp = 42–43 °C. FT-IR (neat): 3447, 1715, 1514, 1213, 1115 cm^−1^; ^1^H NMR (400.13 MHz) (CDCl_3_) δ = 6.86 (d, *J* = 2.0 Hz, 1H, Ph-H), 6.81–6.79 (m, 1H, Ph-H), 6.75 (dd, *J*_1_ = 8.2 Hz, *J*_2_ = 2.0 Hz, 1H, Ph-H), 6.52 (s, 2H, Ph-H), 5.75 (bs, 1H, -OH), 5.55 (bs, 1H, -OH), 4.11–4.10 (m, 4H, -CH_2_-O), 3.88 (s, 6H, -OCH_3_), 3.87 (s, 3H, -OCH_3_), 3.54 (s, 2H, COCH_2_-), 3.51 (s, 2H, COCH_2_-), 1.68 (m, 4 H, CH_2_-CH_2_-CH_2_); ^13^C NMR (100.6 MHz) (CDCl_3_) δ = 171.8 (C), 171.5 (C), 147.0 (C), 145.8 (C), 145.6 (C), 133.9 (C), 127.2 (C), 124.9 (C), 120.8 (CH), 115.5 (CH), 110.8 (CH), 106.0 (CH), 64.4 (CH_2_), 64.2 (CH_2_), 56.3 (CH_3_), 56.0 (CH_3_), 41.4 (CH_2_), 40.7 (CH_2_), 25.24 (CH_2_), 25.20 (CH_2_); HRMS: *m*/*z* [M + H]+ calcd. for C_23_H_29_O_9_: 449.1812; found: 449.1788.
*Butane-1,4-diyl bis(2-(4-hydroxy-3,5-dimethoxyphenyl)acetate)* **44**. White solid, mp = 129–130 °C. FT-IR (neat): 3423, 1733, 1615, 1521, 1468 cm^−1^; ^1^H NMR (400.13 MHz) (CDCl_3_) δ = 6.51 (s, 4H, Ph-H), 5.54 (bs, 2H, -OH), 4.10 (bs, 4H, -CH_2_-O), 3.86 (s, 12H, -OCH_3_), 3.52 (s, 4H, COCH_2_-), 1.68 (bs, 4H, CH_2_-CH_2_-CH_2_); ^13^C NMR (100.6 MHz) (CDCl_3_) δ = 171.7 (C), 147.0 (C), 133.9 (C), 124.9 (C), 106.0 (CH), 64.3 (CH_2_), 56.3 (CH_3_), 41.4 (CH_2_), 25.2 (CH_2_); HRMS: *m*/*z* [M + H]+ calcd. for C_24_H_31_O_10_: 479.1917; found: 479.1896.

### 3.4. Evaluation of the LogP

The lipophilicity of all esters and diesters was estimated with a logP = log10, where P = partition coefficient (ratio between the concentration of a sample in 1-octanol and water), calculated using the software Chemdraw Professional 15.1. LogP < 0 means that the sample has a higher affinity for water (it is hydrophilic); logP > 0 indicates that the sample has a higher affinity for 1-octanol (it is lipophilic).

### 3.5. Evaluation of the In Vitro Antioxidant Activity

#### 3.5.1. DPPH Assay

The procedure used was reported in the literature [52] and here briefly described. 2,2-Diphenyl-1-picrylidrazyl stock methanol solution (DPPH, 75 μM) was prepared. Compounds (1.5 mM) were dissolved in methanol and tested at different concentrations (5, 10, 25, 35, 42, 50, and 75 μM). A total of 0.05 mL of each dilution was mixed with 0.95 mL of DPPH reagent solution. The absorbance was measured using a spectrophotometer (λ = 517 nm) after 30 min of incubation in the dark at room temperature. A total of 1 mL solution (0.05 mL of methanol and 0.95 mL DPPH solution) was used as a blank, and the percentage of RSA (radical scavenging activity) was calculated using the following formula:

$$RSA(\%)=\frac{Ab-As}{Ab}\times 100\;$$

where Ab refers to the control reaction (containing all reagents, except the tested compound), and As is the absorbance of the test reaction (containing all reagents with the tested compound). The increase in RSA corresponded to the decrease in the absorbance value. The ability of each new synthetic compound to reduce DPPH is reported as an IC_50_ value, which represents the concentration of the sample required to scavenge 50% of the free radicals in the reaction mixture. The smaller the IC_50_ value, the larger the RSA value, and the higher the antioxidant activity.

#### 3.5.2. ABTS Assay

The procedure used is reported in the literature [53] and here briefly described. A total of 30.7 mg of 2,2′-azino-bis(3-ethylbenzothiazoline-6-sulfonic acid) (ABTS) and 5.3 mg of potassium persulfate (K_2_S_2_O_8_) were solubilized in 8 mL of distilled H_2_O. The solution was stored in the dark at room temperature for 16 h to form the blue ABTS radical cation (ABTS^•+^) and then diluted to reach an absorbance of 0.7 at λ = 734 nm. A total of 0.95 mL of the ABTS^•+^ solution was combined with an ethanolic compound solution (0.05 mL) diluted at different concentrations (2, 4, 8, 16, 32, and 50 μM). The solutions were incubated in the dark for 10 min at room temperature, and the ABTS^•+^ was quantified spectrophotometrically. Each data are expressed as the Trolox equivalent antioxidant capacity (TEAC), where Trolox, an analog of vitamin E, is a water-soluble standard reference compound. The compound’s ability to scavenge free radicals is compared to that of Trolox, indicating the amount of Trolox required to produce the same antioxidant effect. The radical scavenging percentage was compared to a blank solution containing 0.05 mL of ethanol. The extent of decolorization was calculated as the percentage reduction in the absorbance. The scavenging capability of each tested compound was calculated using the following equation:

$$\mathrm{Radical}\;\mathrm{cation}\;\mathrm{ABTS}\;(\%)=\frac{Ab-As}{Ab}\times 100$$

where Ab refers to the absorbance of the control reaction (containing all reagents, except the tested compound), and As is the absorbance of the remaining ABTS^•+^ in the presence of the scavenger. The TEAC values were calculated by comparing the compound slope line with the Trolox slope.

### 3.6. Statistical Analysis

All determinations represent the means of three independent experiments; each conducted in triplicate (n = 3). The IC_50_, TEAC values, and standard deviations (μM) were calculated using linear regression. The data are expressed as the mean ± standard deviation (SD) and assessed with one-way analysis of variance (ANOVA) using RStudio Software 2024.09.1 +394, Posit team (2025), RStudio: Integrated Development Environment for R, Posit Software 2024.09.1 +394, PBC, Boston, MA, URL: http://www.posit.co/ (accessed on 3 March 2025) [54]. Significant differences among means were determined using the Tukey post hoc test and Dunnett post hoc test (*p* < 0.05), analyzed with RStudio packages [55,56,57].

### 3.7. Qualitative Evaluation of the Bactericidal Properties Against Staphylococcus aureus and Escherichia coli

#### 3.7.1. Bacterial Stains

*Staphylococcus aureus* ATCC 25923 and *Escherichia coli* ATCC 25922 were available from Bioricerche Srl. The Baird Parker agar base for *Staphylococcus aureus*, maximum recovery diluent (sterile saline), and tryptone bile X-Gluc (TBX) agar for *Escherichia coli* were purchased from Biolife Italiana Srl (Milan, Italy); the sterile plasticware was furnished from Unifo srl (Zero Branco, Treviso, Italy).

#### 3.7.2. Growth Inhibition Assay

A dilution method described in the literature was used [58]. Briefly, a stock solution from each compound was prepared at a concentration of 10 mmol/L by dissolving it in sterile saline with 10% dimethyl sulfoxide (DMSO). From this stock, solutions of 5 mmol/L and 1 mmol/L were prepared through serial dilution using the same saline–DMSO mixture. Gentamicin sulfate (>590 mg/mg, Sigma Aldrich G1264) was used as a positive control. A stock solution of 500 mmol/L was prepared in a similar manner to the compounds’ solutions. From this stock, additional dilutions were prepared, obtaining final test solutions of 250 and 100 mmol/L.

Bacterial suspensions were obtained by reviving the strains from cryovials using Brain Heart Infusion broth and culturing them until optimal growth was achieved (37 °C, 24 h). Once ready, the bacterial suspension was adjusted to 0.5 McFarland, which corresponds to approximately 10^8^ CFU/mL. For each test, 1 mL of the standardized bacterial suspension was mixed with 9 mL of the solution to be tested. Subsequently, the tubes were incubated for 24 h at 37 °C. After the incubation period, samples from each tube were streaked onto a Baird Parker Agar Base for *Staphylococcus aureus* and TBX for *Escherichia coli* according to the manufacturer’s instructions and incubated for 48 h and 24 h, respectively. The minimum bactericidal concentration (MBC, mmol/L) was determined by observing the plates for any bacterial growth. All tests were carried out in triplicate.

## 4. Conclusions

In conclusion, novel hydroxyalkyl esters **10**–**29** were synthetized by Fischer esterification in good to excellent yields (60–96%) from hydroxyphenylacetic acids **1**–**5** and α,ω-diols **6**–**9** of increasing chain lengths from 2 to 8 carbon atoms. As examples of diesters, butyl diarylacetates **30**–**34** and **36**–**44** were obtained from hydroxybutyl esters **11**, **15**, **19**, **23**, and **27** and hydroxyphenylacetic acids **1**–**5** under Mitsunobu conditions in moderate to good yields (40–78%). The DPPH and ABTS assays of the isolated diesters **30**–**34** and **36**–**44** were performed to evaluate their in vitro antioxidant activity, and a structure–activity relationship related to the substitution pattern on the aromatic rings was reported.

Diesters **31**, **36**, **37**, and **38**, having a catecholic moiety on one of two aromatic rings and increased lipophilicity, were the most effective, and their antibacterial activity against *Staphylococcus aureus* and *Escherichia coli* was preliminarily evaluated. These compounds seemed to be effective; nevertheless, further experimental investigations are necessary to validate these results. Due to these preliminary biological activity results, hydroxybutyl esters and butyl diarylacetates could have a broad spectrum of potential food and biomedical applications.

## Figures and Tables

**Table 1:** Synthesis of hydroxyalkyl esters **10**–**29**.

Entry	Acid	Diol	Time (h)	Ester	Yield (%) ^a^	LogP ^b^
1	**1**	**6**	5	**10**	78	0.89
2	**1**	**7**	1	**11**	85	1.45
3	**1**	**8**	2	**12**	88	2.29
4	**1**	**9**	1.5	**13**	72	3.12
5	**2**	**6**	4	**14**	79	0.50
6	**2**	**7**	1	**15**	69	1.06
7	**2**	**8**	2	**16**	76	1.90
8	**2**	**9**	1	**17**	46	2.73
9	**3**	**6**	2	**18**	82	0.77
10	**3**	**7**	1	**19**	96	1.32
11	**3**	**8**	1	**20**	65	2.16
12	**3**	**9**	1	**21**	60	2.99
13	**4**	**6**	5	**22**	67	0.77
14	**4**	**7**	3	**23**	80	1.32
15	**4**	**8**	1	**24**	81	2.16
16	**4**	**9**	1	**25**	65	2.99
17	**5**	**6**	3	**26**	94	0.64
18	**5**	**7**	1.5	**27**	86	1.20
19	**5**	**8**	4	**28**	79	2.03
20	**5**	**9**	0.5	**29**	65	2.87

^a^ Refers to the isolated ester. For ester **17**, the yield was calculated by ^1^H NMR of the fraction containing the starting diol. ^b^ Calculated using the software Chemdraw Professional 15.1.

**Table 2:** IC_50_ and TEAC values of hydroxyphenylacetic acids **1**–**5** and hydroxyalkyl esters **10**–**29**.

Entry	Compound	IC_50_ (μM) ^a^	TEAC (μM) ^a^
1	**1**	>200	<0.05
2	**10**	>200	<0.05
3	**11**	>200	<0.05
4	**12**	>200	<0.05
5	**13**	>200	<0.05
6	**2**	12.5 ± 0.2	0.92 ± 0.05
7	**14**	13.0 ± 0.4	0.88 ± 0.07
8	**15**	13.1 ± 0.5	0.85 ± 0.10
9	**16**	17.8 ± 0.4	0.63 ± 0.17
10	**17**	16.0 ± 0.1	0.52 ± 0.01
11	**3**	56.8 ± 1.6	0.14 ± 0.01
12	**18**	39.5 ± 0.4	0.16 ± 0.01
13	**19**	40.3 ± 1.5	0.17 ± 0.01
14	**20**	51.4 ± 1.1	0.15 ± 0.02
15	**21**	51.2 ± 0.7	0.15 ± 0.02
16	**4**	59.7 ± 3.3	0.10 ± 0.02
17	**22**	41.2 ± 0.2	0.09 ± 0.01
18	**23**	48.9 ± 2.6	0.13 ± 0.01
19	**24**	49.7 ± 1.4	0.09 ± 0.01
20	**25**	50.6 ± 0.4	0.09 ± 0.01
21	**5**	25.8 ± 1.2	0.42 ± 0.04
22	**26**	24.2 ± 1.0	0.25 ± 0.09
23	**27**	24.3 ± 0.9	0.28 ± 0.01
24	**28**	26.1 ± 0.4	0.24 ± 0.01
25	**29**	28.2 ± 1.5	0.36 ± 0.06

^a^ IC_50_ and TEAC values (μM) are expressed as the mean ± standard deviation (n = 3).

**Table 3:** Reactions yields and logP of butyl diarylacetates **30**–**44**.

Entry	Ester	Acid	Time (h)	Diester	Yield (%) ^a^	LogP ^b^
1	**11**	**1**	4	**30**	56	3.13
2	**11**	**2**	2.5	**31**	59	2.74
3	**11**	**3**	2	**32**	60	3.01
4	**11**	**4**	5	**33**	53	3.01
5	**11**	**5**	1	**34**	55	2.88
6	**15**	**2**	5	**35**	Not isolated	Not calculated
7	**15**	**3**	2	**36**	58	2.62
8	**15**	**4**	4.5	**37**	45	2.62
9	**15**	**5**	2	**38**	78	2.49
10	**19**	**3**	3	**39**	48	2.88
11	**19**	**4**	1.5	**40**	60	2.88
12	**19**	**5**	3	**41**	48	2.75
13	**23**	**4**	1	**42**	65	2.88
14	**23**	**5**	6	**43**	40	2.75
15	**27**	**5**	1.5	**44**	71	2.63

^a^ Refers to the isolated diester. ^b^ Calculated using the software ChemDraw Professional 15.1.

**Table 4:** IC_50_ and TEAC values of butyl diarylacetates **30**–**34** and **36**–**44**.

Entry	Diester	IC_50_ (μM) ^a^	TEAC (μM) ^a^
1	**30**	>200	<0.05
2	**31**	19.5 ± 0.7	0.94 ± 0.01
3	**32**	60.8 ± 1.0	0.08 ± 0.02
4	**33**	43.1 ± 4.5	0.09 ± 0.03
5	**34**	26.9 ± 0.3	0.38 ± 0.01
6	**36**	12.6 ± 0.1	0.75 ± 0.22
7	**37**	12.2 ± 0.1	0.71 ± 0.03
8	**38**	17.5 ± 0.8	0.49 ± 0.04
9	**39**	38.2 ± 0.4	0.13 ± 0.06
10	**40**	35.3 ± 0.1	0.13 ± 0.08
11	**41**	27.3 ± 0.8	0.56 ± 0.02
12	**42**	31.3 ± 2.8	0.11 ± 0.01
13	**43**	26.4 ± 1.5	0.68 ± 0.01
14	**44**	14.3 ± 1.8	0.20 ± 0.01

^a^ IC_50_ and TEAC values (μM) are expressed as the mean ± standard deviation (n = 3).

**Table 5:** Minimum bactericidal concentration (MBC) of hydroxyphenylacetic acids **1**–**5** and butyl diarylacetates **31**, **36**, **37**, and **38** against Gram-positive *S. aureus* ATCC 25923 and Gram-negative *E. coli* ATCC 25922.

Entry	Compound	MBC (mmol/L) *S. aureus* ATCC 25923	MBC (mmol/L) *E. coli* ATCC 25922
1	**1**	-- ^a^	-- ^a^
2	**2**	10	10
3	**3**	10	-- ^a^
4	**4**	10	10
5	**5**	5.0	-- ^a^
6	**31**	5.0	10
7	**36**	5.0	10
8	**37**	10	10
9	**38**	5.0	10
10	Gentamicin sulfate ^b^	0.25	<0.10

^a^ Presence of bacterial growth even at 10 mmol/L. ^b^ Positive control.

## Data Availability

^1^H NMR, ^13^C NMR, HRMS and UV spectra of all synthetized compounds are described within the article and reported in Supplementary Materials.

## Supplementary Materials

The following supporting information can be downloaded at: https://www.mdpi.com/article/10.3390/molecules30153087/s1. ^1^H and ^13^C NMR, FT-IR and HRMS Spectra are included.
