# Supplementary material for: Synthesis of Novel Bioactive Lipophilic Hydroxyalkyl Esters and Diesters Based on Hydroxyphenylacetic Acids
Source: Molecules. 2025 Jul 23;30(15):3087. doi: 10.3390/molecules30153087 (PMC12348810; doi:10.3390/molecules30153087)
Supplement: Supplementary file 1 [file molecules-30-03087-s001.zip › molecules-3658301-supplementary.pdf]

Electronic Supporting Information (ESI)

Synthesis of novel bioactive lipophilic hydroxyalkyl esters and diesters based on hydroxyphenylacetic acids

Andrea Fochetti [1*], Noemi Villanova [1], Andrea Lombardi [1,2], Veronica Lelli [3], Yuri Gazzilli [4], Anna Maria Timperio [3], Giancarlo Fabrizi [4] and Roberta Bernini[1*]

[1] Department of Agriculture and Forest Sciences (DAFNE), University of Tuscia, Via San Camillo de Lellis, 01100 Viterbo, Italy; e-mail: andrea.fochetti@unitus.it; noemi.villanova@unitus.it; andrea.lombardi@unitus.it; roberta.bernini@unitus.it

[2] Bioricerche S.r.l., Loc. Ferro di Cavallo, 58034 Castell'Azzara, Italy

[3] Department of Ecological and Biological Sciences (DEB), University of Tuscia, 01100 Viterbo (Italy); v.lelli@unitus.it; timperio@unitus.it

[4] Department of Chemistry and Technology of Drugs, Sapienza, University of Rome, P.le Aldo Moro 5, 00185 Rome, Italy; yuri.gazzilli@uniroma1.it; giancarlo.fabrizi@uniroma1.it

* Correspondence:
Andrea Fochetti (A.F.), e-mail: andrea.fochetti@unitus.it; Roberta Bernini (R.B.), e-mail: roberta.bernini@unitus.it

^{1}H NMR, **^{13}C NMR**, **HRMS** and **FTIR spectra** of hydroxyalkyl esters **10-29** and butyl diarylacetates **30-34, 36-44**

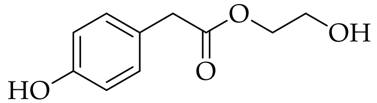

2-Hydroxyethyl 2-(4-hydroxyphenyl)acetate **10**

^{1}H NMR

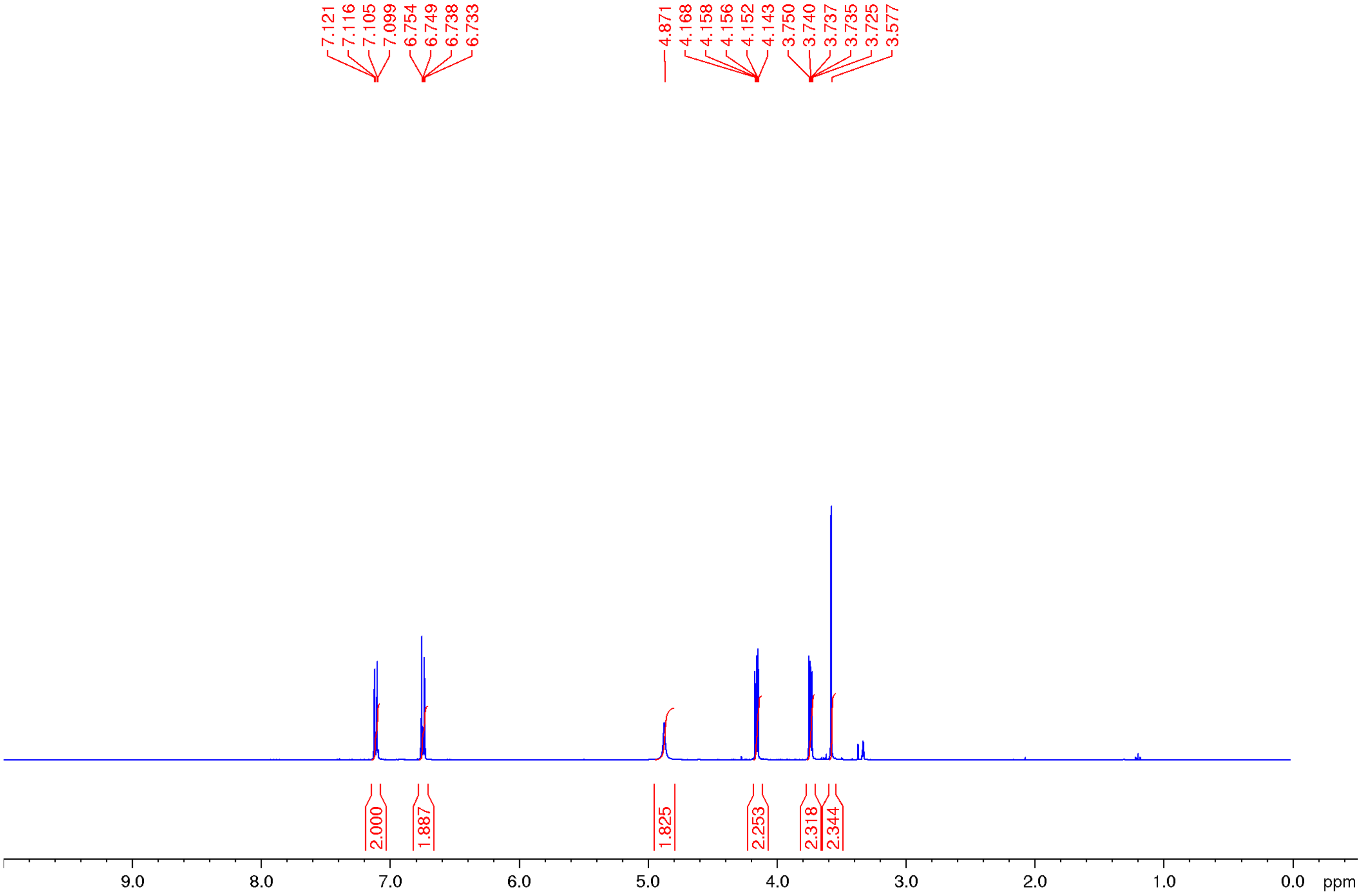

^{13}C NMR

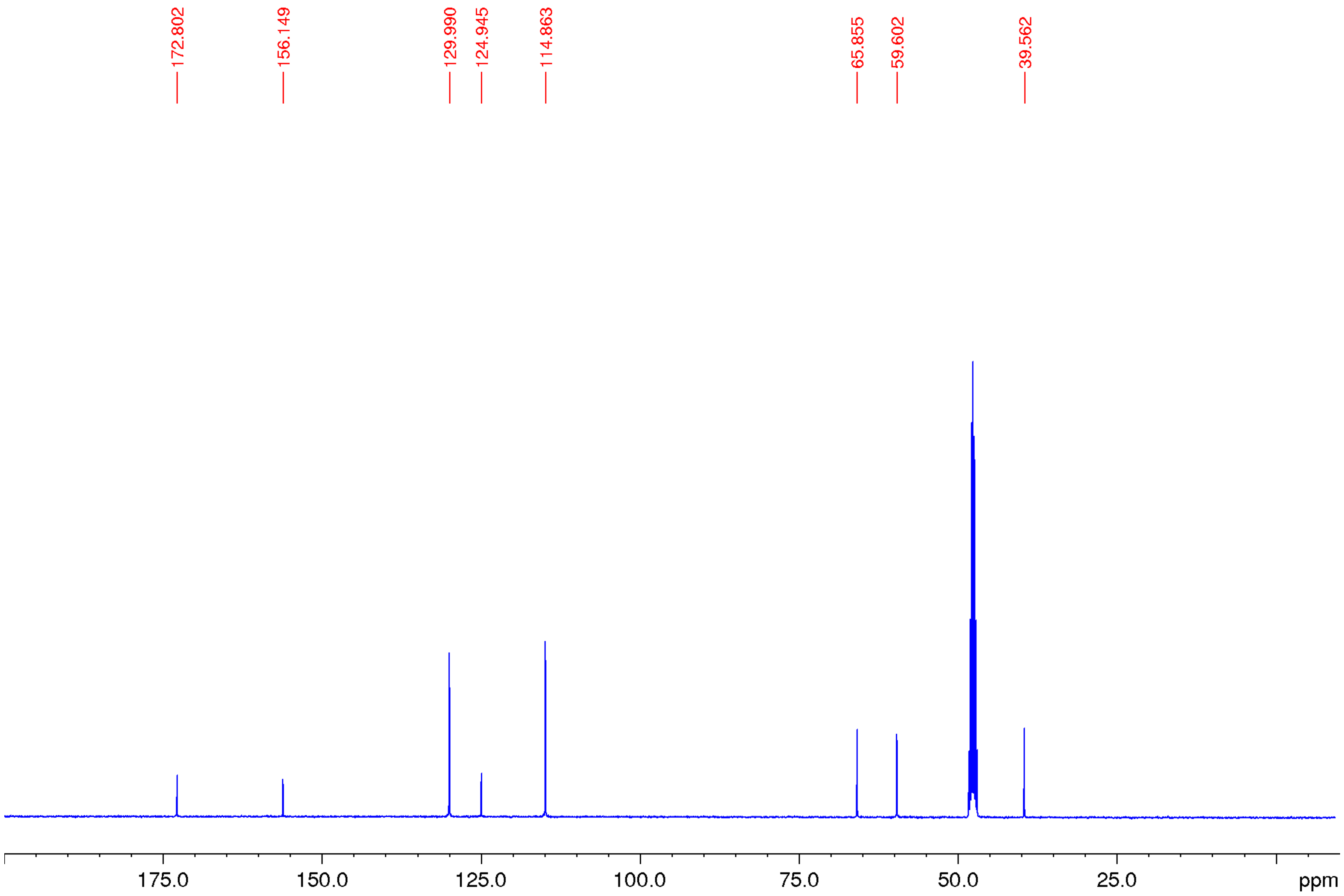
172.802
156.149
129.990
124.945
114.863
65.855
59.602
39.562
175.0
150.0
125.0
100.0
75.0
50.0
25.0
ppm

HRMS

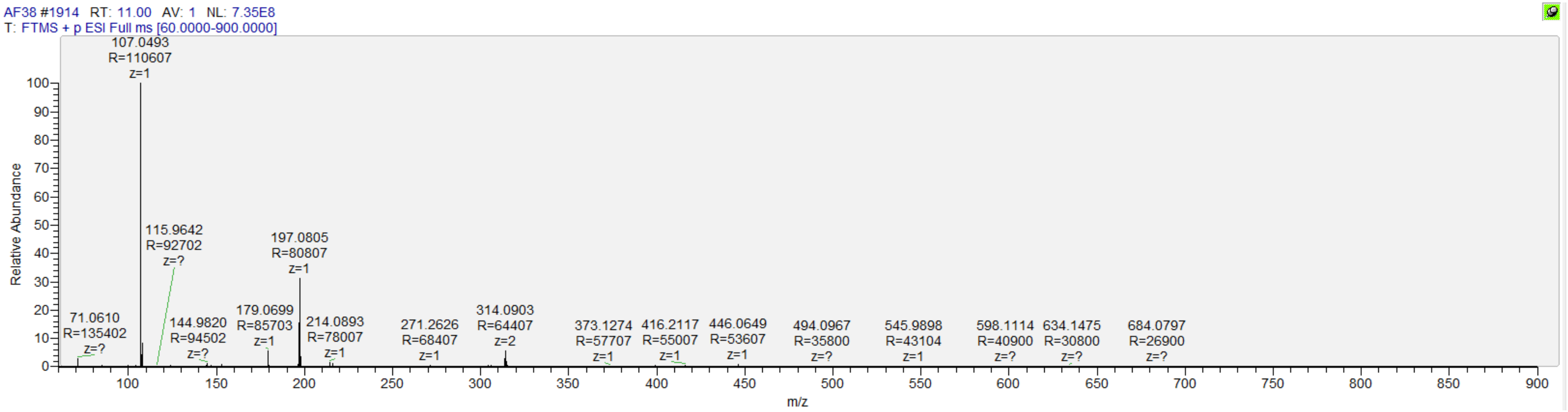
AF38 #1914 RT: 11.00 AV: 1 NL: 7.35E8
T: FTMS + p ESI Full ms [60.0000-900.0000]
107.0493 R=110607 z=1
115.9642 R=92702 z=?
197.0805 R=80807 z=1
71.0610 R=135402 z=?
144.9820 R=94502 z=?
179.0699 R=85703 z=1
214.0893 R=78007 z=1
271.2626 R=68407 z=1
314.0903 R=64407 z=2
373.1274 R=57707 z=1
416.2117 R=55007 z=1
446.0649 R=53607 z=1
494.0967 R=35800 z=?
545.9898 R=43104 z=1
598.1114 R=40900 z=?
634.1475 R=30800 z=?
684.0797 R=26900 z=?
Relative Abundance
m/z

FTIR

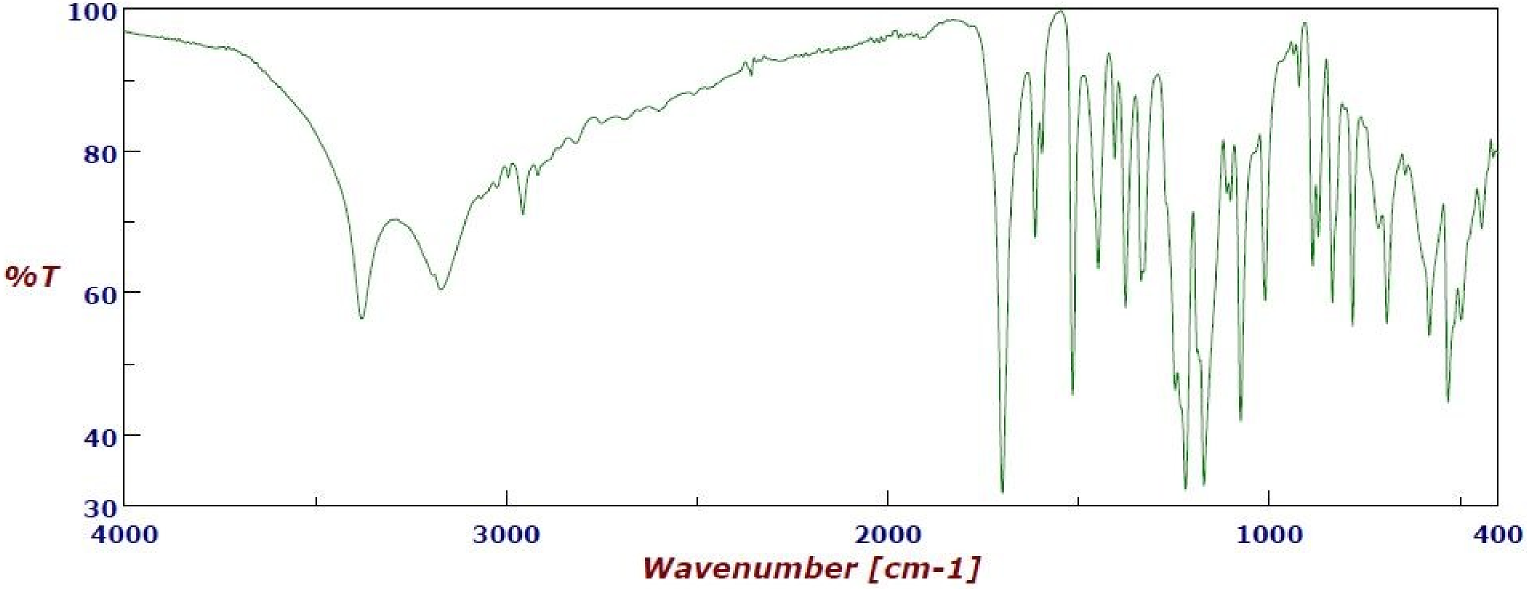
%T
Wavenumber [cm-1]

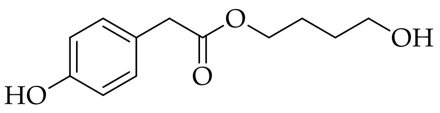

4-Hydroxybutyl 2-(4-hydroxyphenyl)acetate **11**

^{1}H NMR

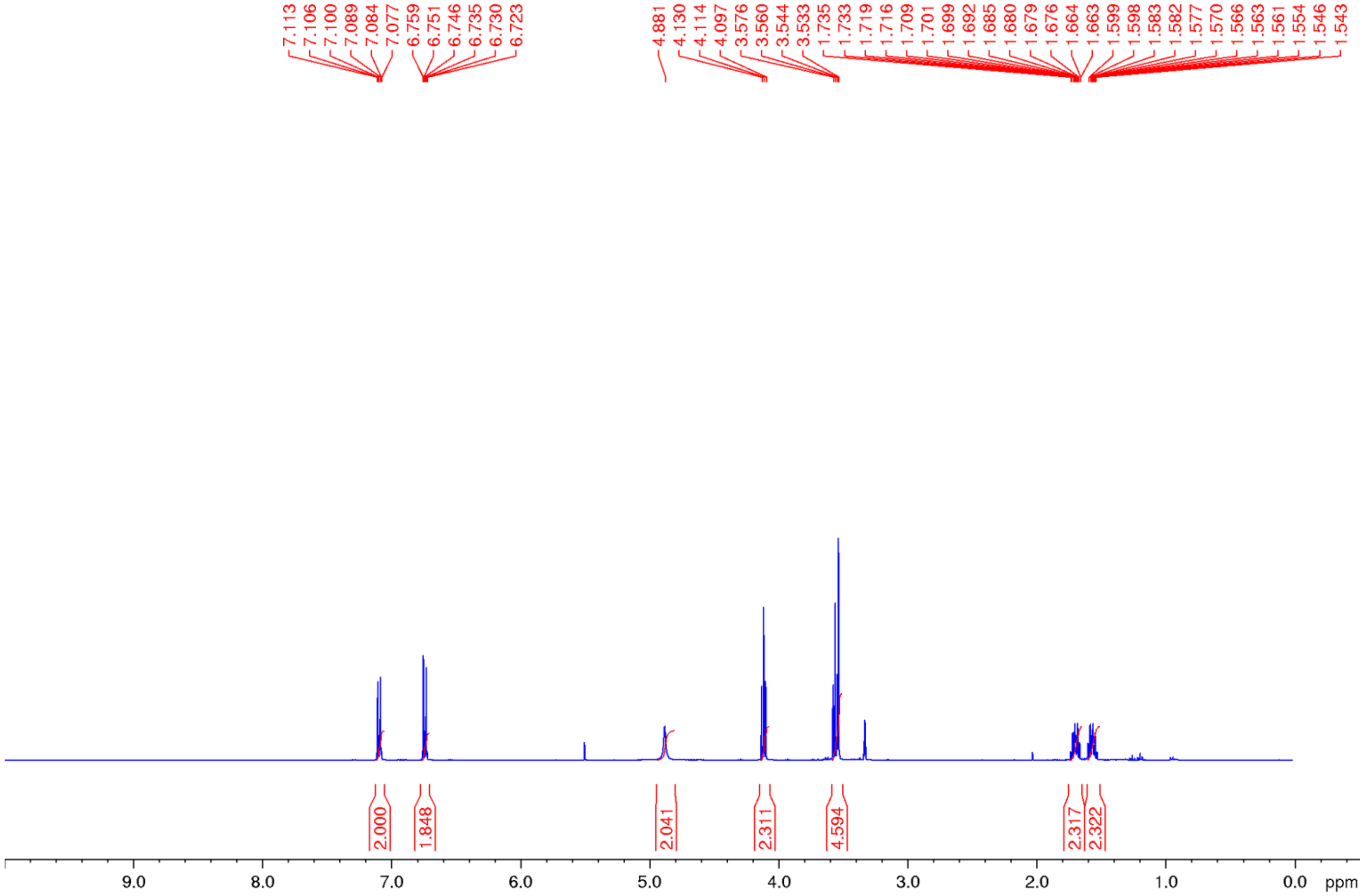

^{13}C NMR

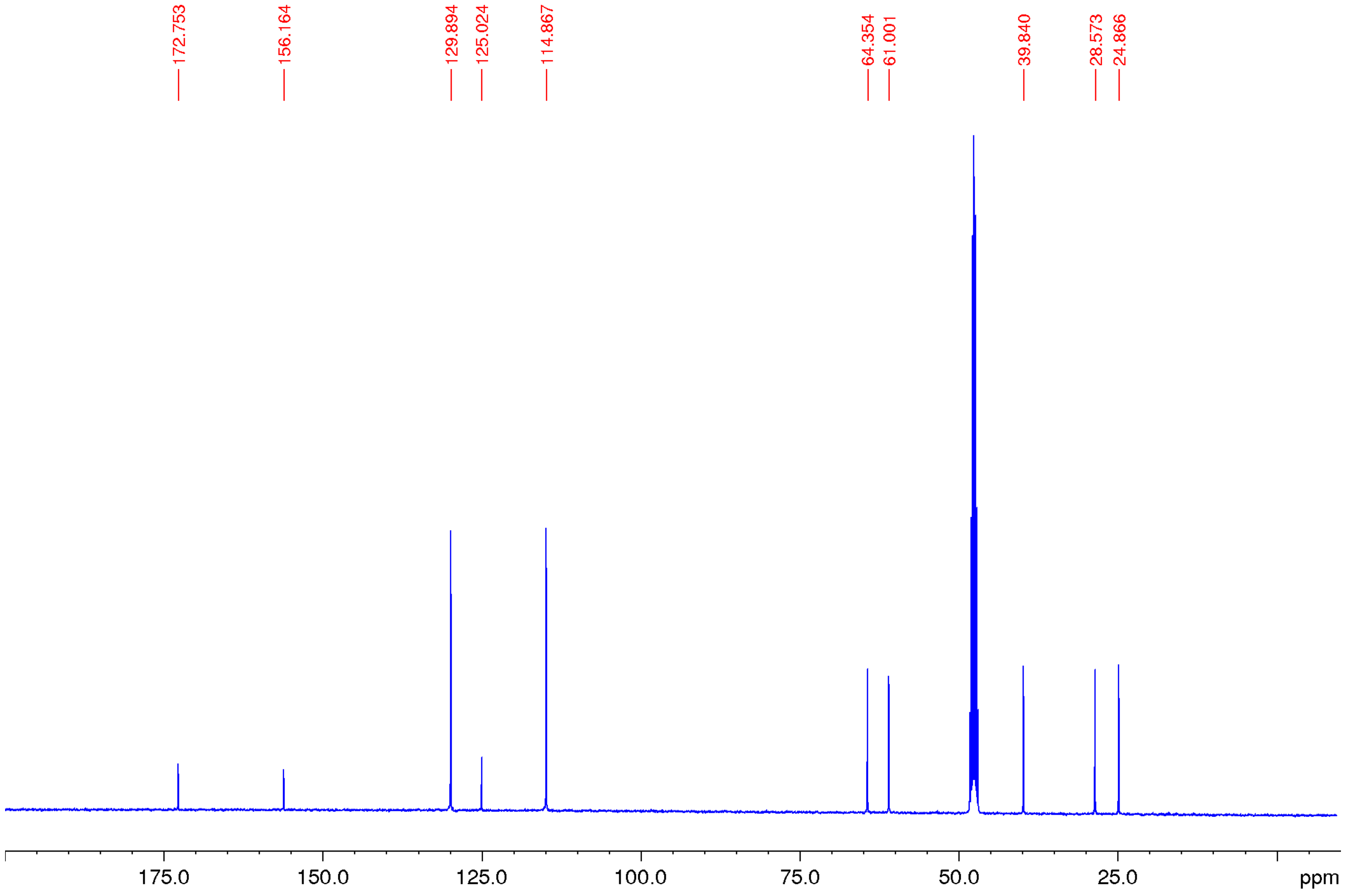

172.753
156.164
129.894
125.024
114.867
64.354
61.001
39.840
28.573
24.866
175.0
150.0
125.0
100.0
75.0
50.0
25.0
ppm

HRMS

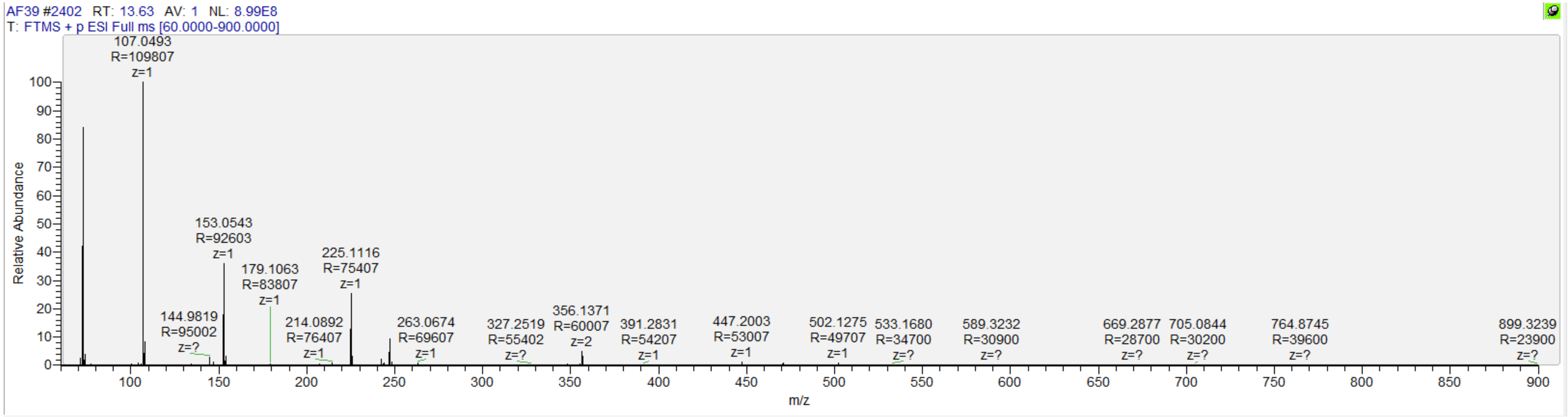
AF39 #2402 RT: 13.63 AV: 1 NL: 8.99E8
T: FTMS + p ESI Full ms [60.0000-900.0000]
107.0493
R=109807
z=1
153.0543
R=92603
z=1
144.9819
R=95002
z=?
179.1063
R=83807
z=1
214.0892
R=76407
z=1
225.1116
R=75407
z=1
263.0674
R=69607
z=1
327.2519
R=55402
z=?
356.1371
R=60007
z=2
391.2831
R=54207
z=1
447.2003
R=53007
z=1
502.1275
R=49707
z=1
533.1680
R=34700
z=?
589.3232
R=30900
z=?
669.2877
R=28700
z=?
705.0844
R=30200
z=?
764.8745
R=39600
z=?
899.3239
R=23900
z=?
Relative Abundance
m/z

FTIR

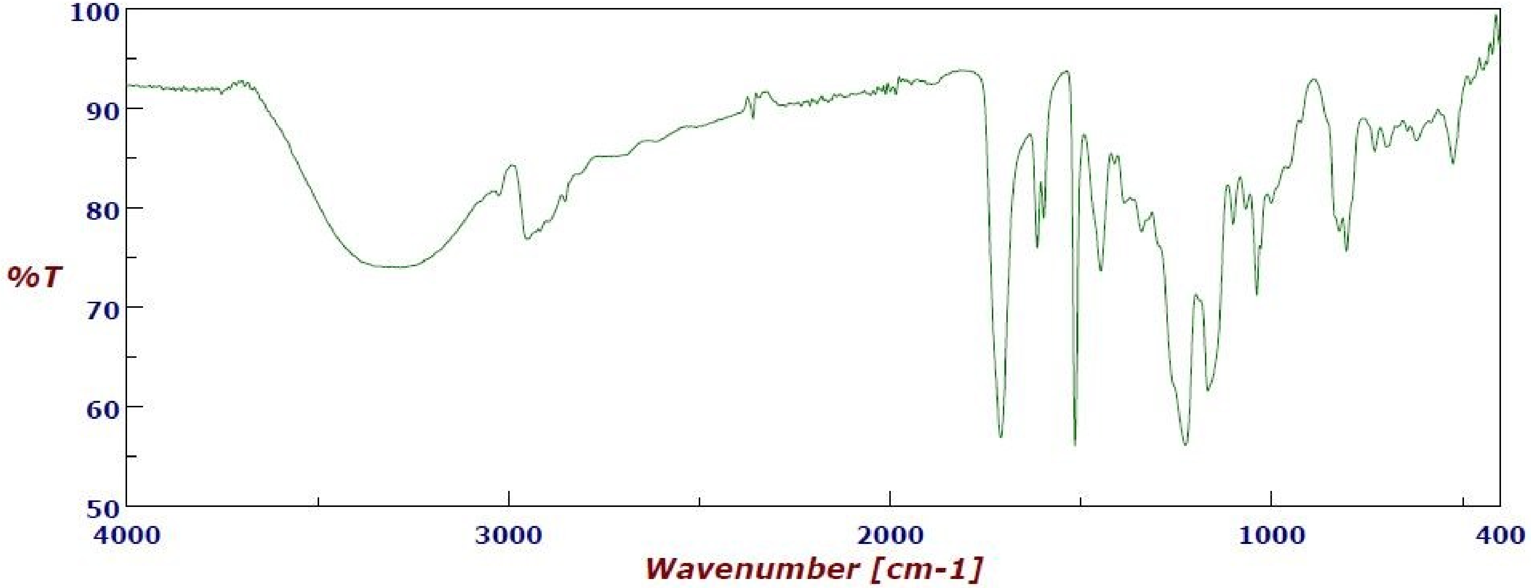
%T
Wavenumber [cm-1]

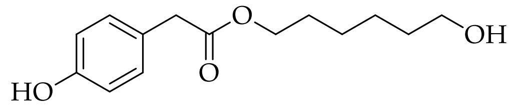

6-Hydroxyhexyl 2-(4-hydroxyphenyl)acetate **12**

^{1}H NMR

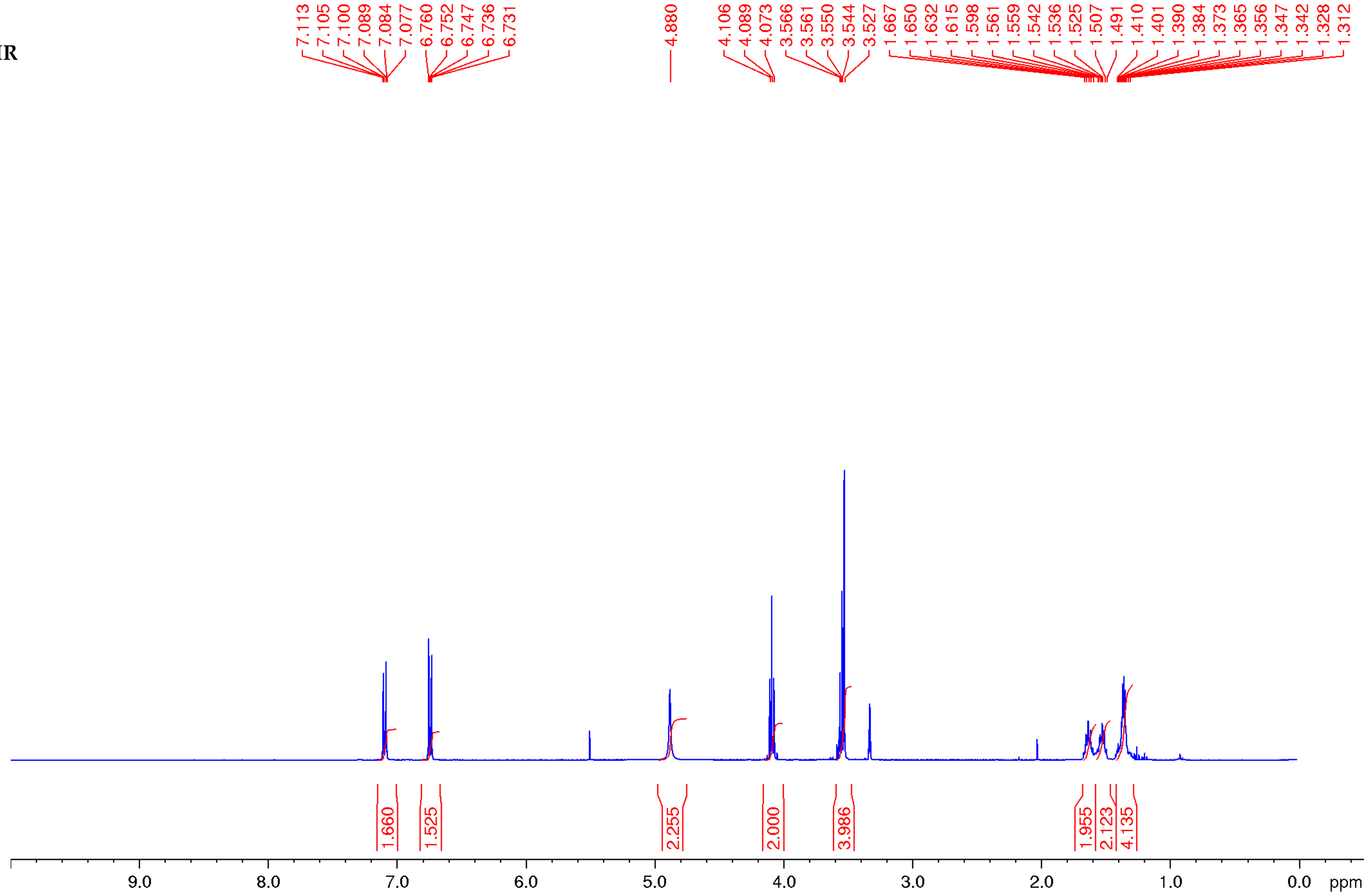

¹³C NMR

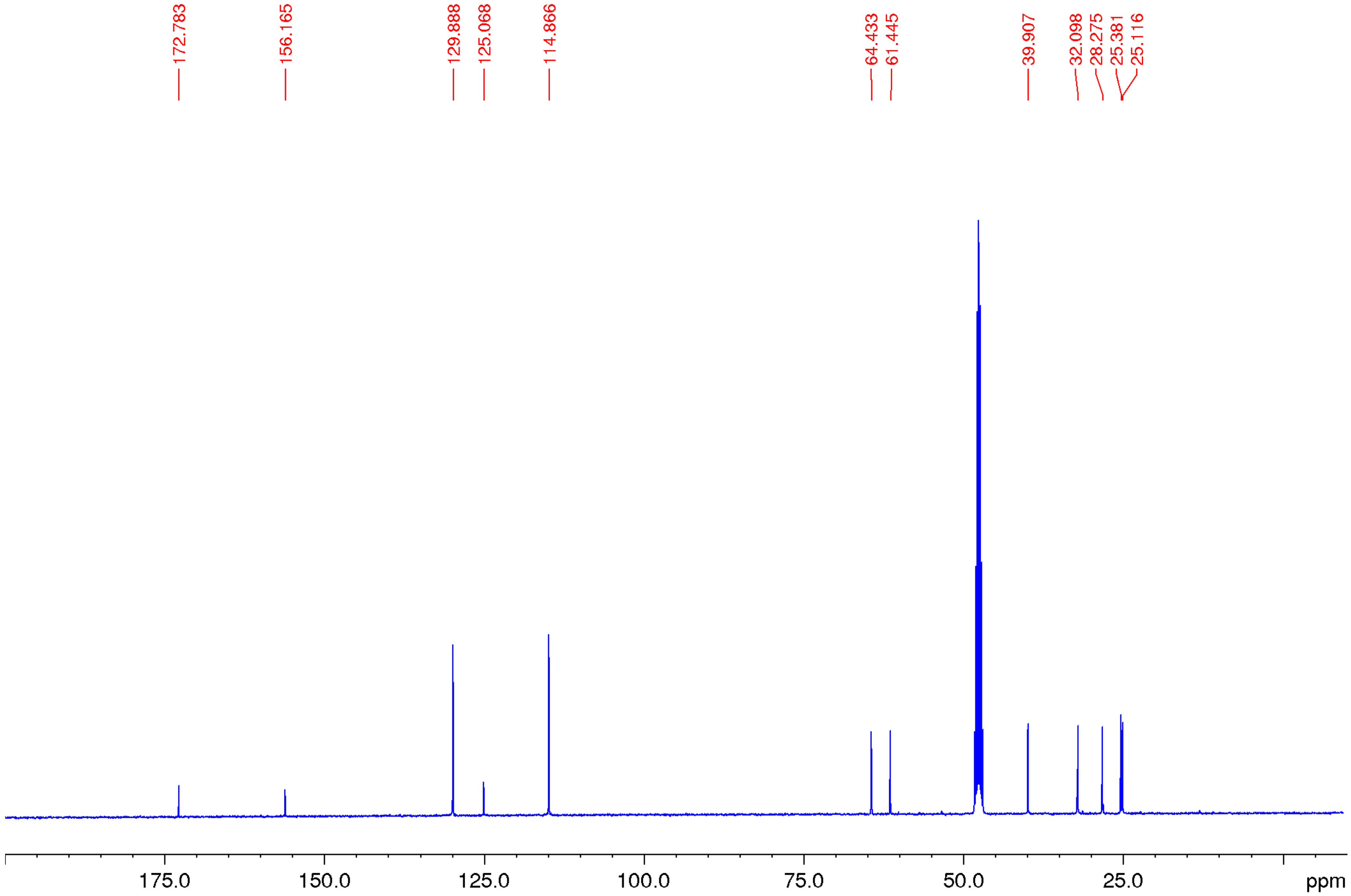
172.783
156.165
129.888
125.068
114.866
64.433
61.445
39.907
32.098
28.275
25.381
25.116
175.0
150.0
125.0
100.0
75.0
50.0
25.0
ppm

HRMS

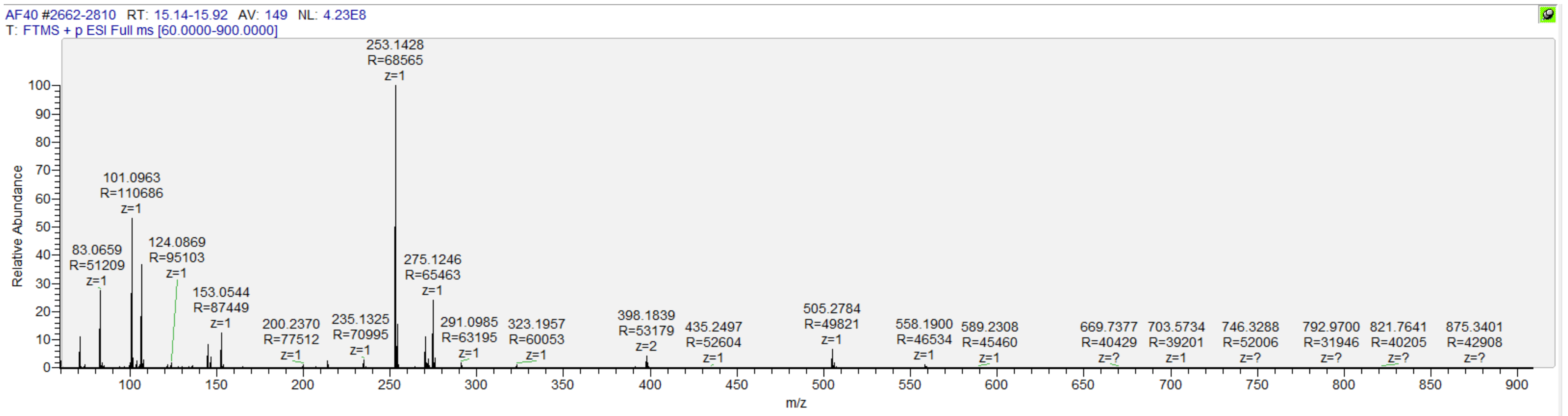
AF40 #2662-2810 RT: 15.14-15.92 AV: 149 NL: 4.23E8
T: FTMS + p ESI Full ms [60.0000-900.0000]
253.1428 R=68565 z=1
101.0963 R=110686 z=1
83.0659 R=51209 z=1
124.0869 R=95103 z=1
153.0544 R=87449 z=1
200.2370 R=77512 z=1
235.1325 R=70995 z=1
275.1246 R=65463 z=1
291.0985 R=63195 z=1
323.1957 R=60053 z=1
398.1839 R=53179 z=2
435.2497 R=52604 z=1
505.2784 R=49821 z=1
558.1900 R=46534 z=1
589.2308 R=45460 z=1
669.7377 R=40429 z=?
703.5734 R=39201 z=1
746.3288 R=52006 z=?
792.9700 R=31946 z=?
821.7641 R=40205 z=?
875.3401 R=42908 z=?
Relative Abundance
m/z

FTIR

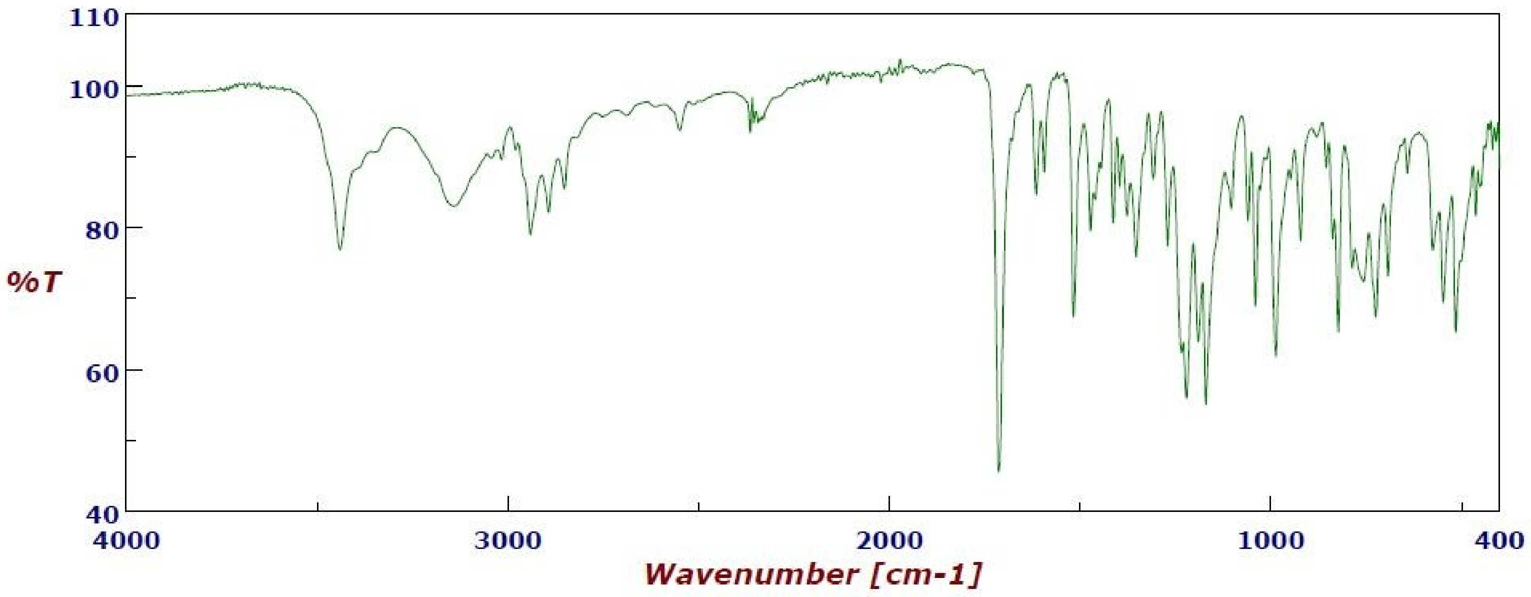
%T
Wavenumber [cm-1]

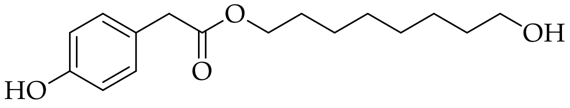

8-Hydroxyoctyl 2-(4-hydroxyphenyl)acetate **13**

^{1}H NMR

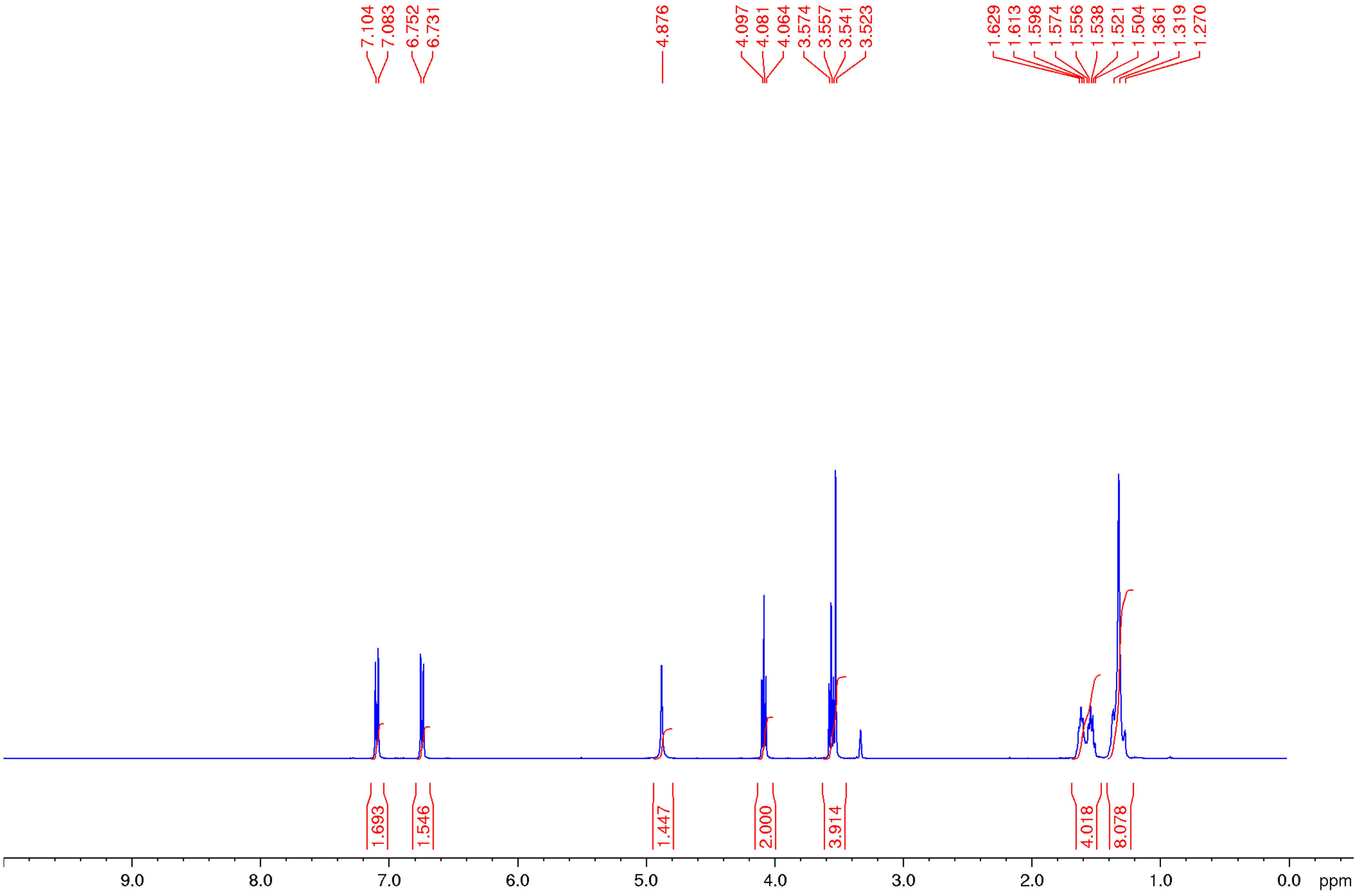

^{13}C NMR

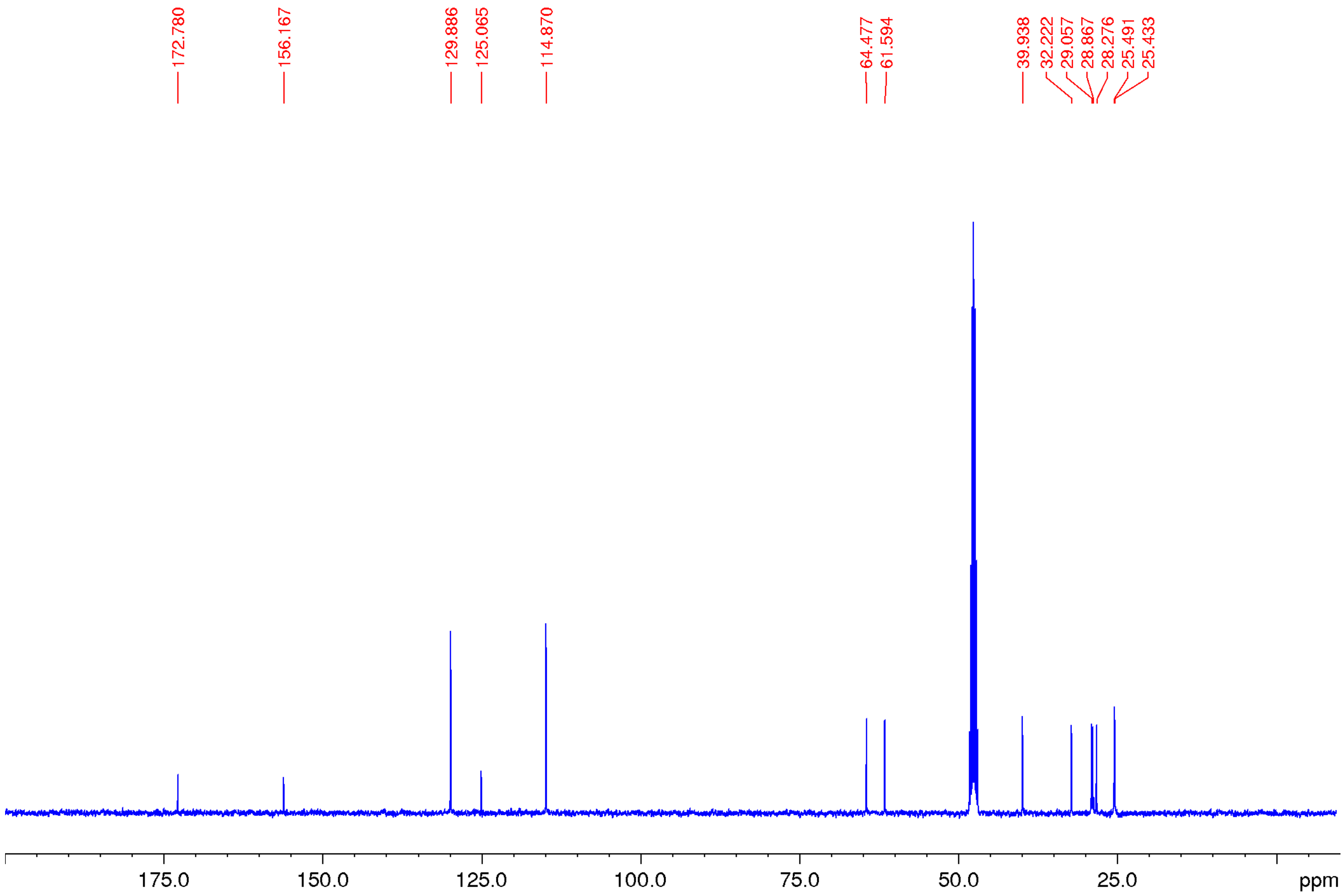

172.780
156.167
129.886
125.065
114.870
64.477
61.594
39.938
32.222
29.057
28.867
28.276
25.491
25.433
175.0
150.0
125.0
100.0
75.0
50.0
25.0
ppm

HRMS

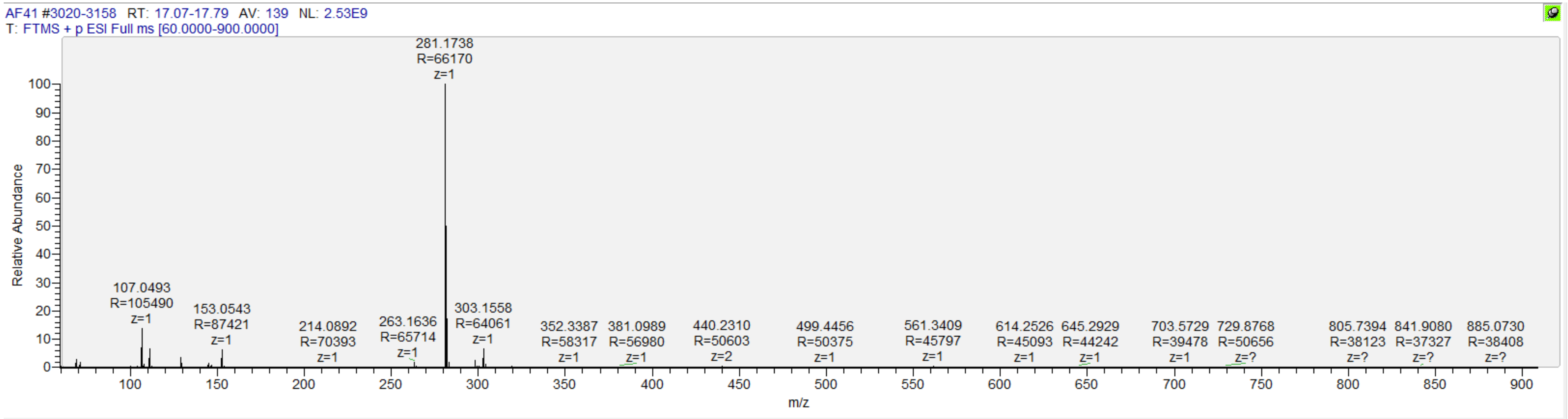
AF41 #3020-3158 RT: 17.07-17.79 AV: 139 NL: 2.53E9
T: FTMS + p ESI Full ms [60.0000-900.0000]
281.1738
R=66170
z=1
107.0493
R=105490
z=1
153.0543
R=87421
z=1
214.0892
R=70393
z=1
263.1636
R=65714
z=1
303.1558
R=64061
z=1
352.3387
R=58317
z=1
381.0989
R=56980
z=1
440.2310
R=50603
z=2
499.4456
R=50375
z=1
561.3409
R=45797
z=1
614.2526
R=45093
z=1
645.2929
R=44242
z=1
703.5729
R=39478
z=1
729.8768
R=50656
z=?
805.7394
R=38123
z=?
841.9080
R=37327
z=?
885.0730
R=38408
z=?
Relative Abundance
m/z

FTIR

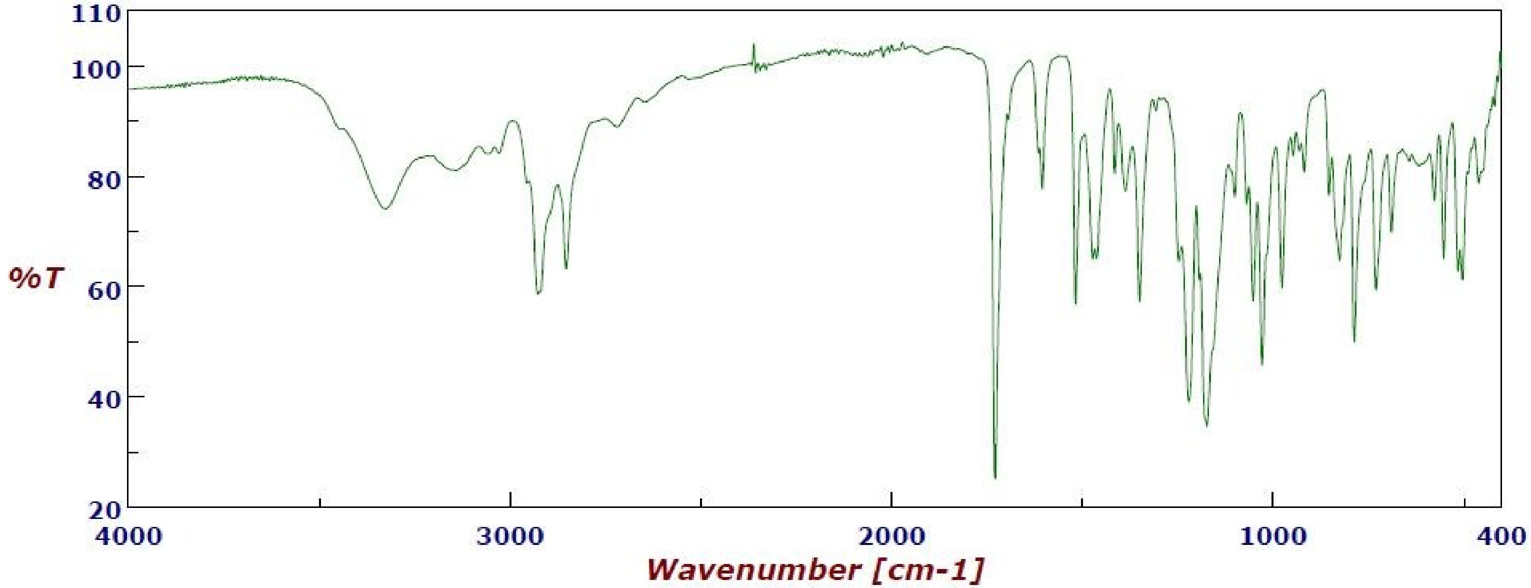
%T
Wavenumber [cm-1]

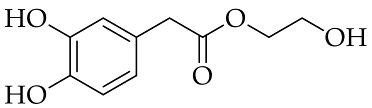

2-Hydroxyethyl 2-(3,4-dihydroxyphenyl)acetate **14**

^{1}H NMR

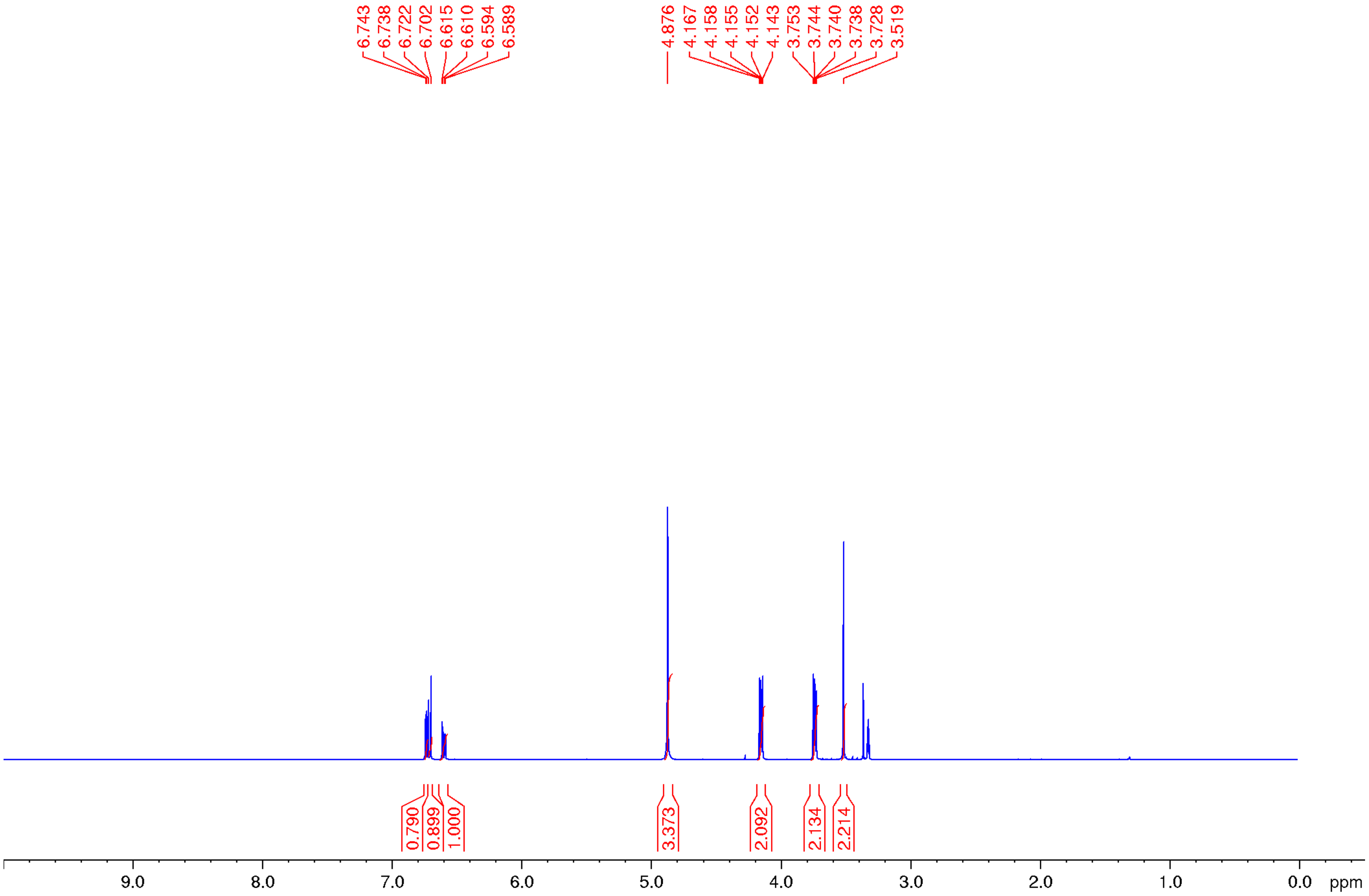

^{13}C NMR

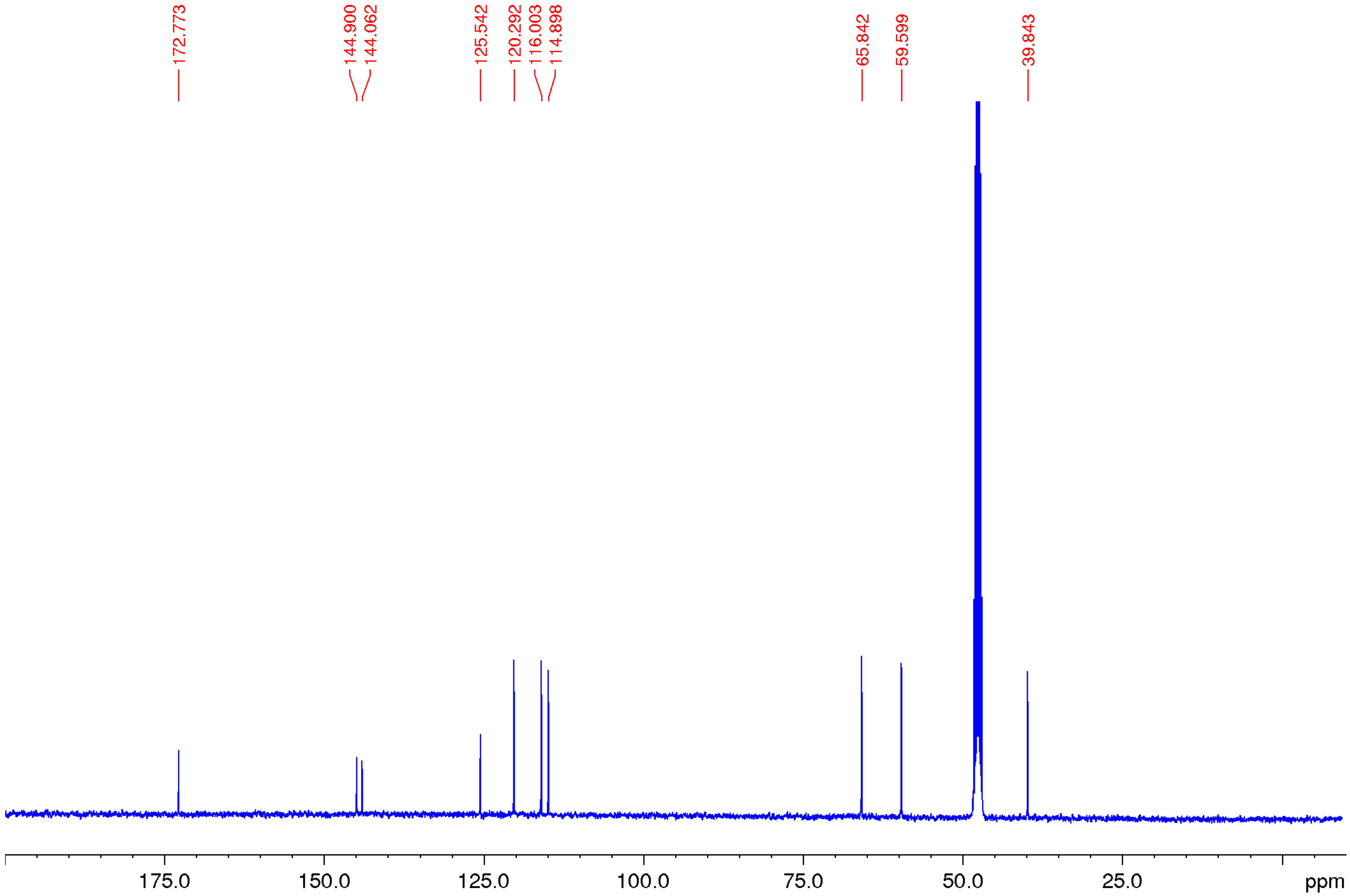
172.773
144.900
144.062
125.542
120.292
116.003
114.898
65.842
59.599
39.843
175.0
150.0
125.0
100.0
75.0
50.0
25.0
ppm

HRMS

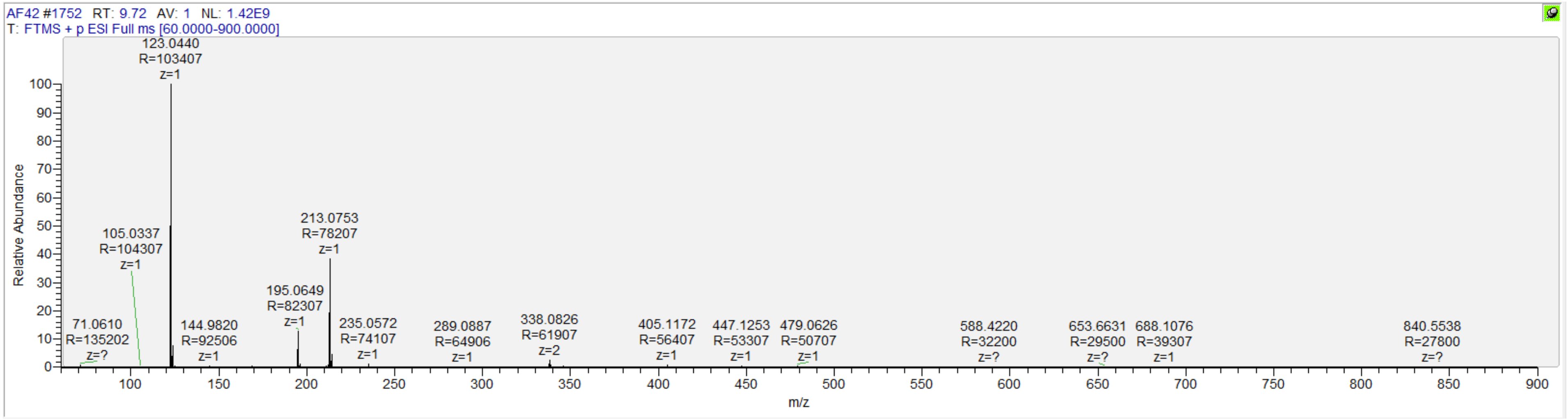
AF42 #1752 RT: 9.72 AV: 1 NL: 1.42E9
T: FTMS + p ESI Full ms [60.0000-900.0000]
123.0440
R=103407
z=1
105.0337
R=104307
z=1
213.0753
R=78207
z=1
195.0649
R=82307
z=1
71.0610
R=135202
z=?
144.9820
R=92506
z=1
235.0572
R=74107
z=1
289.0887
R=64906
z=1
338.0826
R=61907
z=2
405.1172
R=56407
z=1
447.1253
R=53307
z=1
479.0626
R=50707
z=1
588.4220
R=32200
z=?
653.6631
R=29500
z=?
688.1076
R=39307
z=1
840.5538
R=27800
z=?
Relative Abundance
m/z

FTIR

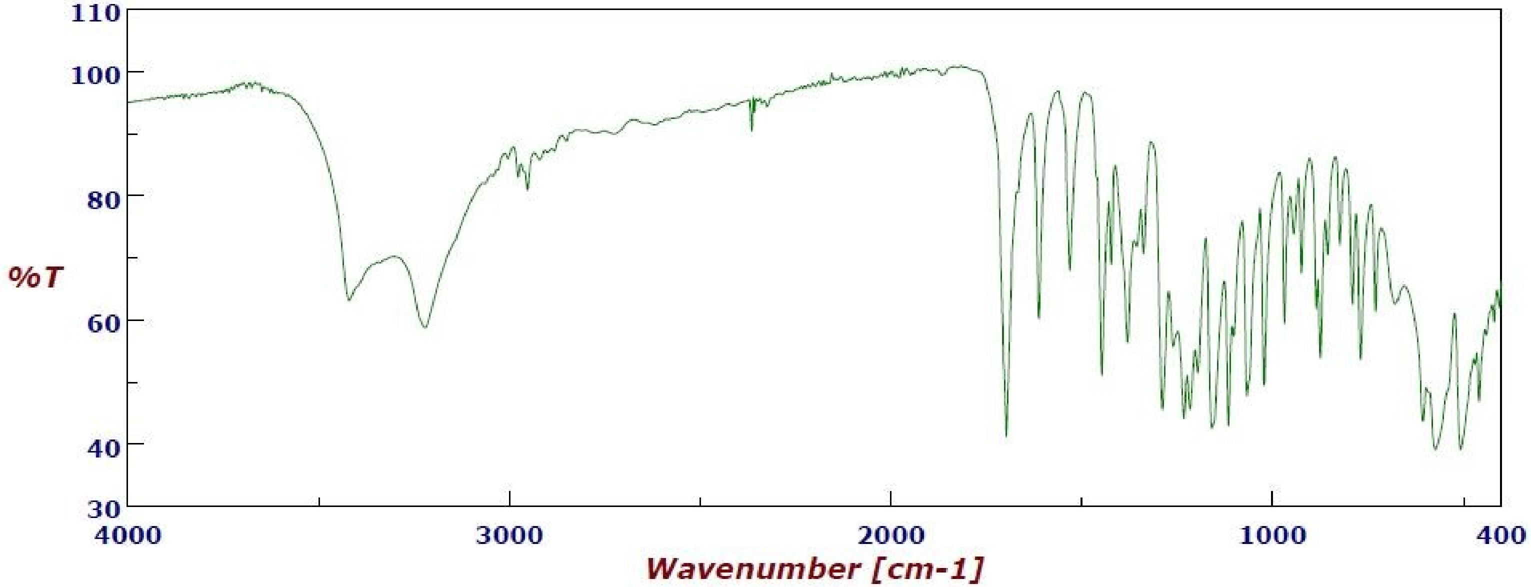
%T
Wavenumber [cm-1]

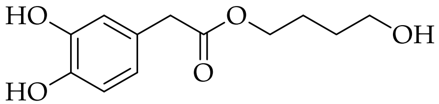

4-Hydroxybutyl 2-(3,4-dihydroxyphenyl)acetate **15**

^{1}H NMR

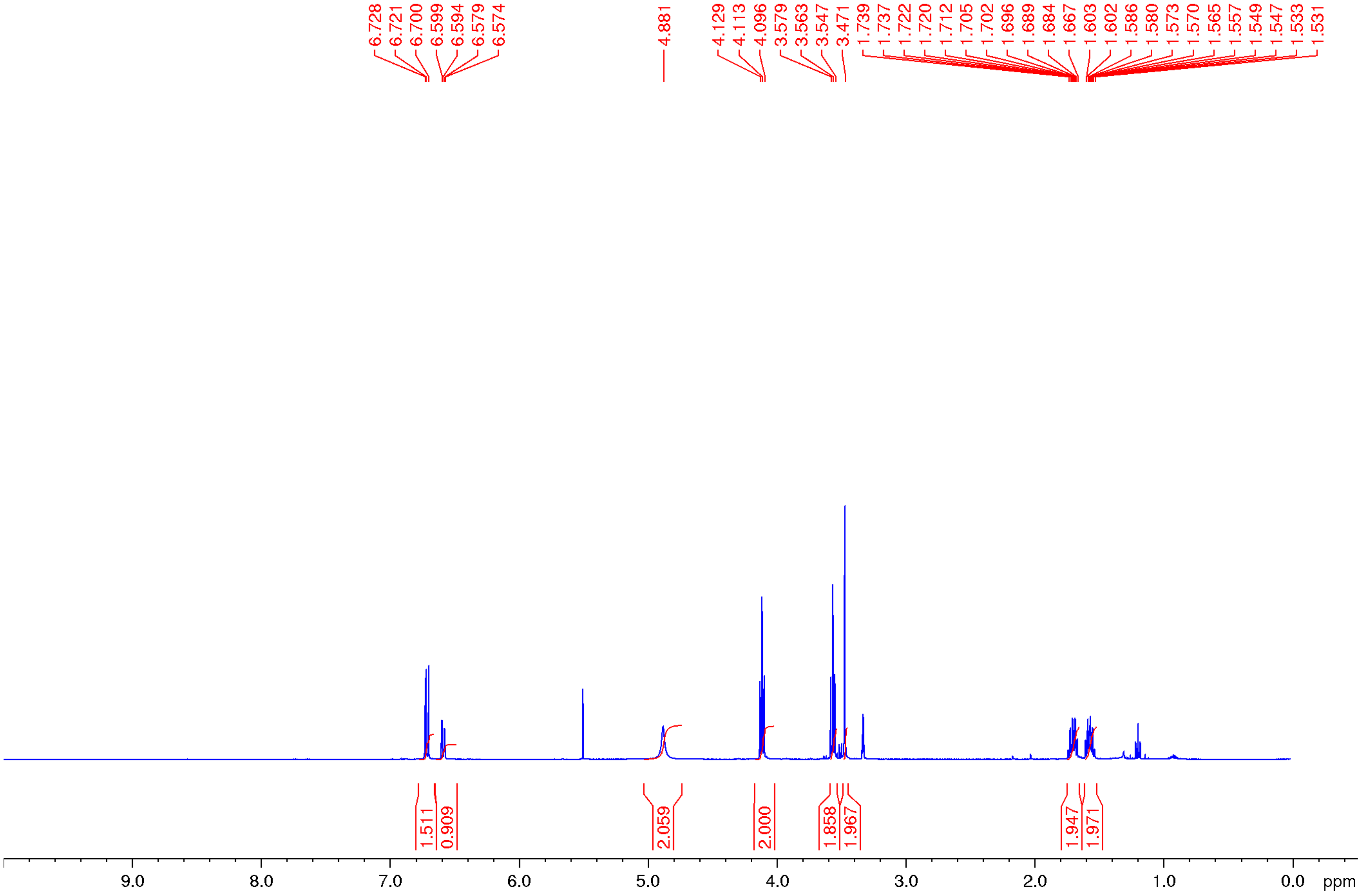

¹³C NMR

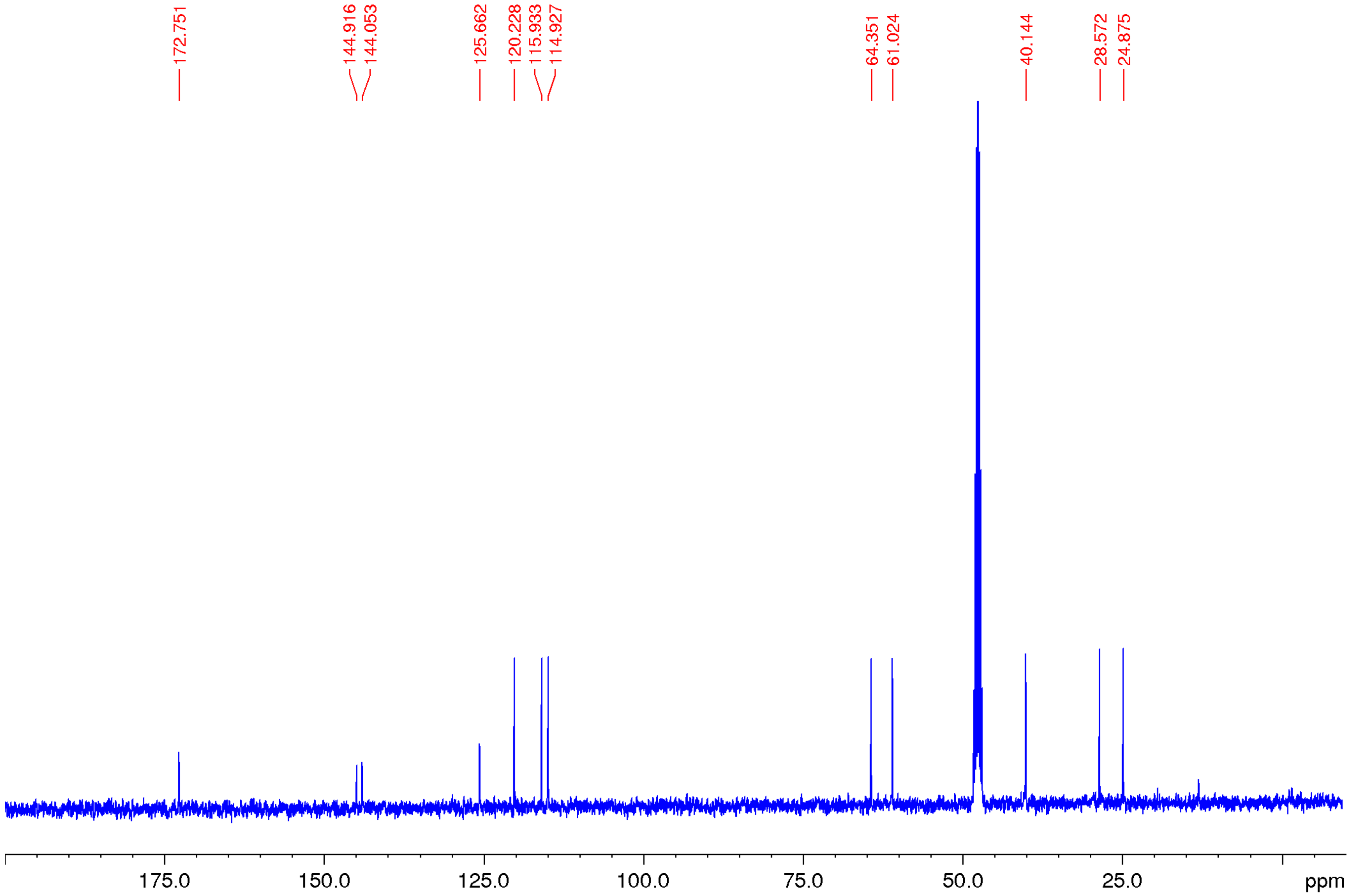
172.751
144.916
144.053
125.662
120.228
115.933
114.927
64.351
61.024
40.144
28.572
24.875
175.0
150.0
125.0
100.0
75.0
50.0
25.0
ppm

HRMS

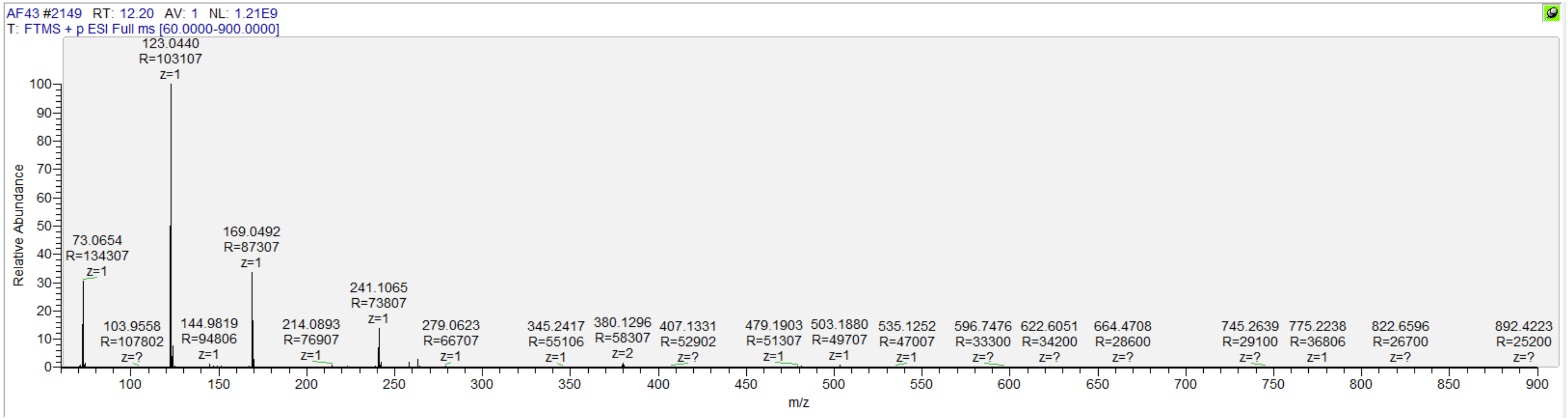
AF43 #2149 RT: 12.20 AV: 1 NL: 1.21E9
T: FTMS + p ESI Full ms [60.0000-900.0000]
123.0440
R=103107
z=1
73.0654
R=134307
z=1
169.0492
R=87307
z=1
241.1065
R=73807
z=1
103.9558
R=107802
z=?
144.9819
R=94806
z=1
214.0893
R=76907
z=1
279.0623
R=66707
z=1
345.2417
R=55106
z=1
380.1296
R=58307
z=2
407.1331
R=52902
z=?
479.1903
R=51307
z=1
503.1880
R=49707
z=1
535.1252
R=47007
z=1
596.7476
R=33300
z=?
622.6051
R=34200
z=?
664.4708
R=28600
z=?
745.2639
R=29100
z=?
775.2238
R=36806
z=1
822.6596
R=26700
z=?
892.4223
R=25200
z=?
Relative Abundance
m/z

FTIR

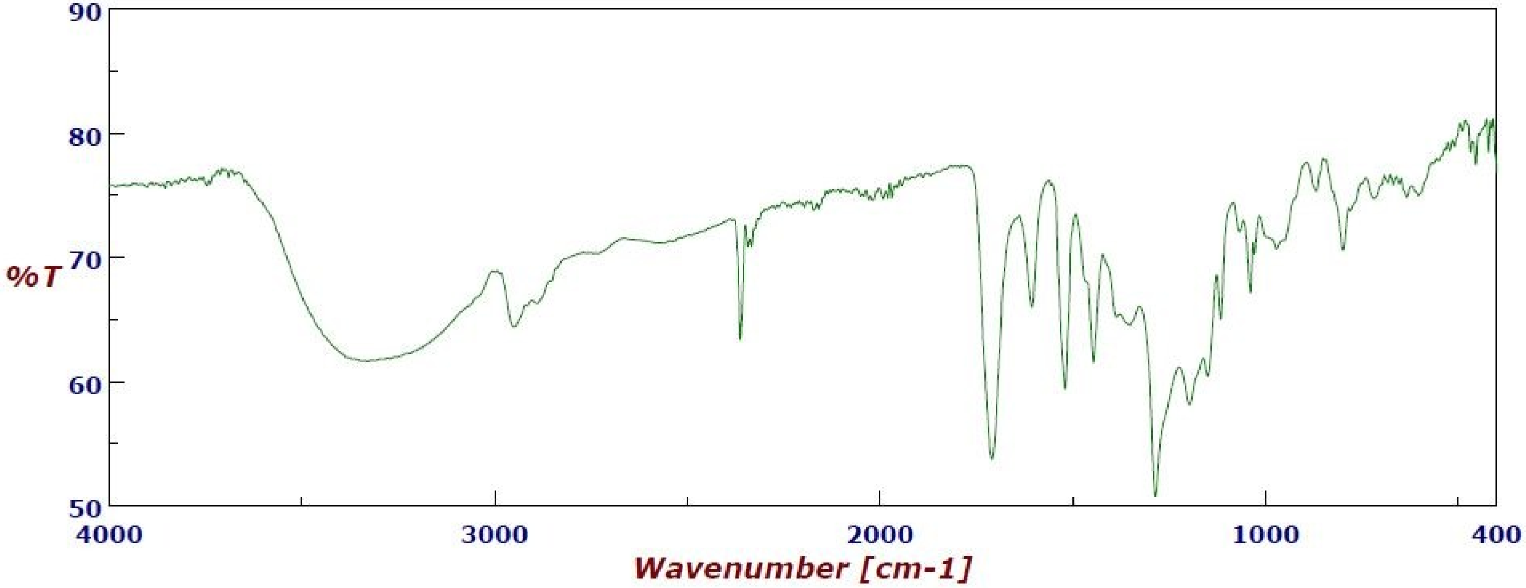
%T
Wavenumber [cm-1]

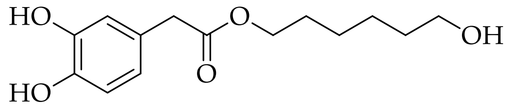

6-Hydroxyhexyl 2-(3,4-dihydroxyphenyl)acetate **16**

^{1}H NMR

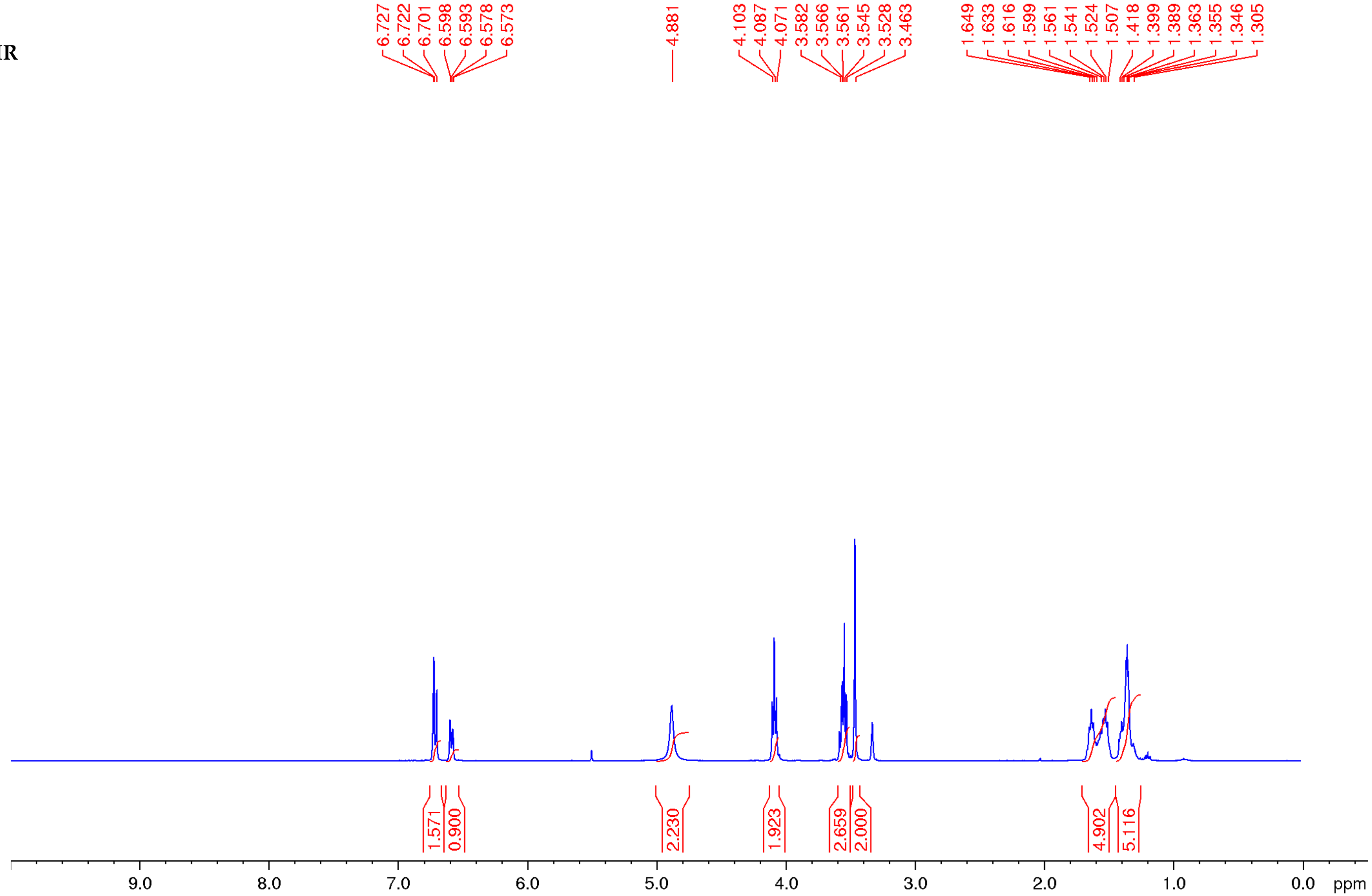

^{13}C NMR

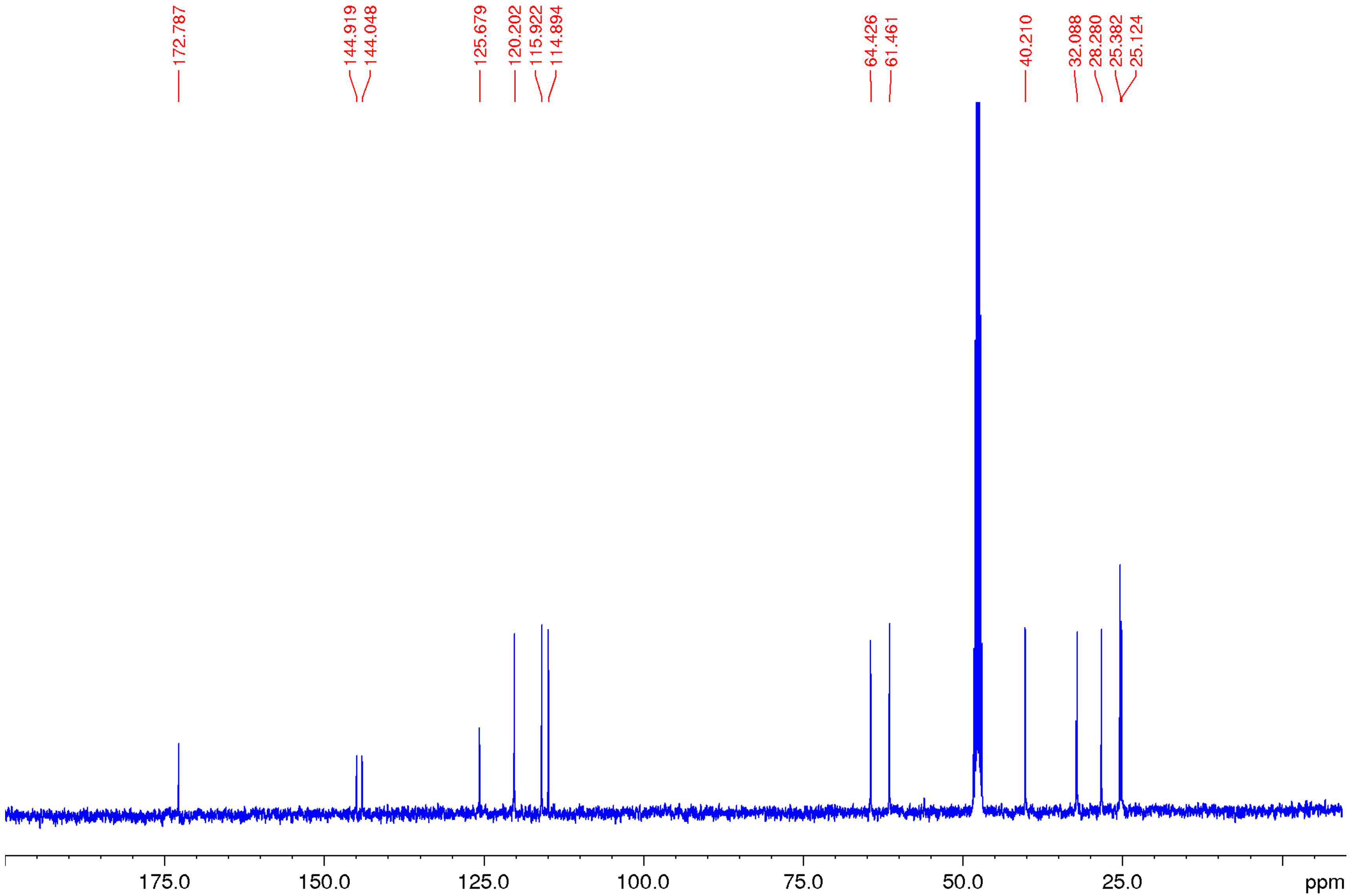

172.787
144.919
144.048
125.679
120.202
115.922
114.894
64.426
61.461
40.210
32.088
28.280
25.382
25.124
175.0
150.0
125.0
100.0
75.0
50.0
25.0
ppm

HRMS

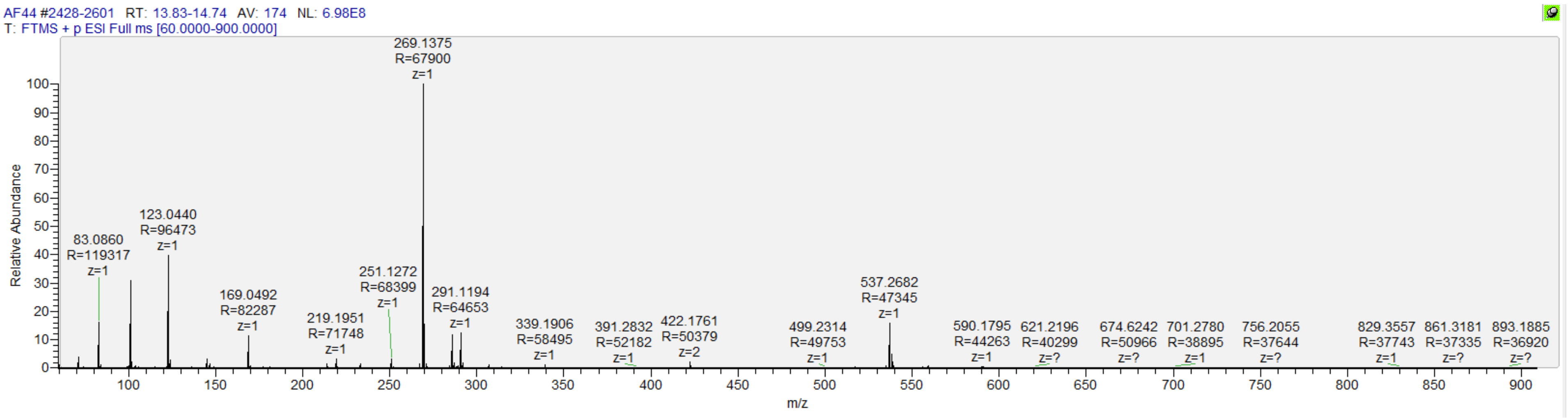
AF44 #2428-2601 RT: 13.83-14.74 AV: 174 NL: 6.98E8
T: FTMS + p ESI Full ms [60.0000-900.0000]
269.1375 R=67900 z=1
123.0440 R=96473 z=1
83.0860 R=119317 z=1
169.0492 R=82287 z=1
219.1951 R=71748 z=1
251.1272 R=68399 z=1
291.1194 R=64653 z=1
339.1906 R=58495 z=1
391.2832 R=52182 z=1
422.1761 R=50379 z=2
499.2314 R=49753 z=1
537.2682 R=47345 z=1
590.1795 R=44263 z=1
621.2196 R=40299 z=?
674.6242 R=50966 z=?
701.2780 R=38895 z=1
756.2055 R=37644 z=?
829.3557 R=37743 z=1
861.3181 R=37335 z=?
893.1885 R=36920 z=?
Relative Abundance
m/z

FTIR

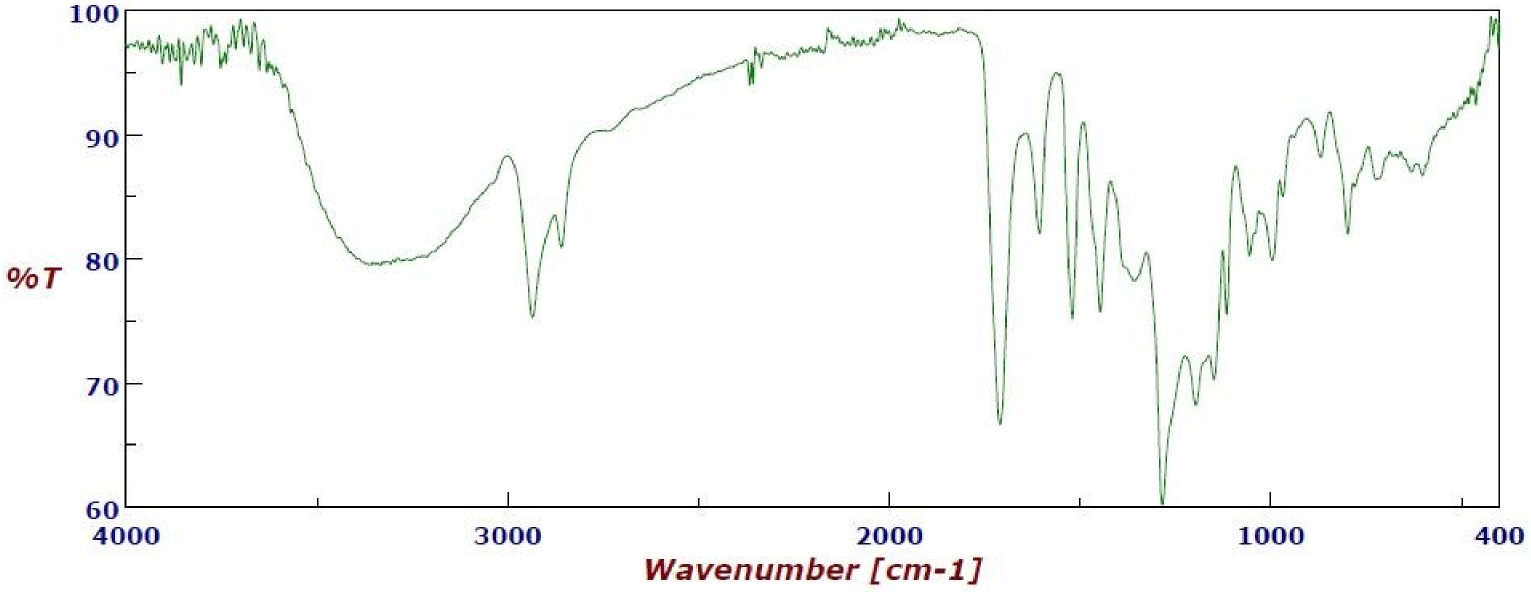
%T
Wavenumber [cm-1]

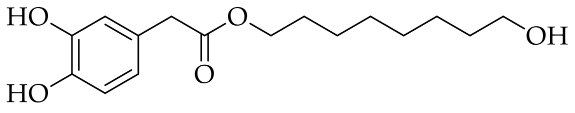

8-Hydroxyoctyl 2-(3,4-dihydroxyphenyl)acetate **17**

^{1}H NMR

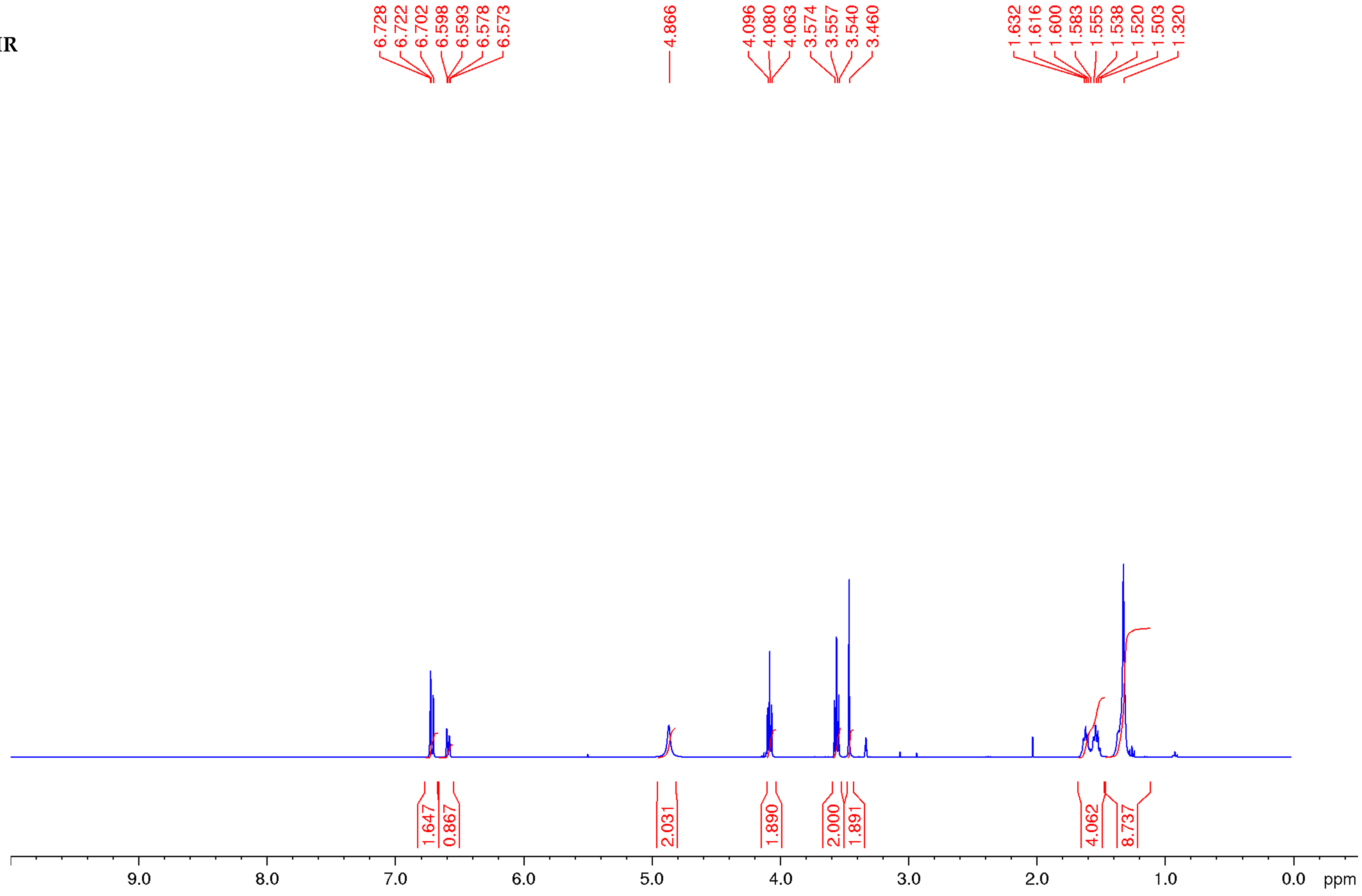

^{13}C NMR

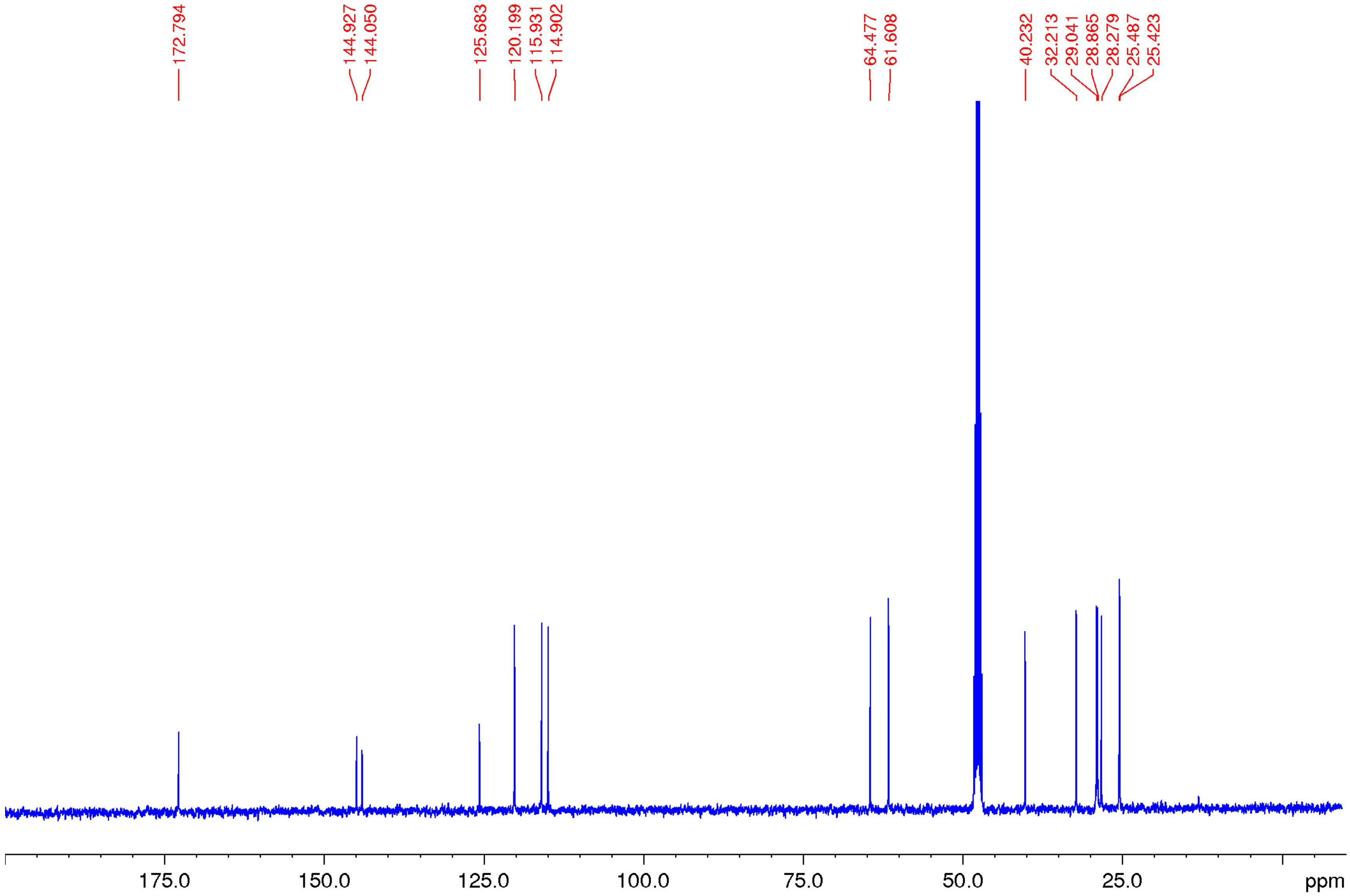

172.794
144.927
144.050
125.683
120.199
115.931
114.902
64.477
61.608
40.232
32.213
29.041
28.865
28.279
25.487
25.423
175.0
150.0
125.0
100.0
75.0
50.0
25.0
ppm

HRMS

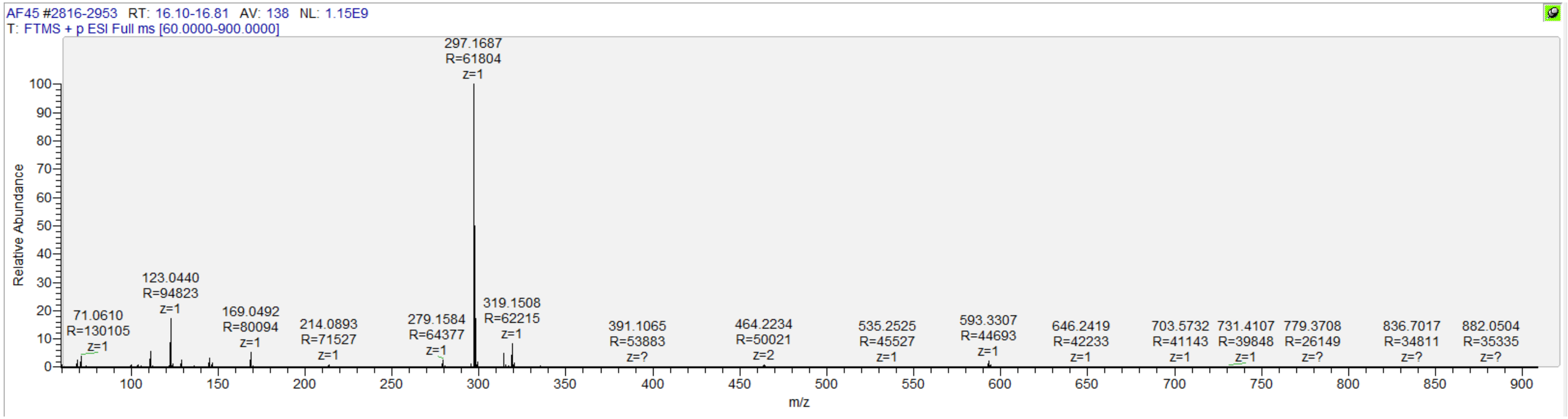
AF45 #2816-2953 RT: 16.10-16.81 AV: 138 NL: 1.15E9
T: FTMS + p ESI Full ms [60.0000-900.0000]
297.1687
R=61804
z=1
123.0440
R=94823
z=1
71.0610
R=130105
z=1
169.0492
R=80094
z=1
214.0893
R=71527
z=1
279.1584
R=64377
z=1
319.1508
R=62215
z=1
391.1065
R=53883
z=?
464.2234
R=50021
z=2
535.2525
R=45527
z=1
593.3307
R=44693
z=1
646.2419
R=42233
z=1
703.5732
R=41143
z=1
731.4107
R=39848
z=1
779.3708
R=26149
z=?
836.7017
R=34811
z=?
882.0504
R=35335
z=?
Relative Abundance
m/z

FTIR

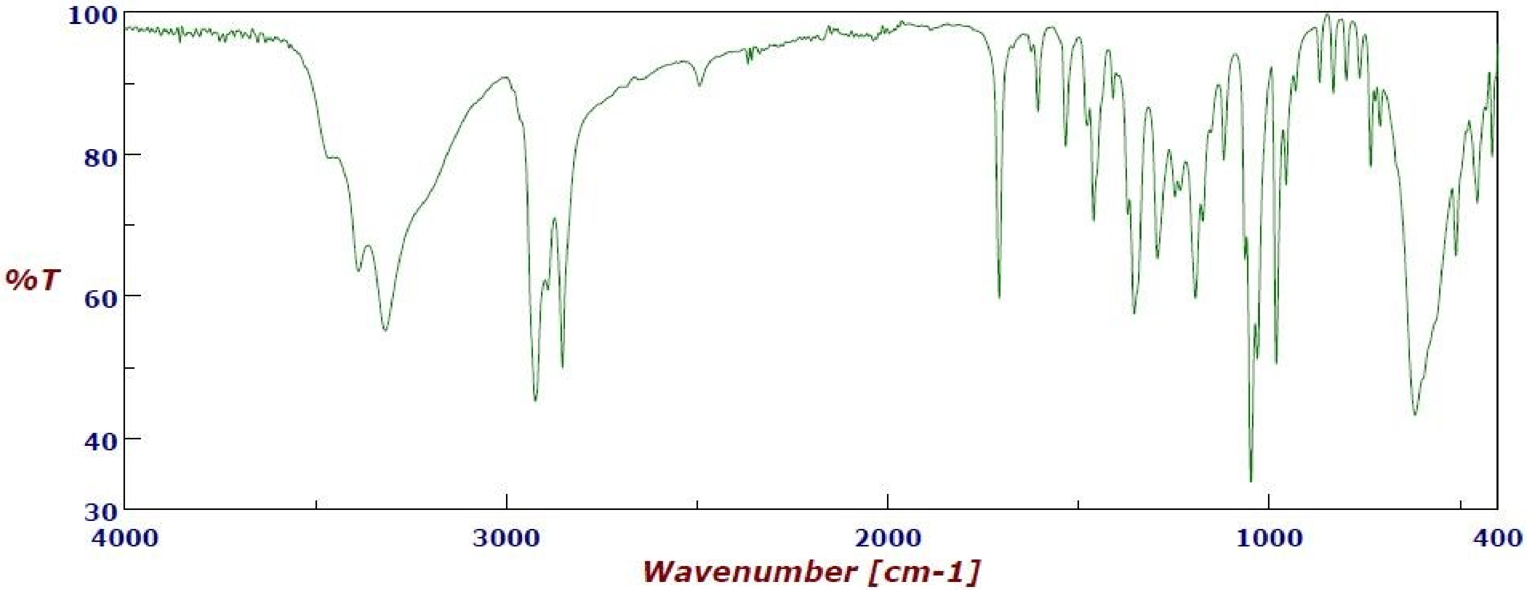
%T
Wavenumber [cm-1]

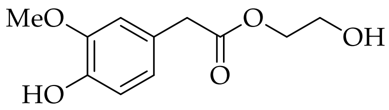

2-Hydroxyethyl 2-(4-hydroxy-3-methoxyphenyl)acetate **18**

^{1}H NMR

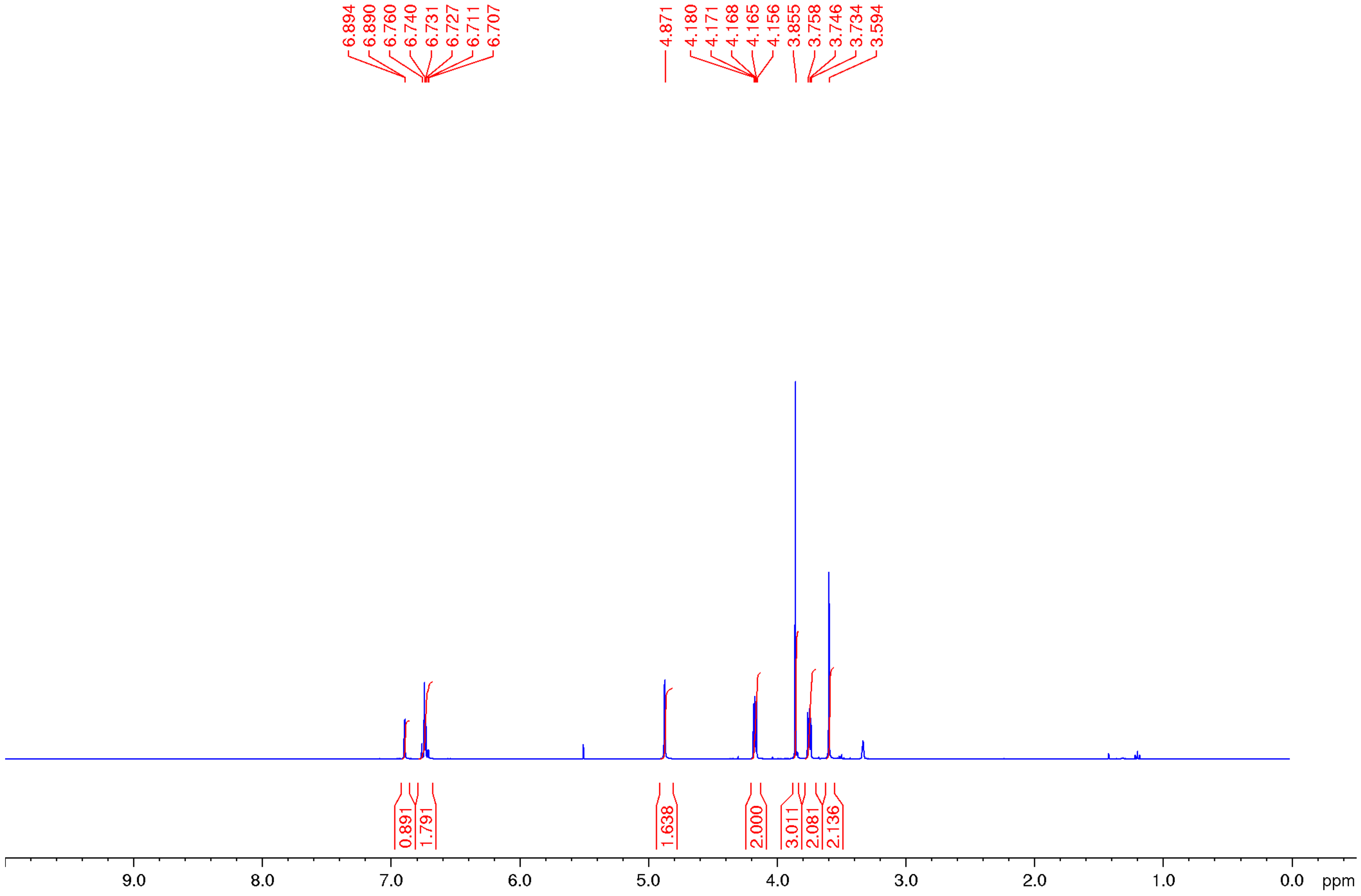

^{13}C NMR

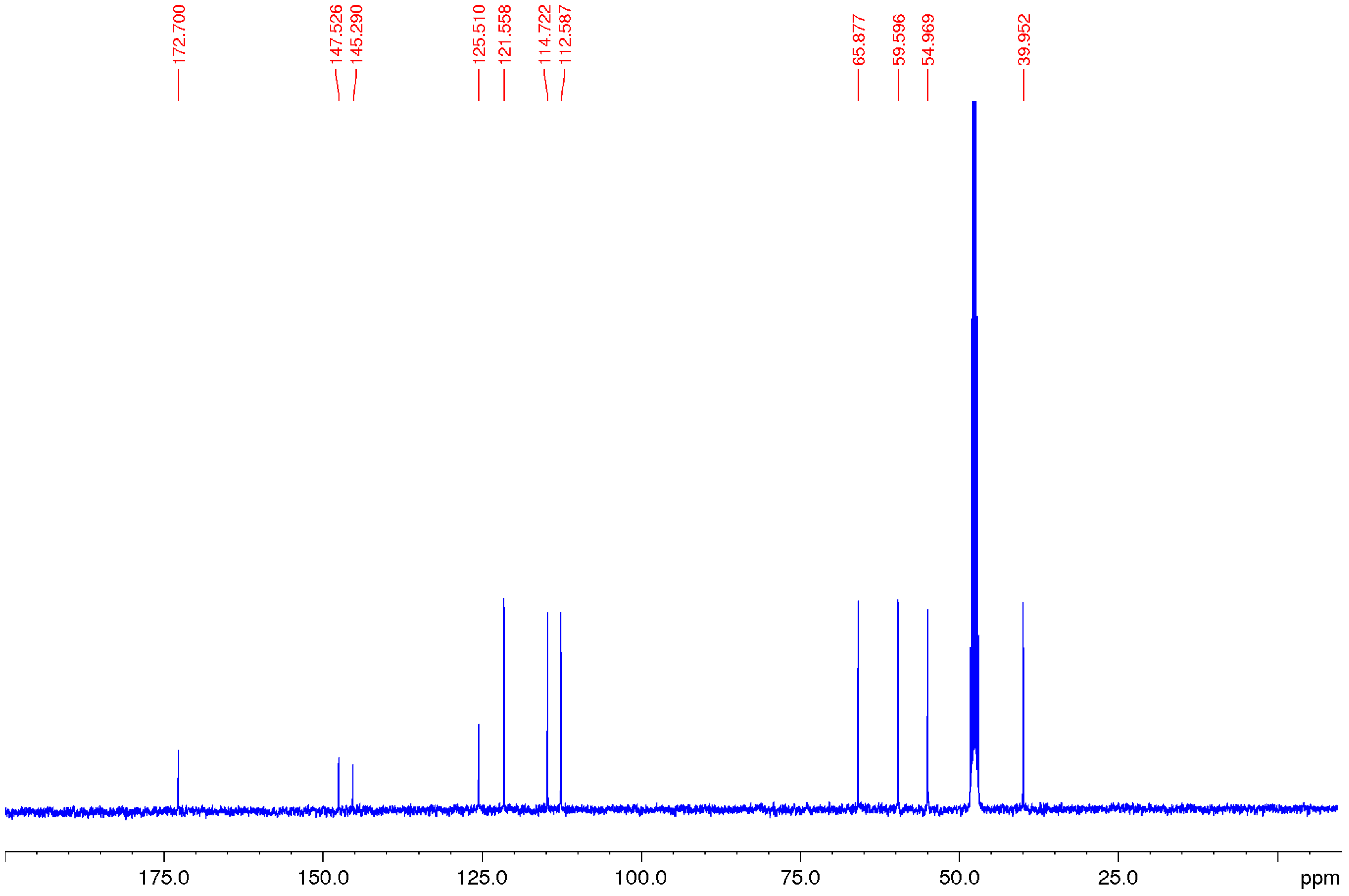
172.700
147.526
145.290
125.510
121.558
114.722
112.587
65.877
59.596
54.969
39.952
175.0
150.0
125.0
100.0
75.0
50.0
25.0
ppm

HRMS

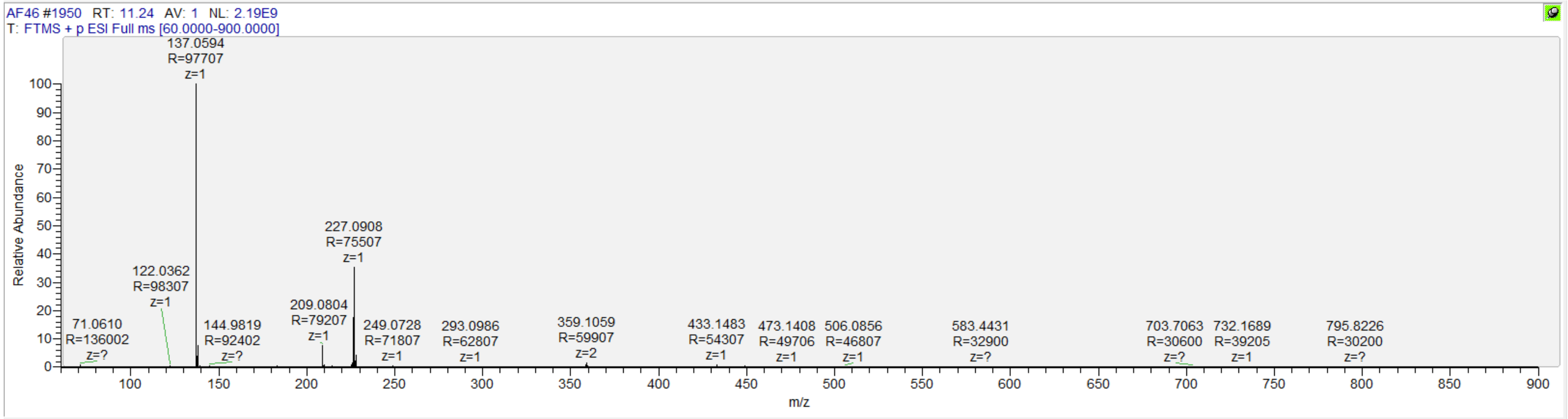
AF46 #1950 RT: 11.24 AV: 1 NL: 2.19E9
T: FTMS + p ESI Full ms [60.0000-900.0000]
137.0594
R=97707
z=1
227.0908
R=75507
z=1
122.0362
R=98307
z=1
209.0804
R=79207
z=1
71.0610
R=136002
z=?
144.9819
R=92402
z=?
249.0728
R=71807
z=1
293.0986
R=62807
z=1
359.1059
R=59907
z=2
433.1483
R=54307
z=1
473.1408
R=49706
z=1
506.0856
R=46807
z=1
583.4431
R=32900
z=?
703.7063
R=30600
z=?
732.1689
R=39205
z=1
795.8226
R=30200
z=?
Relative Abundance
m/z

FTIR

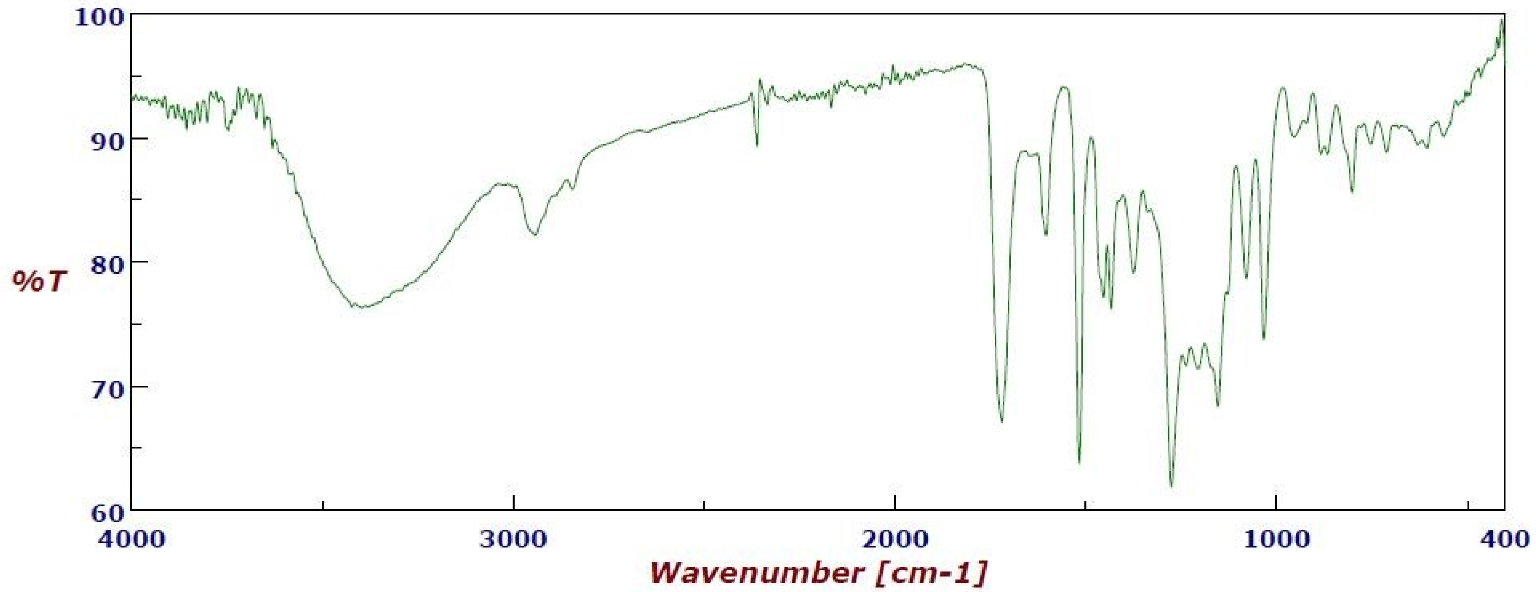
%T
Wavenumber [cm-1]

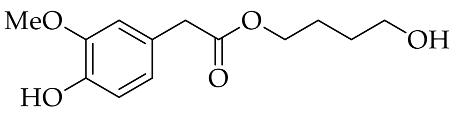

4-Hydroxybutyl 2-(4-hydroxy-3-methoxyphenyl)acetate **19**

^{1}H NMR

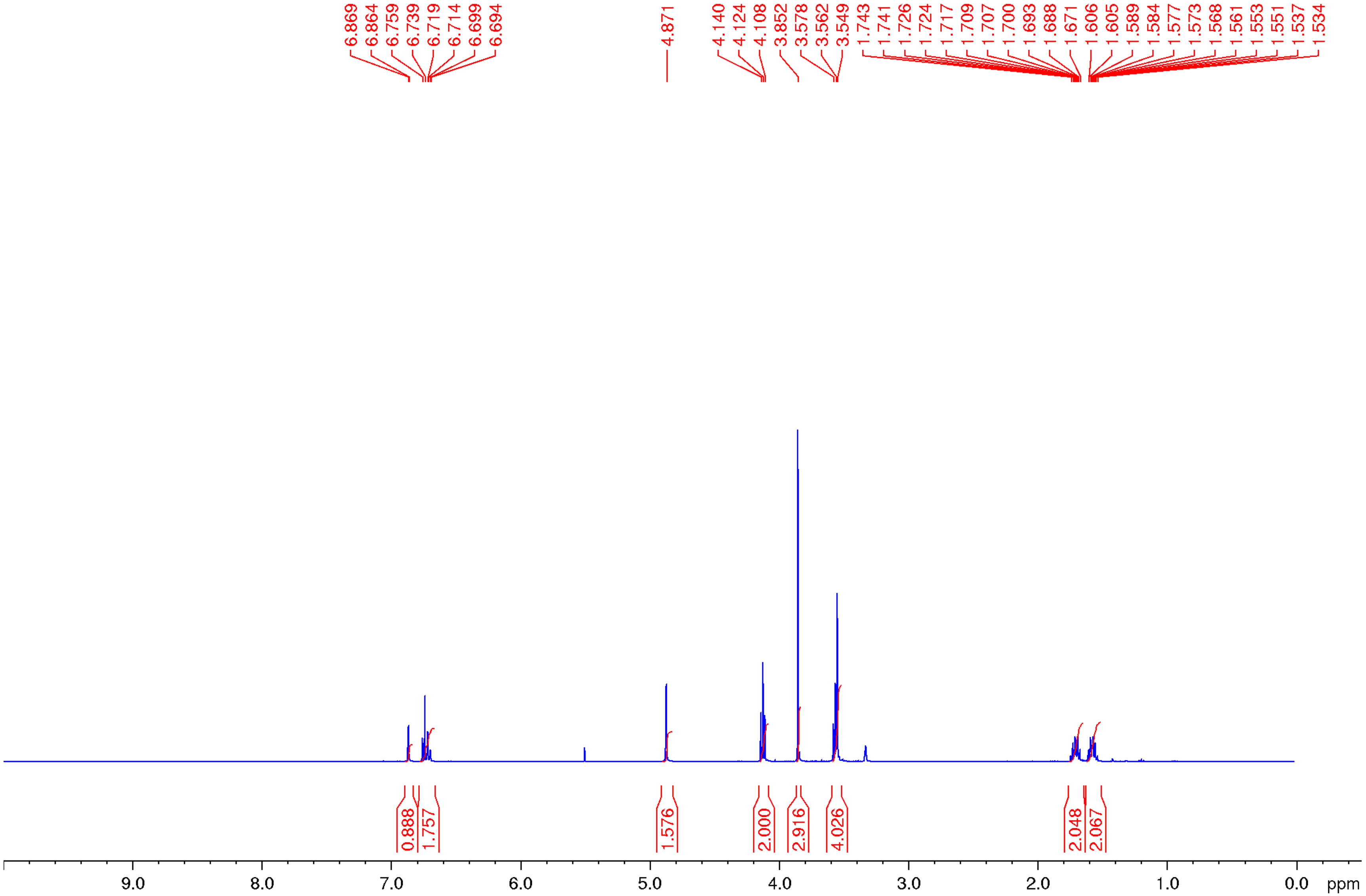

^{13}C NMR

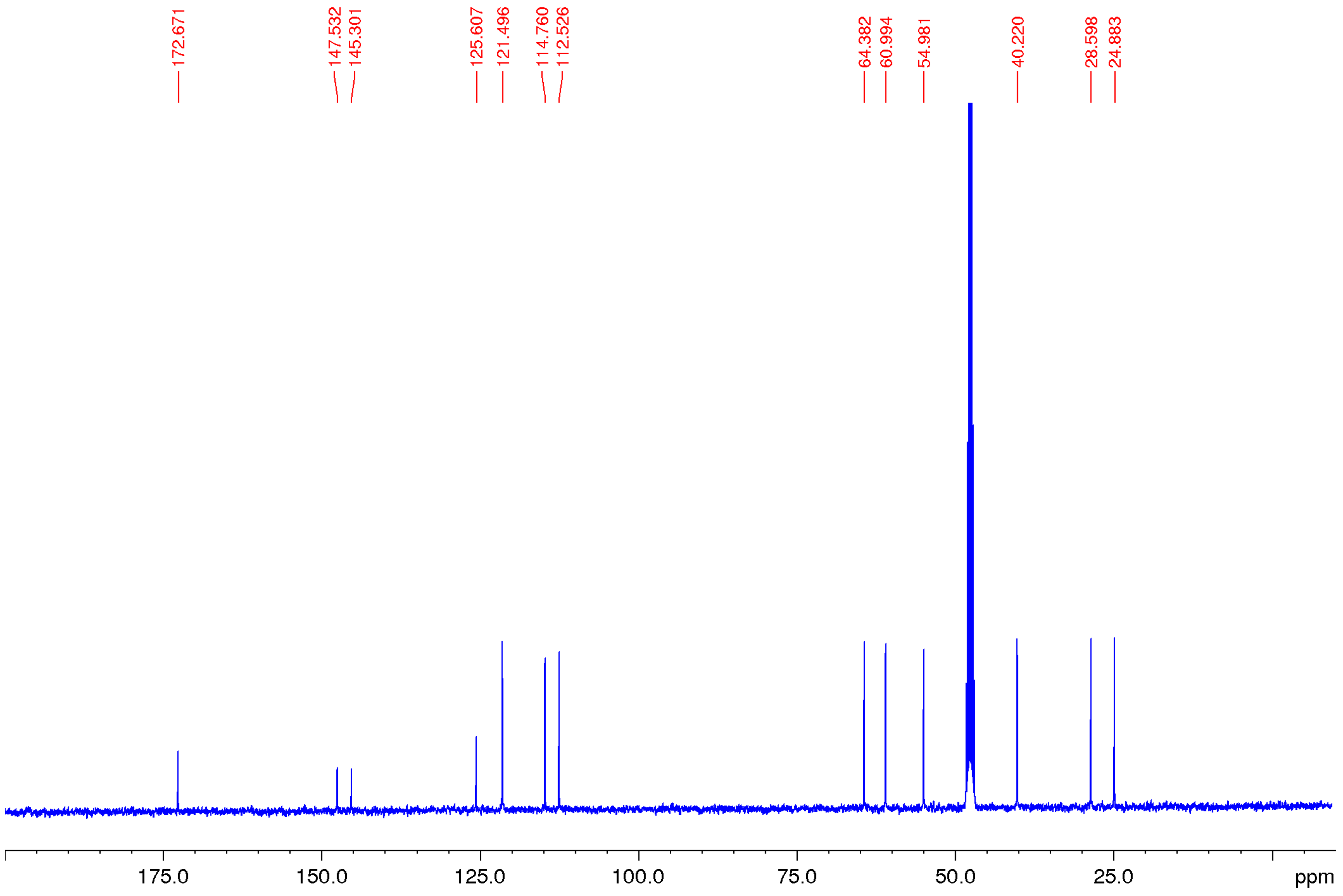
172.671
147.532
145.301
125.607
121.496
114.760
112.526
64.382
60.994
54.981
40.220
28.598
24.883
175.0
150.0
125.0
100.0
75.0
50.0
25.0
ppm

HRMS

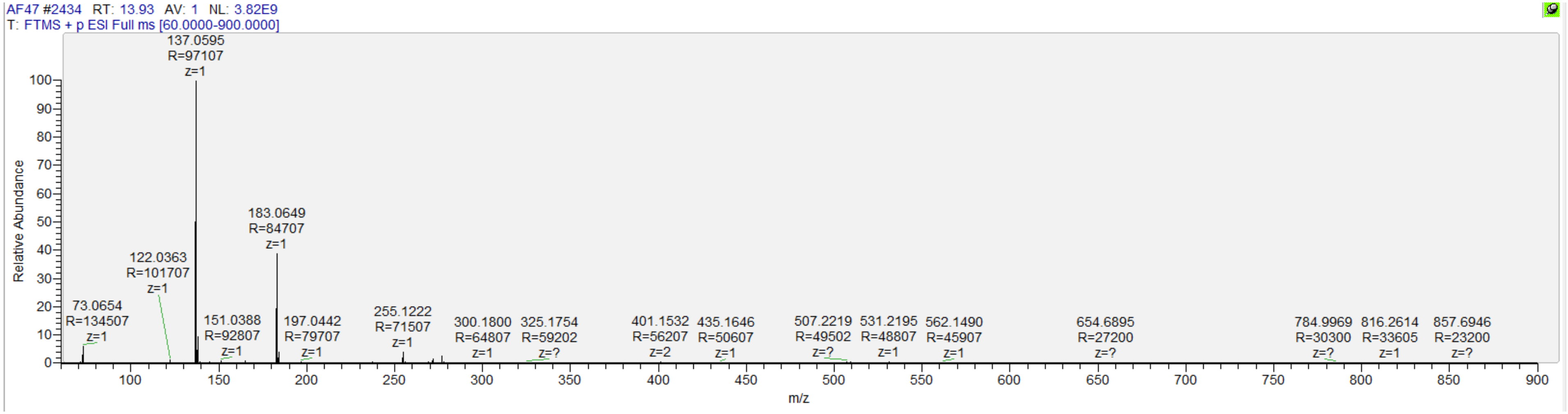
AF47 #2434 RT: 13.93 AV: 1 NL: 3.82E9
T: FTMS + p ESI Full ms [60.0000-900.0000]
137.0595
R=97107
z=1
183.0649
R=84707
z=1
122.0363
R=101707
z=1
73.0654
R=134507
z=1
151.0388
R=92807
z=1
197.0442
R=79707
z=1
255.1222
R=71507
z=1
300.1800
R=64807
z=1
325.1754
R=59202
z=?
401.1532
R=56207
z=2
435.1646
R=50607
z=1
507.2219
R=49502
z=?
531.2195
R=48807
z=1
562.1490
R=45907
z=1
654.6895
R=27200
z=?
784.9969
R=30300
z=?
816.2614
R=33605
z=1
857.6946
R=23200
z=?
Relative Abundance
m/z

FTIR

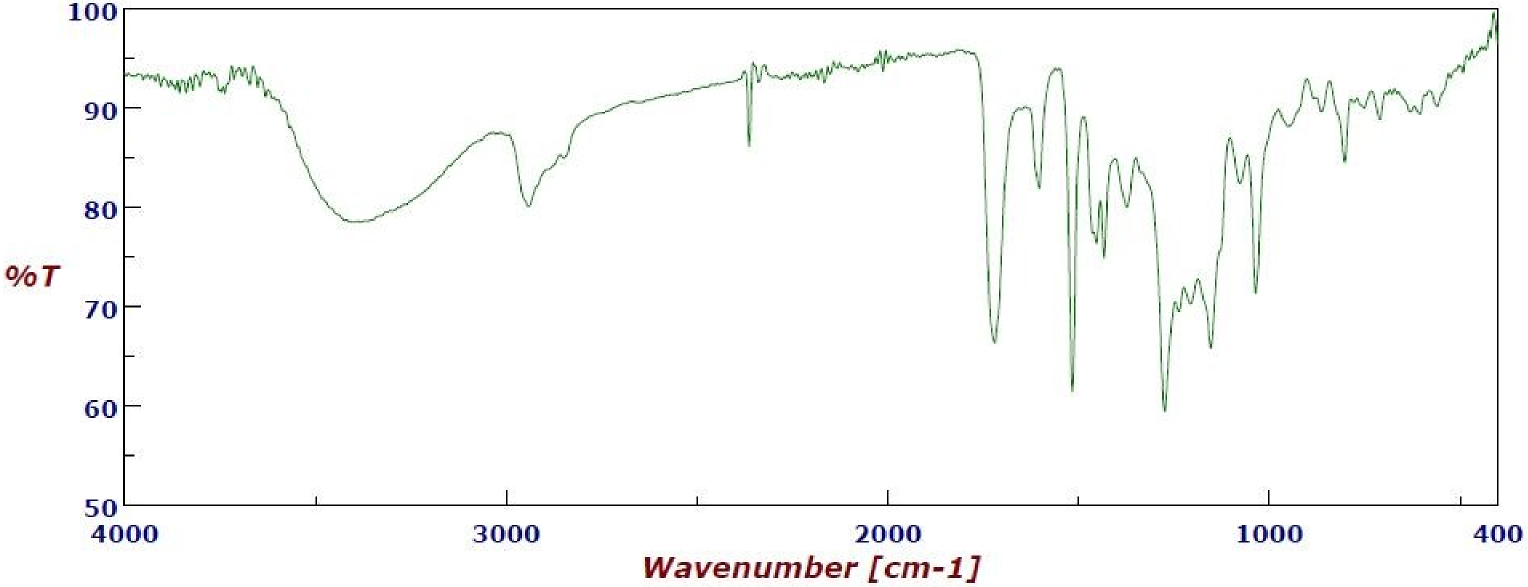
%T
Wavenumber [cm-1]

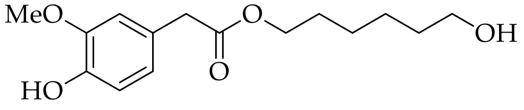

6-Hydroxyhexyl 2-(4-hydroxy-3-methoxyphenyl)acetat*e* **20**

^{1}H NMR

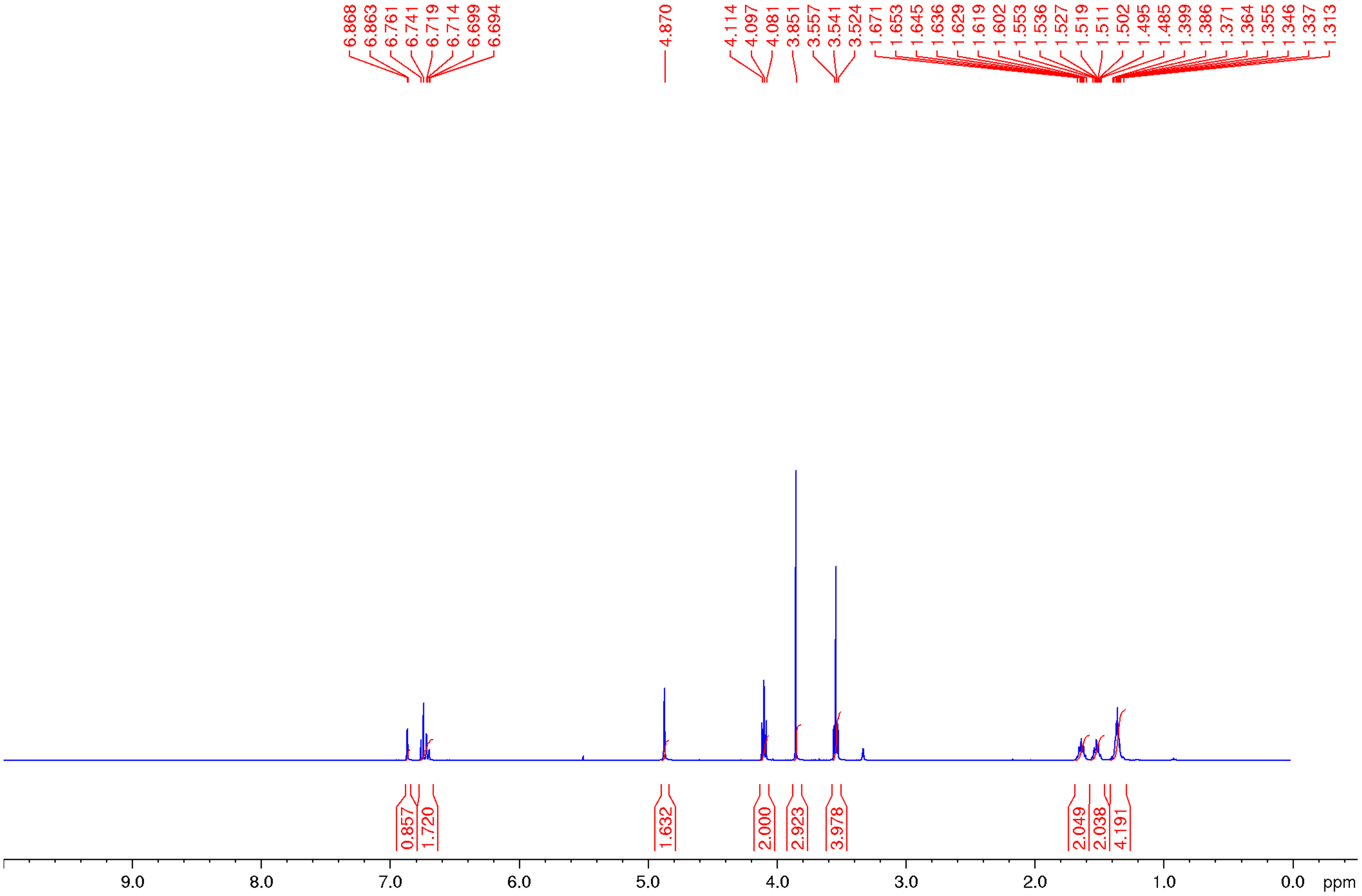

¹³C NMR

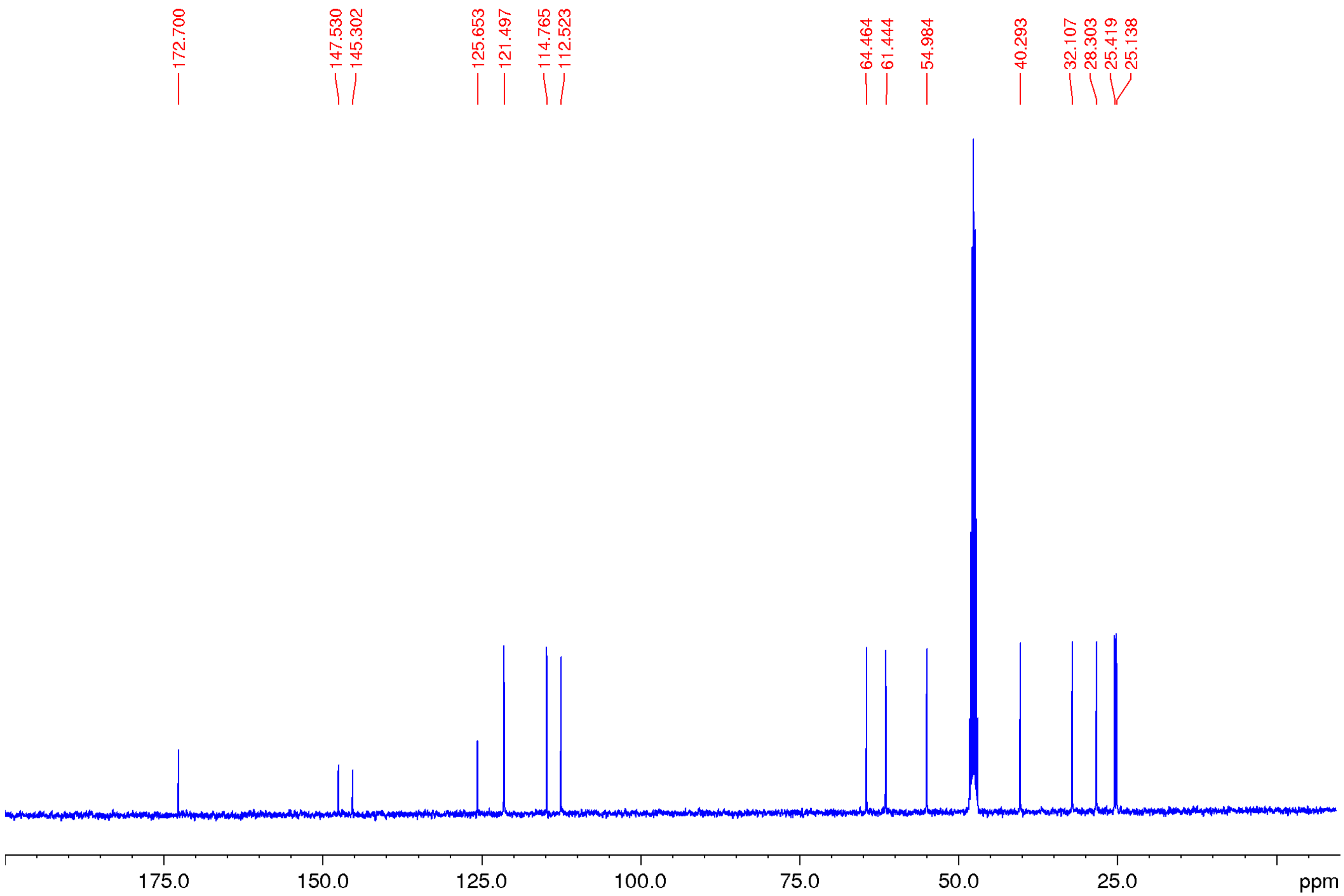

172.700
147.530
145.302
125.653
121.497
114.765
112.523
64.464
61.444
54.984
40.293
32.107
28.303
25.419
25.138
175.0
150.0
125.0
100.0
75.0
50.0
25.0
ppm

HRMS

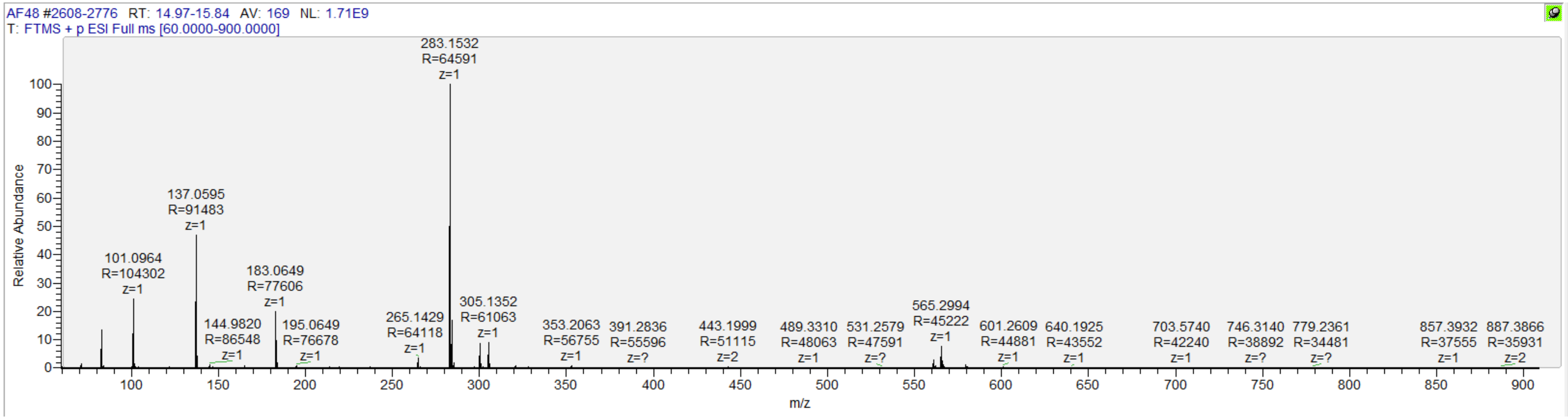
AF48 #2608-2776 RT: 14.97-15.84 AV: 169 NL: 1.71E9
T: FTMS + p ESI Full ms [60.0000-900.0000]
283.1532 R=64591 z=1
137.0595 R=91483 z=1
101.0964 R=104302 z=1
183.0649 R=77606 z=1
144.9820 R=86548 z=1
195.0649 R=76678 z=1
265.1429 R=64118 z=1
305.1352 R=61063 z=1
353.2063 R=56755 z=1
391.2836 R=55596 z=?
443.1999 R=51115 z=2
489.3310 R=48063 z=1
531.2579 R=47591 z=?
565.2994 R=45222 z=1
601.2609 R=44881 z=1
640.1925 R=43552 z=1
703.5740 R=42240 z=1
746.3140 R=38892 z=?
779.2361 R=34481 z=?
857.3932 R=37555 z=1
887.3866 R=35931 z=2
Relative Abundance
m/z

FTIR

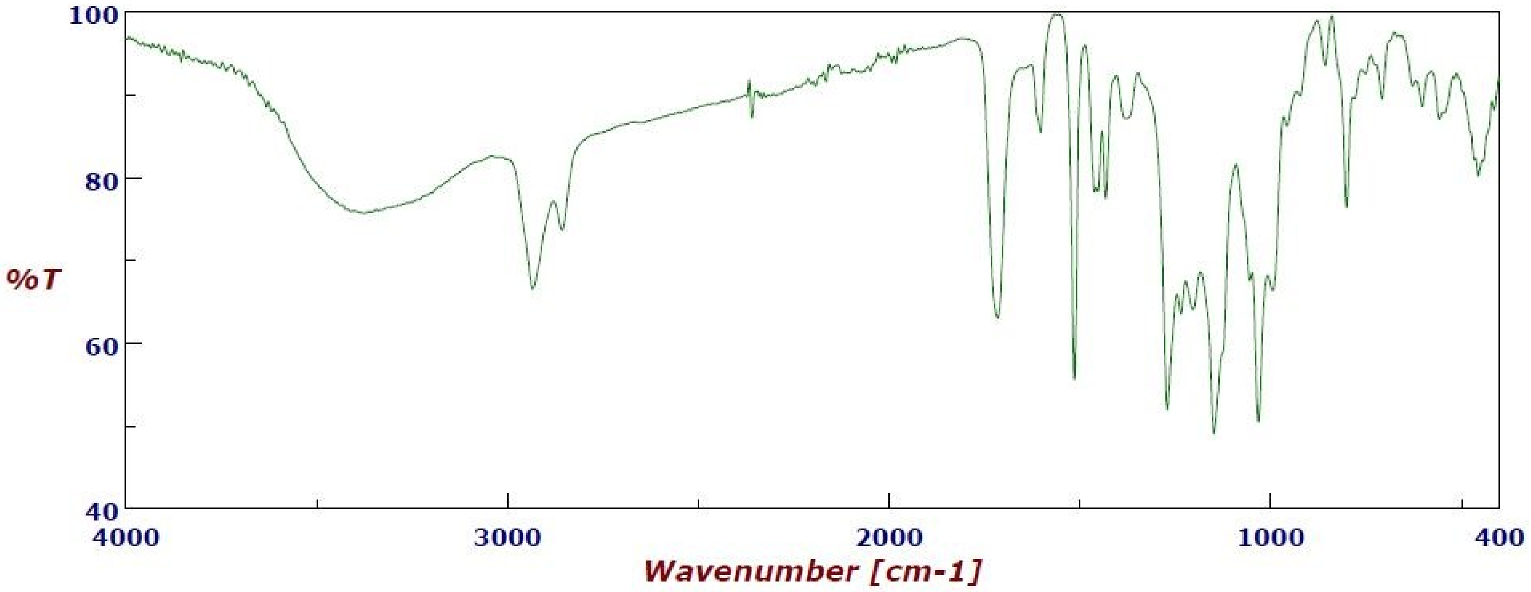
%T
Wavenumber [cm-1]

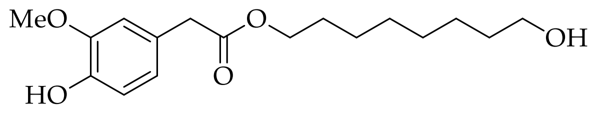

8-Hydroxyoctyl 2-(4-hydroxy-3-methoxyphenyl)acetate **21**

^{1}**H NMR**

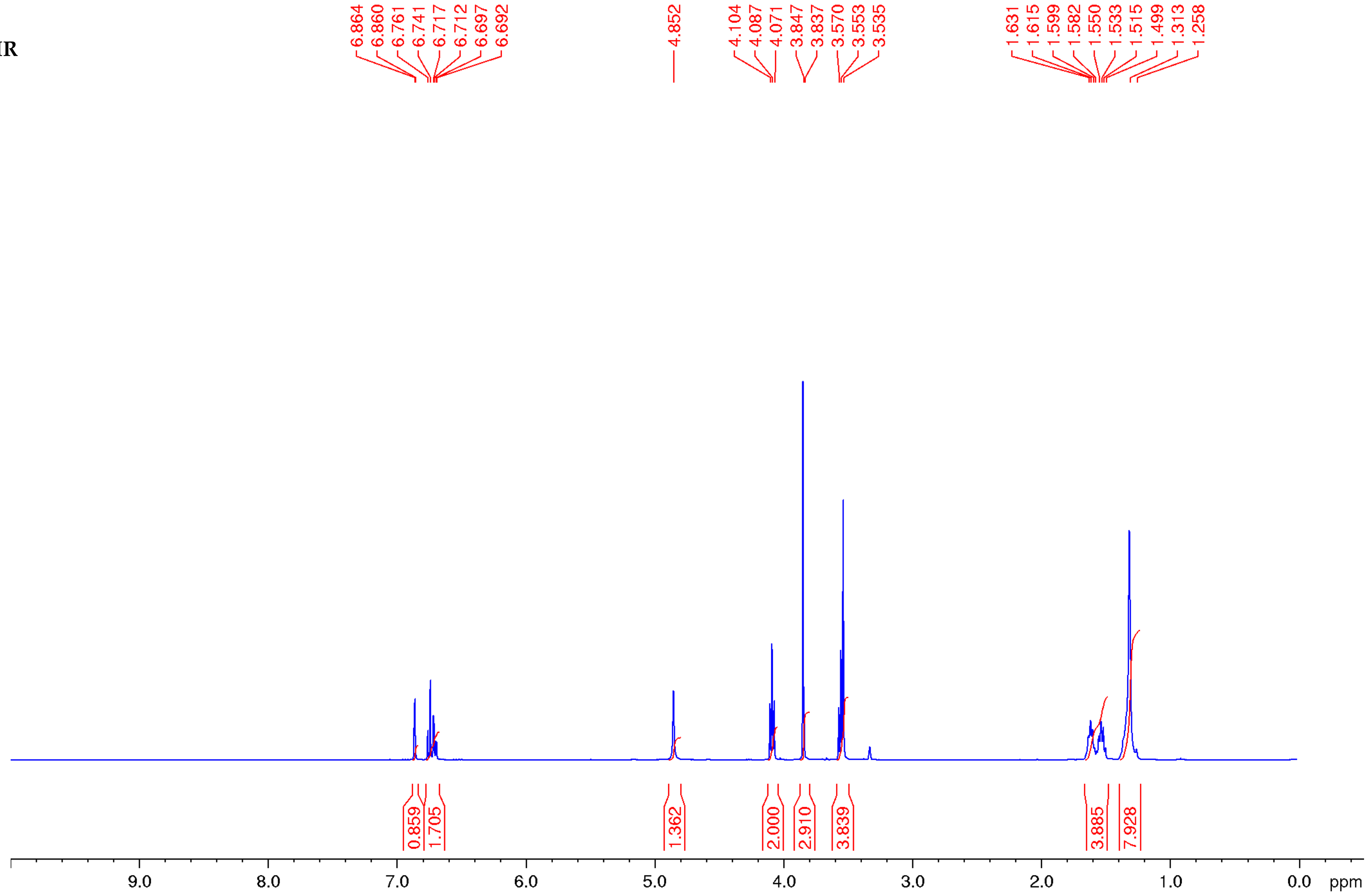

^{13}C NMR

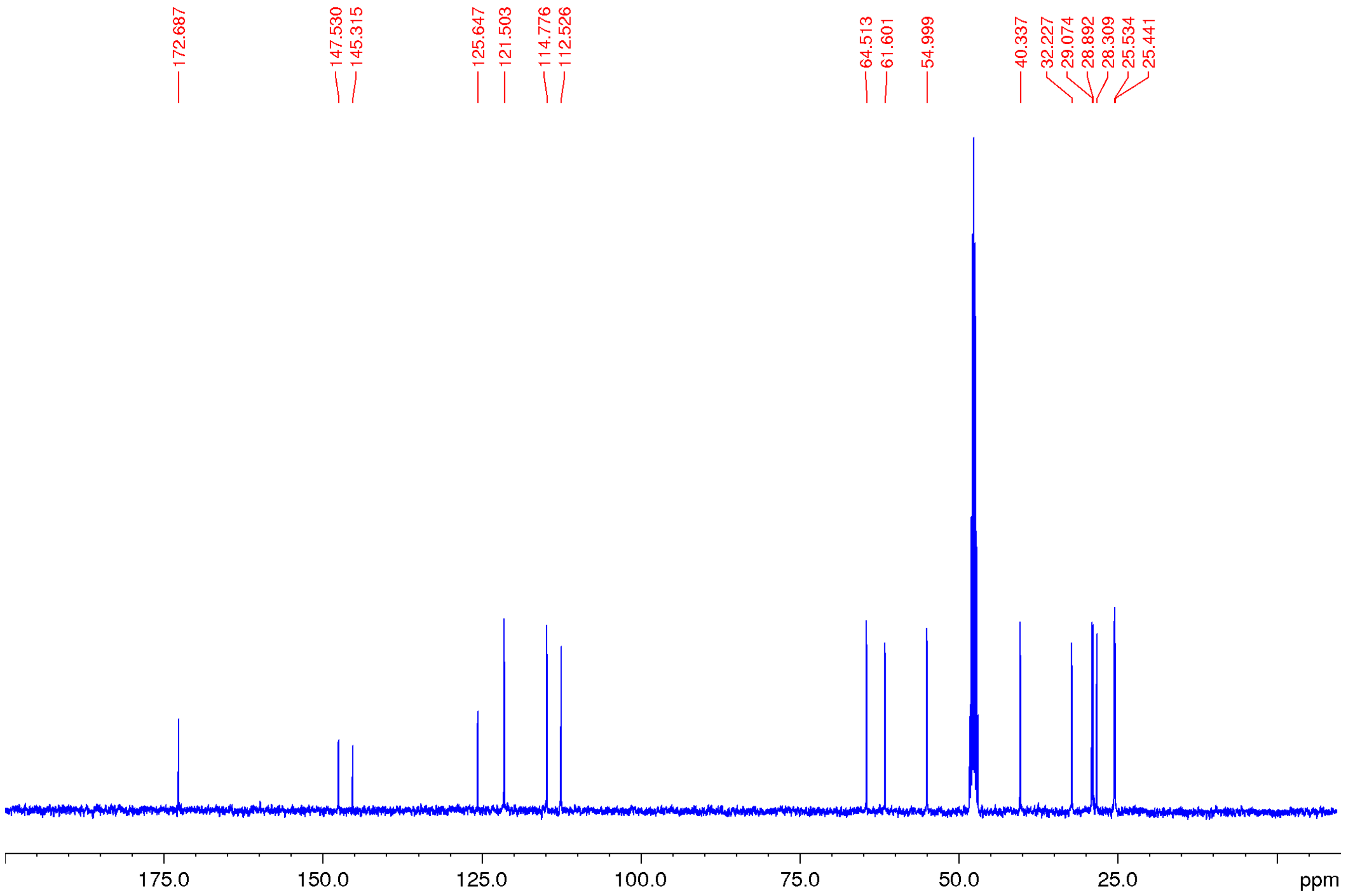
172.687
147.530
145.315
125.647
121.503
114.776
112.526
64.513
61.601
54.999
40.337
32.227
29.074
28.892
28.309
25.534
25.441
175.0
150.0
125.0
100.0
75.0
50.0
25.0
ppm

HRMS

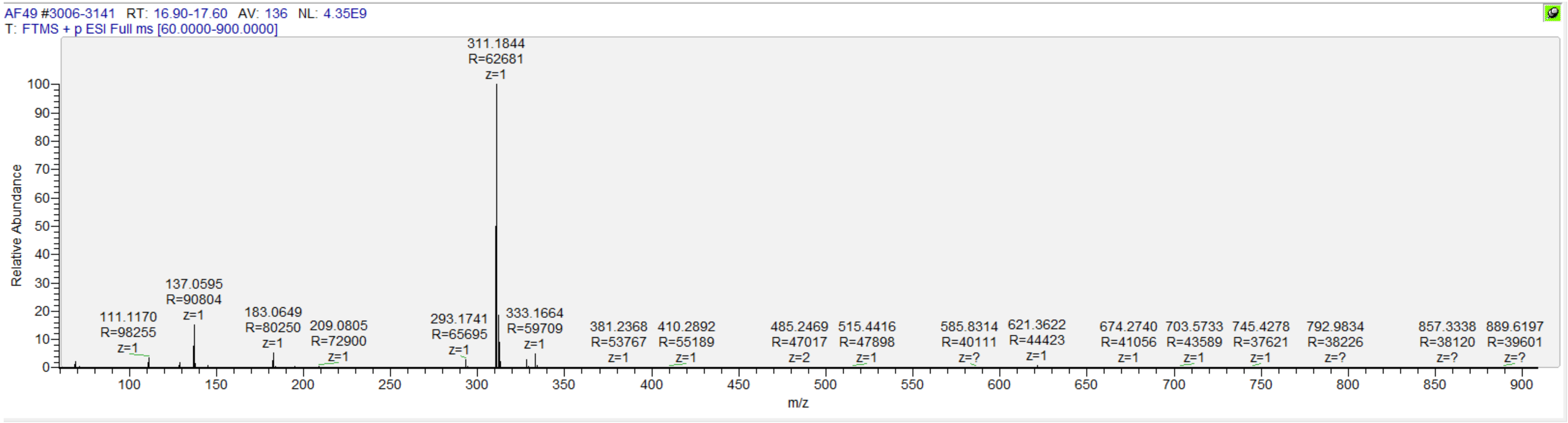
AF49 #3006-3141 RT: 16.90-17.60 AV: 136 NL: 4.35E9
T: FTMS + p ESI Full ms [60.0000-900.0000]
311.1844
R=62681
z=1
137.0595
R=90804
z=1
111.1170
R=98255
z=1
183.0649
R=80250
z=1
209.0805
R=72900
z=1
293.1741
R=65695
z=1
333.1664
R=59709
z=1
381.2368
R=53767
z=1
410.2892
R=55189
z=1
485.2469
R=47017
z=2
515.4416
R=47898
z=1
585.8314
R=40111
z=?
621.3622
R=44423
z=1
674.2740
R=41056
z=1
703.5733
R=43589
z=1
745.4278
R=37621
z=1
792.9834
R=38226
z=?
857.3338
R=38120
z=?
889.6197
R=39601
z=?
Relative Abundance
m/z

FTIR

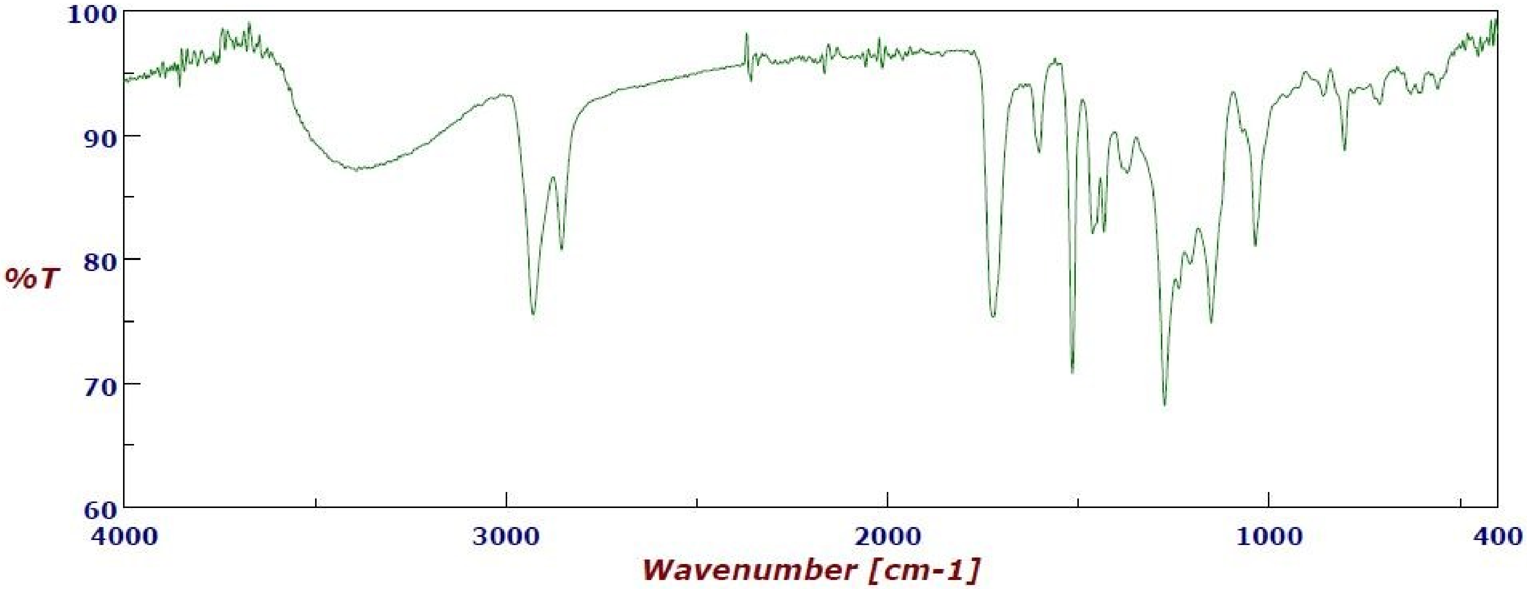
%T
Wavenumber [cm-1]

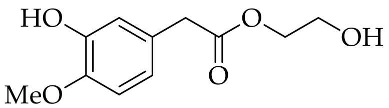

2-Hydroxyethyl 2-(3-hydroxy-4-methoxyphenyl)acetate **22**

^{1}H NMR

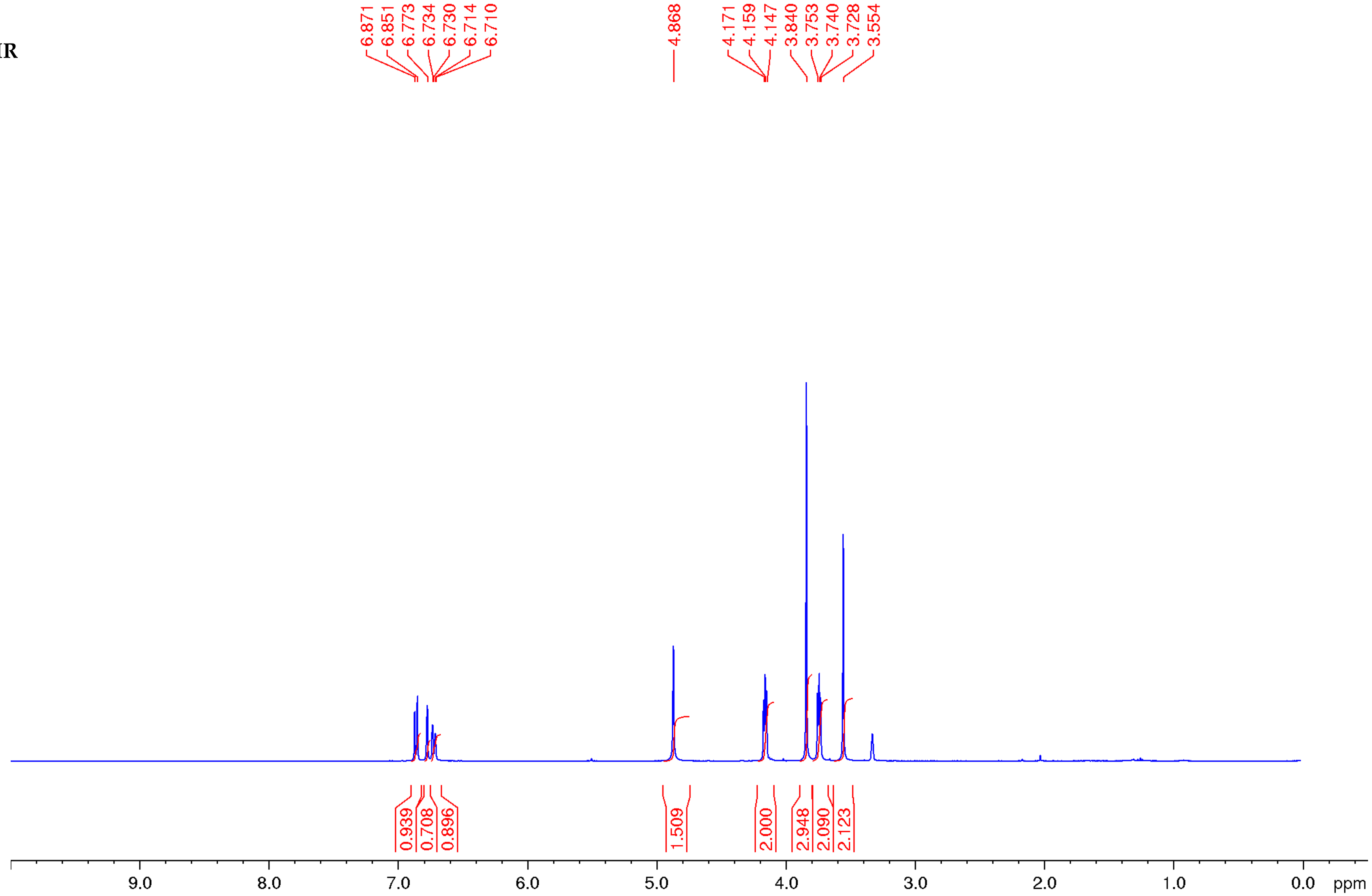

^{13}C NMR

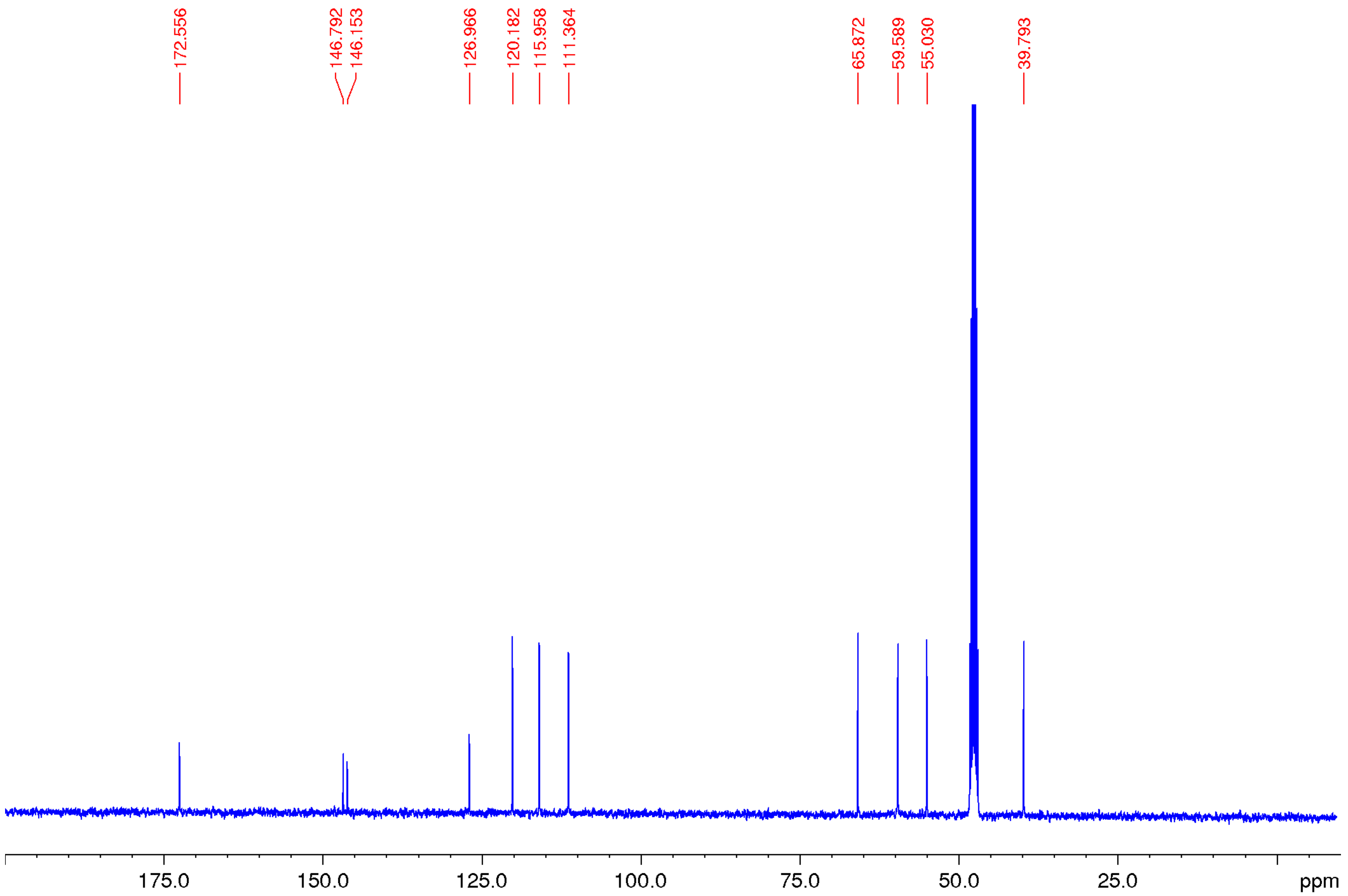
172.556
146.792
146.153
126.966
120.182
115.958
111.364
65.872
59.589
55.030
39.793
175.0
150.0
125.0
100.0
75.0
50.0
25.0
ppm

HRMS

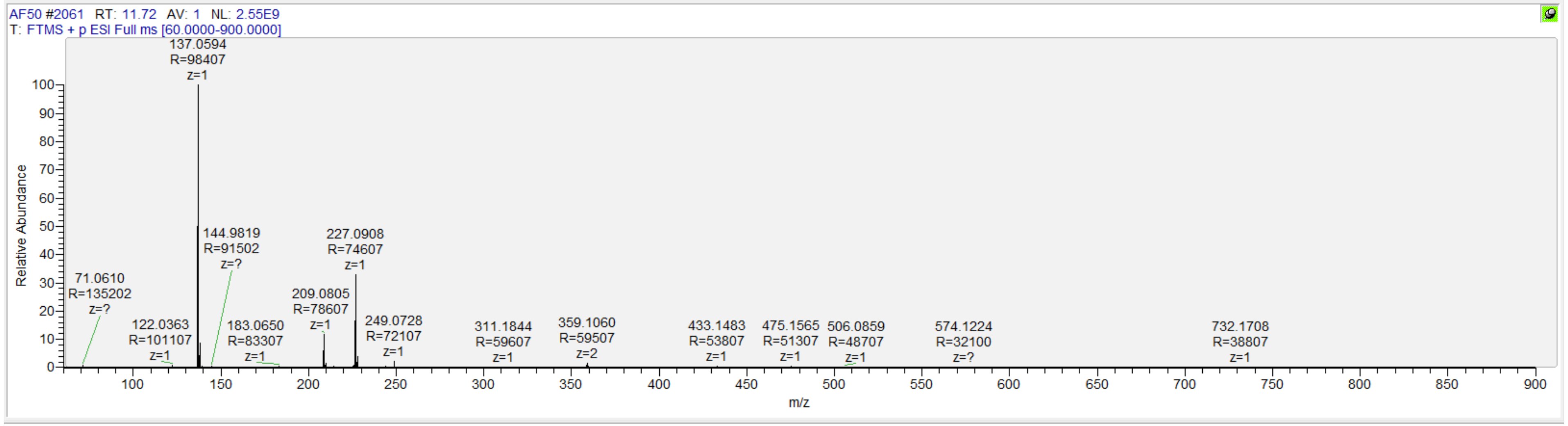
AF50 #2061 RT: 11.72 AV: 1 NL: 2.55E9
T: FTMS + p ESI Full ms [60.0000-900.0000]
137.0594
R=98407
z=1
144.9819
R=91502
z=?
227.0908
R=74607
z=1
71.0610
R=135202
z=?
209.0805
R=78607
z=1
122.0363
R=101107
z=1
183.0650
R=83307
z=1
249.0728
R=72107
z=1
311.1844
R=59607
z=1
359.1060
R=59507
z=2
433.1483
R=53807
z=1
475.1565
R=51307
z=1
506.0859
R=48707
z=1
574.1224
R=32100
z=?
732.1708
R=38807
z=1
Relative Abundance
m/z

FTIR

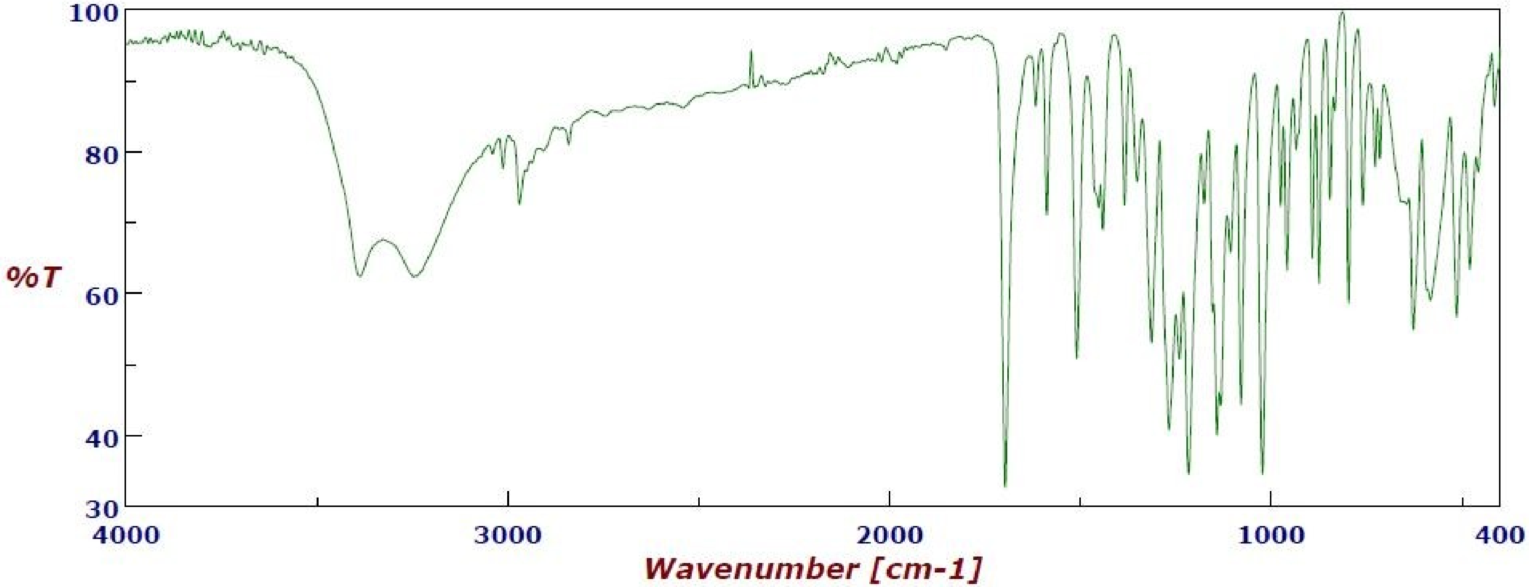
%T
Wavenumber [cm-1]

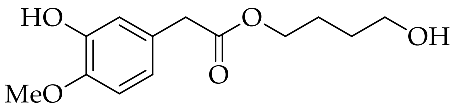

4-Hydroxybutyl 2-(3-hydroxy-4-methoxyphenyl)acetate **23**

^{1}H NMR

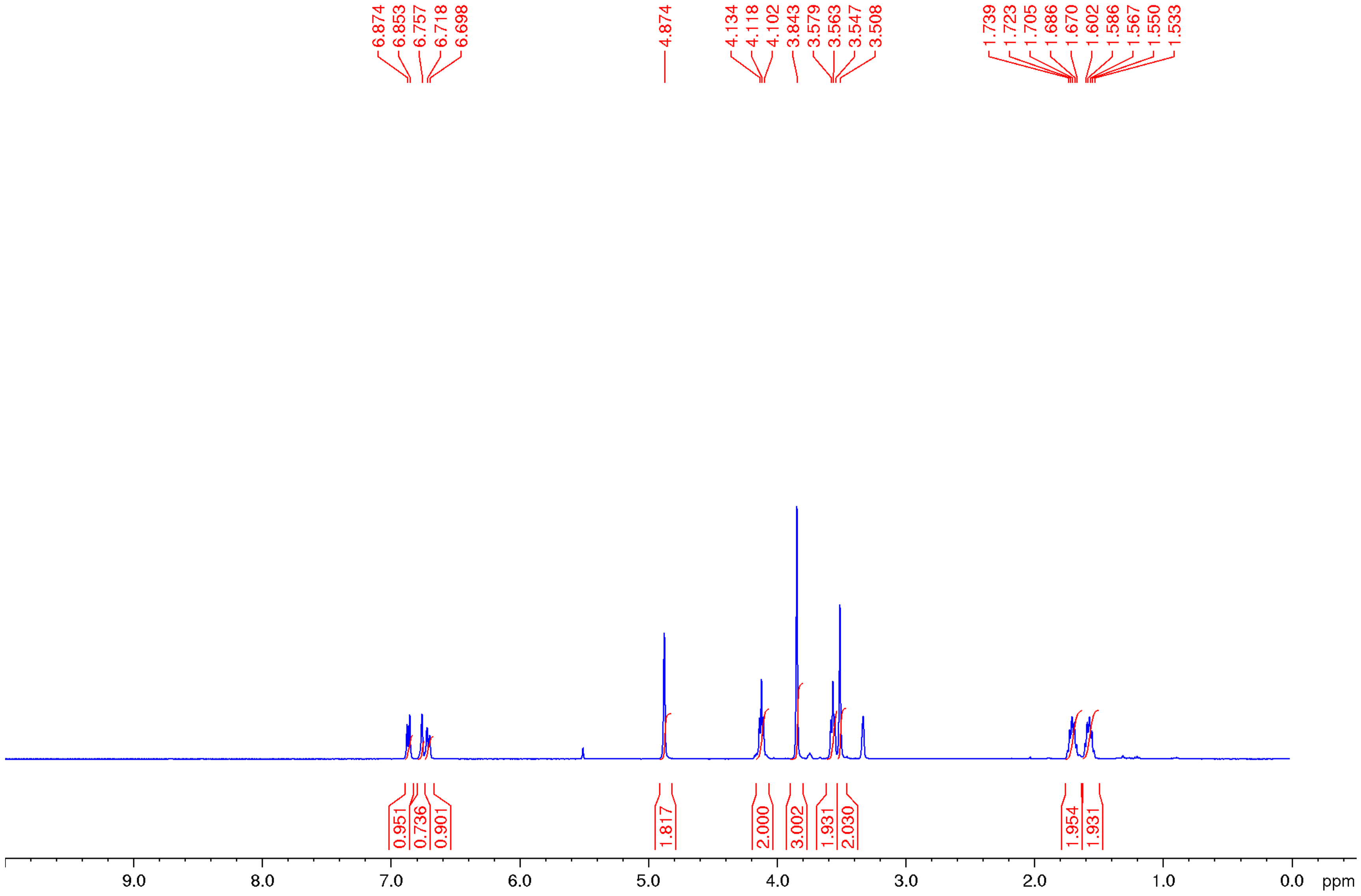

^{13}C NMR

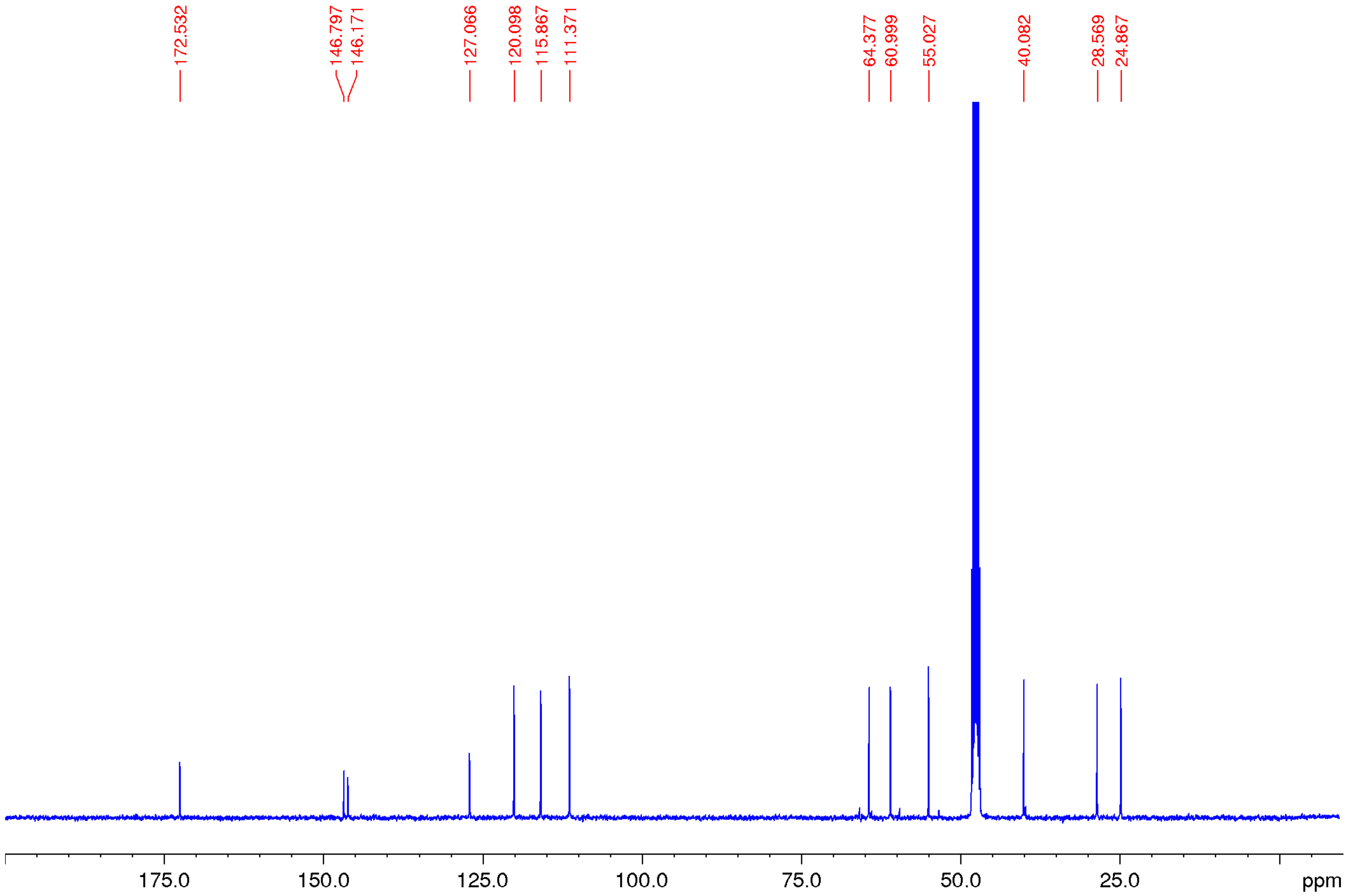
172.532
146.797
146.171
127.066
120.098
115.867
111.371
64.377
60.999
55.027
40.082
28.569
24.867
175.0
150.0
125.0
100.0
75.0
50.0
25.0
ppm

HRMS

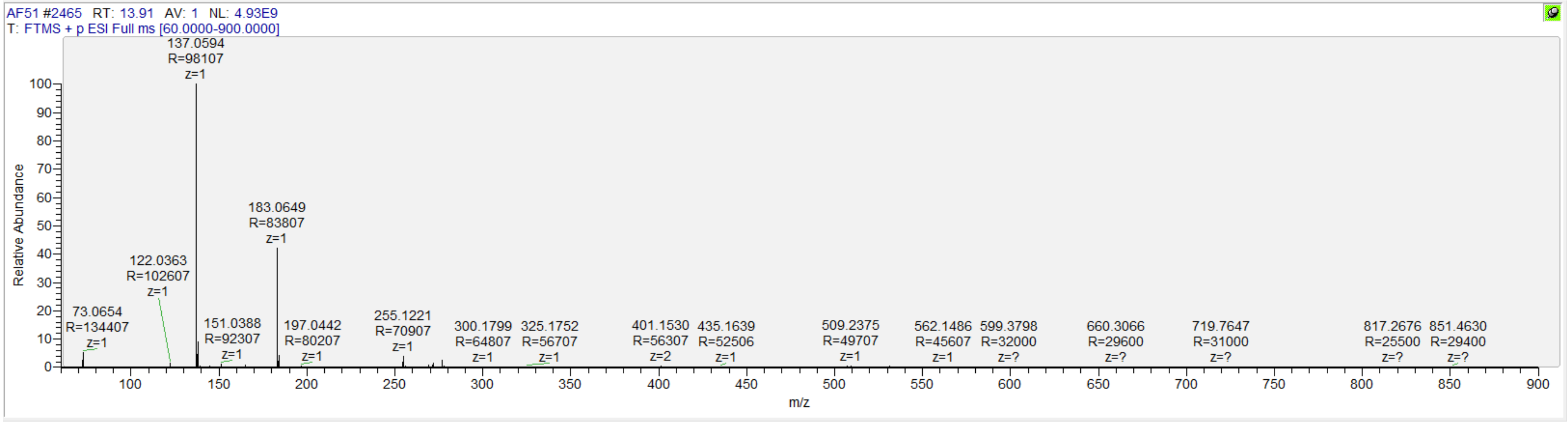
AF51 #2465 RT: 13.91 AV: 1 NL: 4.93E9
T: FTMS + p ESI Full ms [60.0000-900.0000]
137.0594
R=98107
z=1
183.0649
R=83807
z=1
122.0363
R=102607
z=1
73.0654
R=134407
z=1
151.0388
R=92307
z=1
197.0442
R=80207
z=1
255.1221
R=70907
z=1
300.1799
R=64807
z=1
325.1752
R=56707
z=1
401.1530
R=56307
z=2
435.1639
R=52506
z=1
509.2375
R=49707
z=1
562.1486
R=45607
z=1
599.3798
R=32000
z=?
660.3066
R=29600
z=?
719.7647
R=31000
z=?
817.2676
R=25500
z=?
851.4630
R=29400
z=?
Relative Abundance
m/z

FTIR

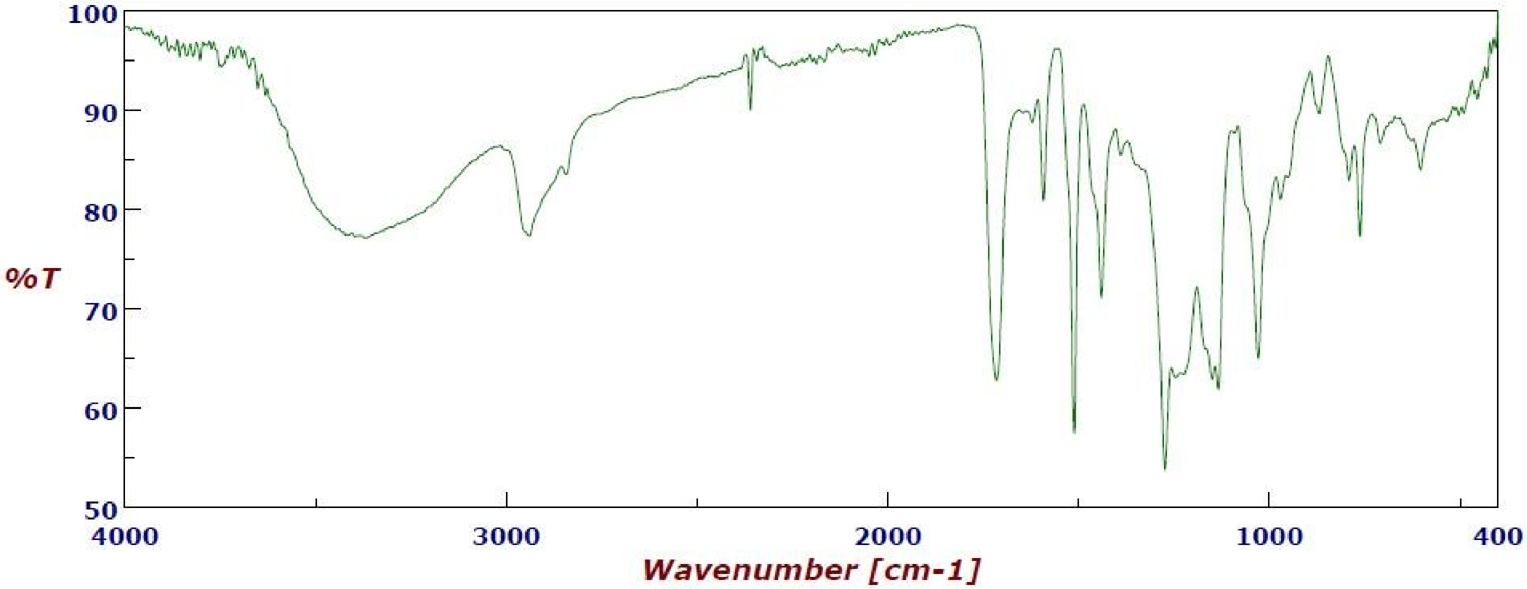
%T
Wavenumber [cm-1]

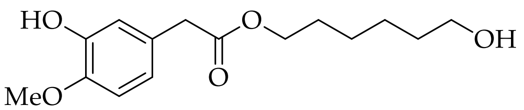

6-Hydroxyhexyl 2-(3-hydroxy-4-methoxyphenyl)acetate **24**

^{1}H NMR

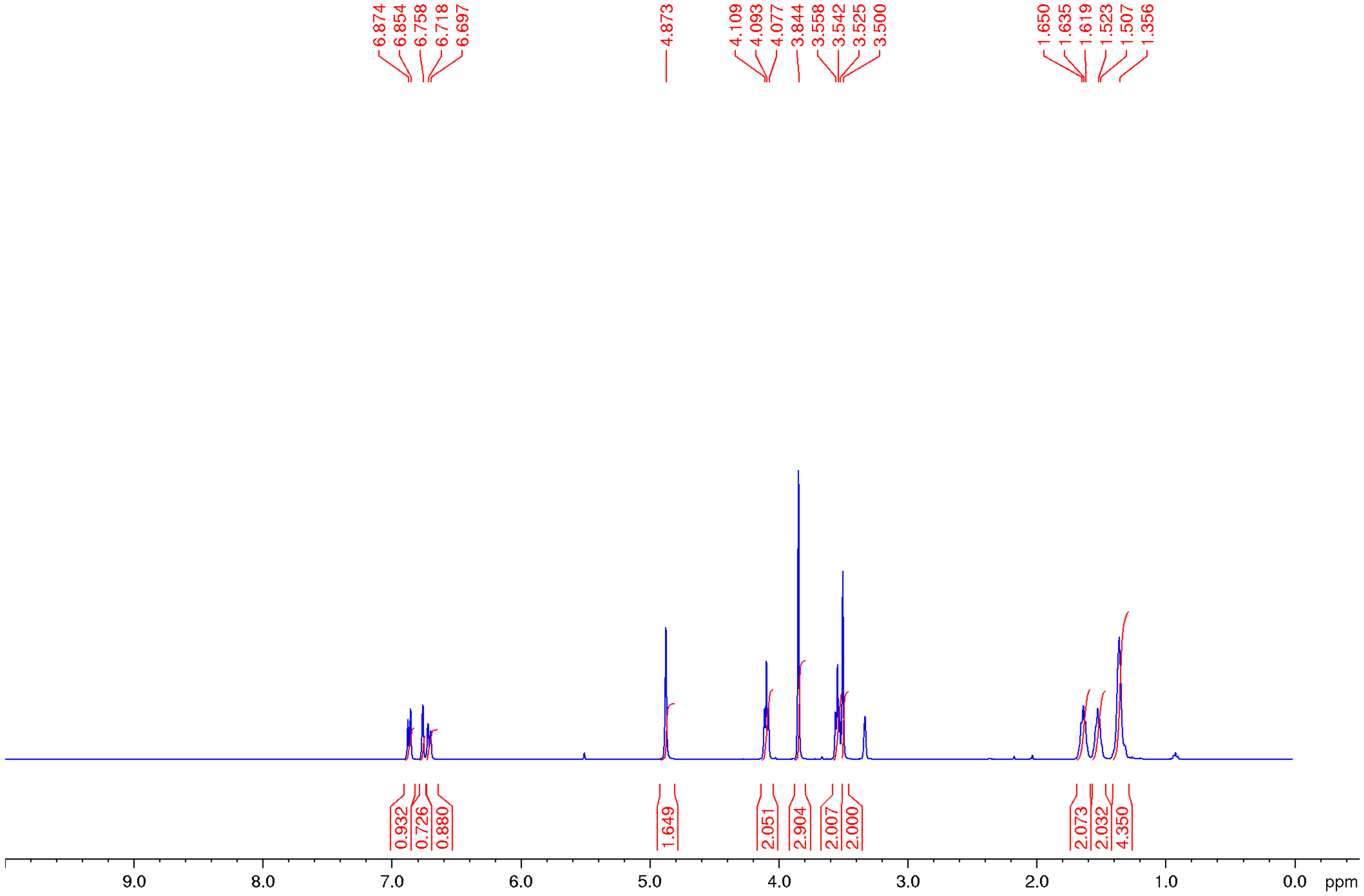

^{13}C NMR

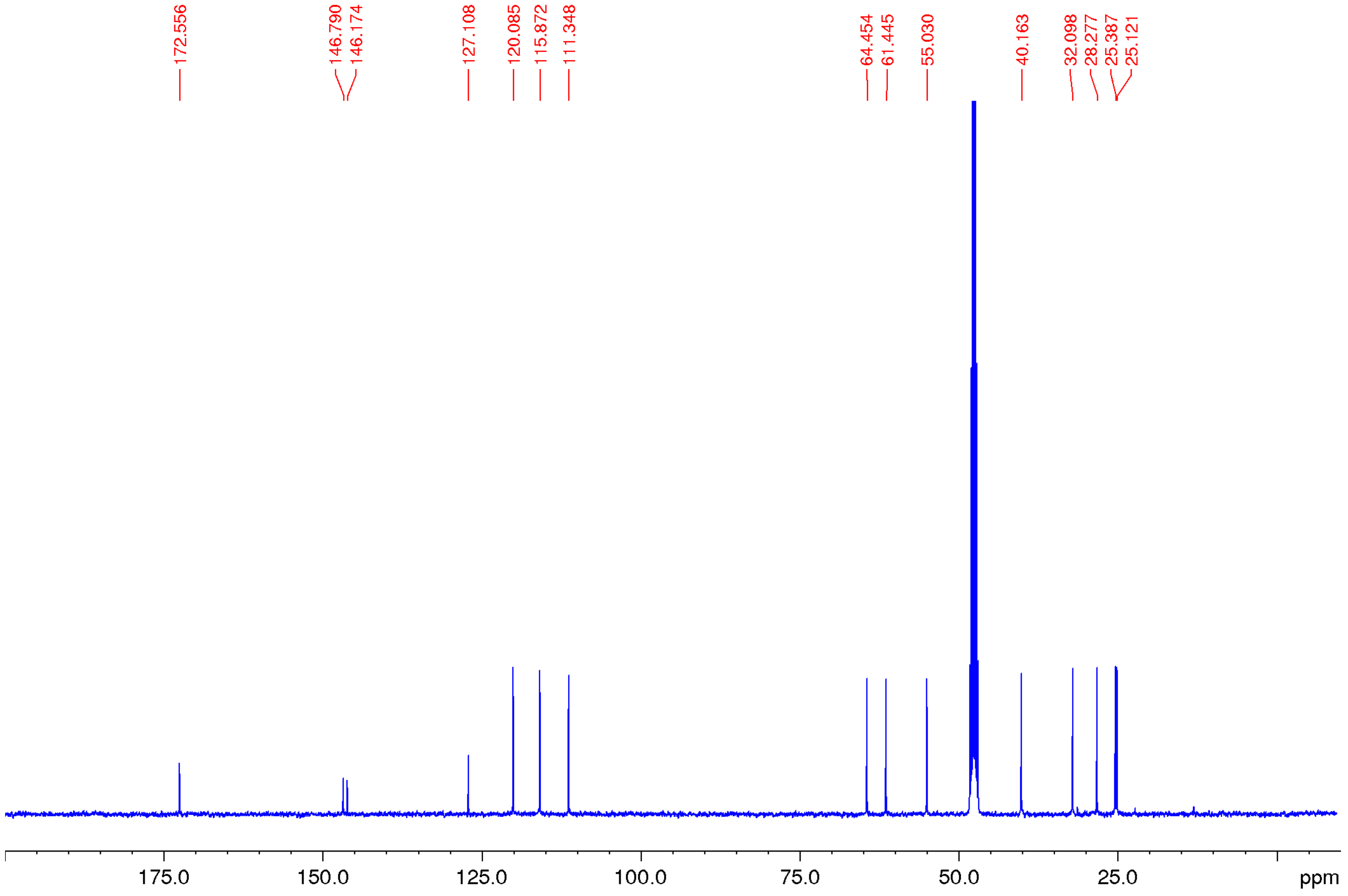

172.556
146.790
146.174
127.108
120.085
115.872
111.348
64.454
61.445
55.030
40.163
32.098
28.277
25.387
25.121
175.0
150.0
125.0
100.0
75.0
50.0
25.0
ppm

HRMS

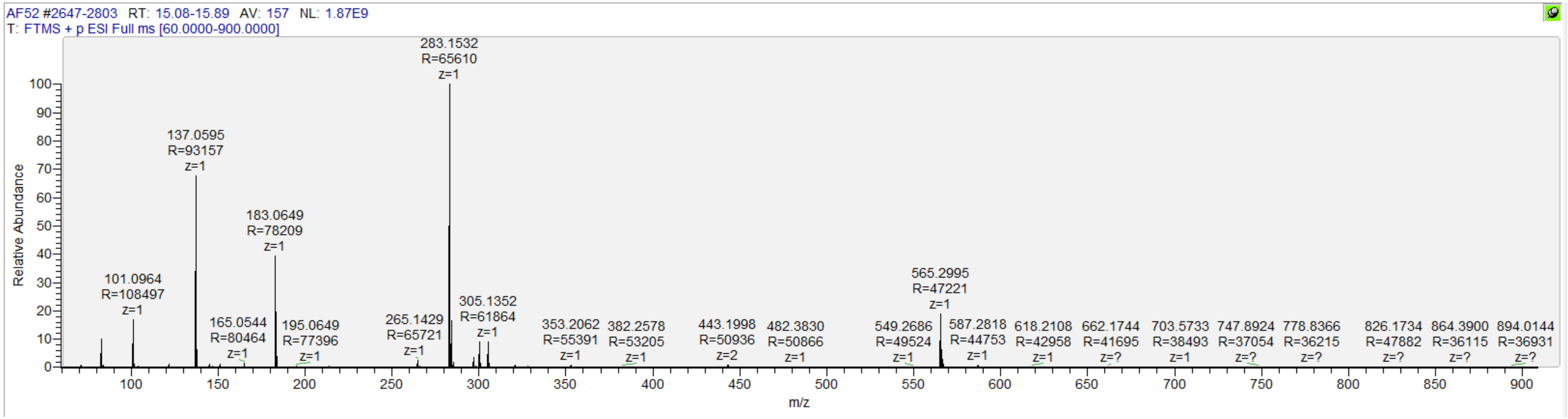

AF52 #2647-2803 RT: 15.08-15.89 AV: 157 NL: 1.87E9
T: FTMS + p ESI Full ms [60.0000-900.0000]
283.1532 R=65610 z=1
137.0595 R=93157 z=1
183.0649 R=78209 z=1
101.0964 R=108497 z=1
165.0544 R=80464 z=1
195.0649 R=77396 z=1
265.1429 R=65721 z=1
305.1352 R=61864 z=1
353.2062 R=55391 z=1
382.2578 R=53205 z=1
443.1998 R=50936 z=2
482.3830 R=50866 z=1
549.2686 R=49524 z=1
565.2995 R=47221 z=1
587.2818 R=44753 z=1
618.2108 R=42958 z=1
662.1744 R=41695 z=?
703.5733 R=38493 z=1
747.8924 R=37054 z=?
778.8366 R=36215 z=?
826.1734 R=47882 z=?
864.3900 R=36115 z=?
894.0144 R=36931 z=?
Relative Abundance
m/z

FTIR

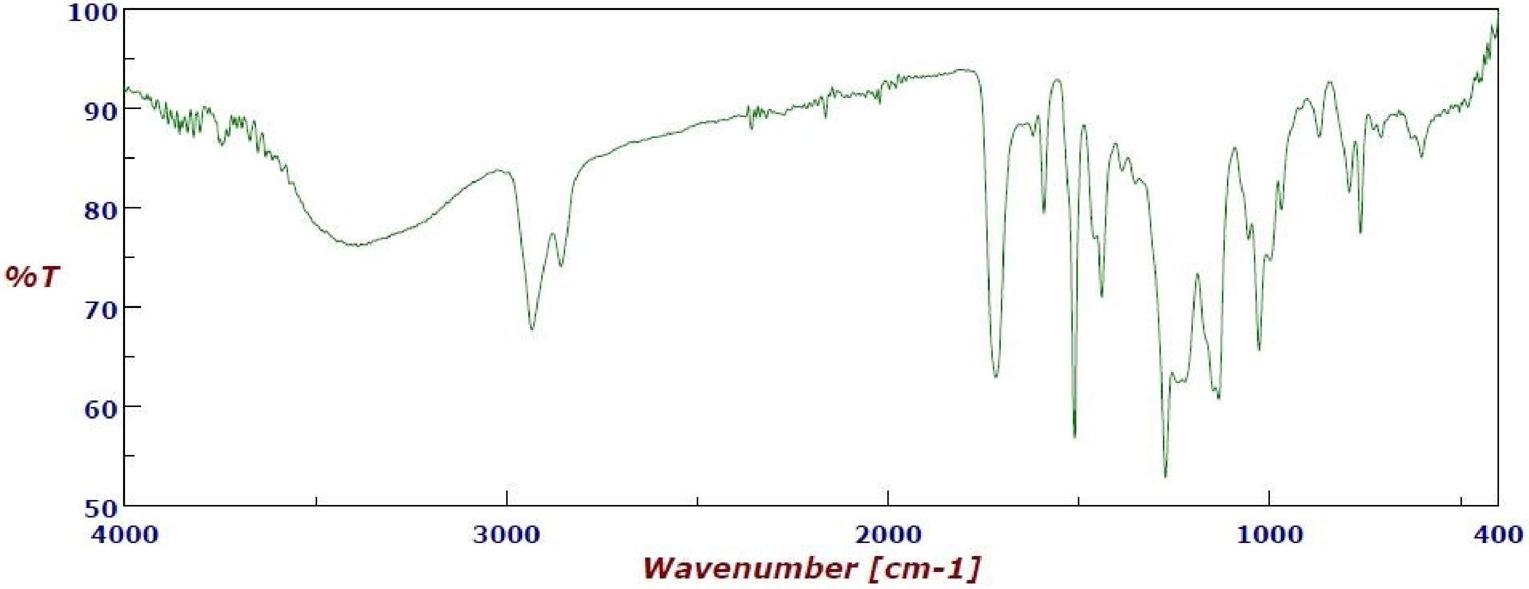

%T
Wavenumber [cm-1]

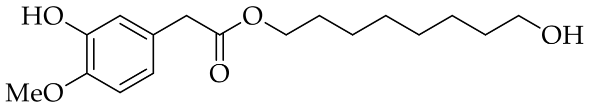

8-Hydroxyoctyl 2-(3-hydroxy-4-methoxyphenyl)acetate **25**

^{1}H NMR

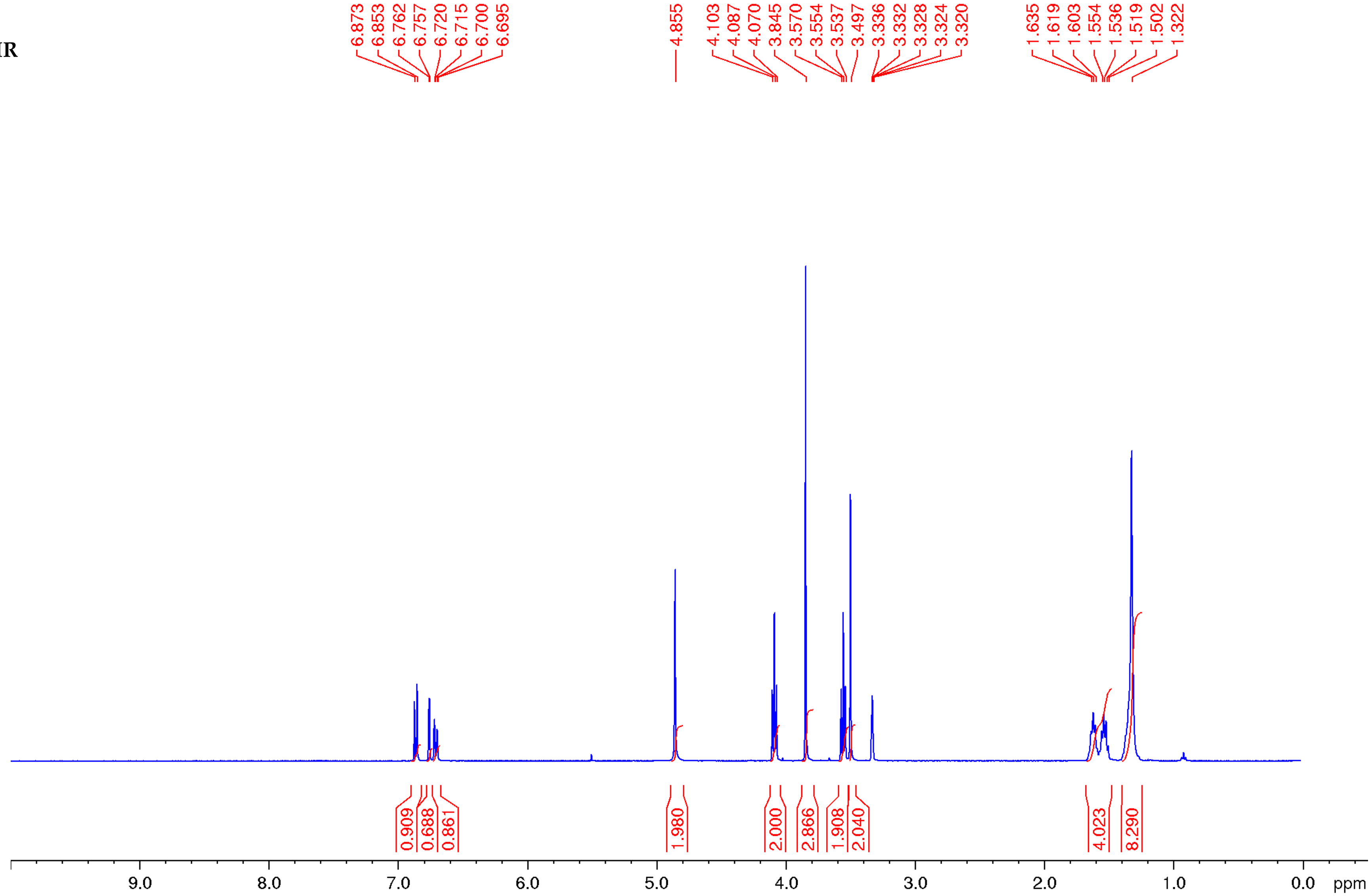

^{13}C NMR

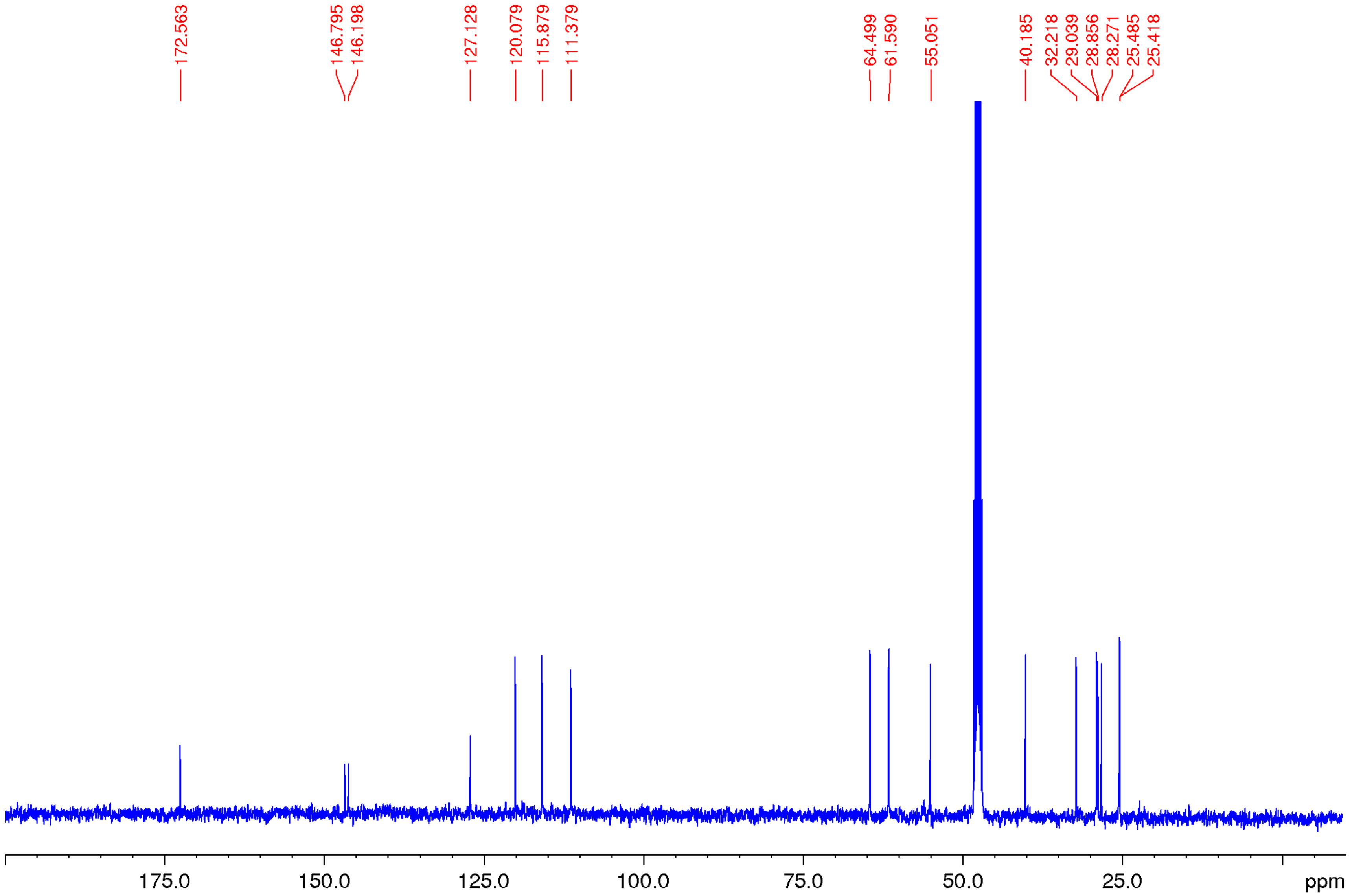

172.563
146.795
146.198
127.128
120.079
115.879
111.379
64.499
61.590
55.051
40.185
32.218
29.039
28.856
28.271
25.485
25.418
175.0
150.0
125.0
100.0
75.0
50.0
25.0
ppm

HRMS

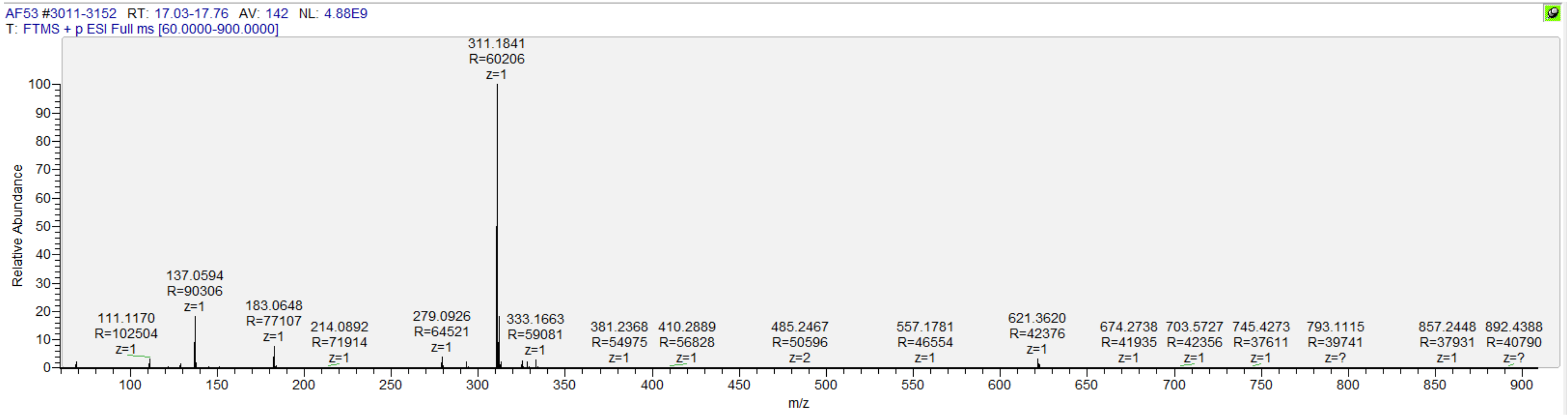
AF53 #3011-3152 RT: 17.03-17.76 AV: 142 NL: 4.88E9
T: FTMS + p ESI Full ms [60.0000-900.0000]
311.1841 R=60206 z=1
137.0594 R=90306 z=1
111.1170 R=102504 z=1
183.0648 R=77107 z=1
214.0892 R=71914 z=1
279.0926 R=64521 z=1
333.1663 R=59081 z=1
381.2368 R=54975 z=1
410.2889 R=56828 z=1
485.2467 R=50596 z=2
557.1781 R=46554 z=1
621.3620 R=42376 z=1
674.2738 R=41935 z=1
703.5727 R=42356 z=1
745.4273 R=37611 z=1
793.1115 R=39741 z=?
857.2448 R=37931 z=1
892.4388 R=40790 z=?
Relative Abundance
m/z

FTIR

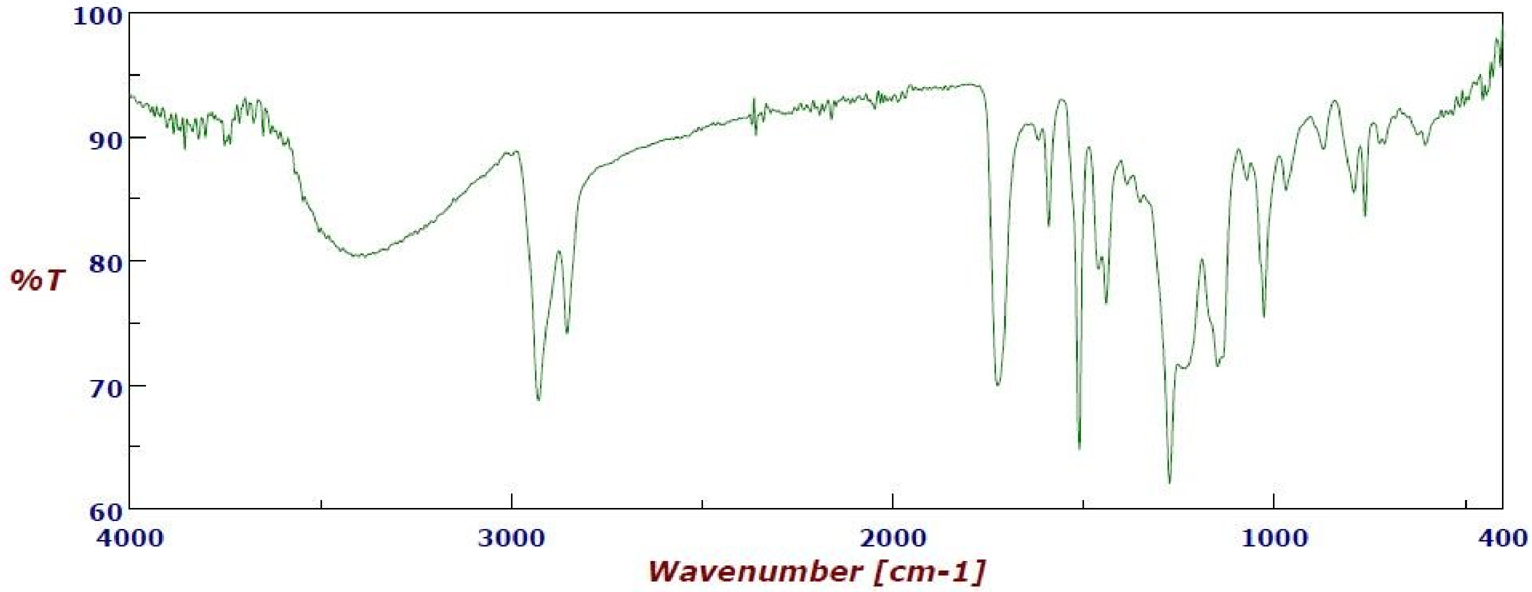
%T
Wavenumber [cm-1]

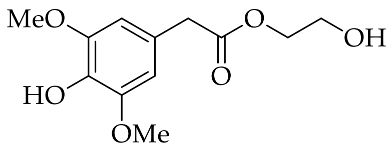

2-Hydroxyethyl 2-(4-hydroxy-3,5-dimethoxyphenyl)acetate **26**

^{1}H NMR

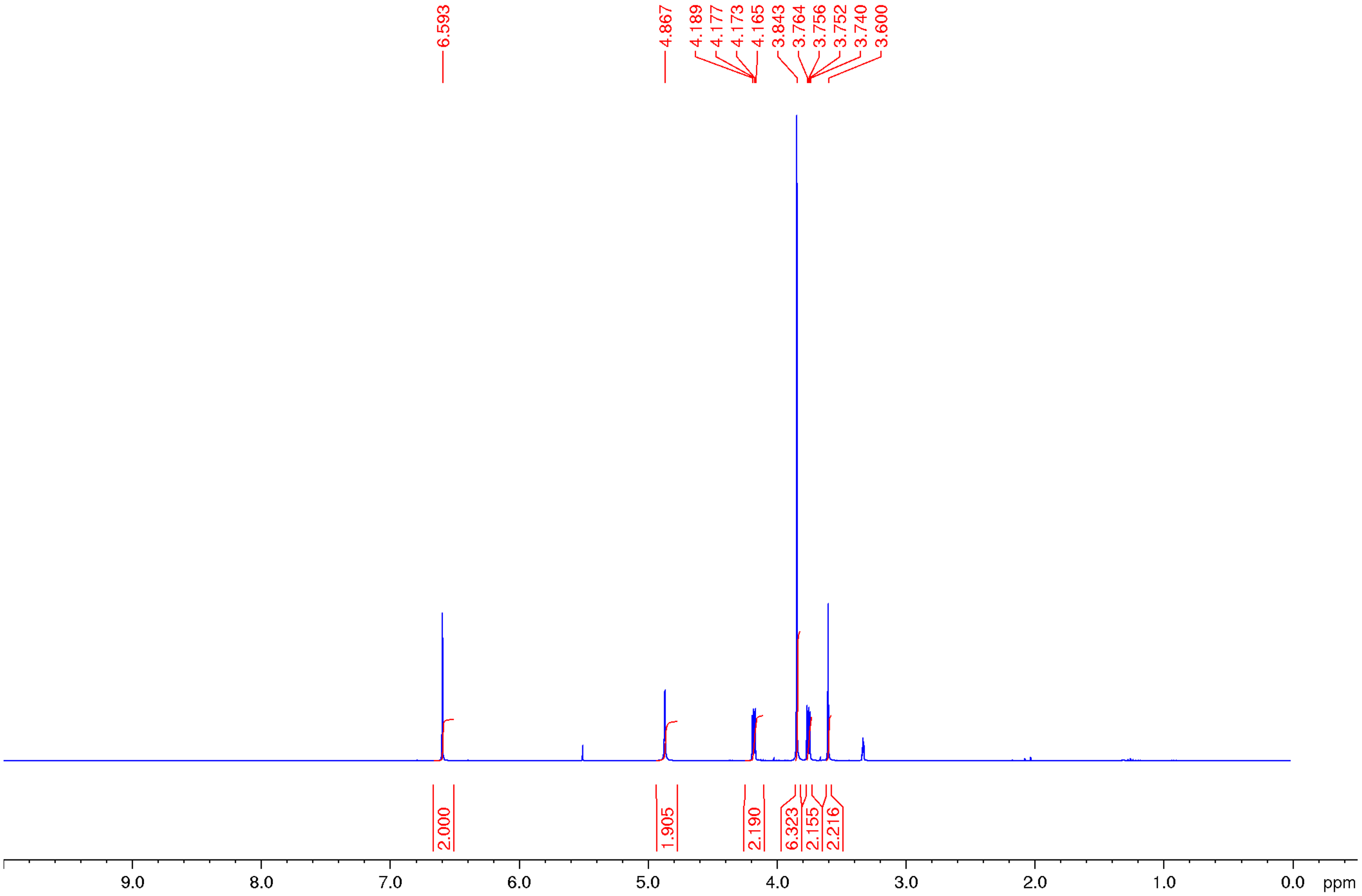

^{13}C NMR

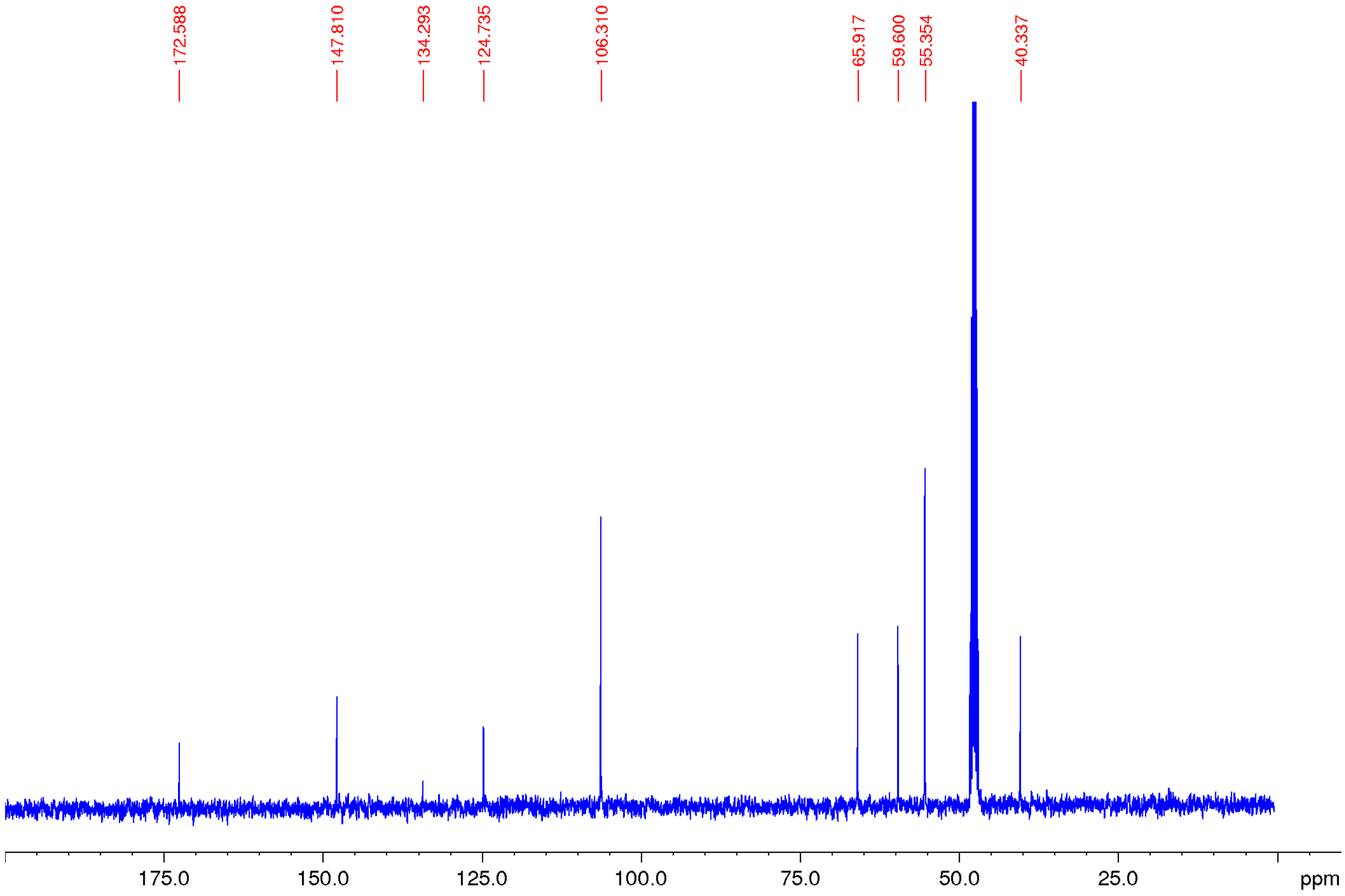

172.588
147.810
134.293
124.735
106.310
65.917
59.600
55.354
40.337
175.0
150.0
125.0
100.0
75.0
50.0
25.0
ppm

HRMS

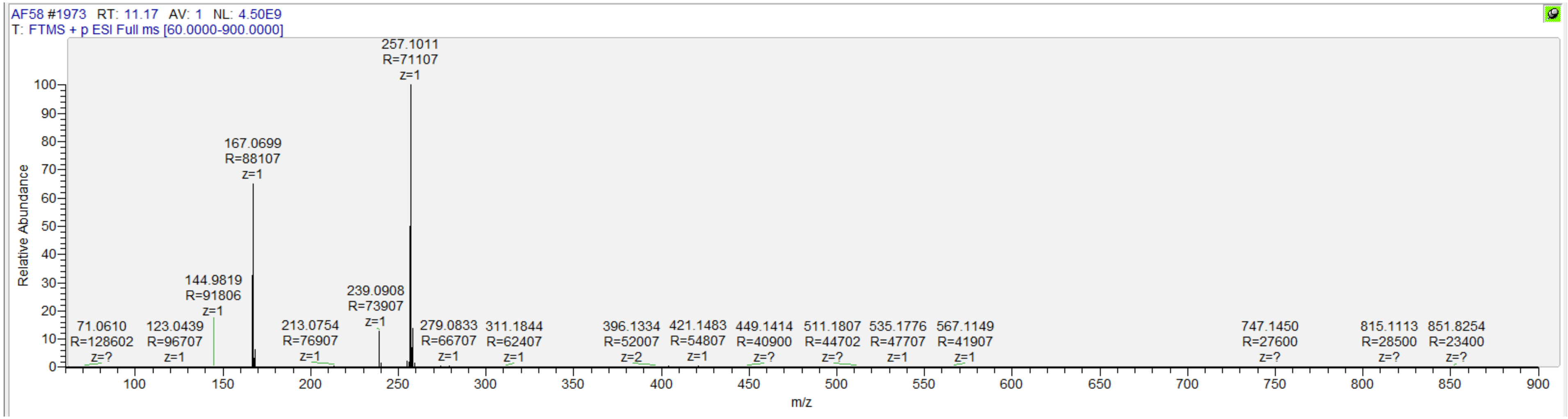

AF58 #1973 RT: 11.17 AV: 1 NL: 4.50E9
T: FTMS + p ESI Full ms [60.0000-900.0000]
257.1011 R=71107 z=1
167.0699 R=88107 z=1
144.9819 R=91806 z=1
239.0908 R=73907 z=1
71.0610 R=128602 z=?
123.0439 R=96707 z=1
213.0754 R=76907 z=1
279.0833 R=66707 z=1
311.1844 R=62407 z=1
396.1334 R=52007 z=2
421.1483 R=54807 z=1
449.1414 R=40900 z=?
511.1807 R=44702 z=?
535.1776 R=47707 z=1
567.1149 R=41907 z=1
747.1450 R=27600 z=?
815.1113 R=28500 z=?
851.8254 R=23400 z=?
Relative Abundance
m/z

FTIR

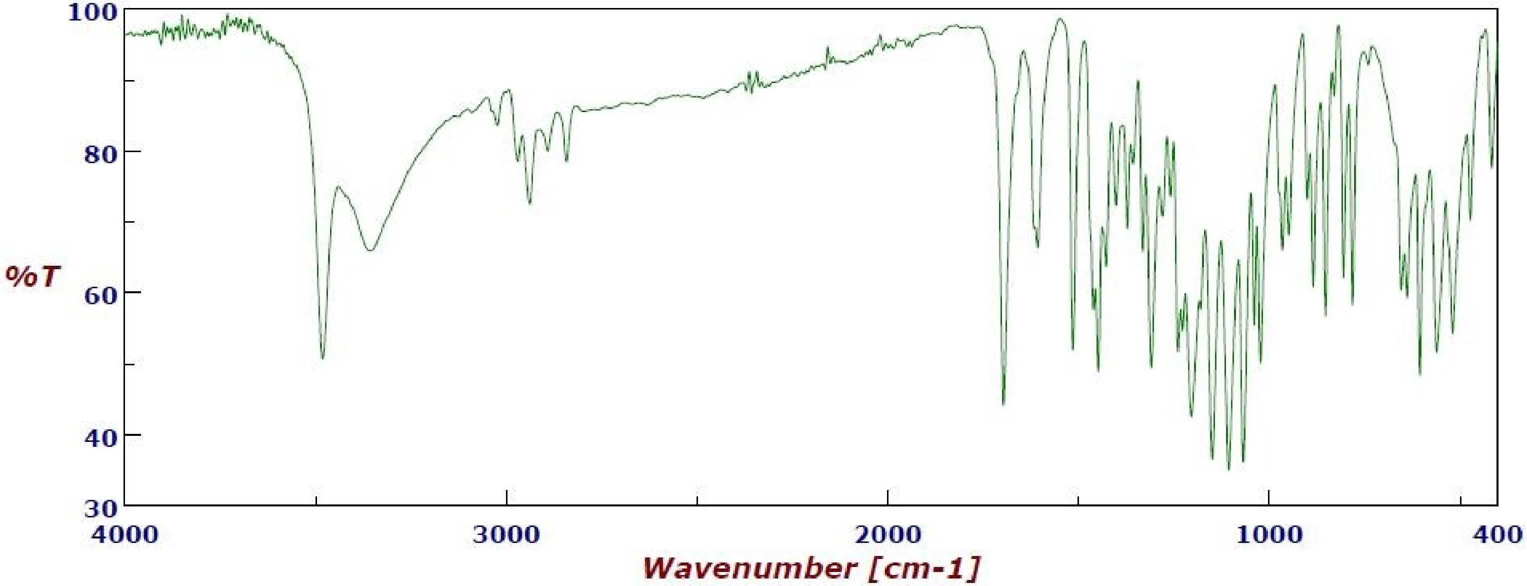

%T
Wavenumber [cm-1]

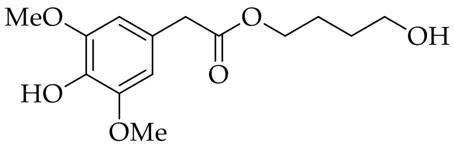

4-Hydroxybutyl 2-(4-hydroxy-3,5-dimethoxyphenyl)acetate **27**

^{1}H NMR

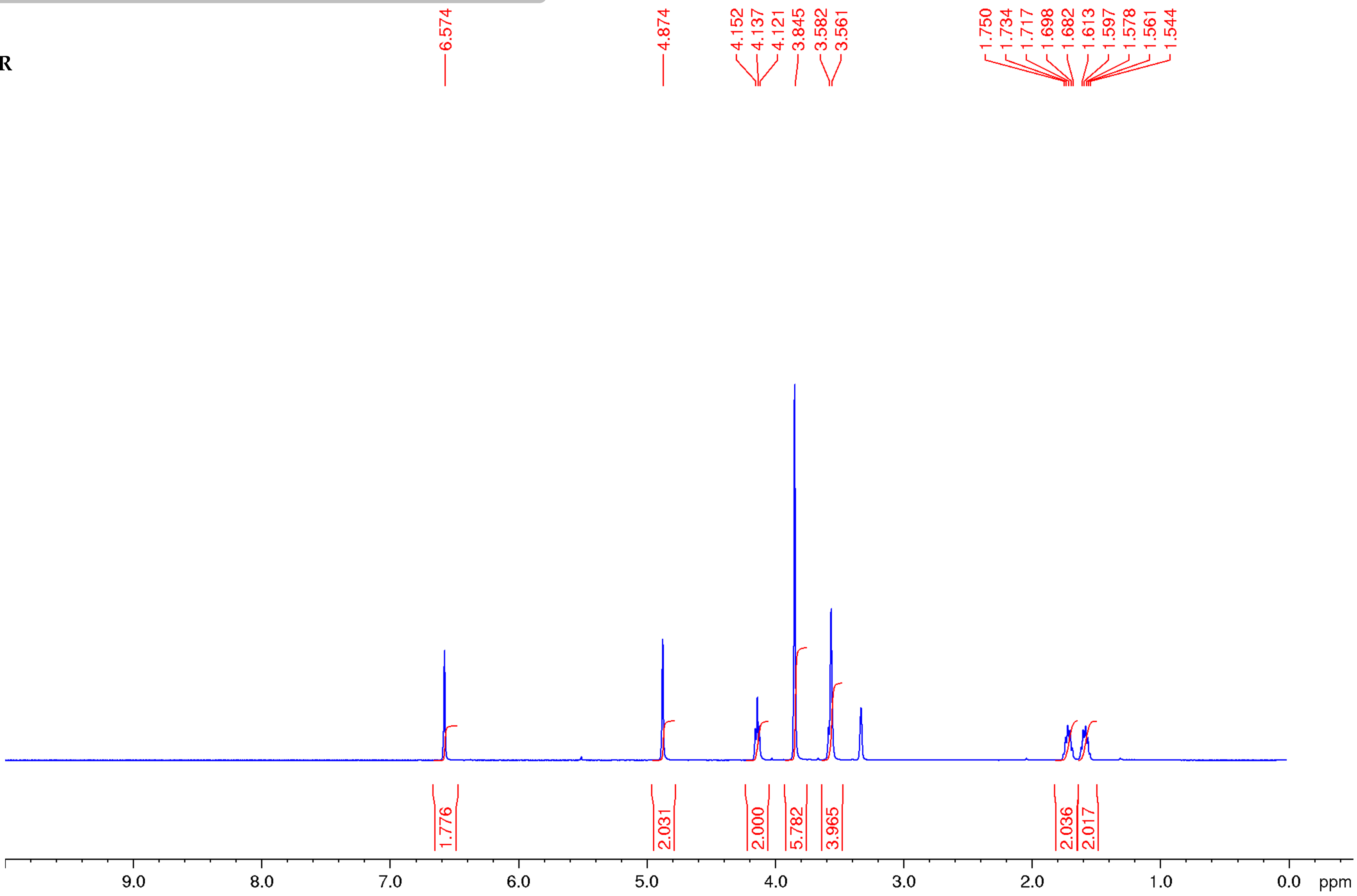

^{13}C NMR

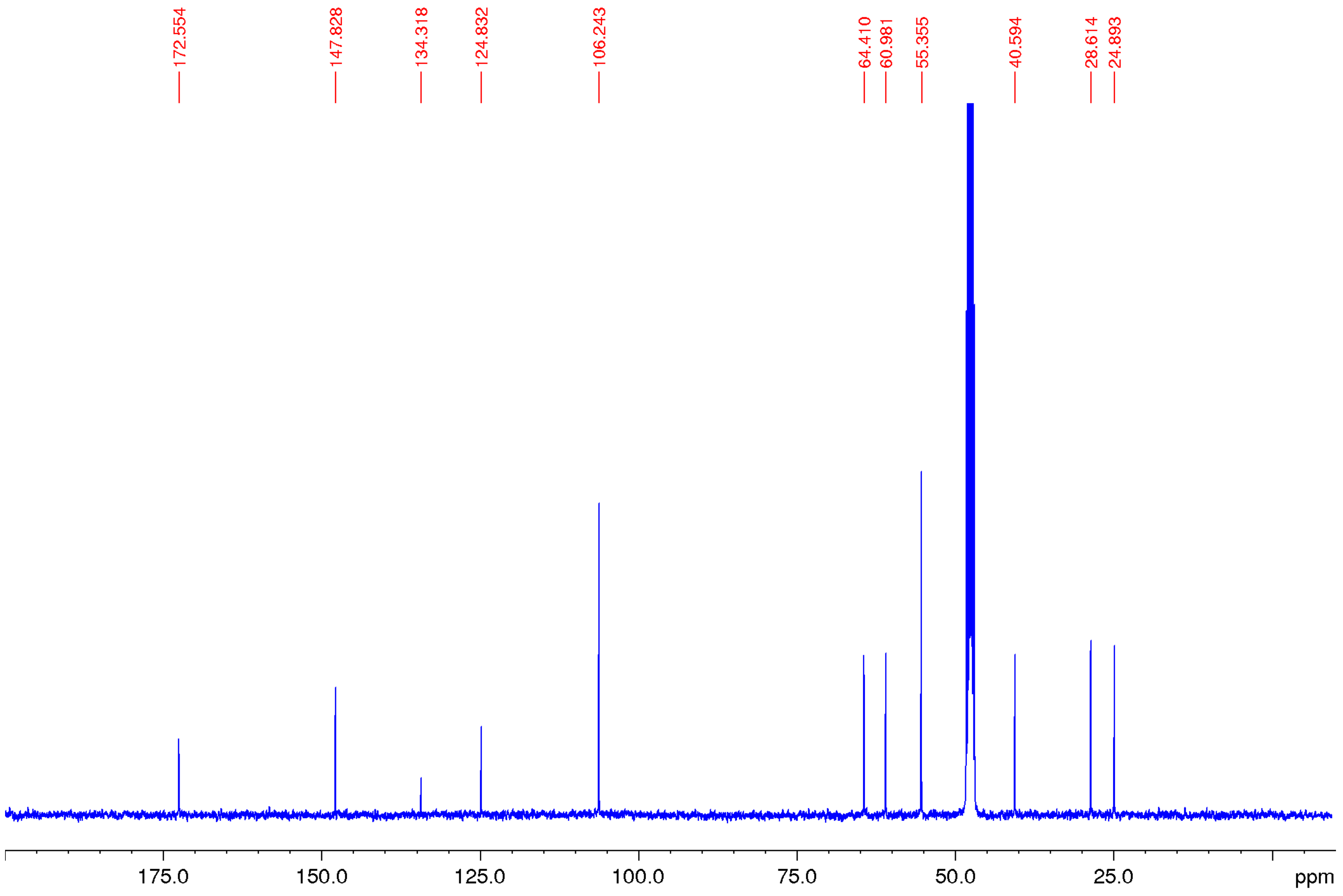

172.554
147.828
134.318
124.832
106.243
64.410
60.981
55.355
40.594
28.614
24.893
175.0
150.0
125.0
100.0
75.0
50.0
25.0
ppm

HRMS

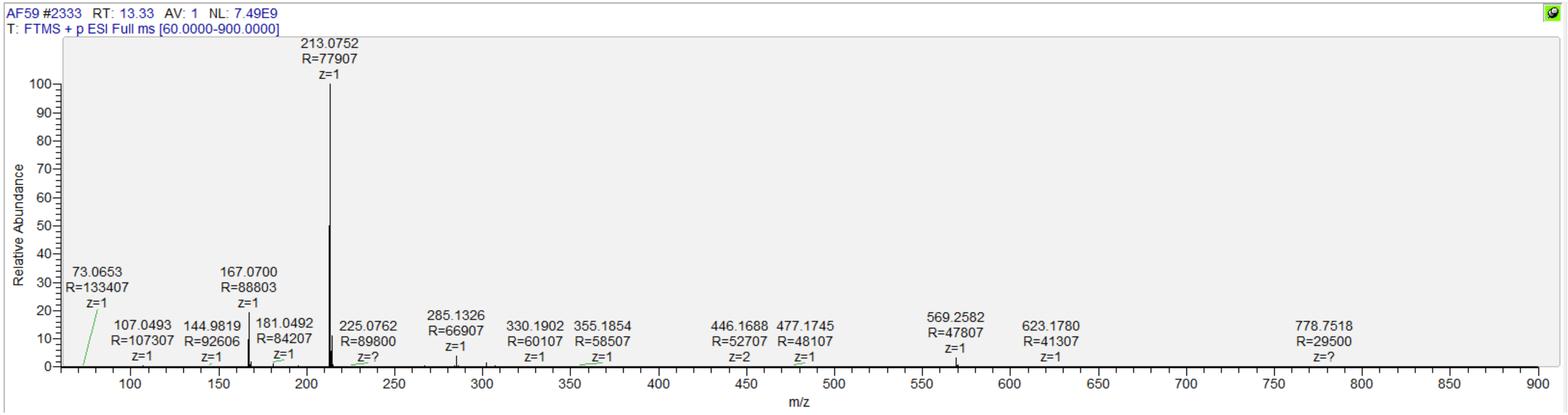

AF59 #2333 RT: 13.33 AV: 1 NL: 7.49E9
T: FTMS + p ESI Full ms [60.0000-900.0000]
213.0752 R=77907 z=1
73.0653 R=133407 z=1
107.0493 R=107307 z=1
144.9819 R=92606 z=1
167.0700 R=88803 z=1
181.0492 R=84207 z=1
225.0762 R=89800 z=?
285.1326 R=66907 z=1
330.1902 R=60107 z=1
355.1854 R=58507 z=1
446.1688 R=52707 z=2
477.1745 R=48107 z=1
569.2582 R=47807 z=1
623.1780 R=41307 z=1
778.7518 R=29500 z=?
Relative Abundance
m/z

FTIR

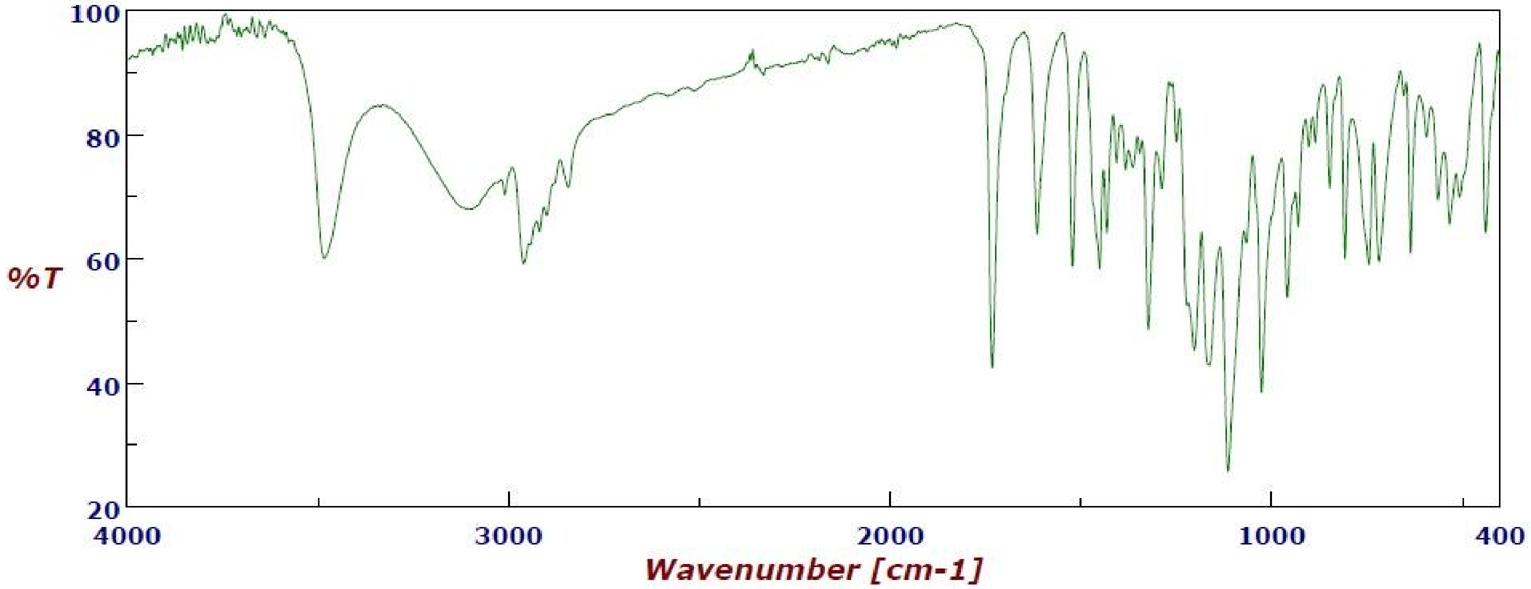

%T
Wavenumber [cm-1]

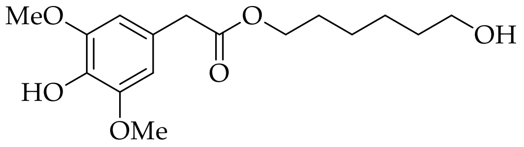

6-Hydroxyhexyl 2-(4-hydroxy-3,5-dimethoxyphenyl)acetate **28**

¹H NMR

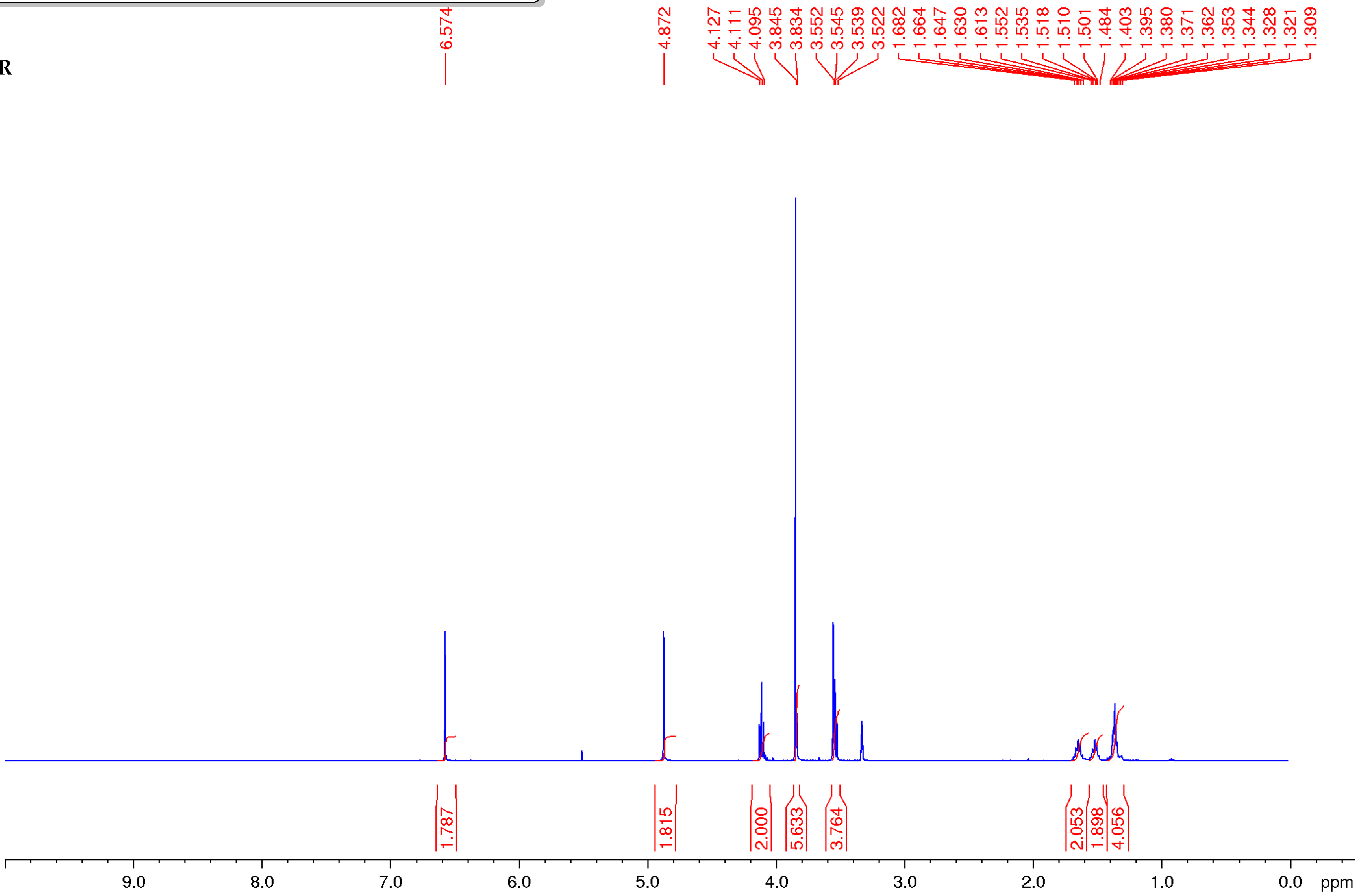

^{13}C NMR

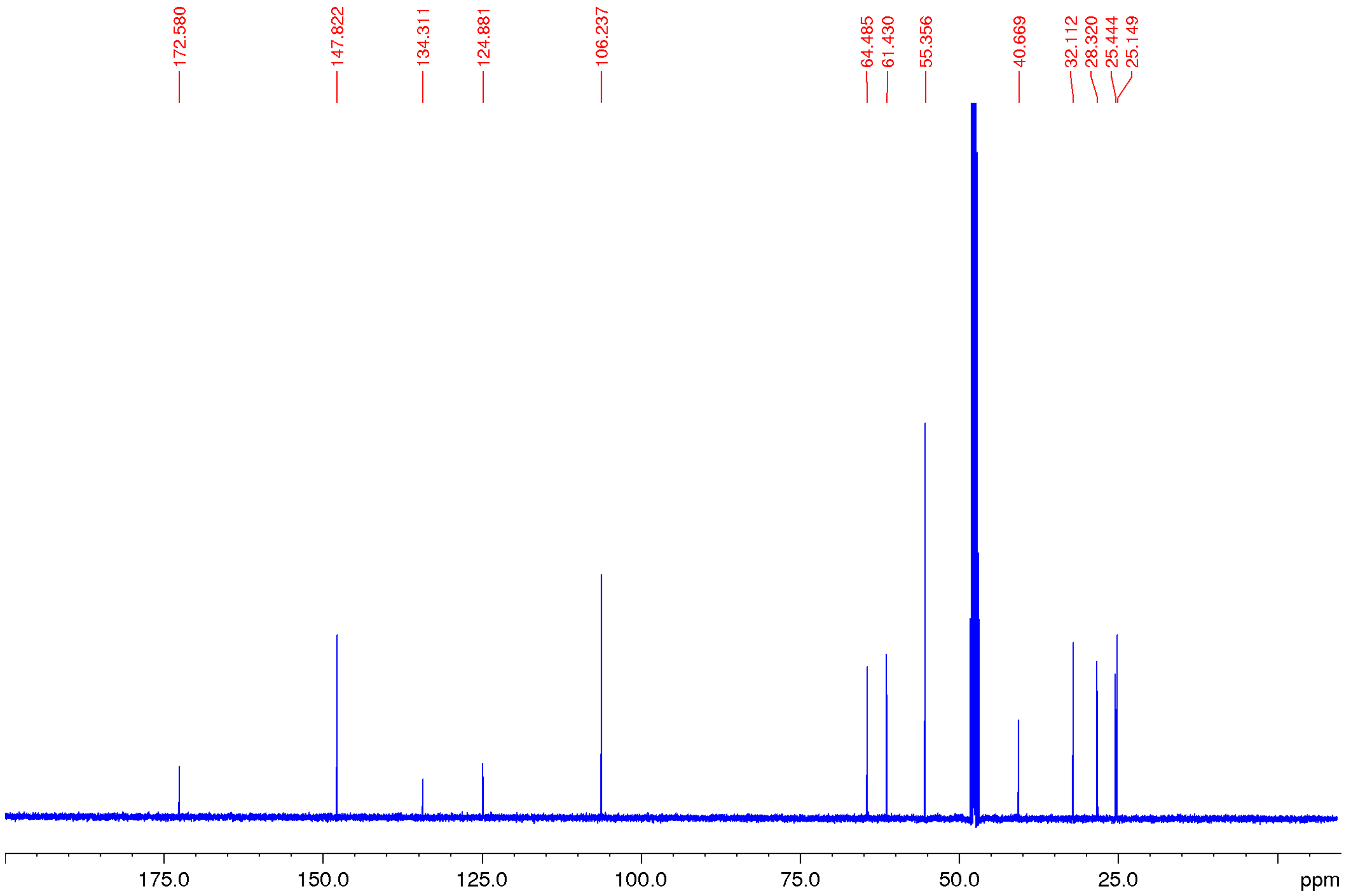

172.580
147.822
134.311
124.881
106.237
64.485
61.430
55.356
40.669
32.112
28.320
25.444
25.149
175.0
150.0
125.0
100.0
75.0
50.0
25.0
ppm

HRMS

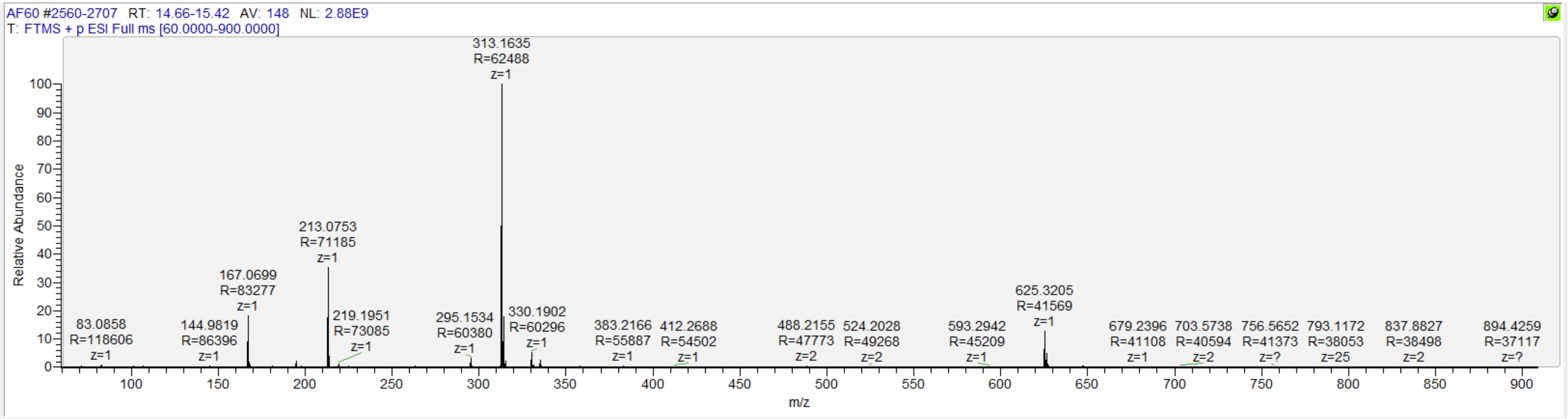

AF60 #2560-2707 RT: 14.66-15.42 AV: 148 NL: 2.88E9
T: FTMS + p ESI Full ms [60.0000-900.0000]
313.1635
R=62488
z=1
213.0753
R=71185
z=1
167.0699
R=83277
z=1
625.3205
R=41569
z=1
83.0858
R=118606
z=1
144.9819
R=86396
z=1
219.1951
R=73085
z=1
295.1534
R=60380
z=1
330.1902
R=60296
z=1
383.2166
R=55887
z=1
412.2688
R=54502
z=1
488.2155
R=47773
z=2
524.2028
R=49268
z=2
593.2942
R=45209
z=1
679.2396
R=41108
z=1
703.5738
R=40594
z=2
756.5652
R=41373
z=?
793.1172
R=38053
z=25
837.8827
R=38498
z=2
894.4259
R=37117
z=?
Relative Abundance
m/z

FTIR

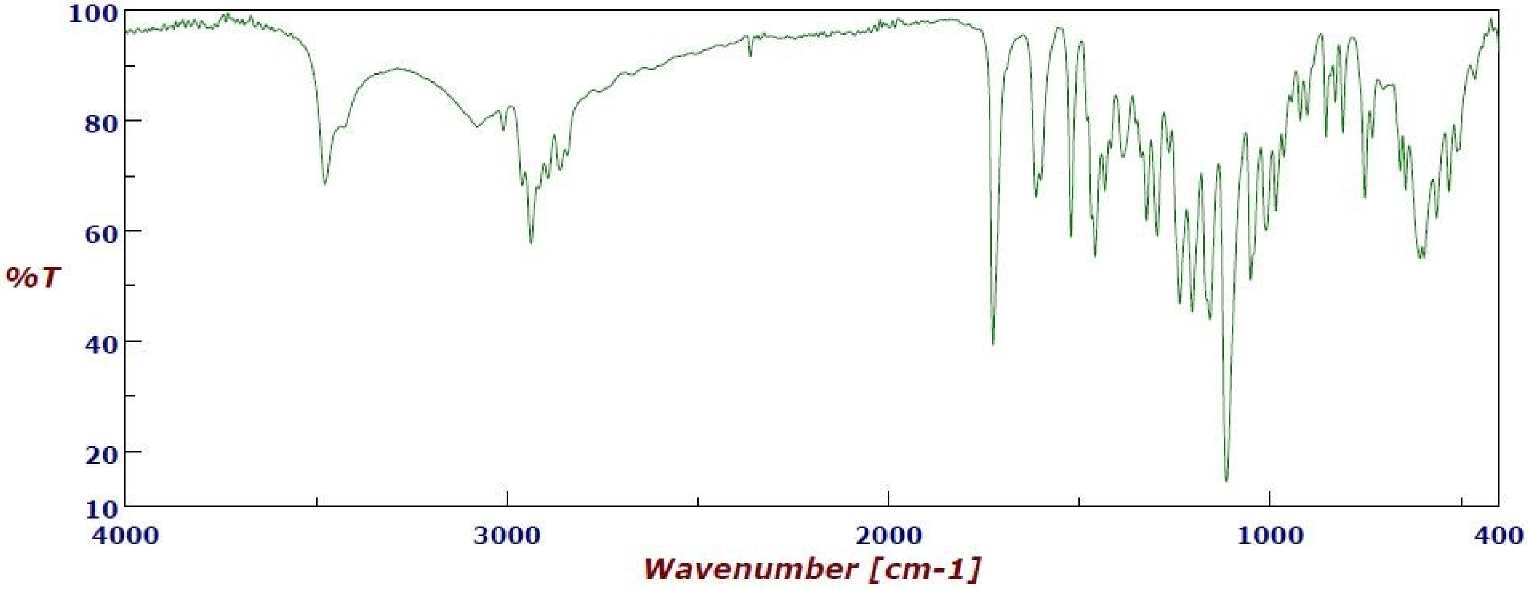

%T
Wavenumber [cm-1]

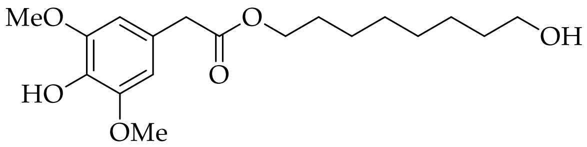

8-Hydroxyoctyl 2-(4-hydroxy-3,5-dimethoxyphenyl)acetate **29**

^{1}H NMR

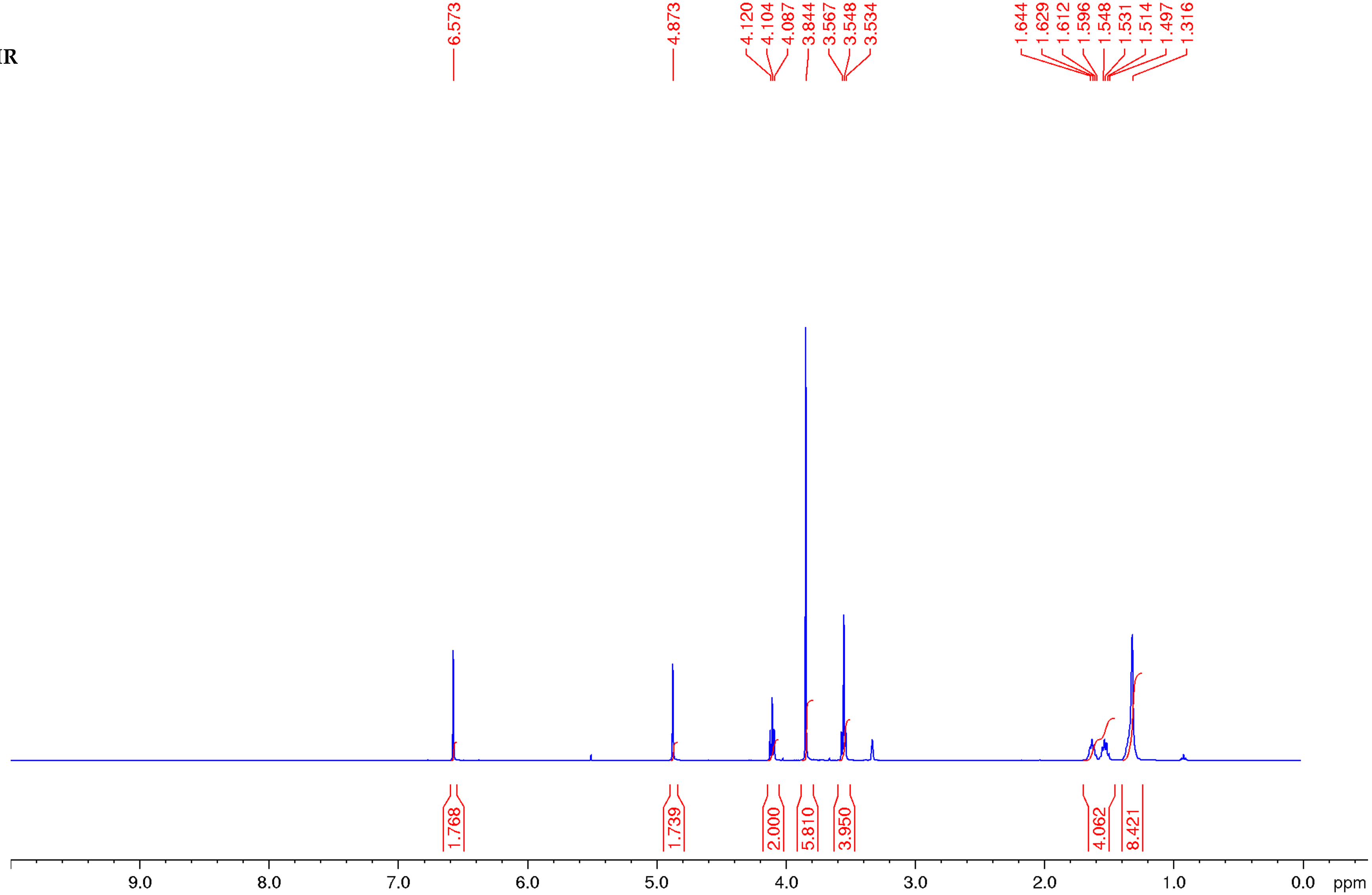

^{13}C NMR

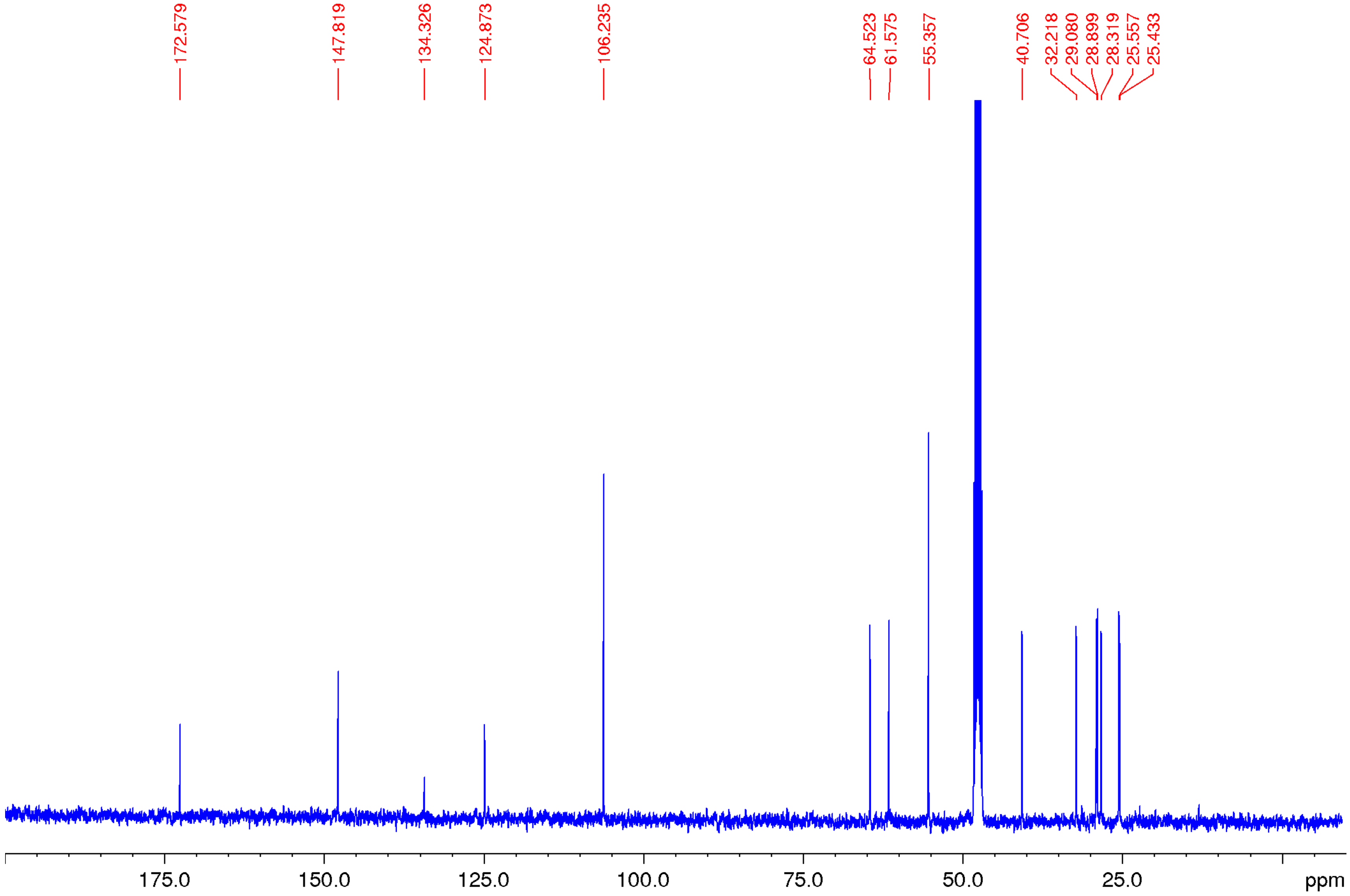
172.579
147.819
134.326
124.873
106.235
64.523
61.575
55.357
40.706
32.218
29.080
28.899
28.319
25.557
25.433
175.0
150.0
125.0
100.0
75.0
50.0
25.0
ppm

HRMS

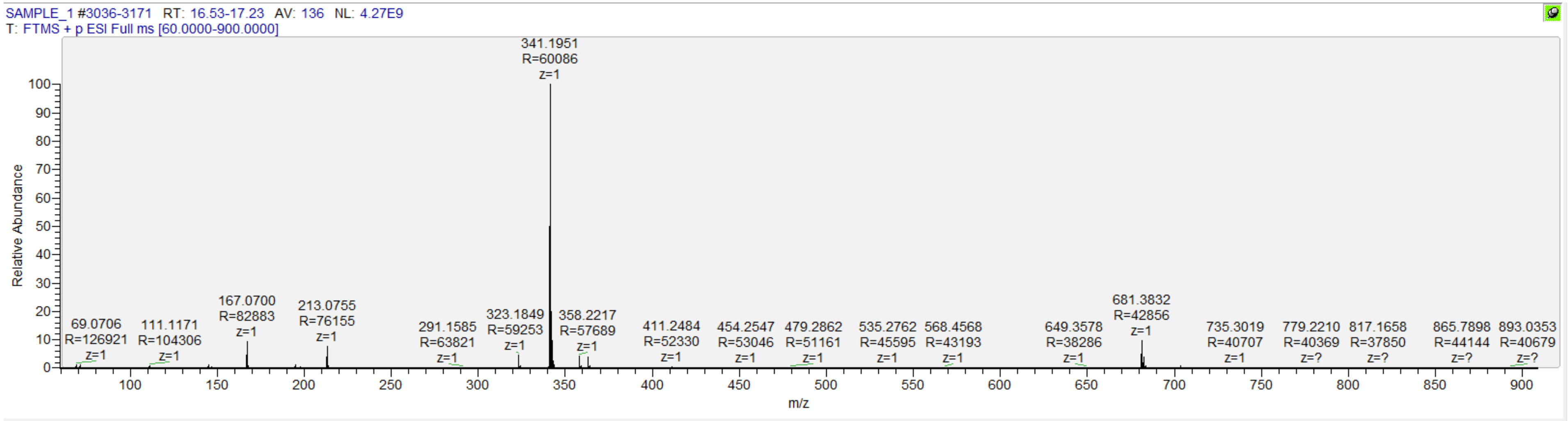

SAMPLE_1 #3036-3171 RT: 16.53-17.23 AV: 136 NL: 4.27E9
T: FTMS + p ESI Full ms [60.0000-900.0000]
341.1951
R=60086
z=1
Relative Abundance
m/z
69.0706
R=126921
111.1171
R=104306
167.0700
R=82883
213.0755
R=76155
291.1585
R=63821
323.1849
R=59253
358.2217
R=57689
411.2484
R=52330
454.2547
R=53046
479.2862
R=51161
535.2762
R=45595
568.4568
R=43193
649.3578
R=38286
681.3832
R=42856
735.3019
R=40707
779.2210
R=40369
817.1658
R=37850
865.7898
R=44144
893.0353
R=40679

FTIR

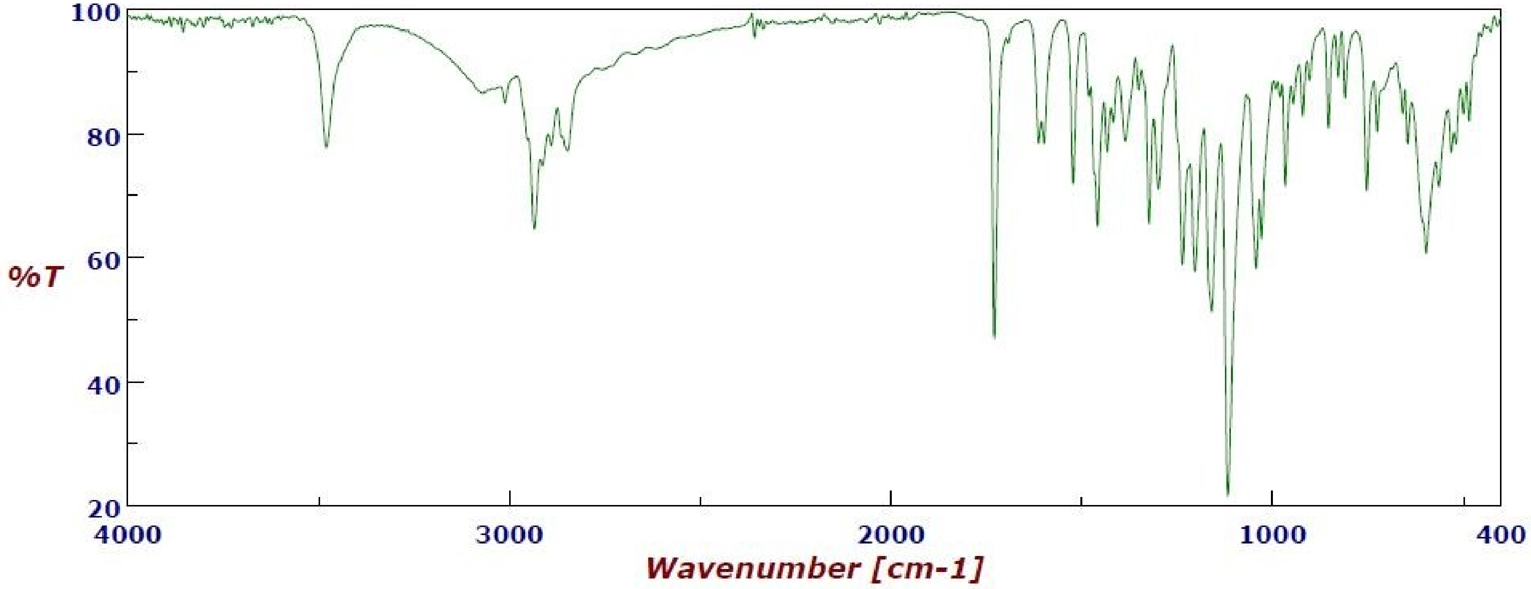

%T
Wavenumber [cm-1]
100
80
60
40
20
4000
3000
2000
1000
400

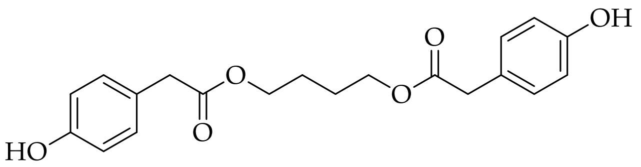

Butane-1,4-diyl bis(2-(4-hydroxyphenyl)acetate) **30**

^{1}H NMR

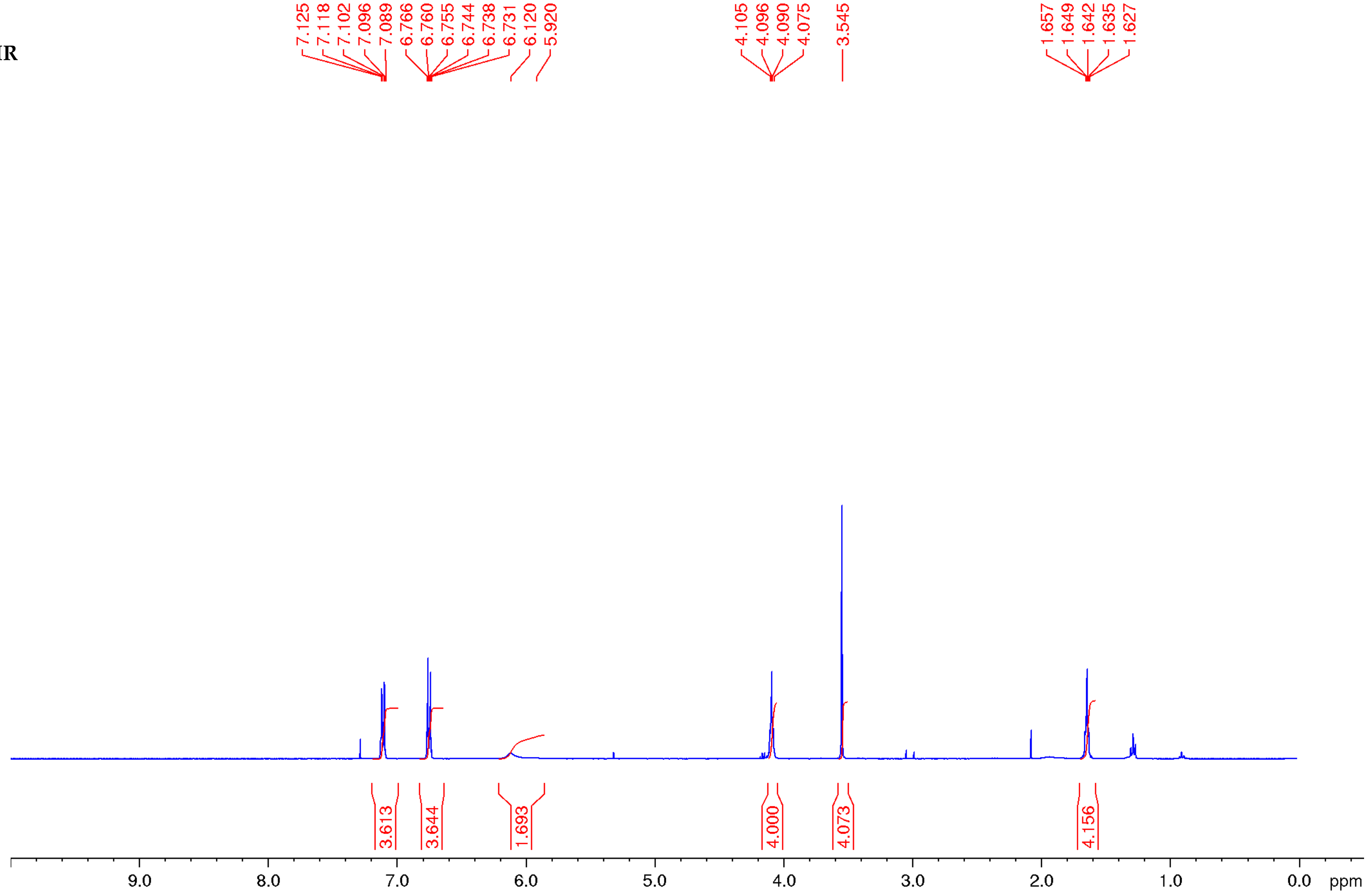

^{13}C NMR

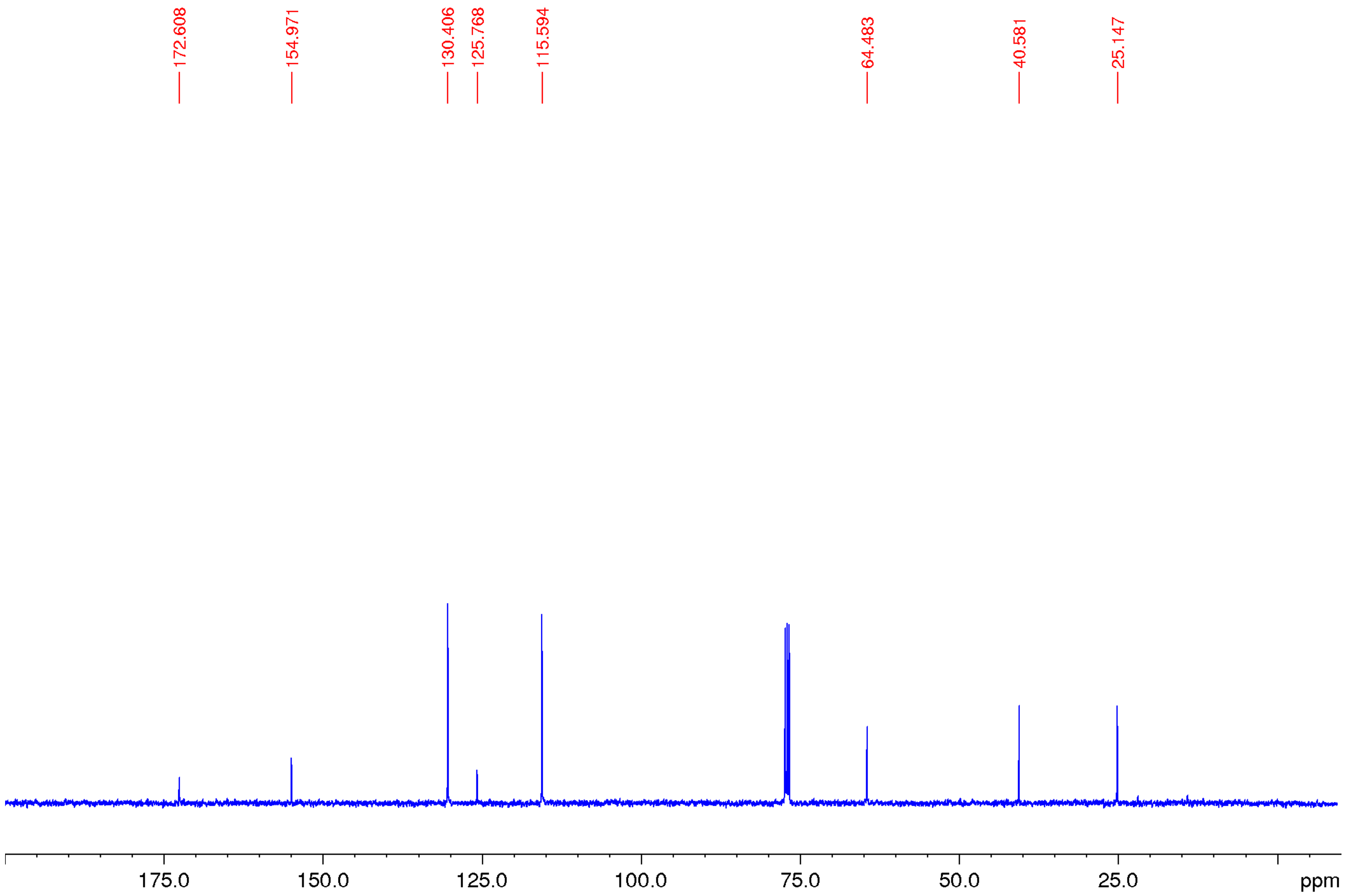

172.608
154.971
130.406
125.768
115.594
64.483
40.581
25.147
175.0
150.0
125.0
100.0
75.0
50.0
25.0
ppm

HRMS

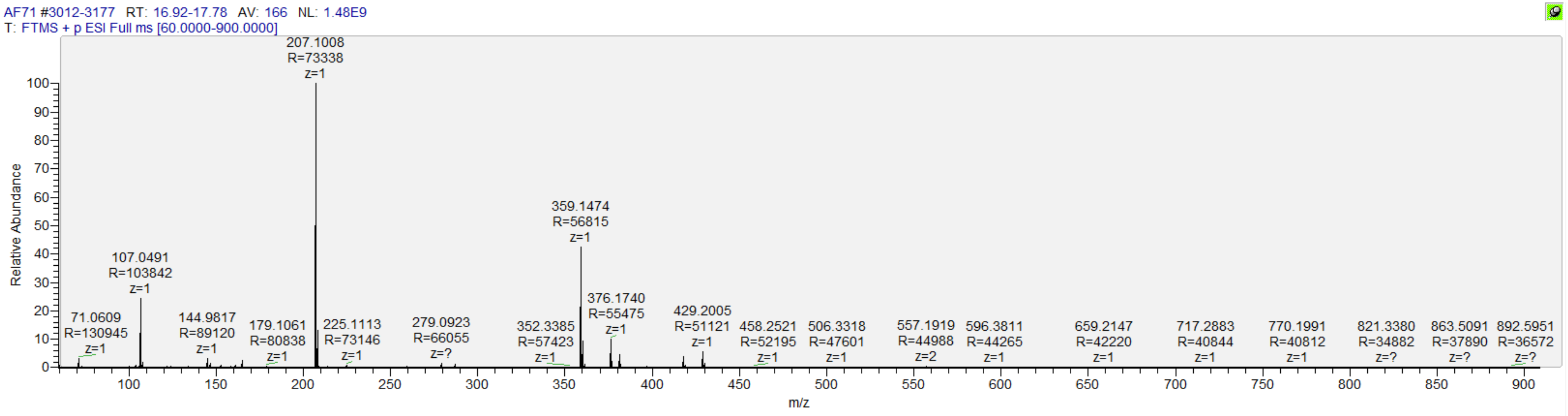

AF71 #3012-3177 RT: 16.92-17.78 AV: 166 NL: 1.48E9
T: FTMS + p ESI Full ms [60.0000-900.0000]
207.1008
R=73338
z=1
359.1474
R=56815
z=1
107.0491
R=103842
z=1
376.1740
R=55475
z=1
71.0609
R=130945
z=1
144.9817
R=89120
z=1
179.1061
R=80838
z=1
225.1113
R=73146
z=1
279.0923
R=66055
z=?
352.3385
R=57423
z=1
429.2005
R=51121
z=1
458.2521
R=52195
z=1
506.3318
R=47601
z=1
557.1919
R=44988
z=2
596.3811
R=44265
z=1
659.2147
R=42220
z=1
717.2883
R=40844
z=1
770.1991
R=40812
z=1
821.3380
R=34882
z=?
863.5091
R=37890
z=?
892.5951
R=36572
z=?
Relative Abundance
m/z

FTIR

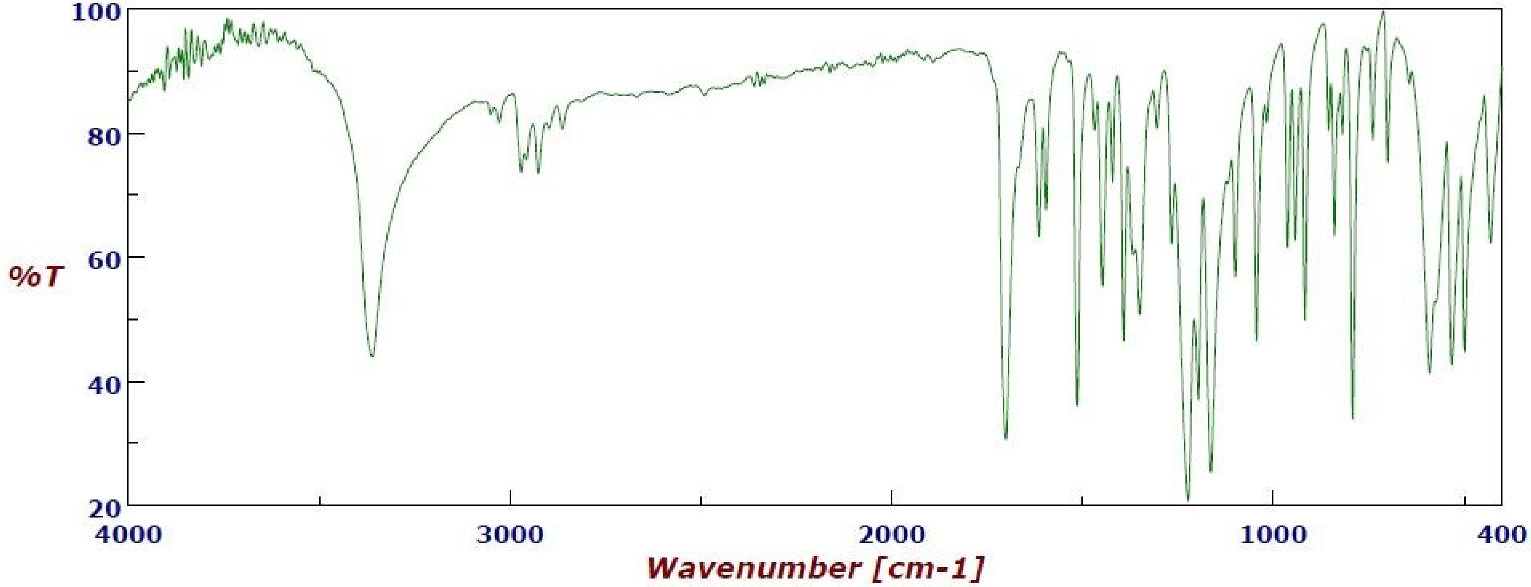

%T
Wavenumber [cm-1]

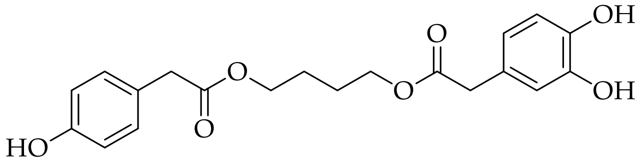

4-(2-(3,4-Dihydroxyphenyl)acetoxy)butyl 2-(4-hydroxyphenyl)acetate **31**

^{1}H NMR

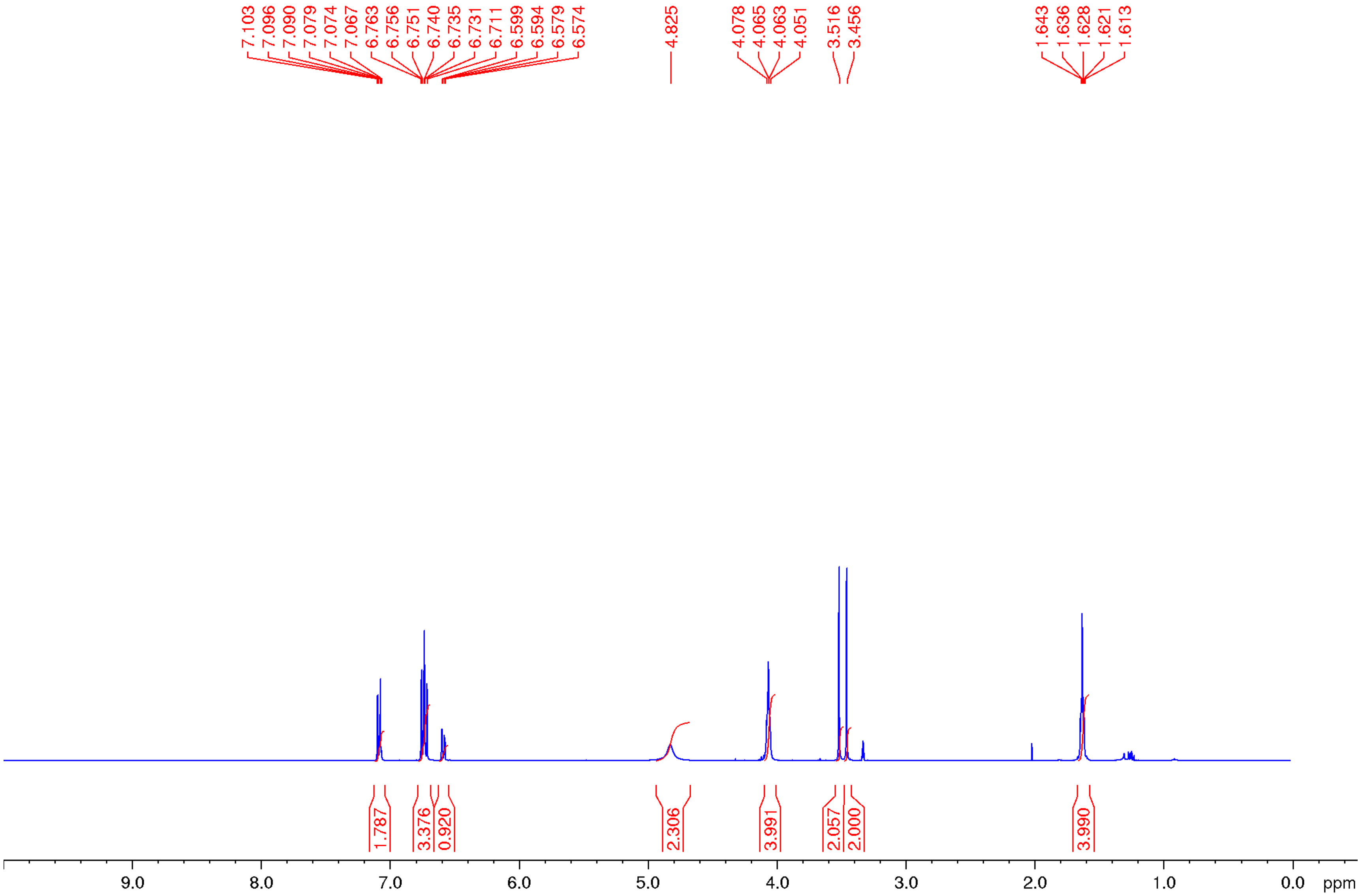

^{13}C NMR

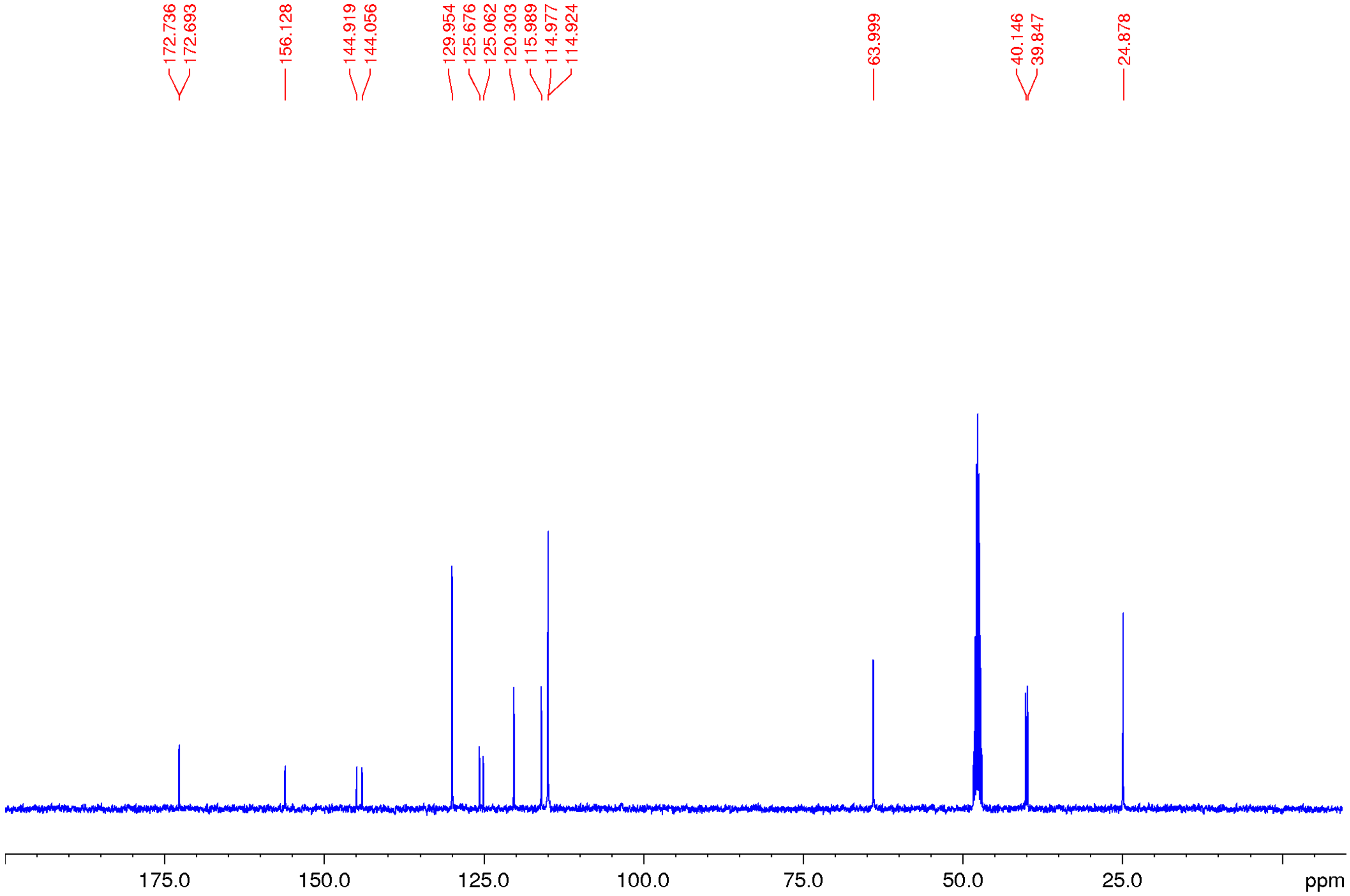

172.736
172.693
156.128
144.919
144.056
129.954
125.676
125.062
120.303
115.989
114.977
114.924
63.999
40.146
39.847
24.878
175.0
150.0
125.0
100.0
75.0
50.0
25.0
ppm

HRMS

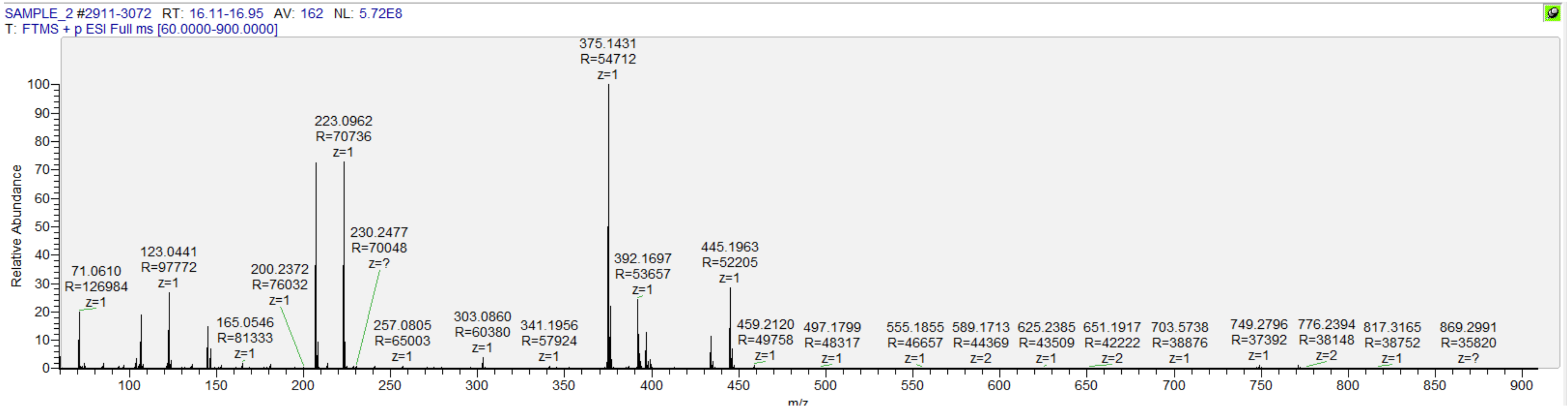
SAMPLE_2 #2911-3072 RT: 16.11-16.95 AV: 162 NL: 5.72E8
T: FTMS + p ESI Full ms [60.0000-900.0000]
375.1431 R=54712 z=1
223.0962 R=70736 z=1
230.2477 R=70048 z=?
200.2372 R=76032 z=1
123.0441 R=97772 z=1
71.0610 R=126984 z=1
165.0546 R=81333 z=1
257.0805 R=65003 z=1
303.0860 R=60380 z=1
341.1956 R=57924 z=1
392.1697 R=53657 z=1
445.1963 R=52205 z=1
459.2120 R=49758 z=1
497.1799 R=48317 z=1
555.1855 R=46657 z=1
589.1713 R=44369 z=2
625.2385 R=43509 z=1
651.1917 R=42222 z=2
703.5738 R=38876 z=1
749.2796 R=37392 z=1
776.2394 R=38148 z=2
817.3165 R=38752 z=1
869.2991 R=35820 z=?
Relative Abundance
m/z

FTIR

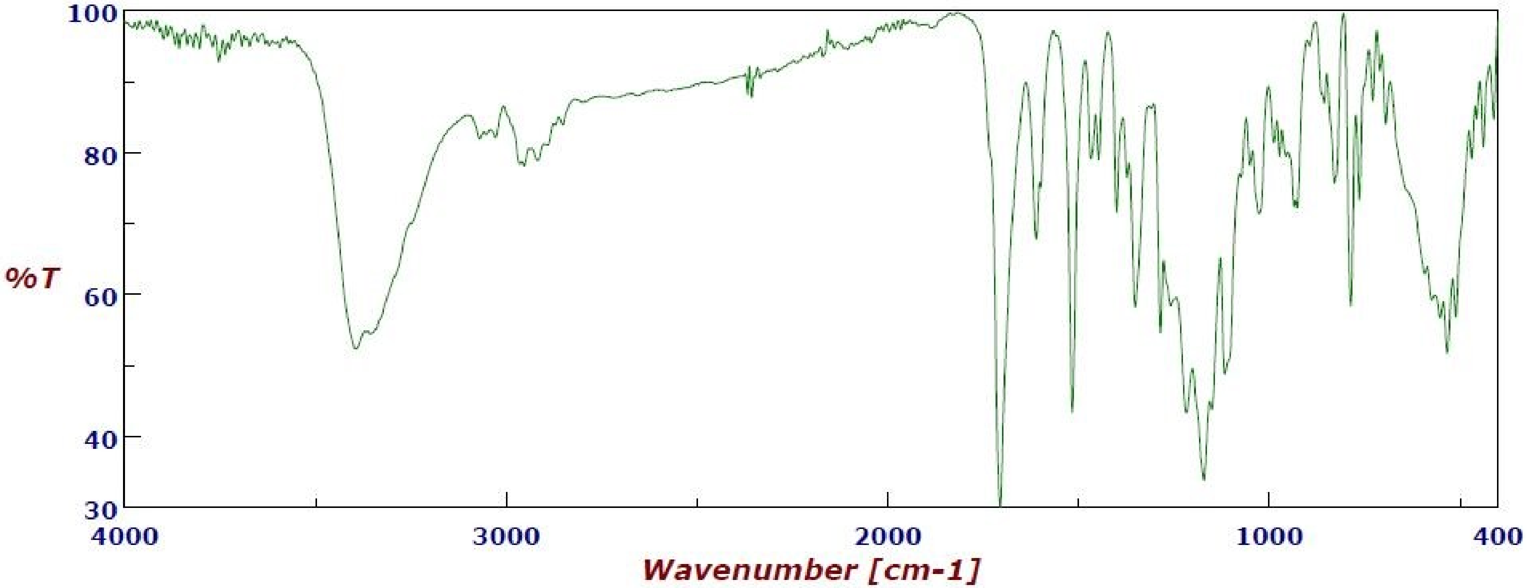
%T
Wavenumber [cm-1]

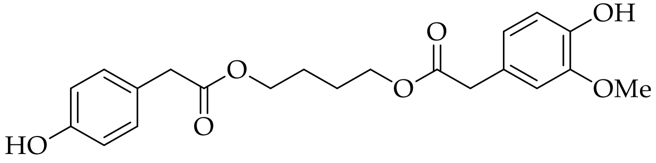

4-(2-(4-Hydroxy-3-methoxyphenyl)acetoxy)butyl 2-(4-hydroxyphenyl)acetate **32**

^{1}H NMR

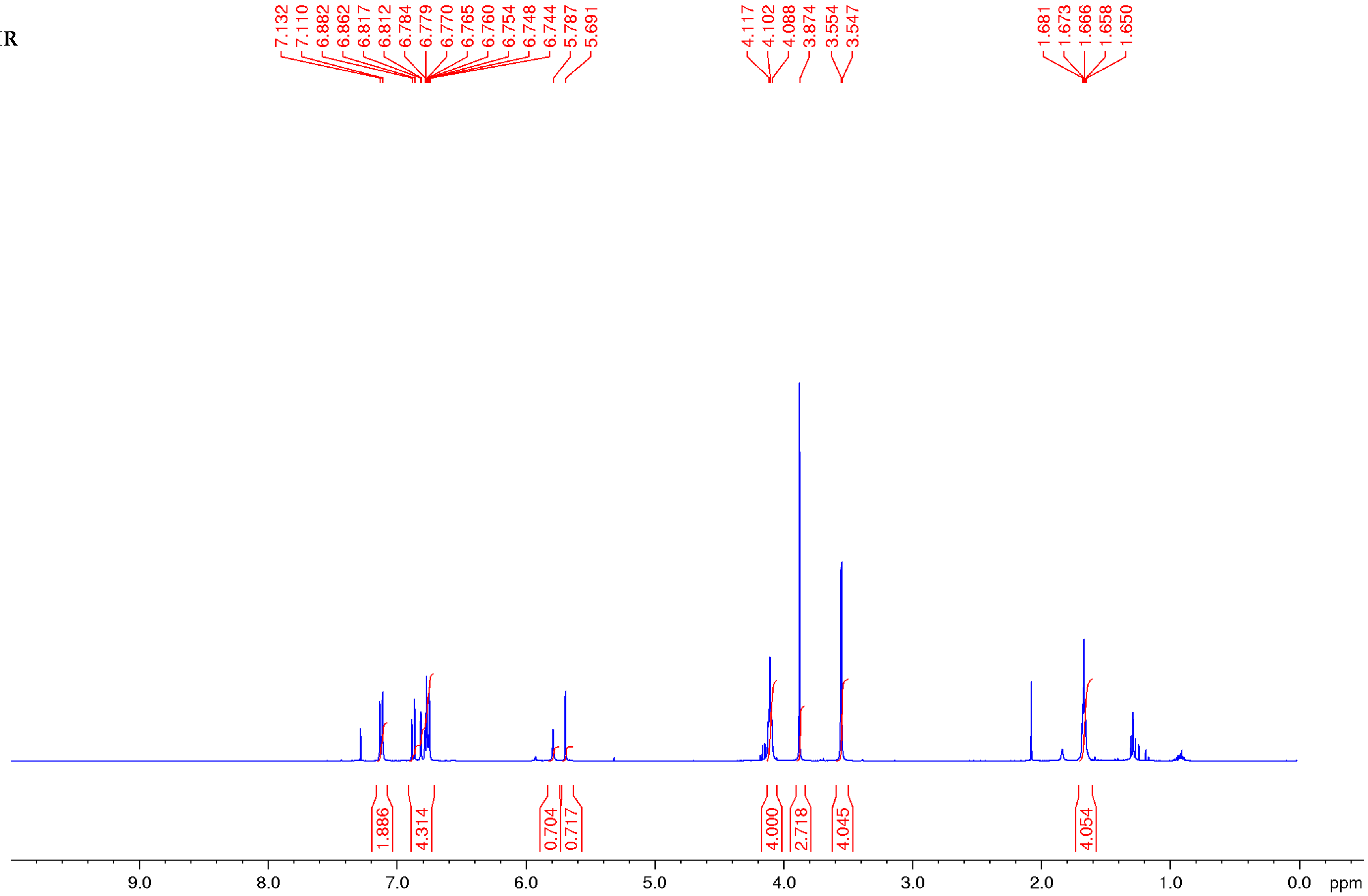

^{13}C NMR

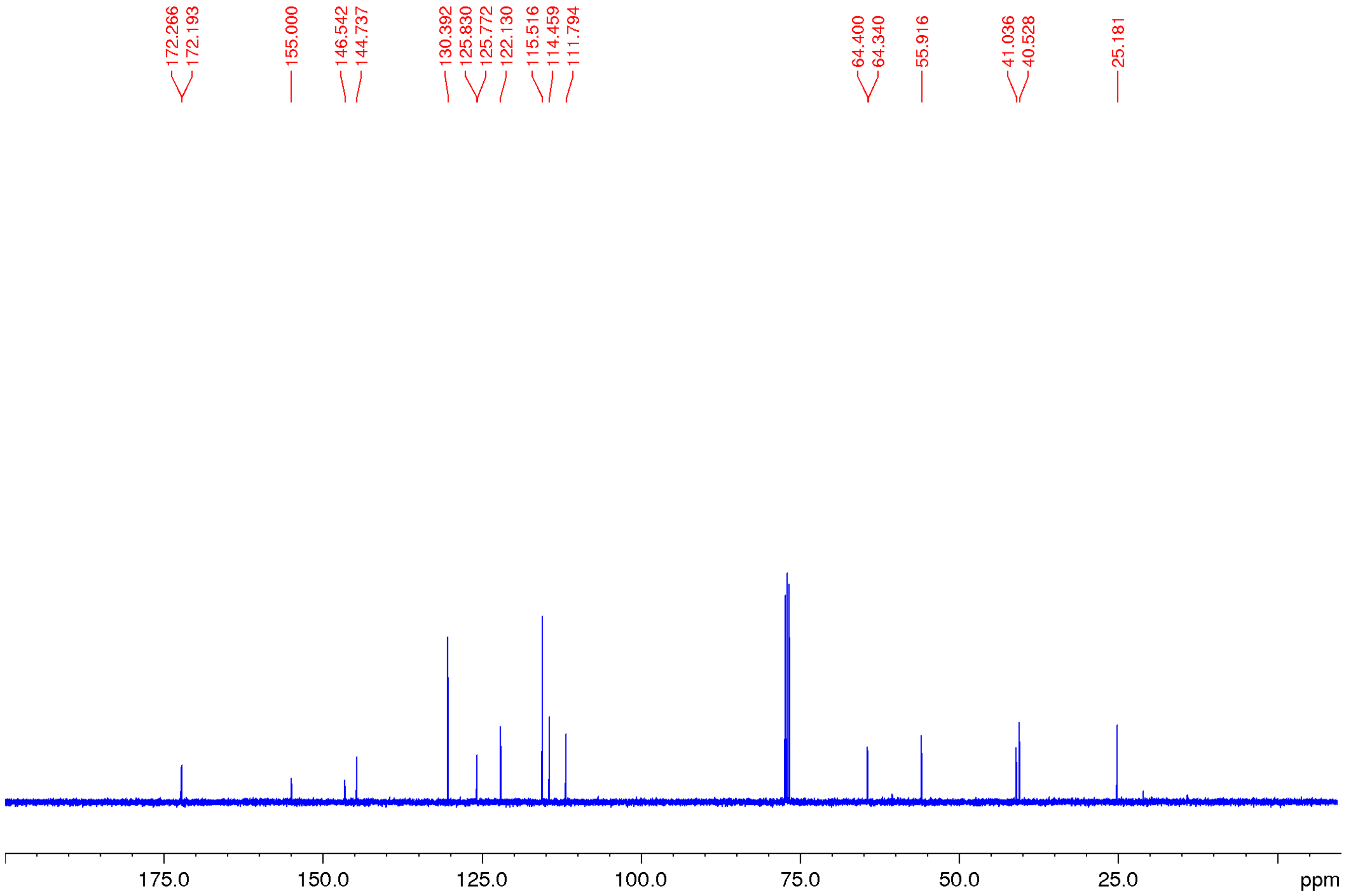

172.266
172.193
155.000
146.542
144.737
130.392
125.830
125.772
122.130
115.516
114.459
111.794
64.400
64.340
55.916
41.036
40.528
25.181
175.0
150.0
125.0
100.0
75.0
50.0
25.0
ppm

HRMS

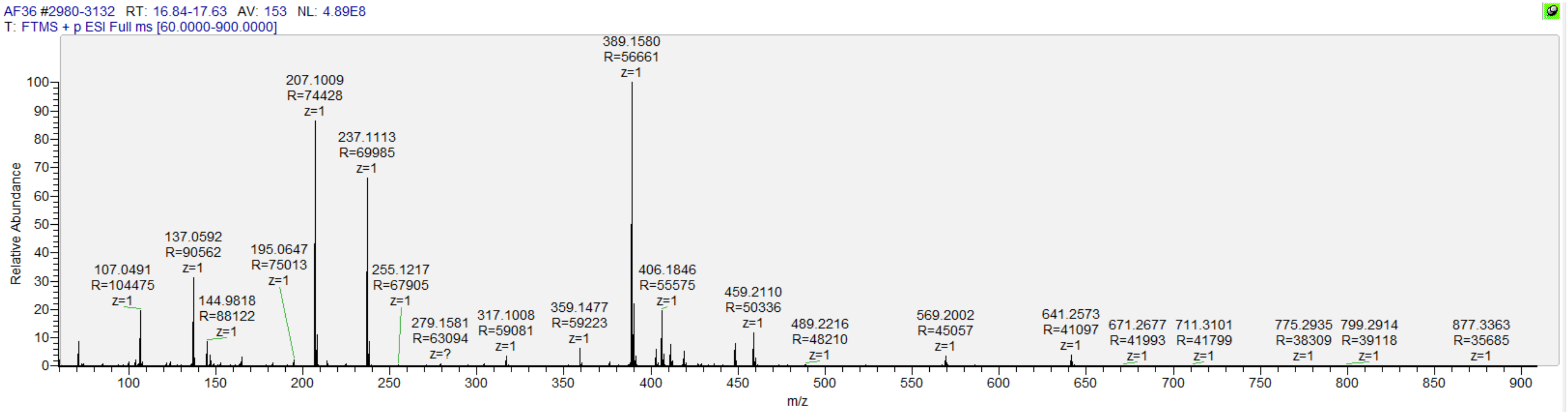
AF36 #2980-3132 RT: 16.84-17.63 AV: 153 NL: 4.89E8
T: FTMS + p ESI Full ms [60.0000-900.0000]
389.1580 R=56661 z=1
207.1009 R=74428 z=1
237.1113 R=69985 z=1
137.0592 R=90562 z=1
195.0647 R=75013 z=1
107.0491 R=104475 z=1
255.1217 R=67905 z=1
406.1846 R=55575 z=1
144.9818 R=88122 z=1
459.2110 R=50336 z=1
279.1581 R=63094 z=?
317.1008 R=59081 z=1
359.1477 R=59223 z=1
489.2216 R=48210 z=1
569.2002 R=45057 z=1
641.2573 R=41097 z=1
671.2677 R=41993 z=1
711.3101 R=41799 z=1
775.2935 R=38309 z=1
799.2914 R=39118 z=1
877.3363 R=35685 z=1
Relative Abundance
m/z

FTIR

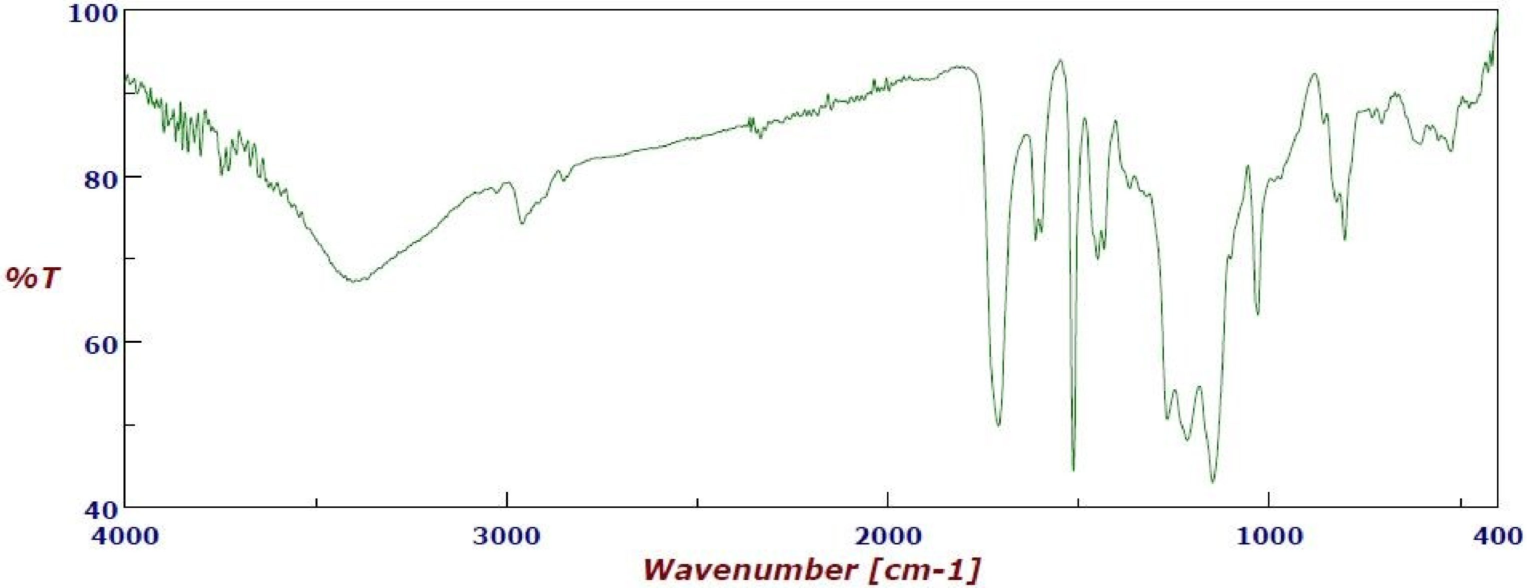
%T
Wavenumber [cm-1]

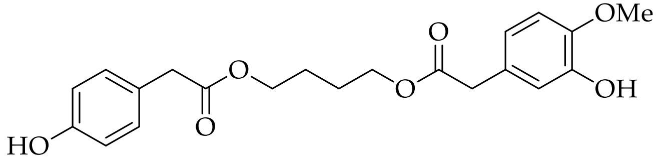

4-(2-(3-Hydroxy-4-methoxyphenyl)acetoxy)butyl 2-(4-hydroxyphenyl)acetate **33**

^{1}H NMR

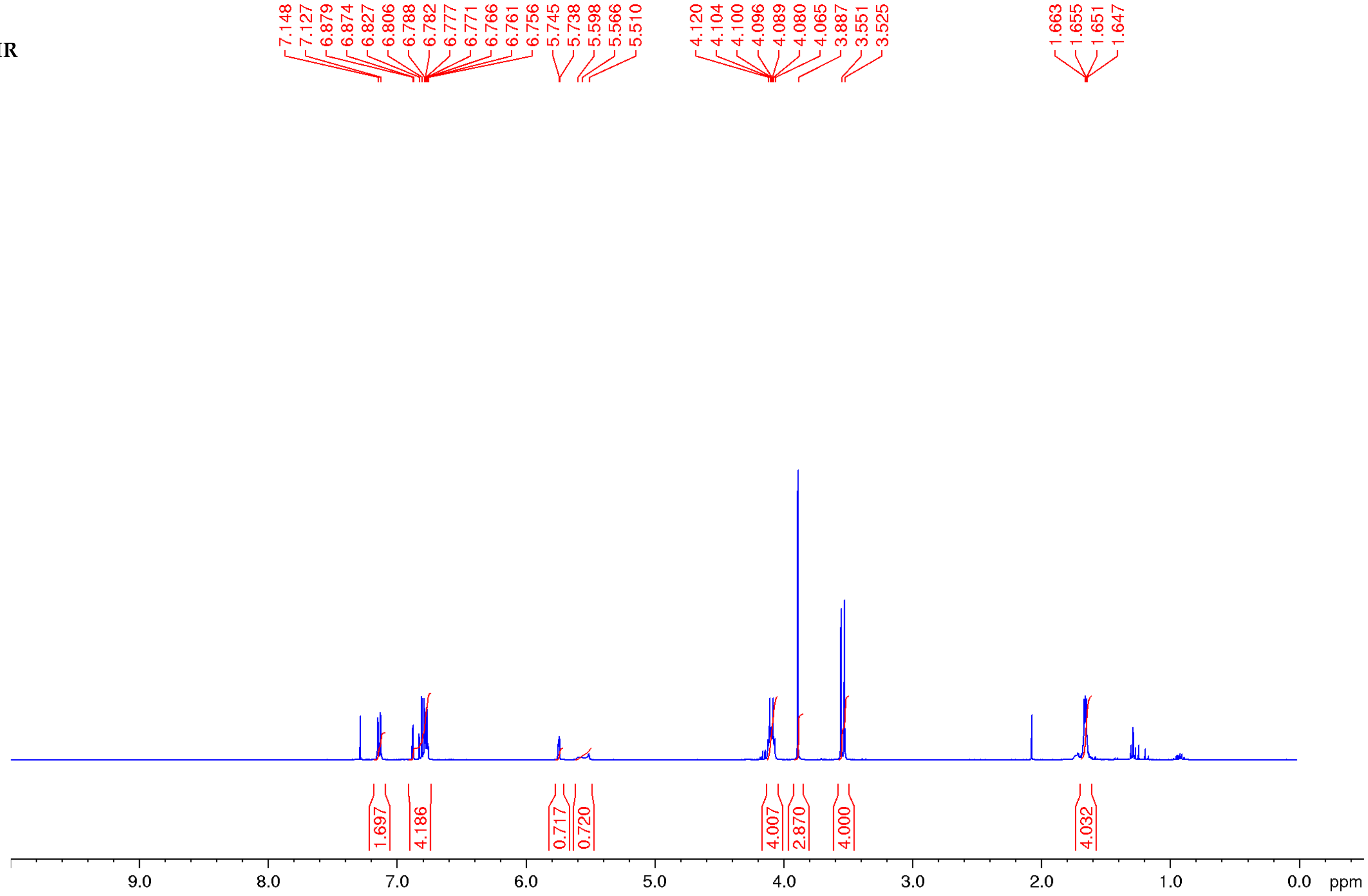

^{13}C NMR

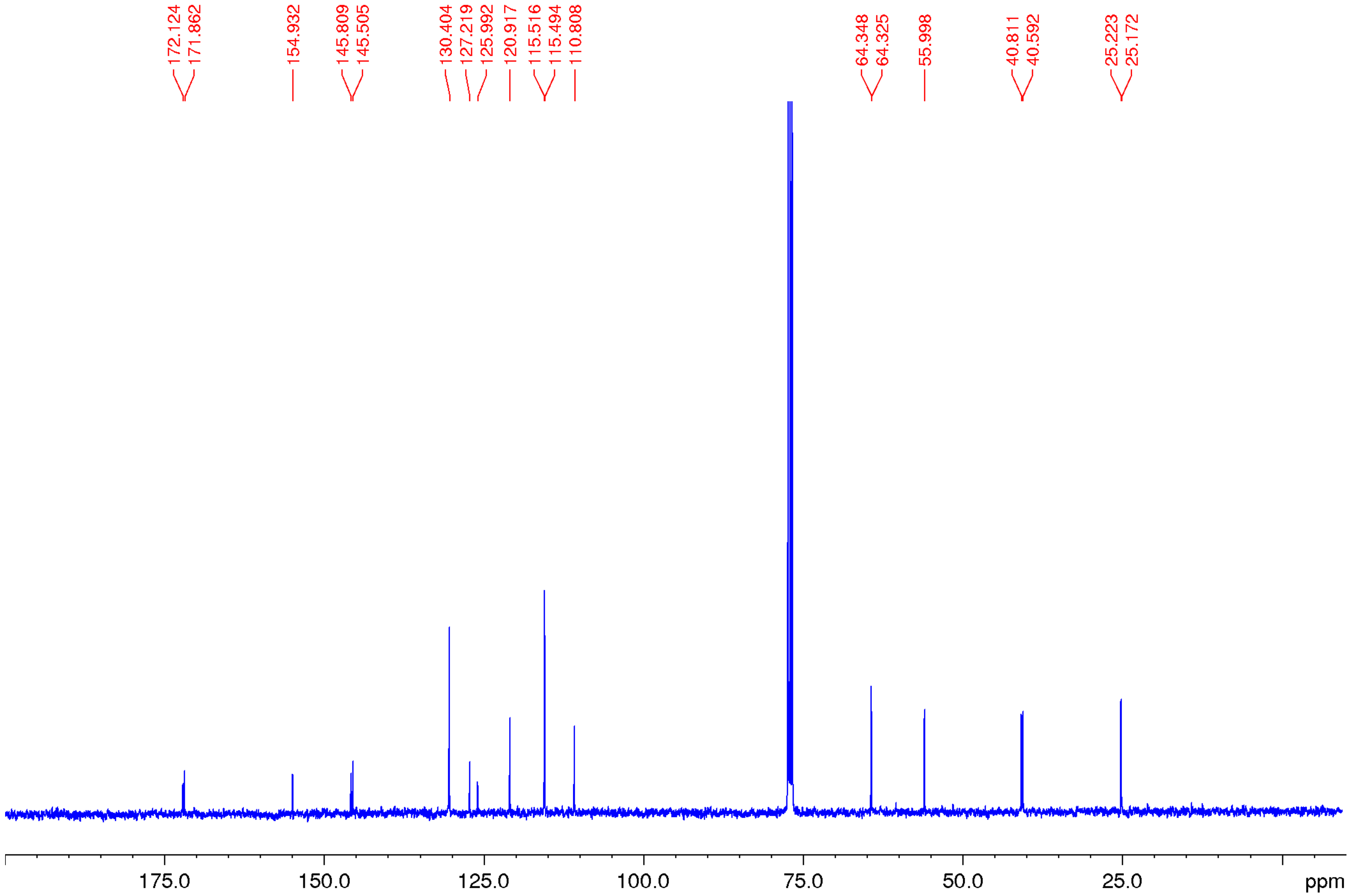

172.124
171.862
154.932
145.809
145.505
130.404
127.219
125.992
120.917
115.516
115.494
110.808
64.348
64.325
55.998
40.811
40.592
25.223
25.172
175.0
150.0
125.0
100.0
75.0
50.0
25.0
ppm

HRMS

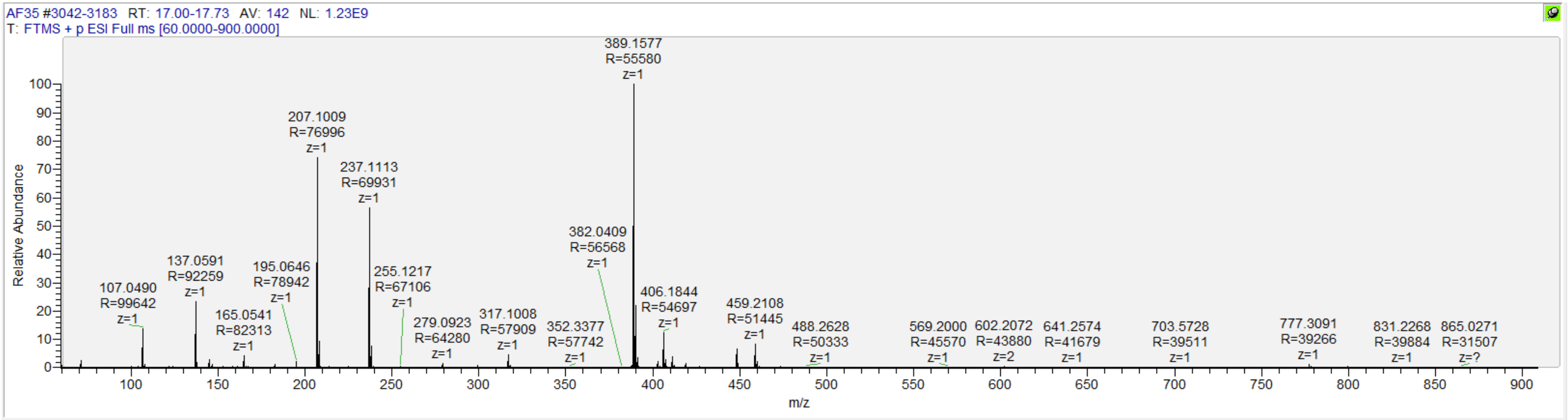
AF35 #3042-3183 RT: 17.00-17.73 AV: 142 NL: 1.23E9
T: FTMS + p ESI Full ms [60.0000-900.0000]
Relative Abundance
m/z
389.1577 R=55580 z=1
207.1009 R=76996 z=1
237.1113 R=69931 z=1
382.0409 R=56568 z=1
137.0591 R=92259 z=1
195.0646 R=78942 z=1
255.1217 R=67106 z=1
107.0490 R=99642 z=1
406.1844 R=54697 z=1
459.2108 R=51445 z=1
165.0541 R=82313 z=1
279.0923 R=64280 z=1
317.1008 R=57909 z=1
352.3377 R=57742 z=1
488.2628 R=50333 z=1
569.2000 R=45570 z=1
602.2072 R=43880 z=2
641.2574 R=41679 z=1
703.5728 R=39511 z=1
777.3091 R=39266 z=1
831.2268 R=39884 z=1
865.0271 R=31507 z=?

FTIR

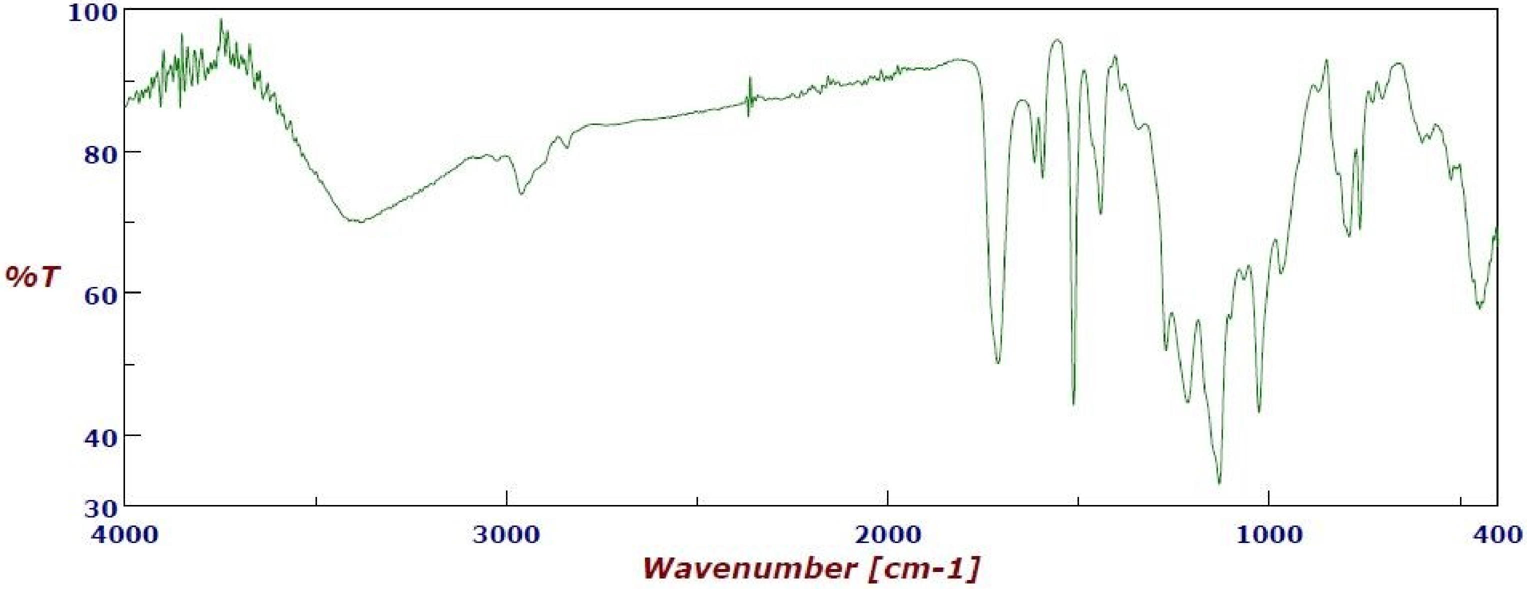
%T
Wavenumber [cm-1]

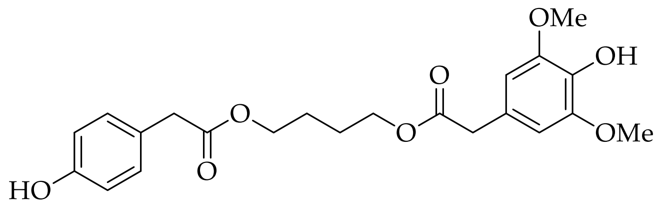

4-(2-(4-Hydroxy-3,5-dimethoxyphenyl)acetoxy)butyl 2-(4-hydroxyphenyl)acetate **34**

^{1}H NMR

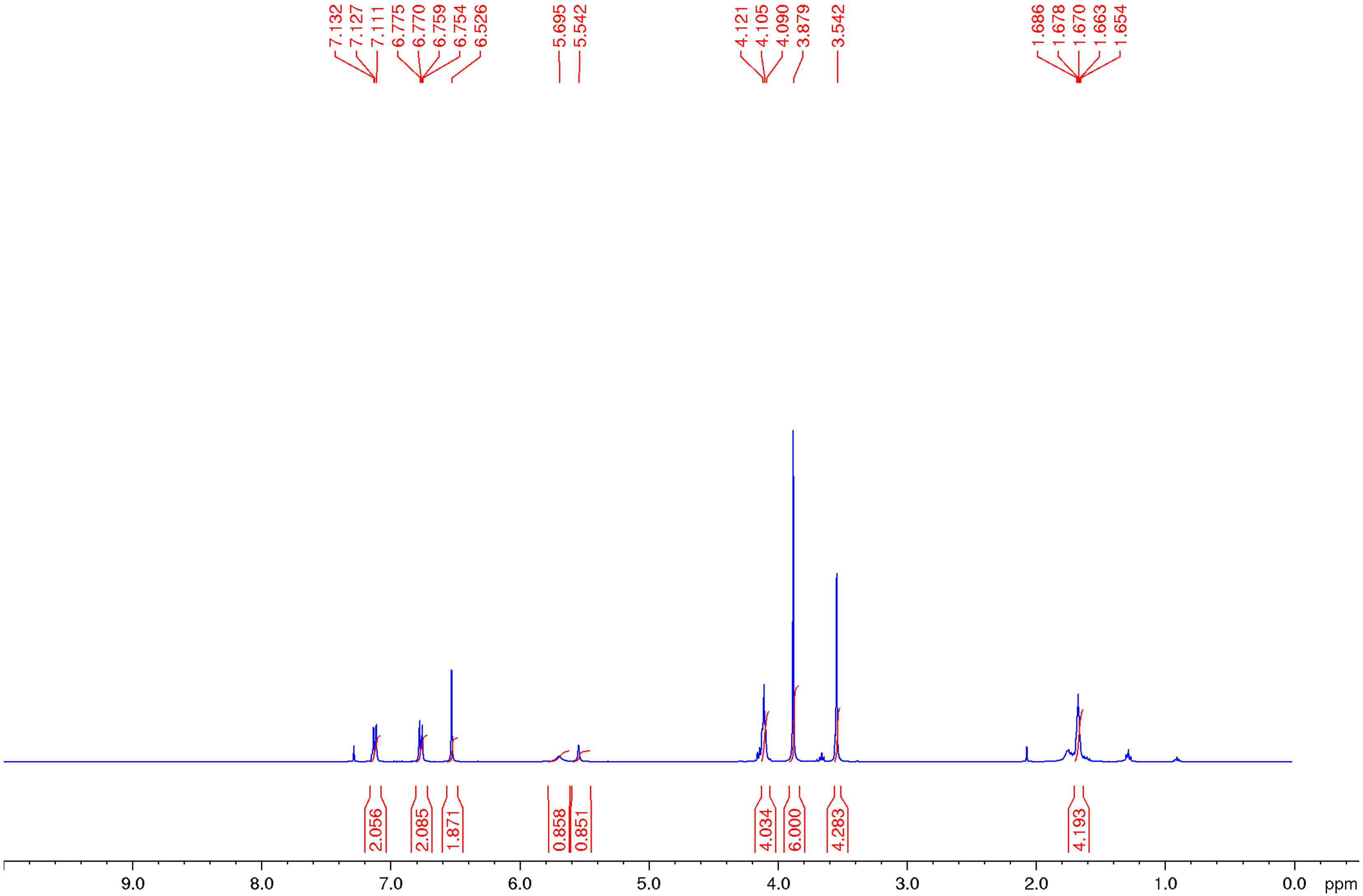

^{13}C NMR

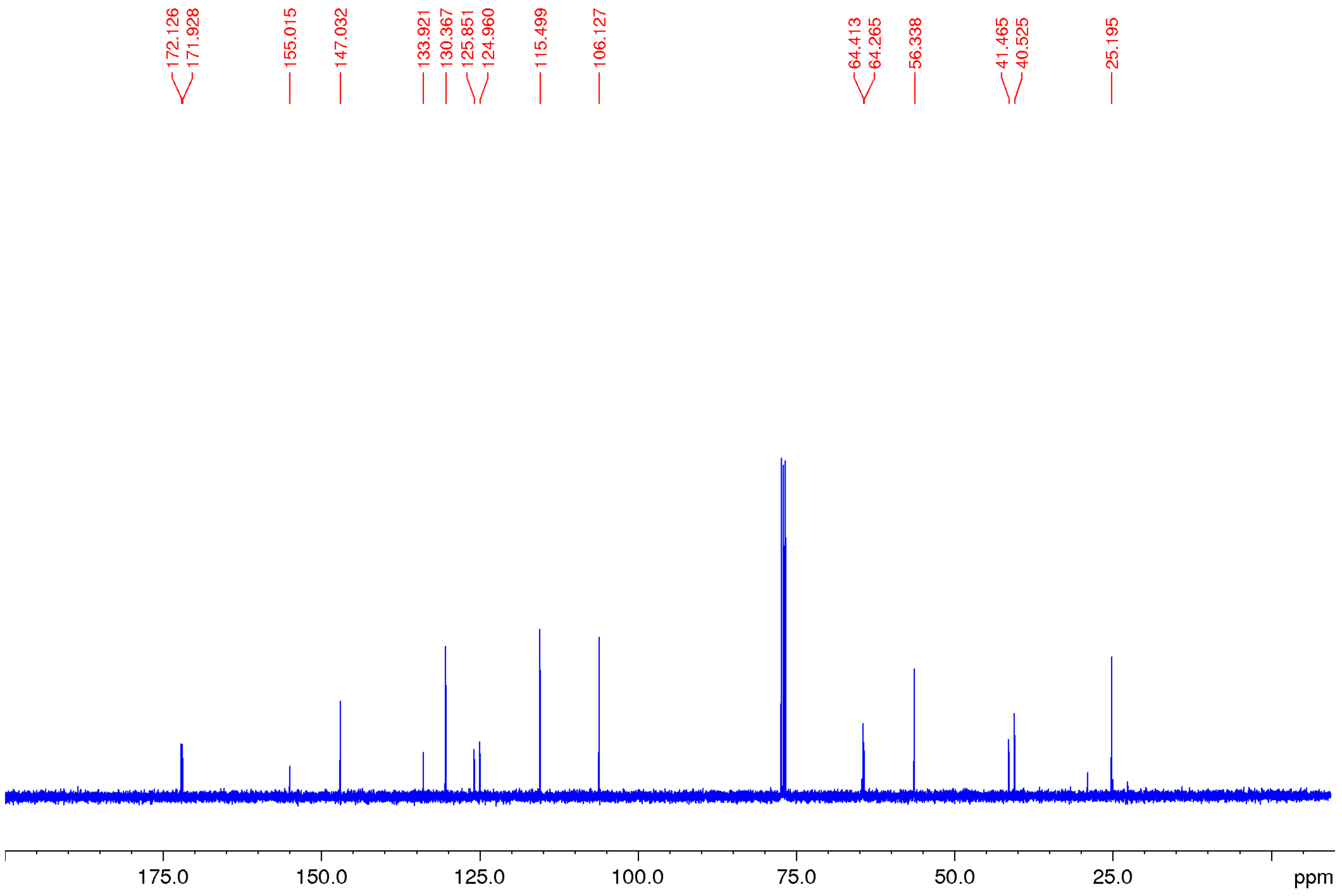

172.126
171.928
155.015
147.032
133.921
130.367
125.851
124.960
115.499
106.127
64.413
64.265
56.338
41.465
40.525
25.195
175.0
150.0
125.0
100.0
75.0
50.0
25.0
ppm

HRMS

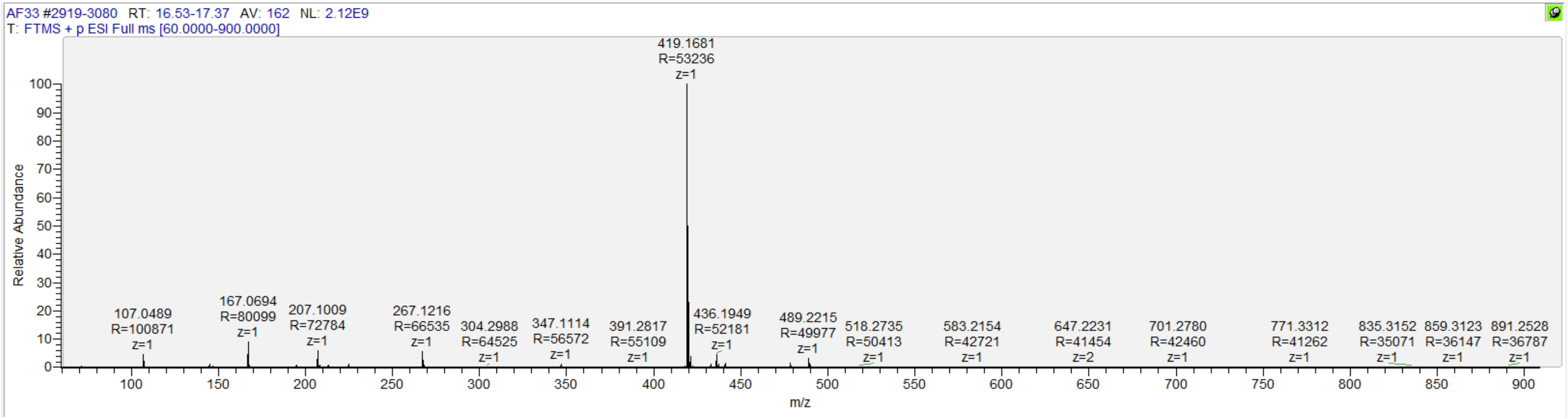
AF33 #2919-3080 RT: 16.53-17.37 AV: 162 NL: 2.12E9
T: FTMS + p ESI Full ms [60.0000-900.0000]
419.1681
R=53236
z=1
Relative Abundance
107.0489 R=100871 z=1
167.0694 R=80099 z=1
207.1009 R=72784 z=1
267.1216 R=66535 z=1
304.2988 R=64525 z=1
347.1114 R=56572 z=1
391.2817 R=55109 z=1
436.1949 R=52181 z=1
489.2215 R=49977 z=1
518.2735 R=50413 z=1
583.2154 R=42721 z=1
647.2231 R=41454 z=2
701.2780 R=42460 z=1
771.3312 R=41262 z=1
835.3152 R=35071 z=1
859.3123 R=36147 z=1
891.2528 R=36787 z=1
m/z

FTIR

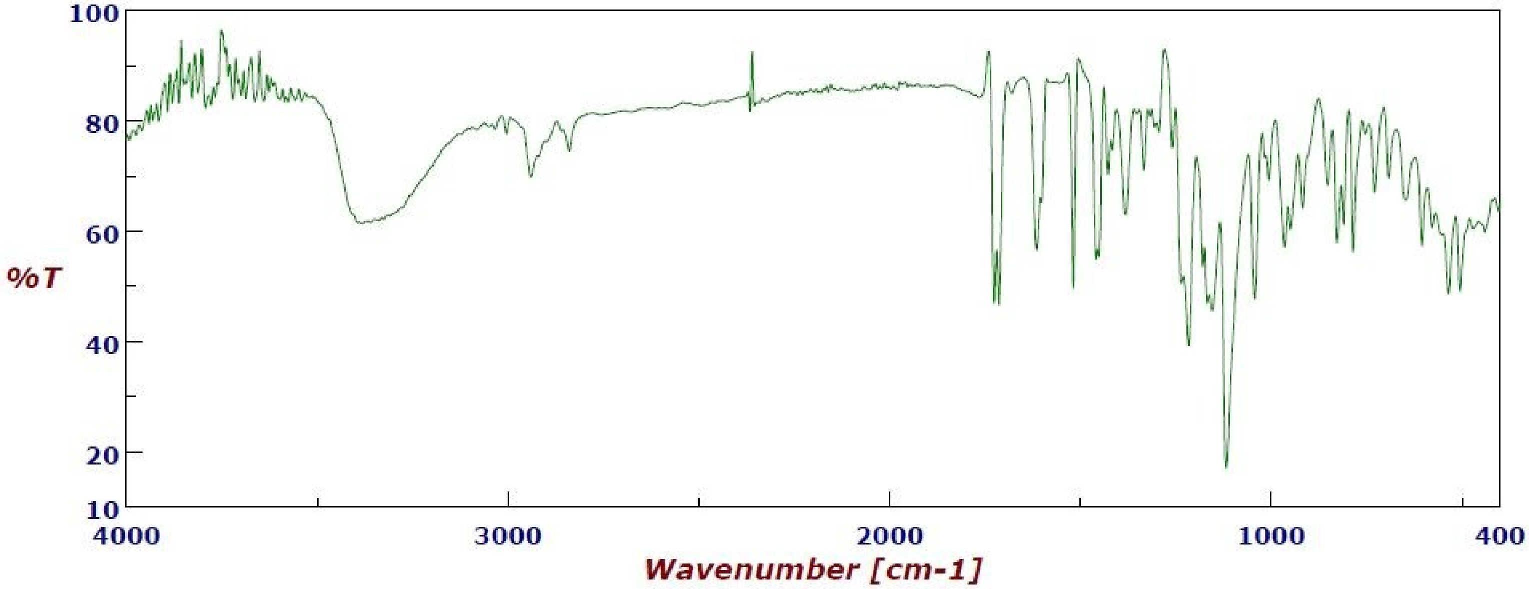
%T
Wavenumber [cm-1]

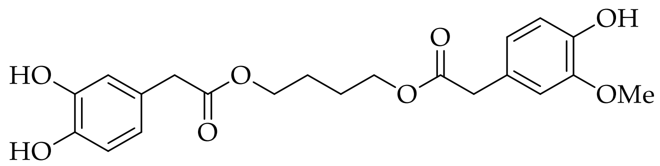

4-(2-(3,4-Dihydroxyphenyl)acetoxy)butyl 2-(4-hydroxy-3-methoxyphenyl)acetate **36**

^{1}H NMR

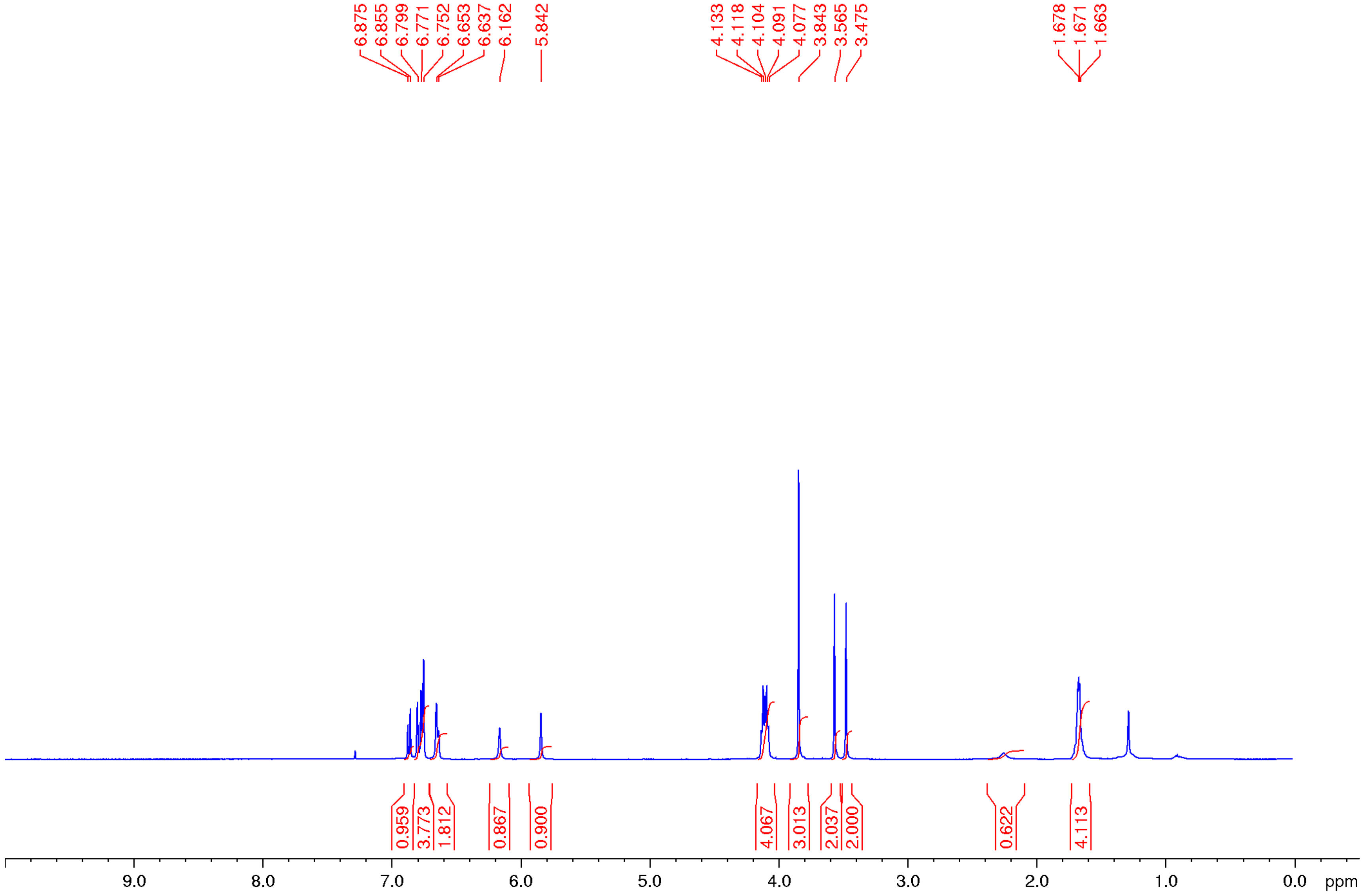

^{13}C NMR

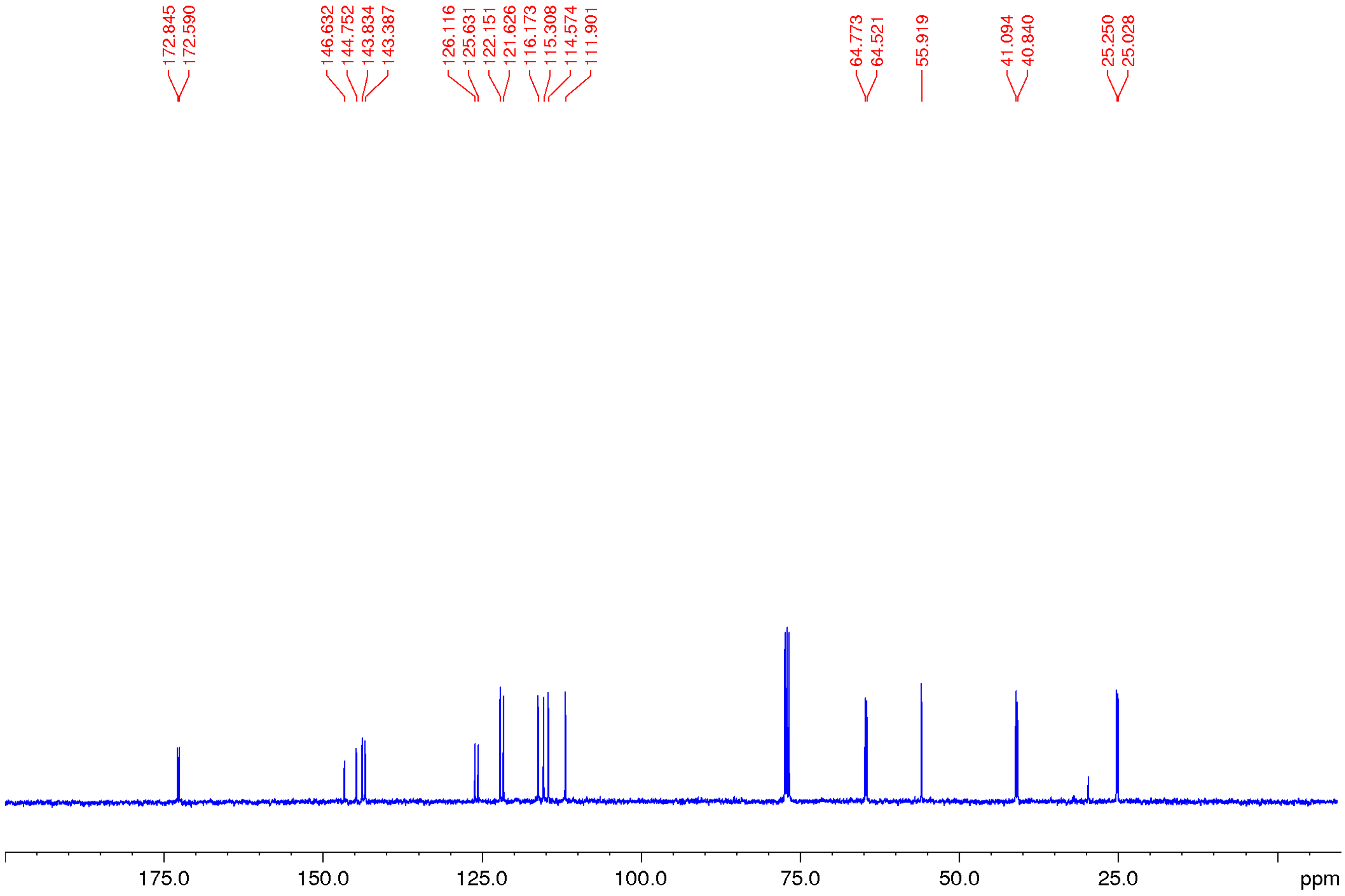

172.845
172.590
146.632
144.752
143.834
143.387
126.116
125.631
122.151
121.626
116.173
115.308
114.574
111.901
64.773
64.521
55.919
41.094
40.840
25.250
25.028
175.0
150.0
125.0
100.0
75.0
50.0
25.0
ppm

HRMS

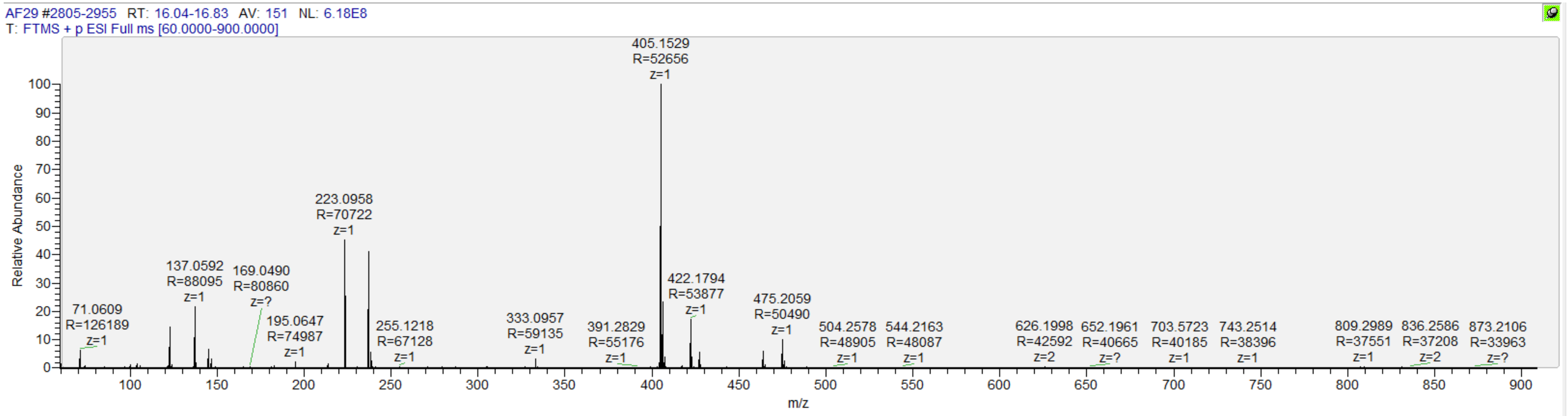

AF29 #2805-2955 RT: 16.04-16.83 AV: 151 NL: 6.18E8
T: FTMS + p ESI Full ms [60.0000-900.0000]
Relative Abundance
m/z
405.1529
R=52656
z=1
223.0958
R=70722
z=1
137.0592
R=88095
z=1
169.0490
R=80860
z=?
71.0609
R=126189
z=1
195.0647
R=74987
z=1
255.1218
R=67128
z=1
333.0957
R=59135
z=1
391.2829
R=55176
z=1
422.1794
R=53877
z=1
475.2059
R=50490
z=1
504.2578
R=48905
z=1
544.2163
R=48087
z=1
626.1998
R=42592
z=2
652.1961
R=40665
z=?
703.5723
R=40185
z=1
743.2514
R=38396
z=1
809.2989
R=37551
z=1
836.2586
R=37208
z=2
873.2106
R=33963
z=?

FTIR

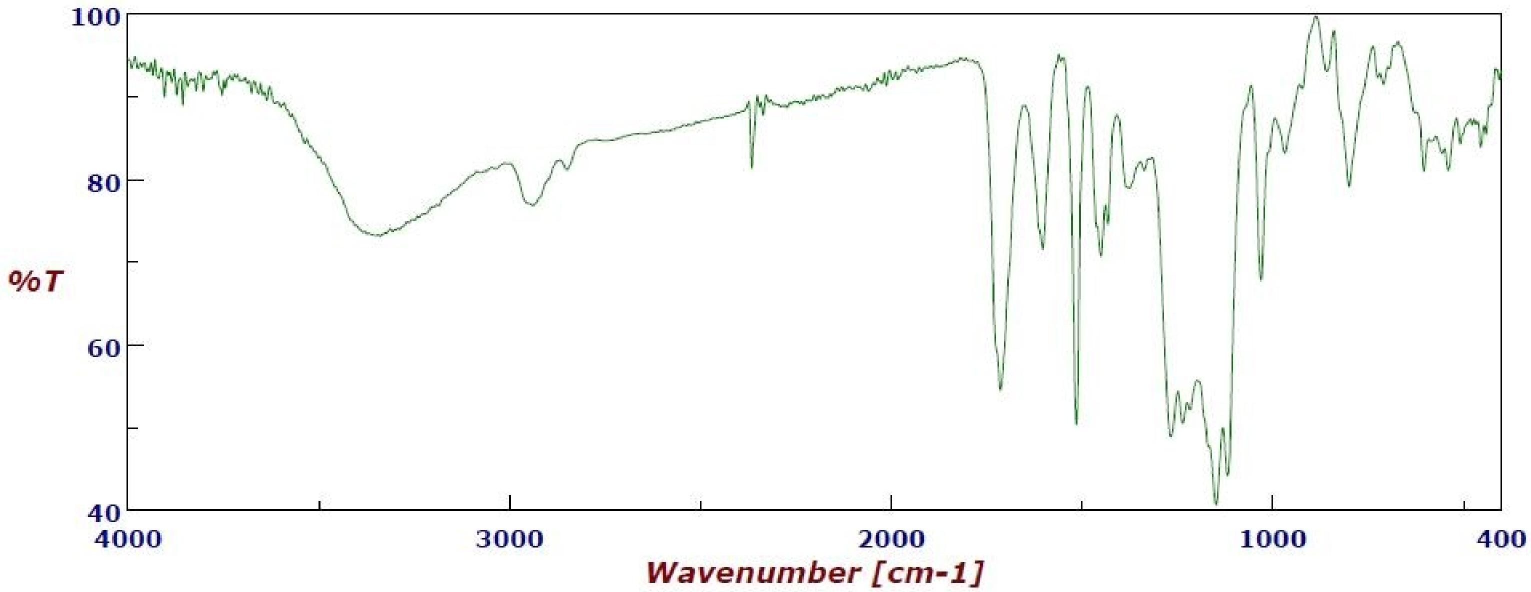

%T
Wavenumber [cm-1]

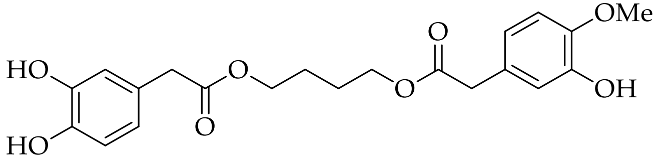

4-(2-(3,4-Dihydroxyphenyl)acetoxy)butyl 2-(3-hydroxy-4-methoxyphenyl)acetate **37**

^{1}H NMR

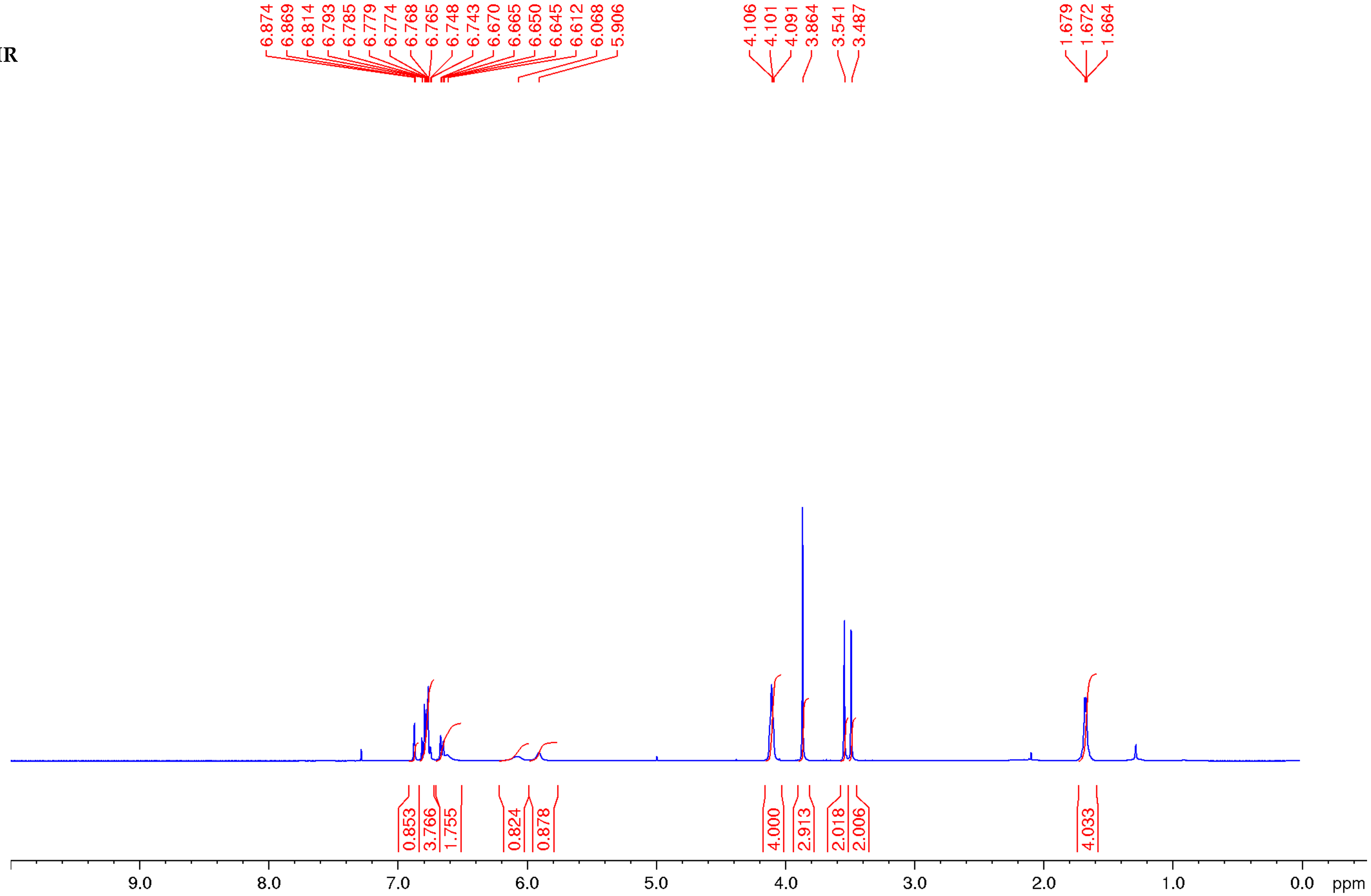

^{13}C NMR

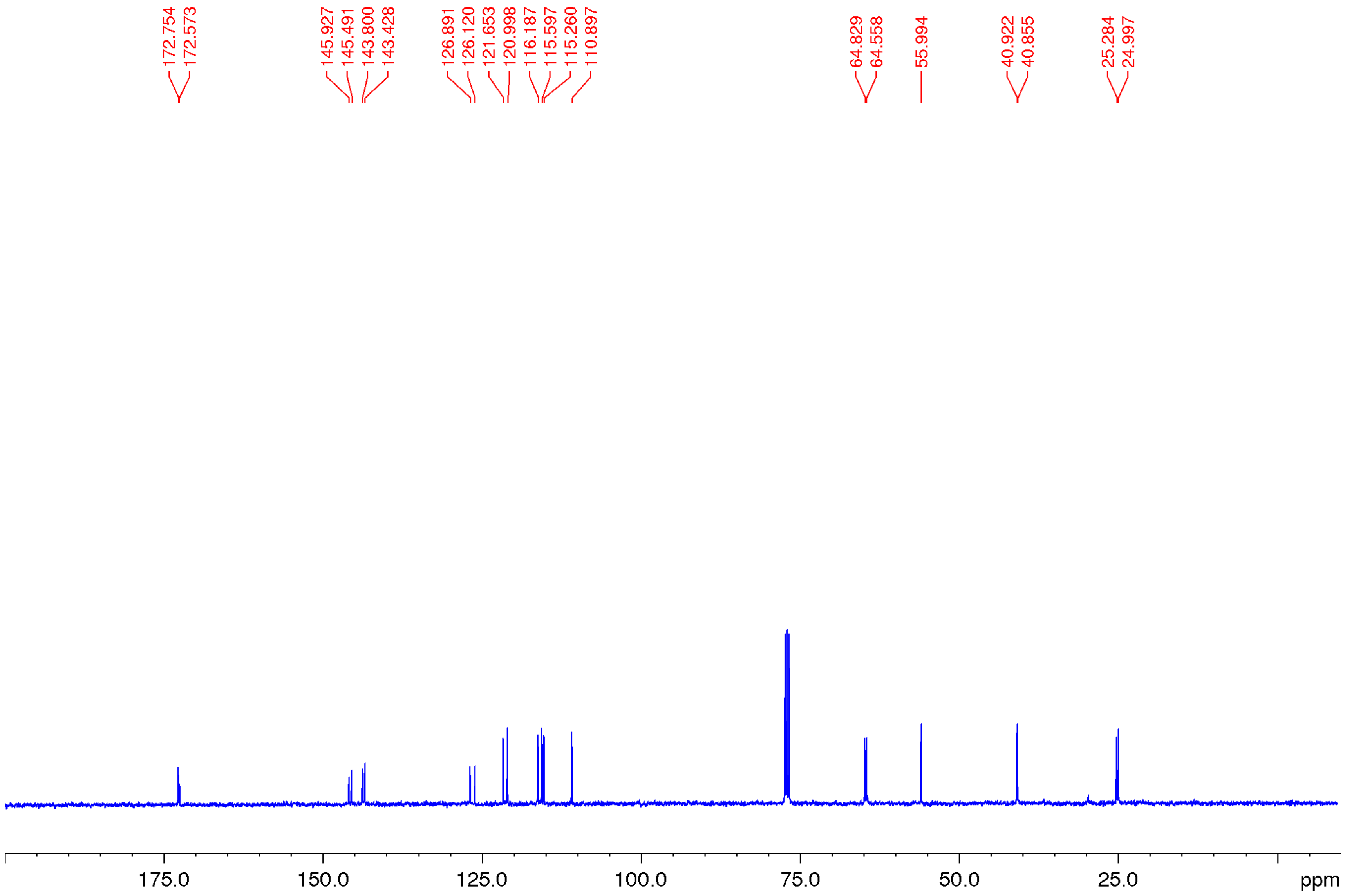

172.754
172.573
145.927
145.491
143.800
143.428
126.891
126.120
121.653
120.998
116.187
115.597
115.260
110.897
64.829
64.558
55.994
40.922
40.855
25.284
24.997
175.0
150.0
125.0
100.0
75.0
50.0
25.0
ppm

HRMS

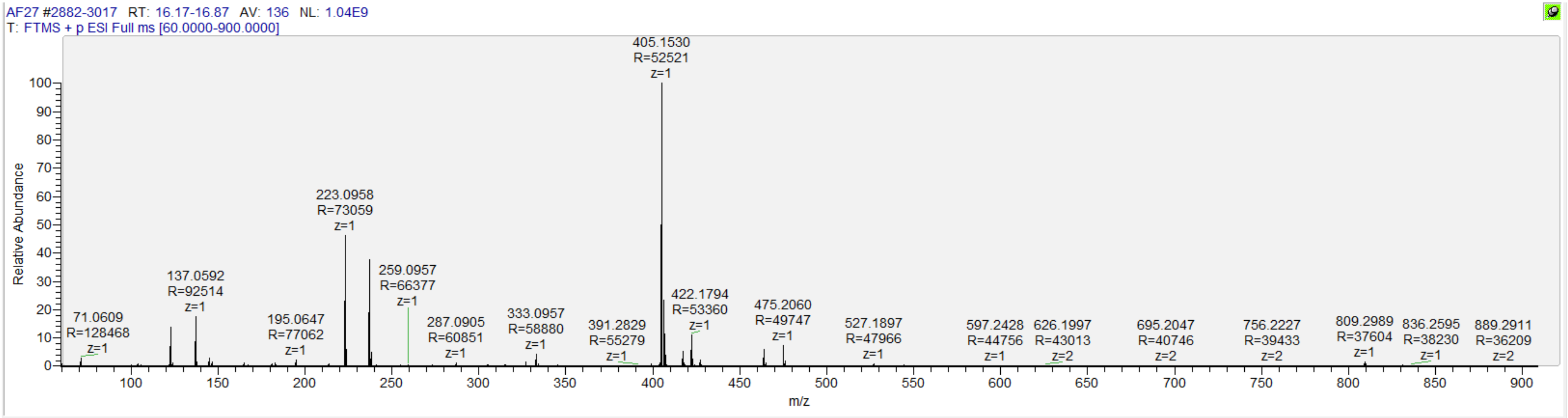
AF27 #2882-3017 RT: 16.17-16.87 AV: 136 NL: 1.04E9
T: FTMS + p ESI Full ms [60.0000-900.0000]
405.1530
R=52521
z=1
223.0958
R=73059
z=1
137.0592
R=92514
z=1
259.0957
R=66377
z=1
71.0609
R=128468
z=1
195.0647
R=77062
z=1
287.0905
R=60851
z=1
333.0957
R=58880
z=1
391.2829
R=55279
z=1
422.1794
R=53360
z=1
475.2060
R=49747
z=1
527.1897
R=47966
z=1
597.2428
R=44756
z=1
626.1997
R=43013
z=2
695.2047
R=40746
z=2
756.2227
R=39433
z=2
809.2989
R=37604
z=1
836.2595
R=38230
z=1
889.2911
R=36209
z=2
Relative Abundance
m/z

FTIR

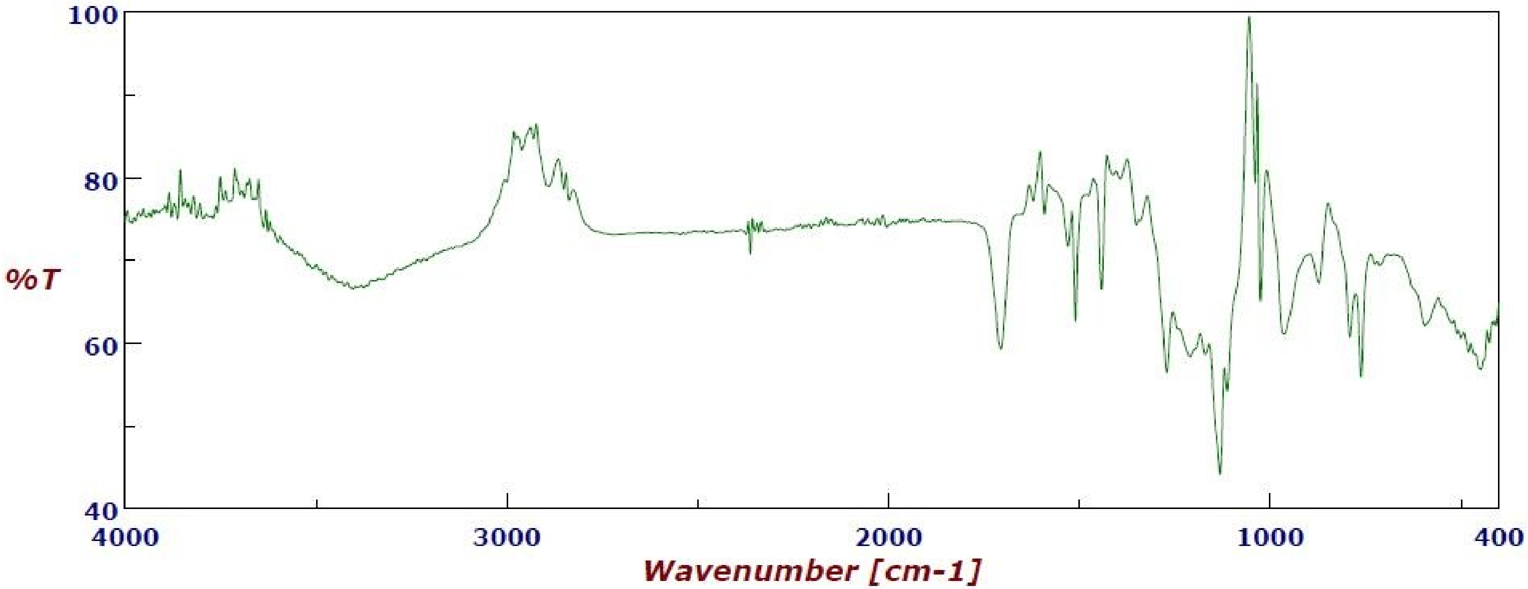
%T
Wavenumber [cm-1]

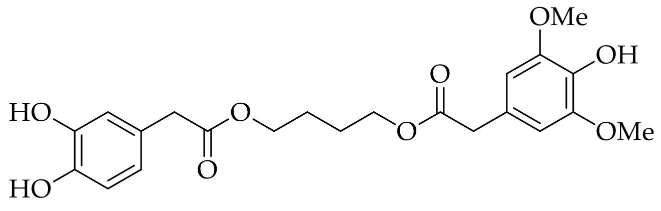

4-(2-(3,4-Dihydroxyphenyl)acetoxy)butyl 2-(4-hydroxy-3,5-dimethoxyphenyl)acetate **38**

^{1}H NMR

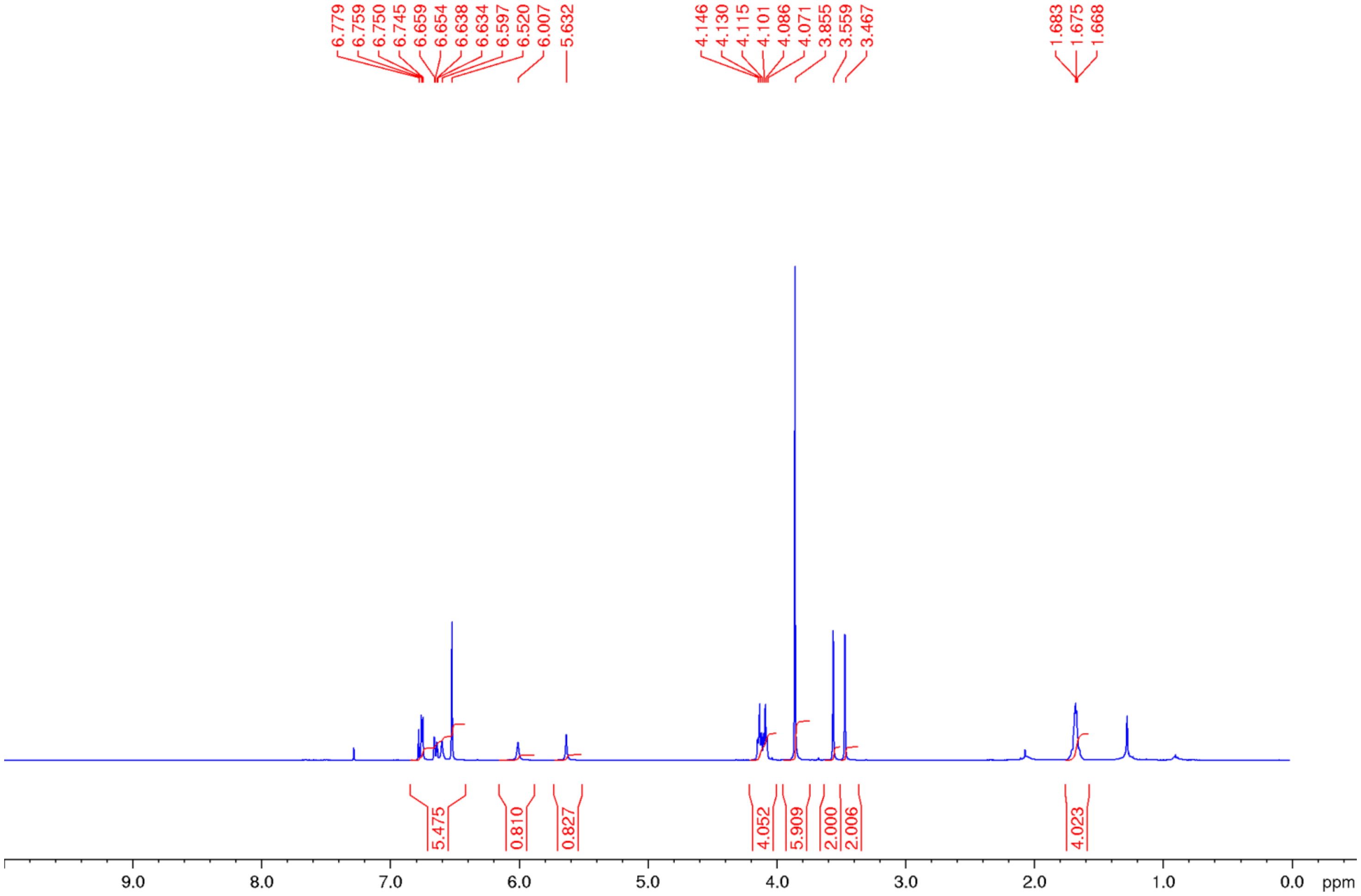

^{13}C NMR

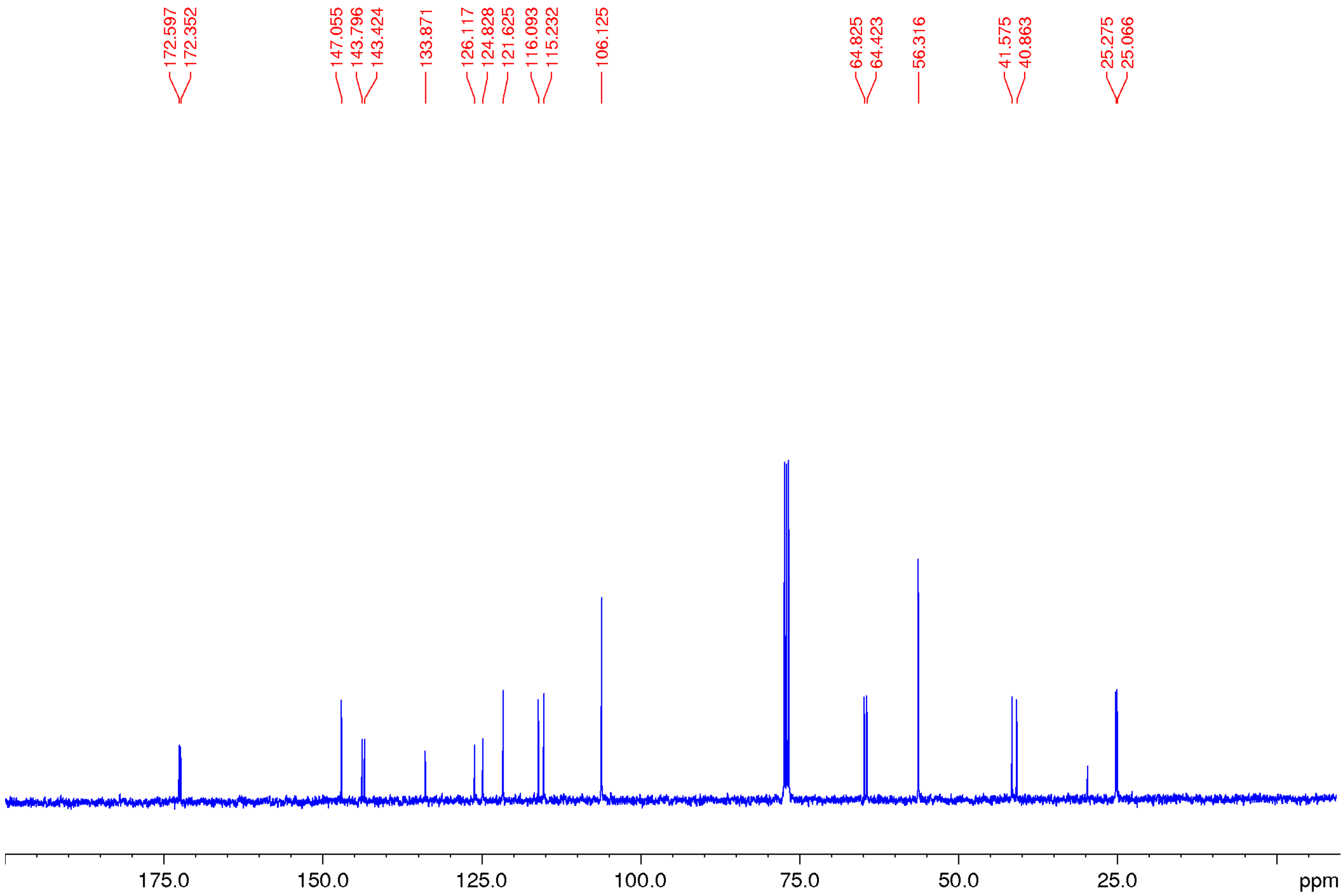
172.597
172.352
147.055
143.796
143.424
133.871
126.117
124.828
121.625
116.093
115.232
106.125
64.825
64.423
56.316
41.575
40.863
25.275
25.066
175.0
150.0
125.0
100.0
75.0
50.0
25.0
ppm

HRMS

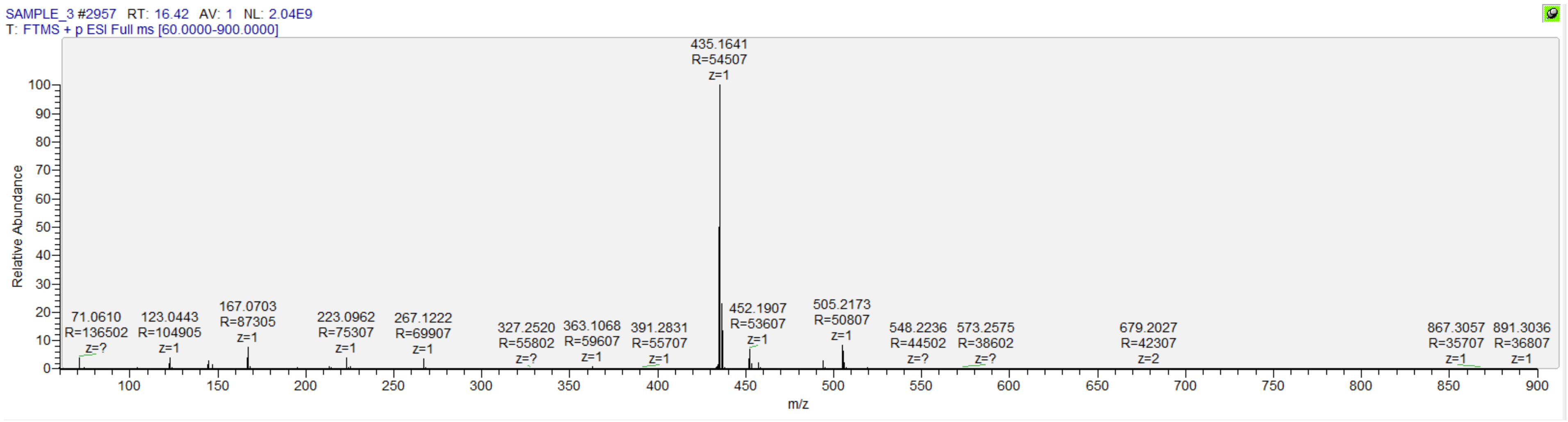

SAMPLE_3 #2957 RT: 16.42 AV: 1 NL: 2.04E9
T: FTMS + p ESI Full ms [60.0000-900.0000]
435.1641
R=54507
z=1
71.0610
R=136502
z=?
123.0443
R=104905
z=1
167.0703
R=87305
z=1
223.0962
R=75307
z=1
267.1222
R=69907
z=1
327.2520
R=55802
z=?
363.1068
R=59607
z=1
391.2831
R=55707
z=1
452.1907
R=53607
z=1
505.2173
R=50807
z=1
548.2236
R=44502
z=?
573.2575
R=38602
z=?
679.2027
R=42307
z=2
867.3057
R=35707
z=1
891.3036
R=36807
z=1
Relative Abundance
m/z

FTIR

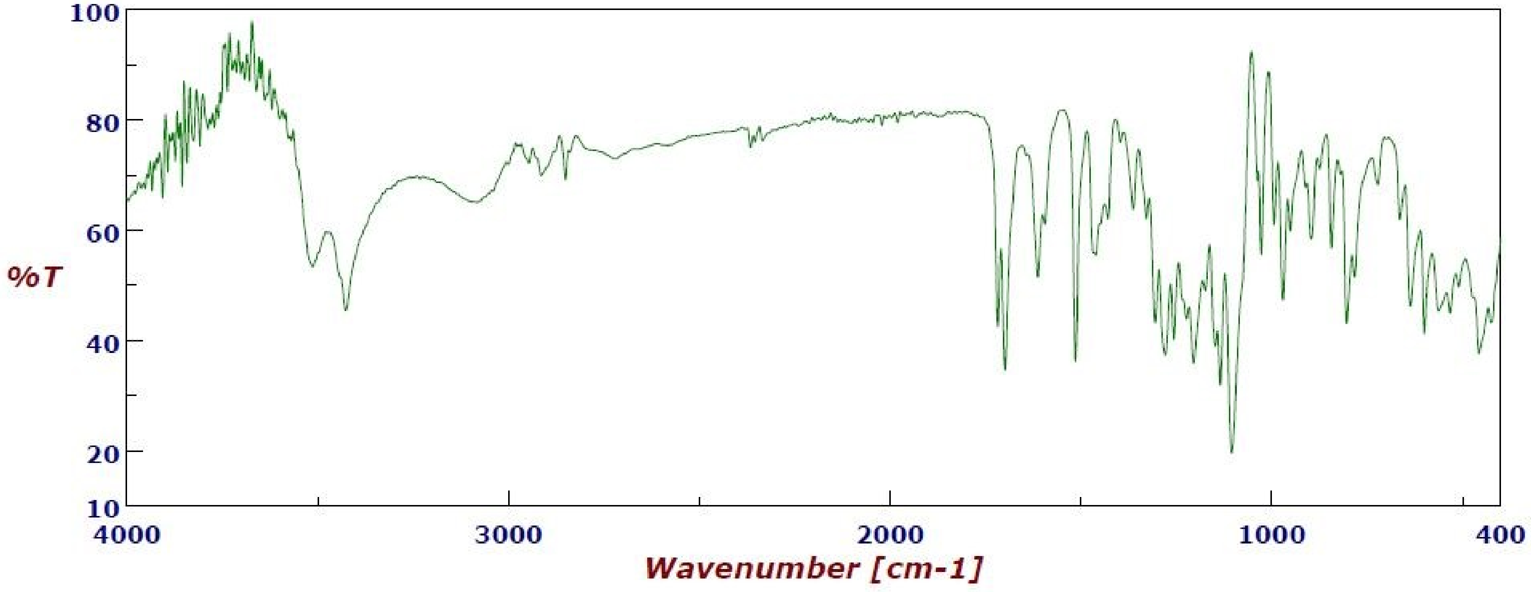

%T
Wavenumber [cm-1]

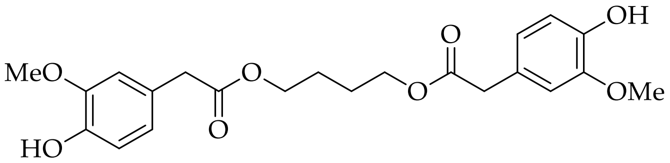

Butane-1,4-diyl bis(2-(4-hydroxy-3-methoxyphenyl)acetate) **39**

^{1}H NMR

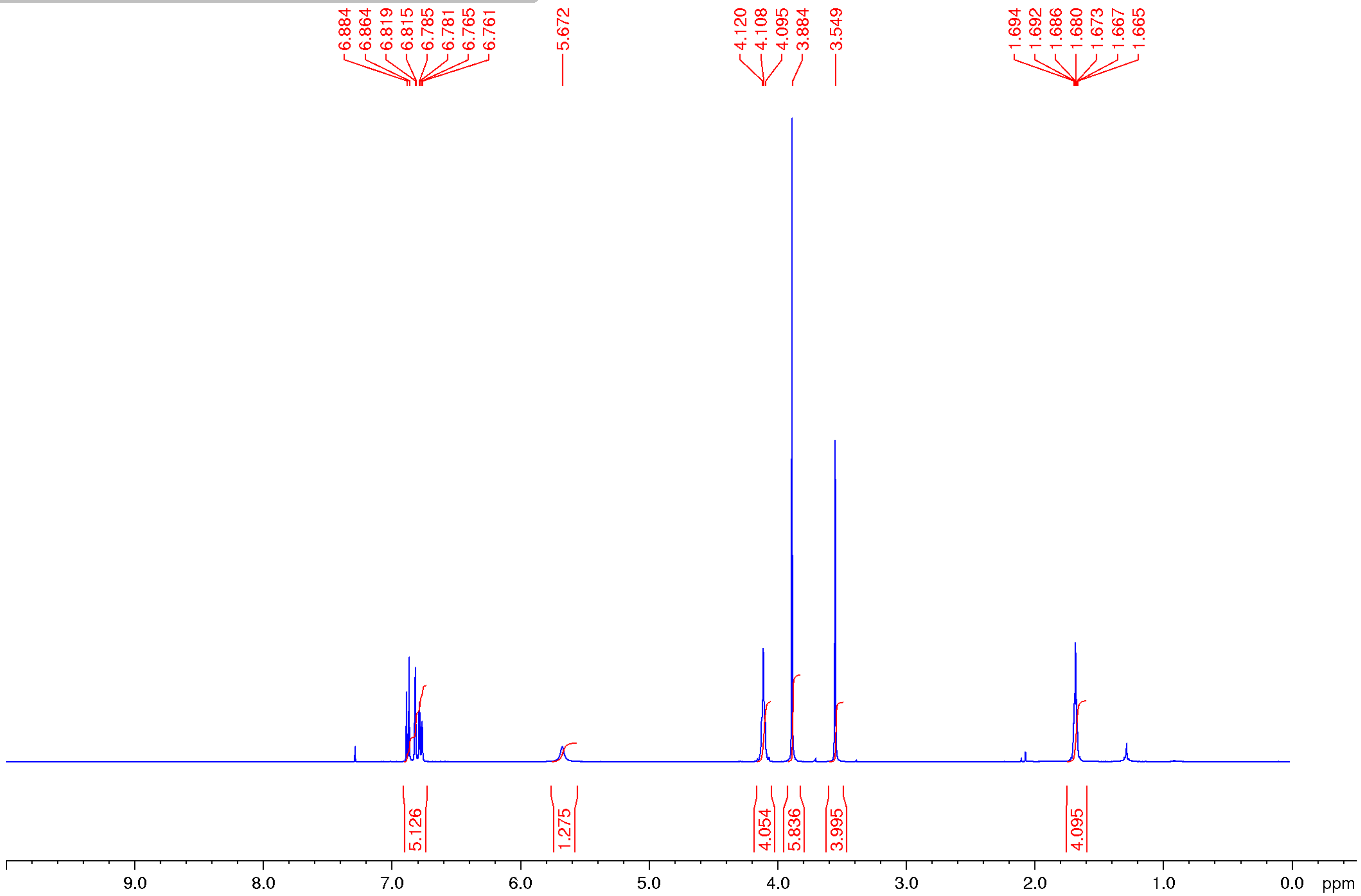

^{13}C NMR

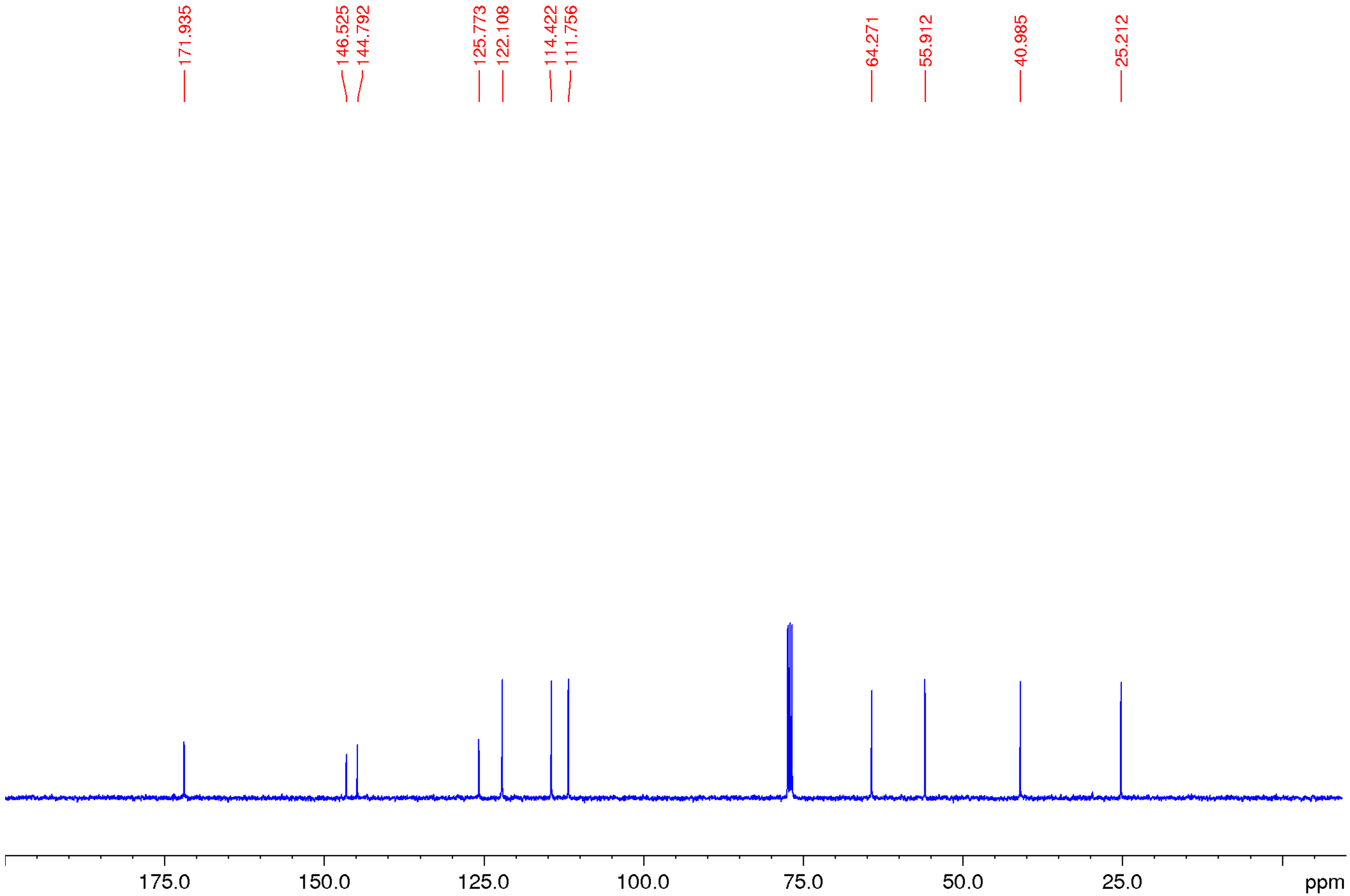
171.935
146.525
144.792
125.773
122.108
114.422
111.756
64.271
55.912
40.985
25.212
175.0
150.0
125.0
100.0
75.0
50.0
25.0
ppm

HRMS

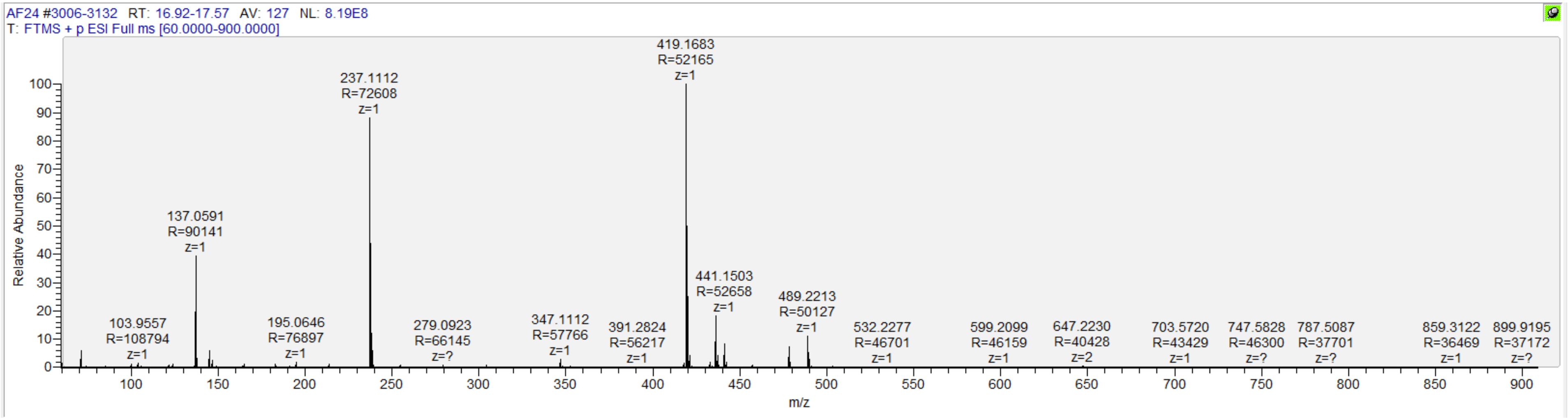
AF24 #3006-3132 RT: 16.92-17.57 AV: 127 NL: 8.19E8
T: FTMS + p ESI Full ms [60.0000-900.0000]
419.1683
R=52165
z=1
237.1112
R=72608
z=1
137.0591
R=90141
z=1
441.1503
R=52658
z=1
489.2213
R=50127
z=1
103.9557
R=108794
z=1
195.0646
R=76897
z=1
279.0923
R=66145
z=?
347.1112
R=57766
z=1
391.2824
R=56217
z=1
532.2277
R=46701
z=1
599.2099
R=46159
z=1
647.2230
R=40428
z=2
703.5720
R=43429
z=1
747.5828
R=46300
z=?
787.5087
R=37701
z=?
859.3122
R=36469
z=1
899.9195
R=37172
z=?
Relative Abundance
m/z

FTIR

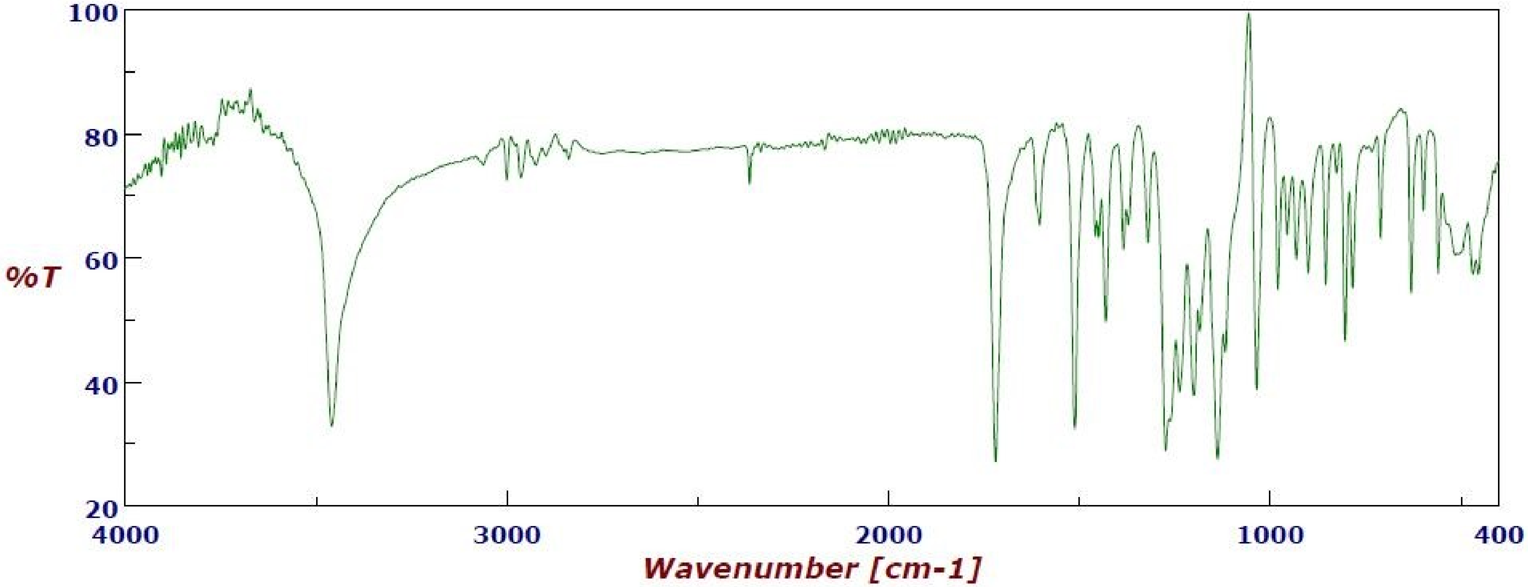
%T
Wavenumber [cm-1]

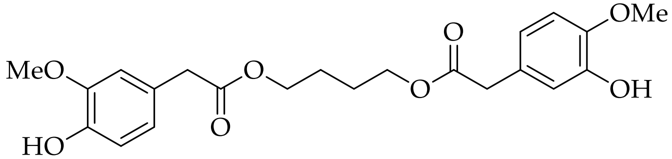

4-(2-(4-Hydroxy-3-methoxyphenyl)acetoxy)butyl 2-(3-hydroxy-4 methoxyphenyl)acetate **40**

^{1}H NMR

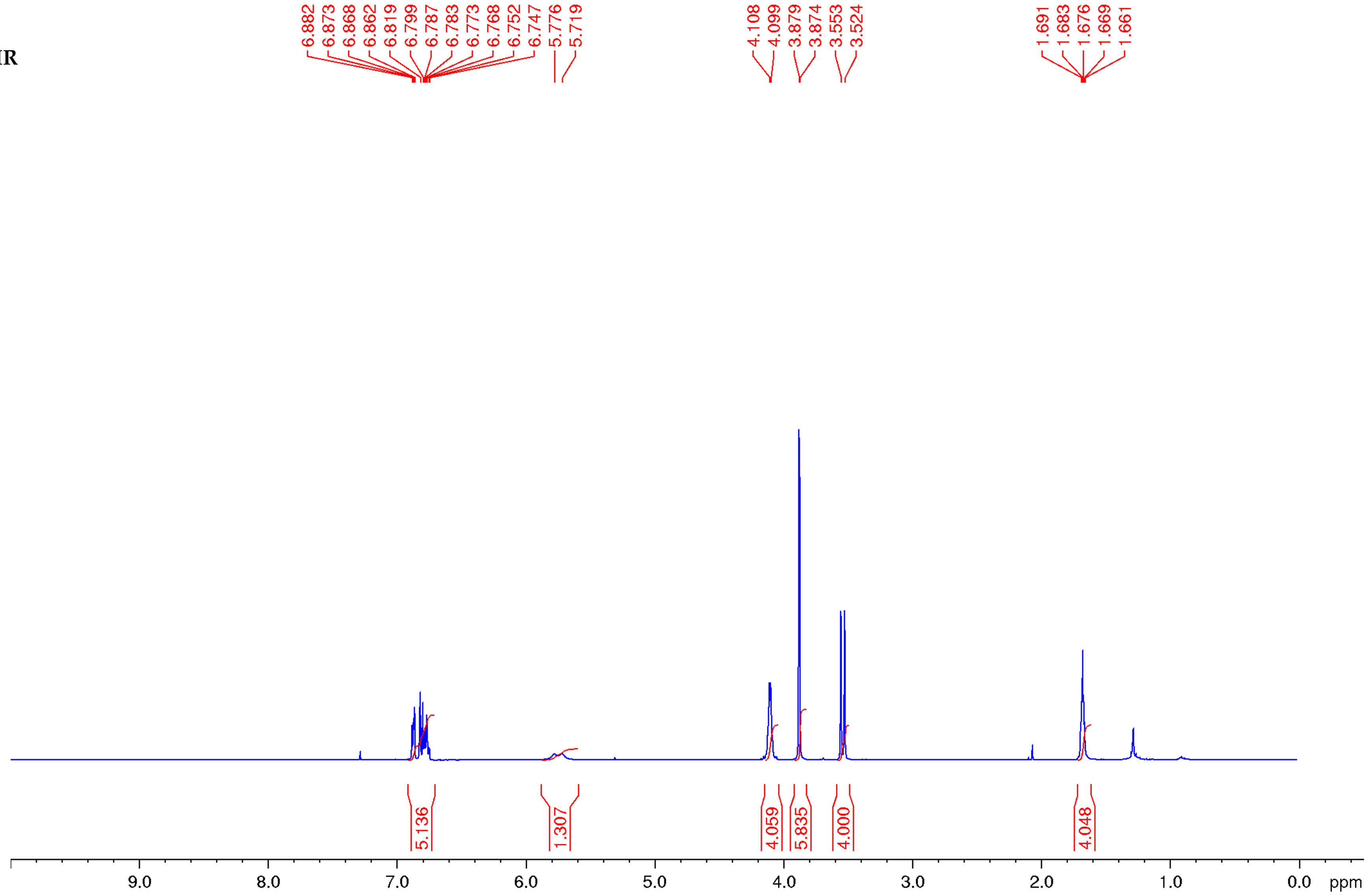

^{13}C NMR

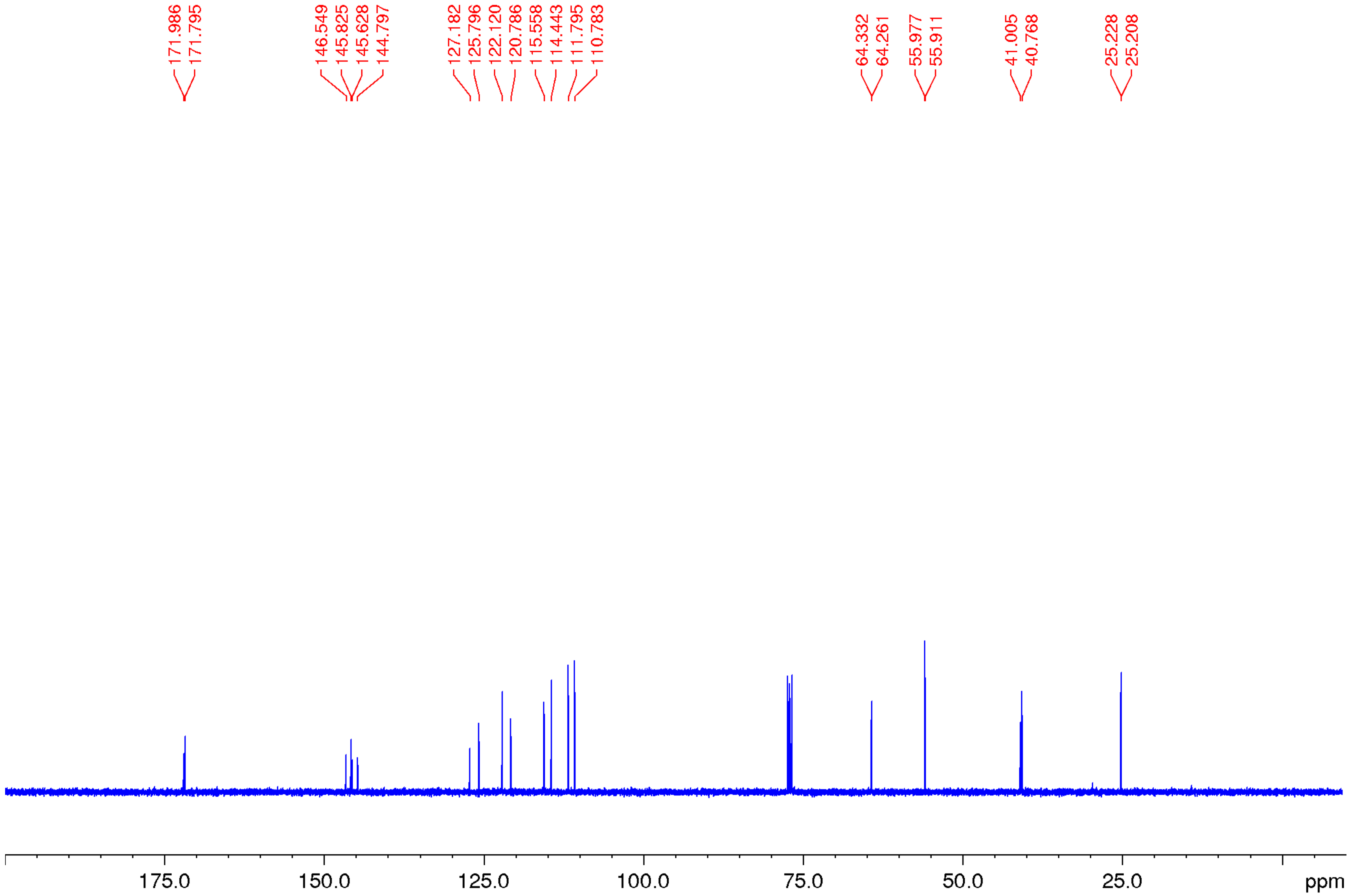
171.986
171.795
146.549
145.825
145.628
144.797
127.182
125.796
122.120
120.786
115.558
114.443
111.795
110.783
64.332
64.261
55.977
55.911
41.005
40.768
25.228
25.208
175.0
150.0
125.0
100.0
75.0
50.0
25.0
ppm

HRMS

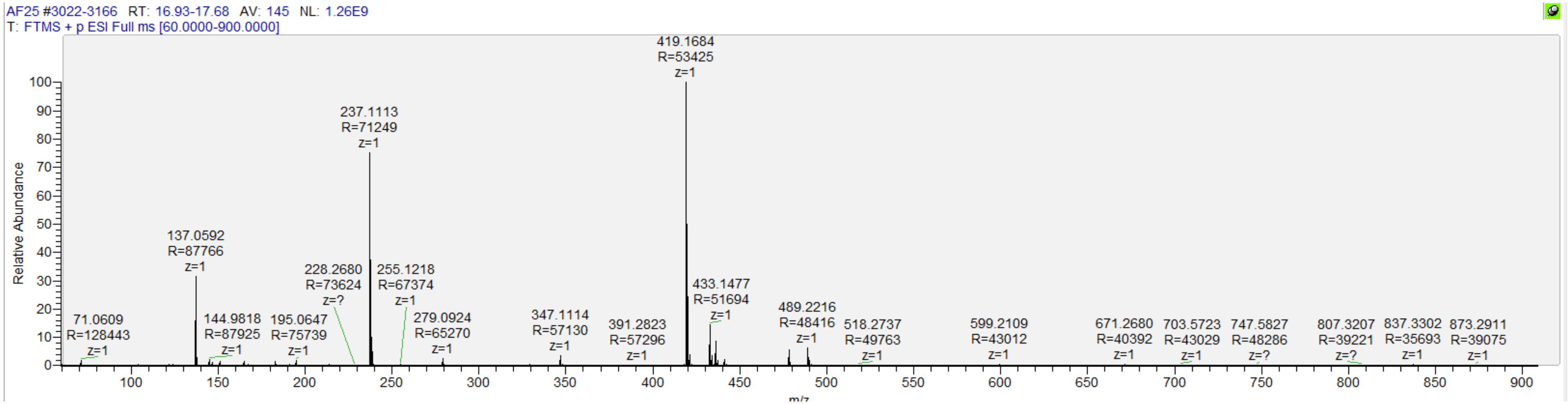
AF25 #3022-3166 RT: 16.93-17.68 AV: 145 NL: 1.26E9
T: FTMS + p ESI Full ms [60.0000-900.0000]
419.1684
R=53425
z=1
237.1113
R=71249
z=1
137.0592
R=87766
z=1
228.2680
R=73624
z=?
255.1218
R=67374
z=1
433.1477
R=51694
z=1
489.2216
R=48416
z=1
71.0609
R=128443
z=1
144.9818
R=87925
z=1
195.0647
R=75739
z=1
279.0924
R=65270
z=1
347.1114
R=57130
z=1
391.2823
R=57296
z=1
518.2737
R=49763
z=1
599.2109
R=43012
z=1
671.2680
R=40392
z=1
703.5723
R=43029
z=1
747.5827
R=48286
z=?
807.3207
R=39221
z=?
837.3302
R=35693
z=1
873.2911
R=39075
z=1
Relative Abundance
m/z

FTIR

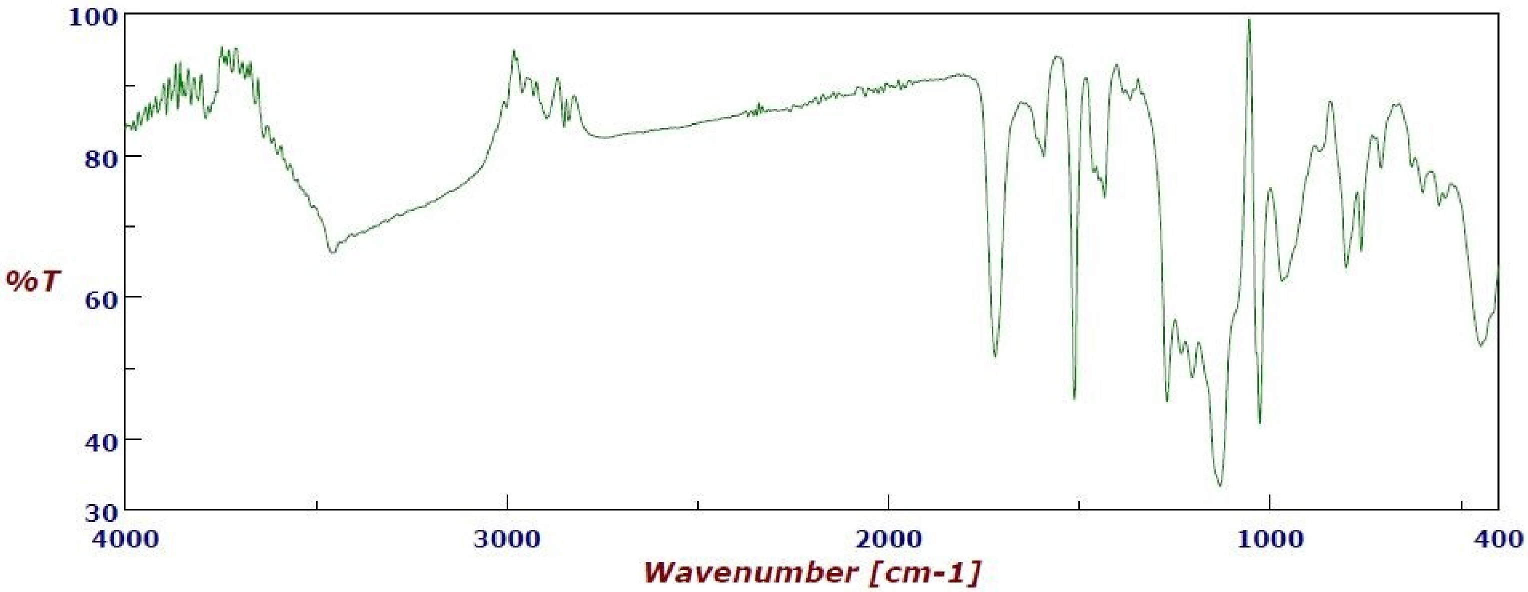
%T
Wavenumber [cm-1]

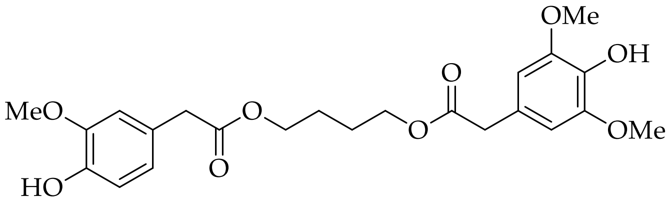

4-(2-(4-Hydroxy-3,5-dimethoxyphenyl)acetoxy)butyl 2-(4-hydroxy-3-methoxyphenyl)acetate **41**

¹H NMR

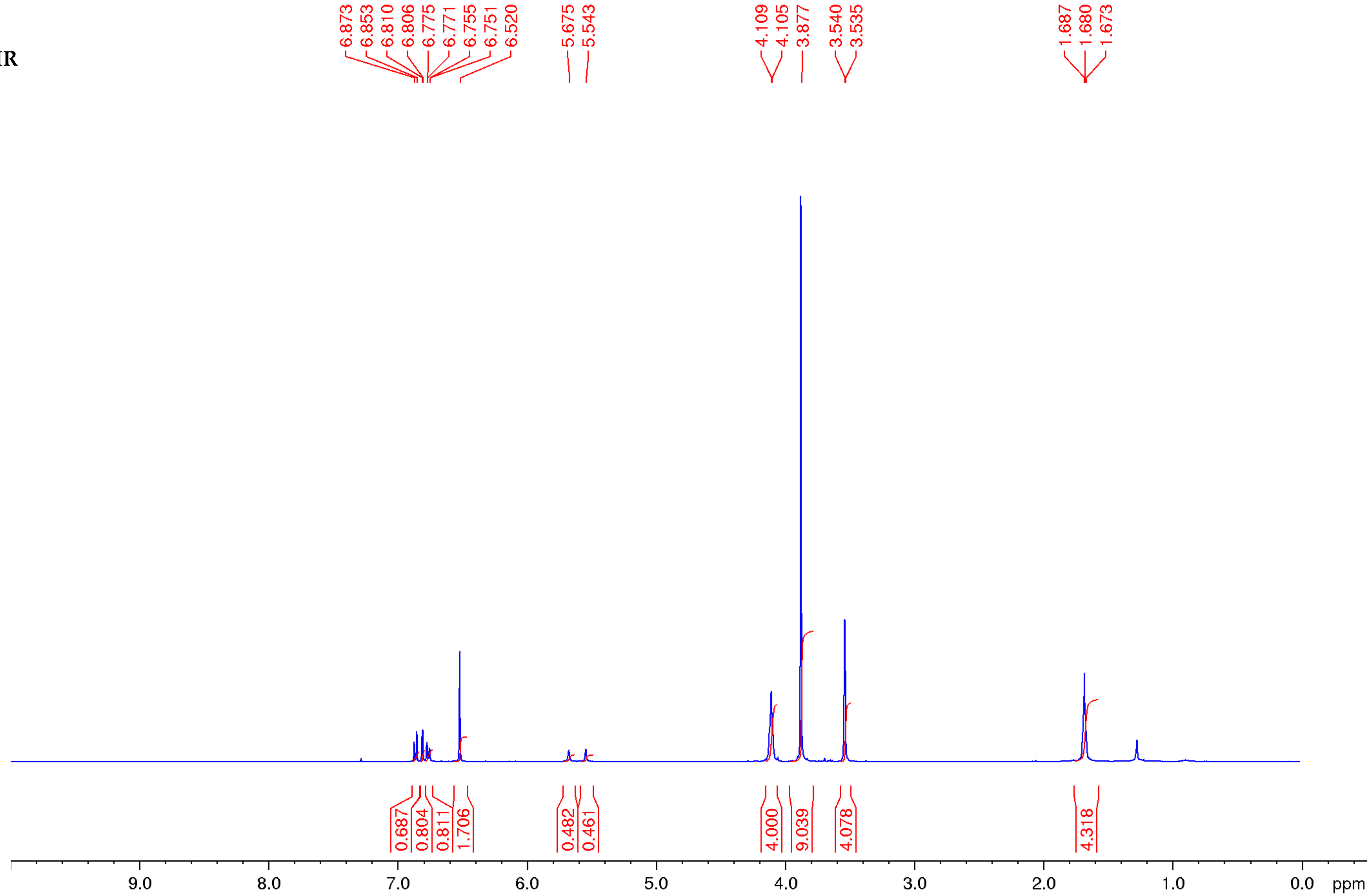

¹³C NMR

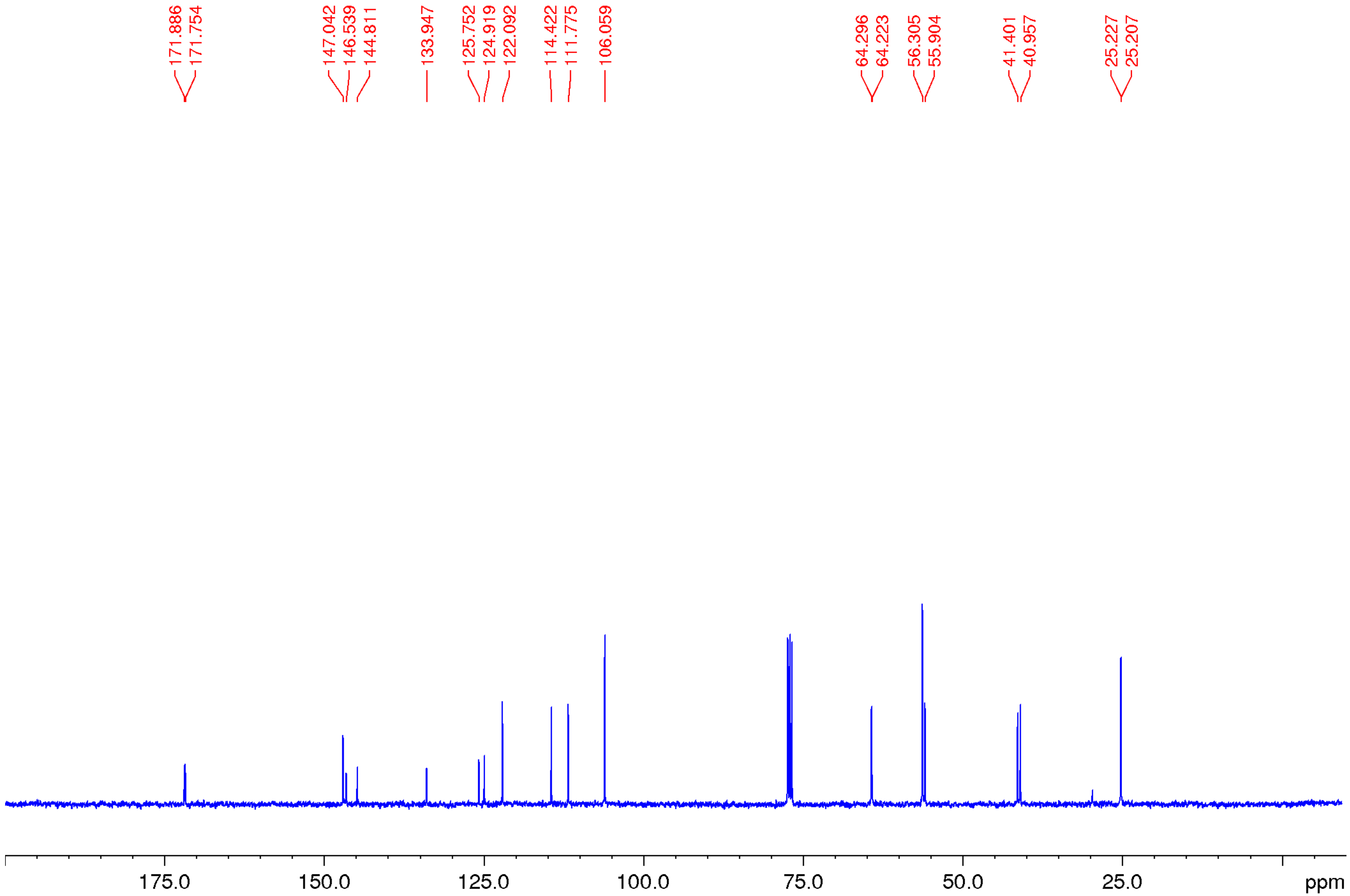
171.886
171.754
147.042
146.539
144.811
133.947
125.752
124.919
122.092
114.422
111.775
106.059
64.296
64.223
56.305
55.904
41.401
40.957
25.227
25.207
175.0
150.0
125.0
100.0
75.0
50.0
25.0
ppm

HRMS

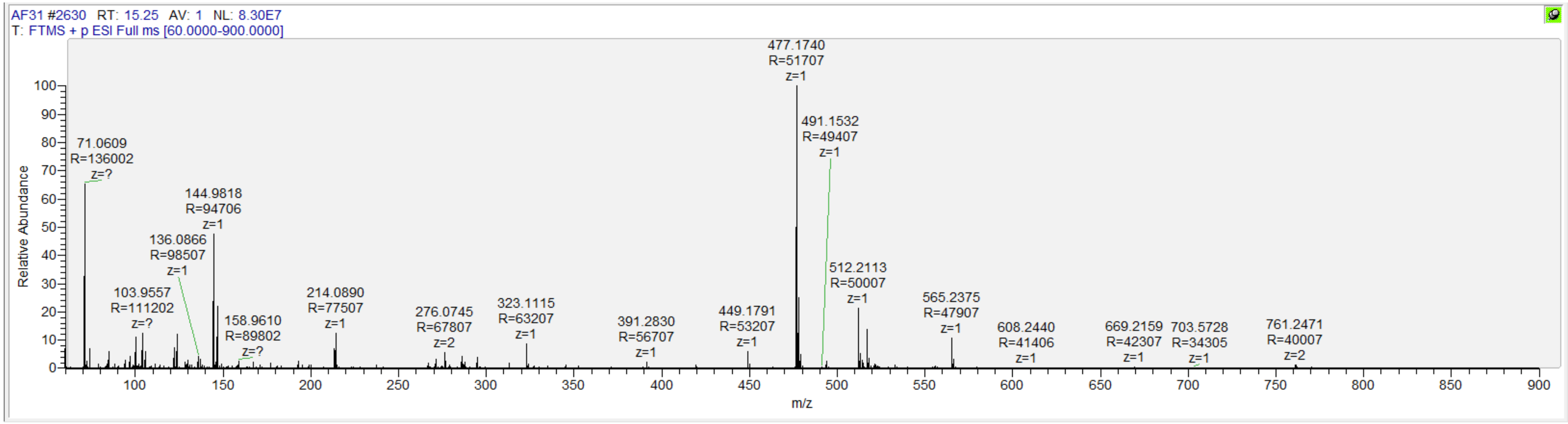
AF31 #2630 RT: 15.25 AV: 1 NL: 8.30E7
T: FTMS + p ESI Full ms [60.0000-900.0000]
Relative Abundance
m/z
477.1740 R=51707 z=1
491.1532 R=49407 z=1
71.0609 R=136002 z=?
144.9818 R=94706 z=1
136.0866 R=98507 z=1
103.9557 R=111202 z=?
158.9610 R=89802 z=?
214.0890 R=77507 z=1
276.0745 R=67807 z=2
323.1115 R=63207 z=1
391.2830 R=56707 z=1
449.1791 R=53207 z=1
512.2113 R=50007 z=1
565.2375 R=47907 z=1
608.2440 R=41406 z=1
669.2159 R=42307 z=1
703.5728 R=34305 z=1
761.2471 R=40007 z=2

FTIR

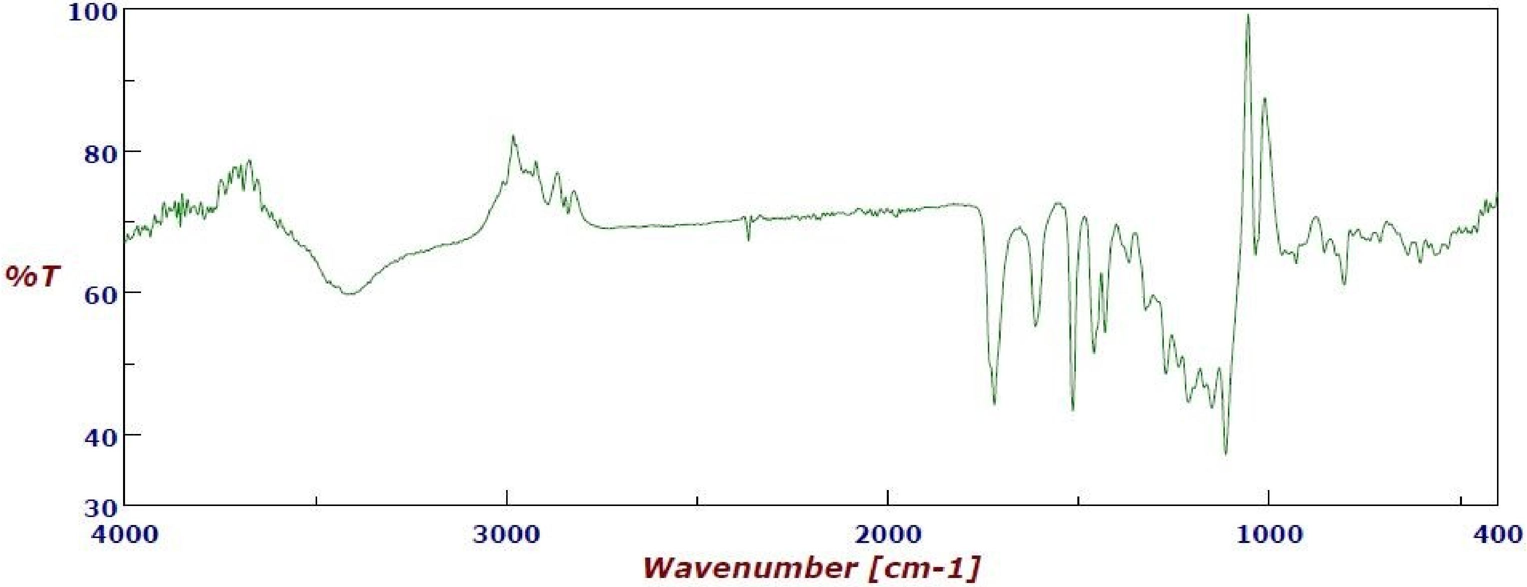
%T
Wavenumber [cm-1]
100
80
60
40
30
4000
3000
2000
1000
400

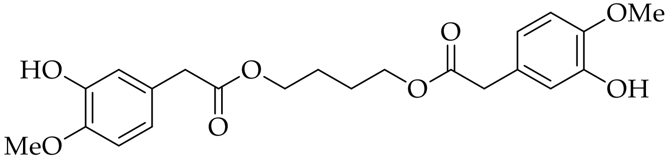

Butane-1,4-diyl bis(2-(3-hydroxy-4-methoxyphenyl)acetate) **42**

^{1}H NMR

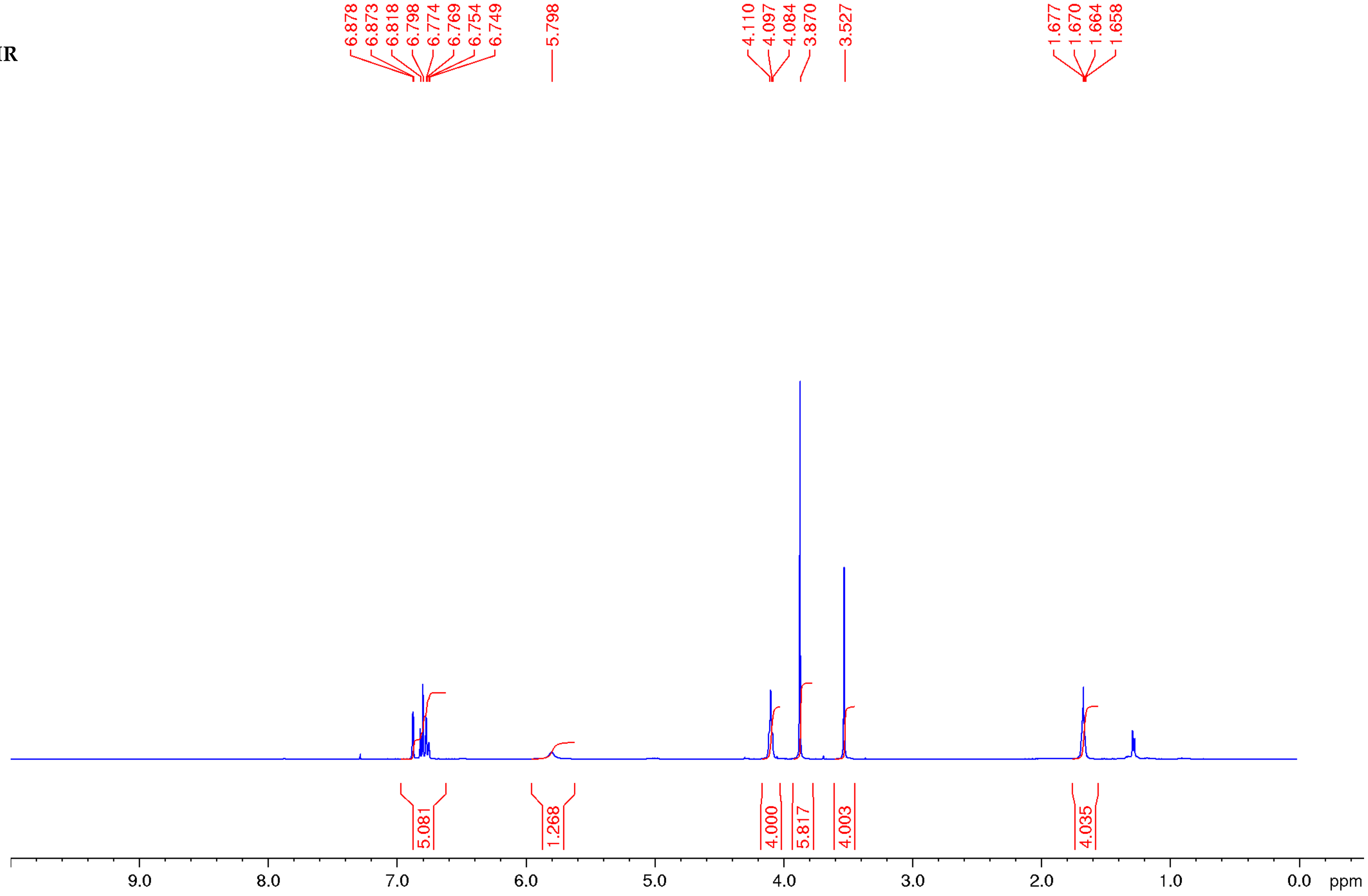

^{13}C NMR

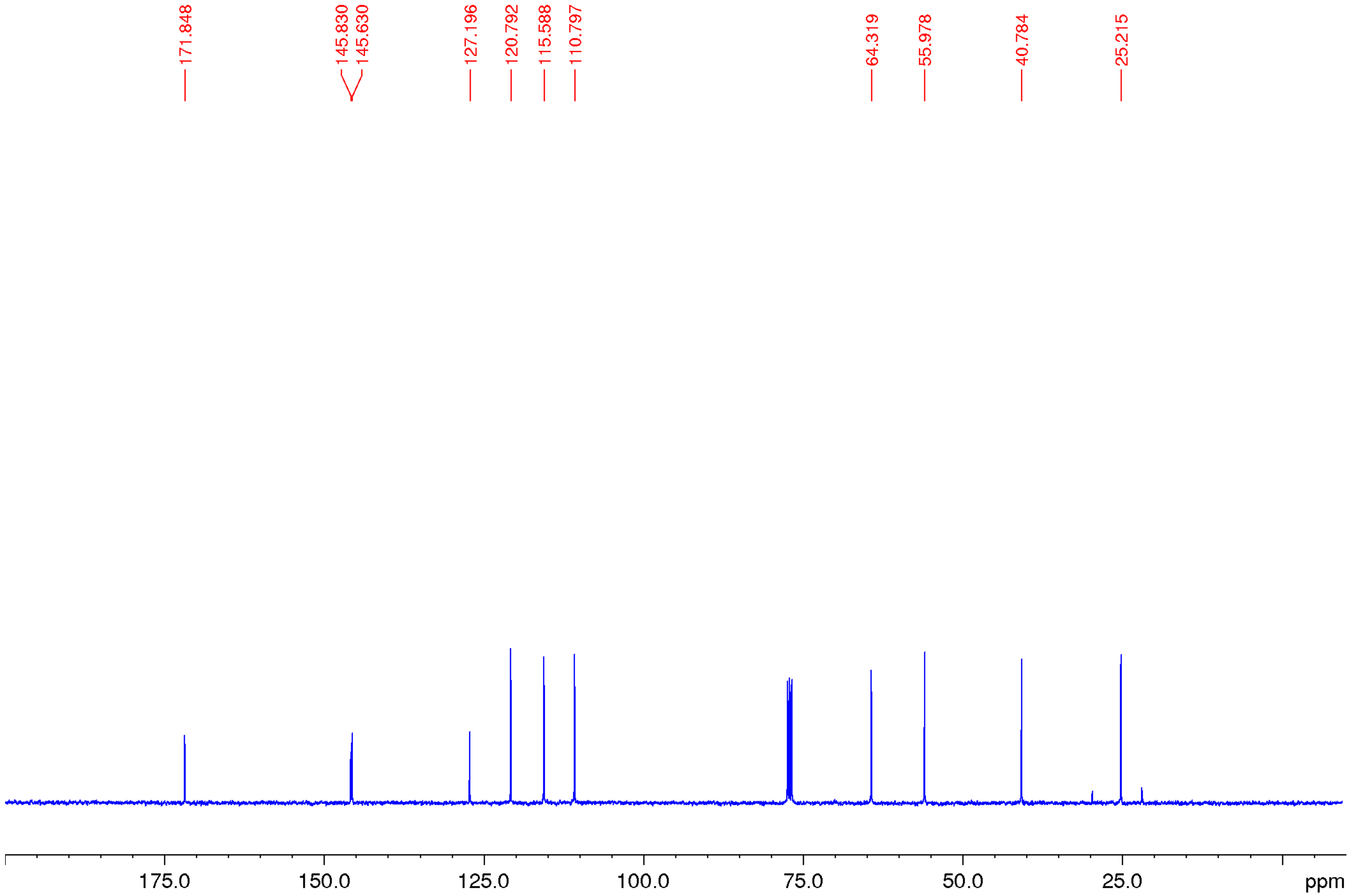

171.848
145.830
145.630
127.196
120.792
115.588
110.797
64.319
55.978
40.784
25.215
175.0
150.0
125.0
100.0
75.0
50.0
25.0
ppm

HRMS

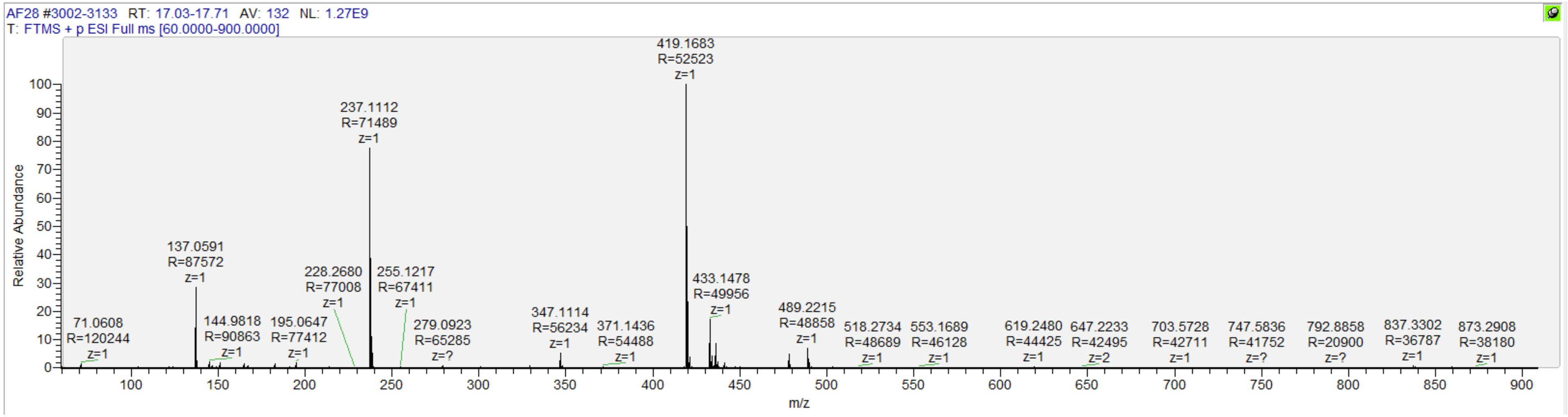
AF28 #3002-3133 RT: 17.03-17.71 AV: 132 NL: 1.27E9
T: FTMS + p ESI Full ms [60.0000-900.0000]
419.1683
R=52523
z=1
237.1112
R=71489
z=1
137.0591
R=87572
z=1
228.2680
R=77008
z=1
255.1217
R=67411
z=1
433.1478
R=49956
z=1
489.2215
R=48858
z=1
71.0608
R=120244
z=1
144.9818
R=90863
z=1
195.0647
R=77412
z=1
279.0923
R=65285
z=?
347.1114
R=56234
z=1
371.1436
R=54488
z=1
518.2734
R=48689
z=1
553.1689
R=46128
z=1
619.2480
R=44425
z=1
647.2233
R=42495
z=2
703.5728
R=42711
z=1
747.5836
R=41752
z=?
792.8858
R=20900
z=?
837.3302
R=36787
z=1
873.2908
R=38180
z=1
Relative Abundance
m/z

FTIR

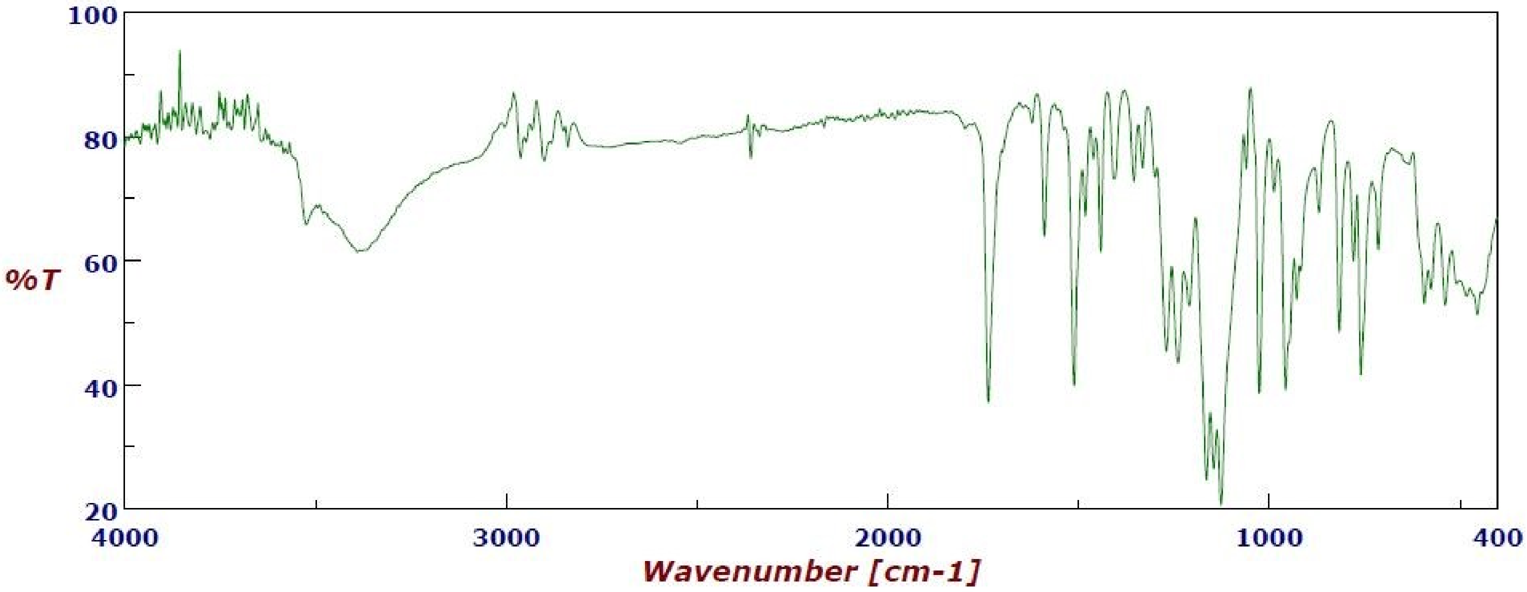
%T
Wavenumber [cm-1]

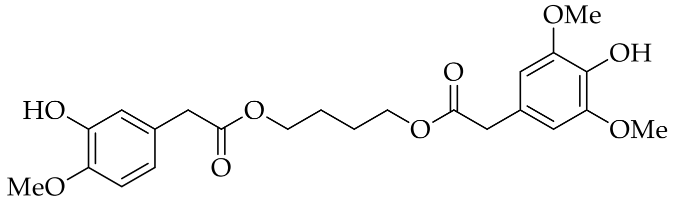

4-(2-(4-Hydroxy-3,5-dimethoxyphenyl)acetoxy)butyl 2-(3-hydroxy-4- methoxyphenyl)acetate **43**

^{1}H NMR

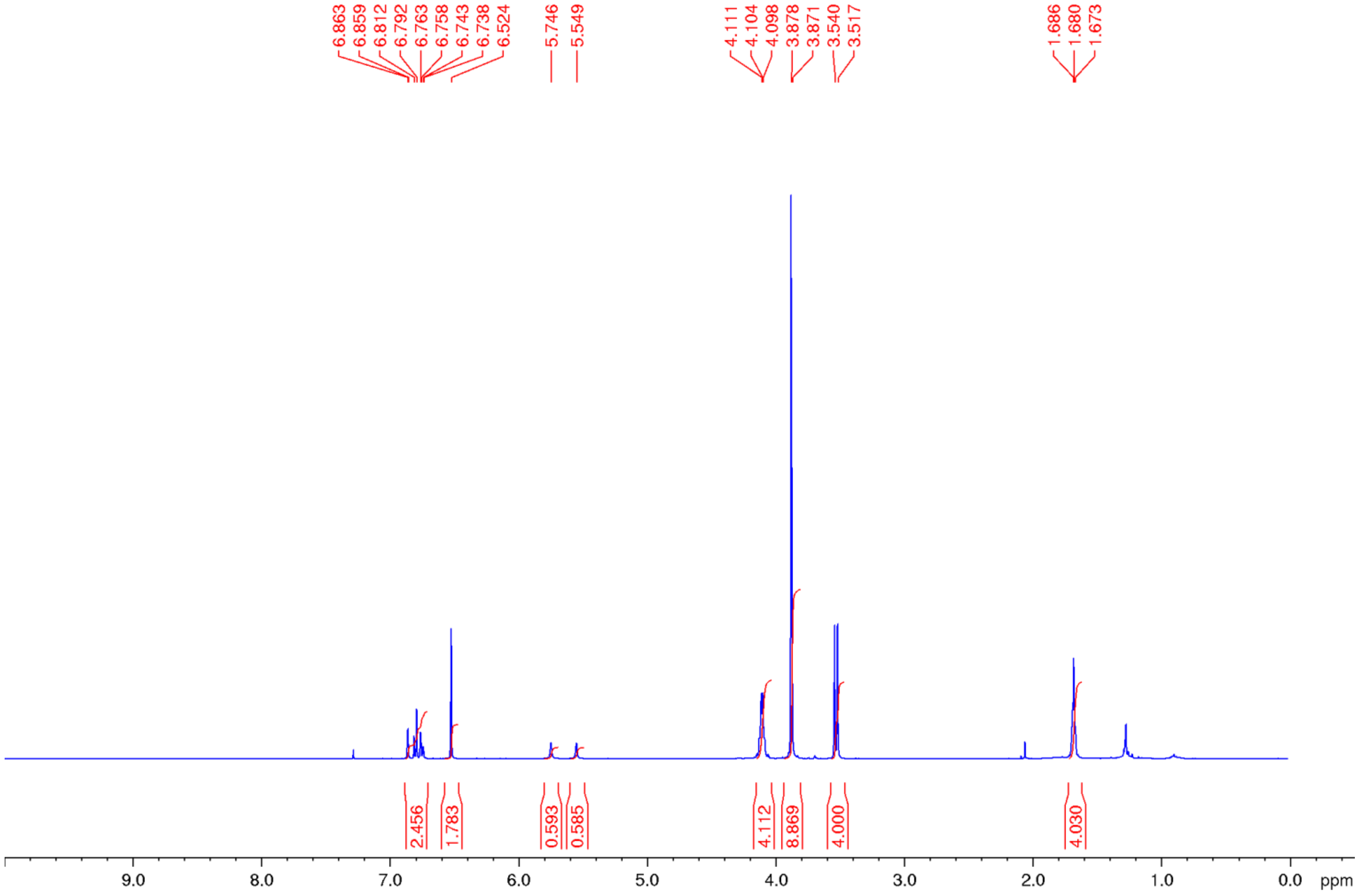

^{13}C NMR

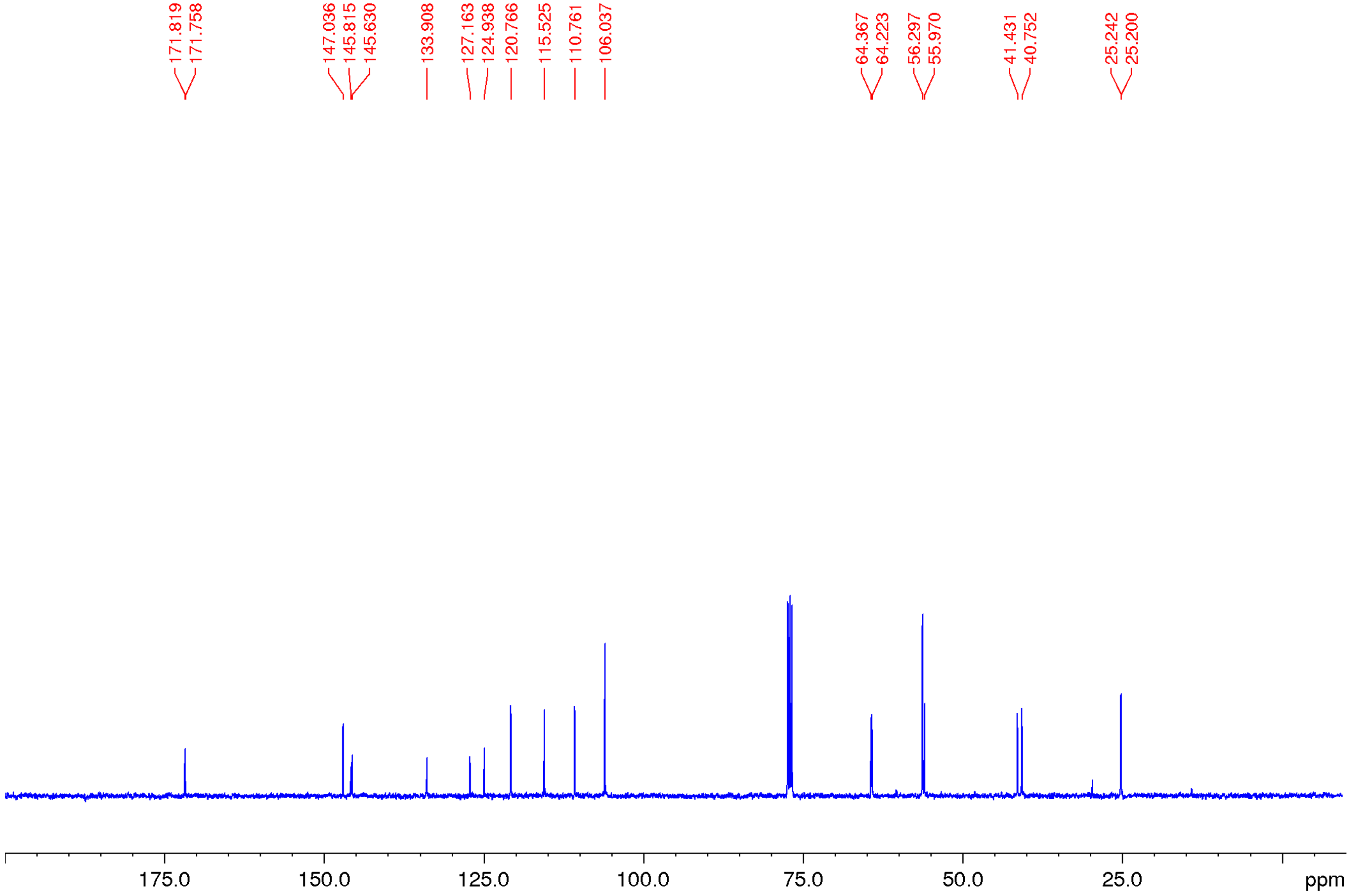

171.819
171.758
147.036
145.815
145.630
133.908
127.163
124.938
120.766
115.525
110.761
106.037
64.367
64.223
56.297
55.970
41.431
40.752
25.242
25.200
175.0
150.0
125.0
100.0
75.0
50.0
25.0
ppm

HRMS

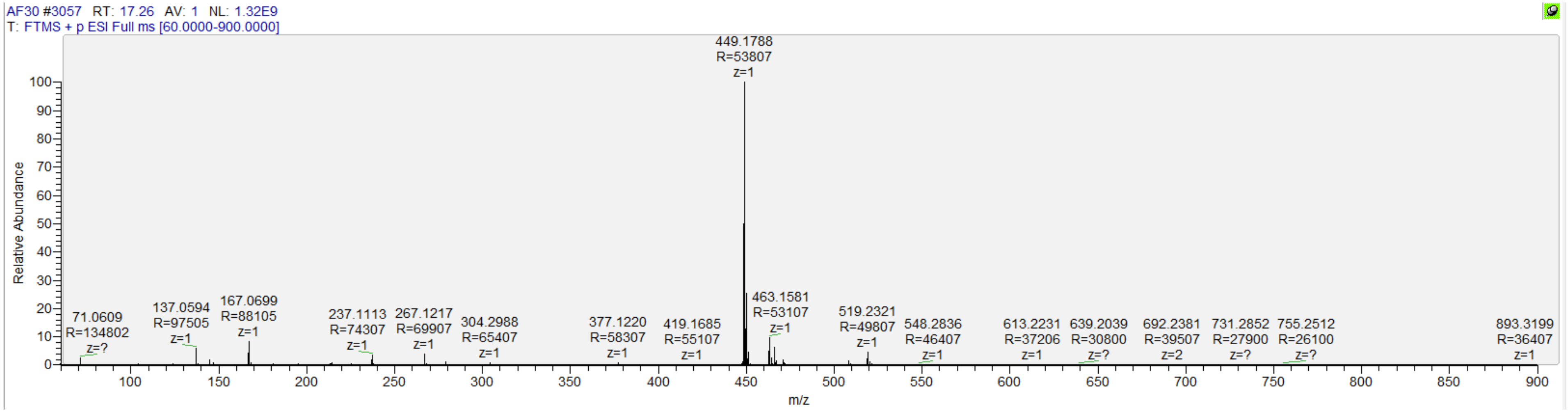
AF30 #3057 RT: 17.26 AV: 1 NL: 1.32E9
T: FTMS + p ESI Full ms [60.0000-900.0000]
449.1788
R=53807
z=1
463.1581
R=53107
z=1
71.0609 R=134802 z=?
137.0594 R=97505 z=1
167.0699 R=88105 z=1
237.1113 R=74307 z=1
267.1217 R=69907 z=1
304.2988 R=65407 z=1
377.1220 R=58307 z=1
419.1685 R=55107 z=1
519.2321 R=49807 z=1
548.2836 R=46407 z=1
613.2231 R=37206 z=1
639.2039 R=30800 z=?
692.2381 R=39507 z=2
731.2852 R=27900 z=?
755.2512 R=26100 z=?
893.3199 R=36407 z=1
Relative Abundance
m/z

FTIR

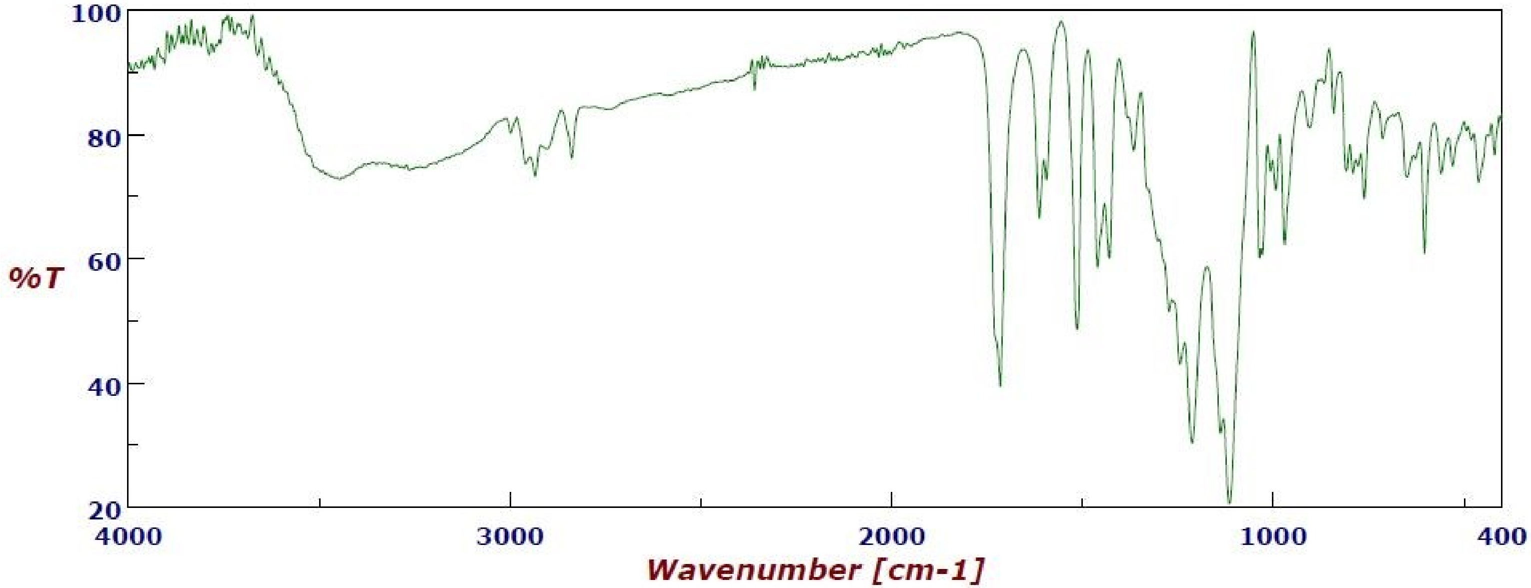
%T
Wavenumber [cm-1]
100
80
60
40
20
4000
3000
2000
1000
400

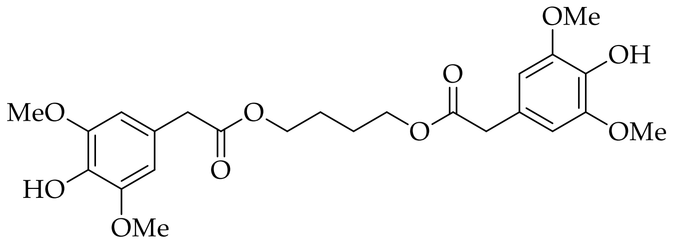

Butane-1,4-diyl bis(2-(4-hydroxy-3,5-dimethoxyphenyl)acetate) **44**

^{1}H NMR

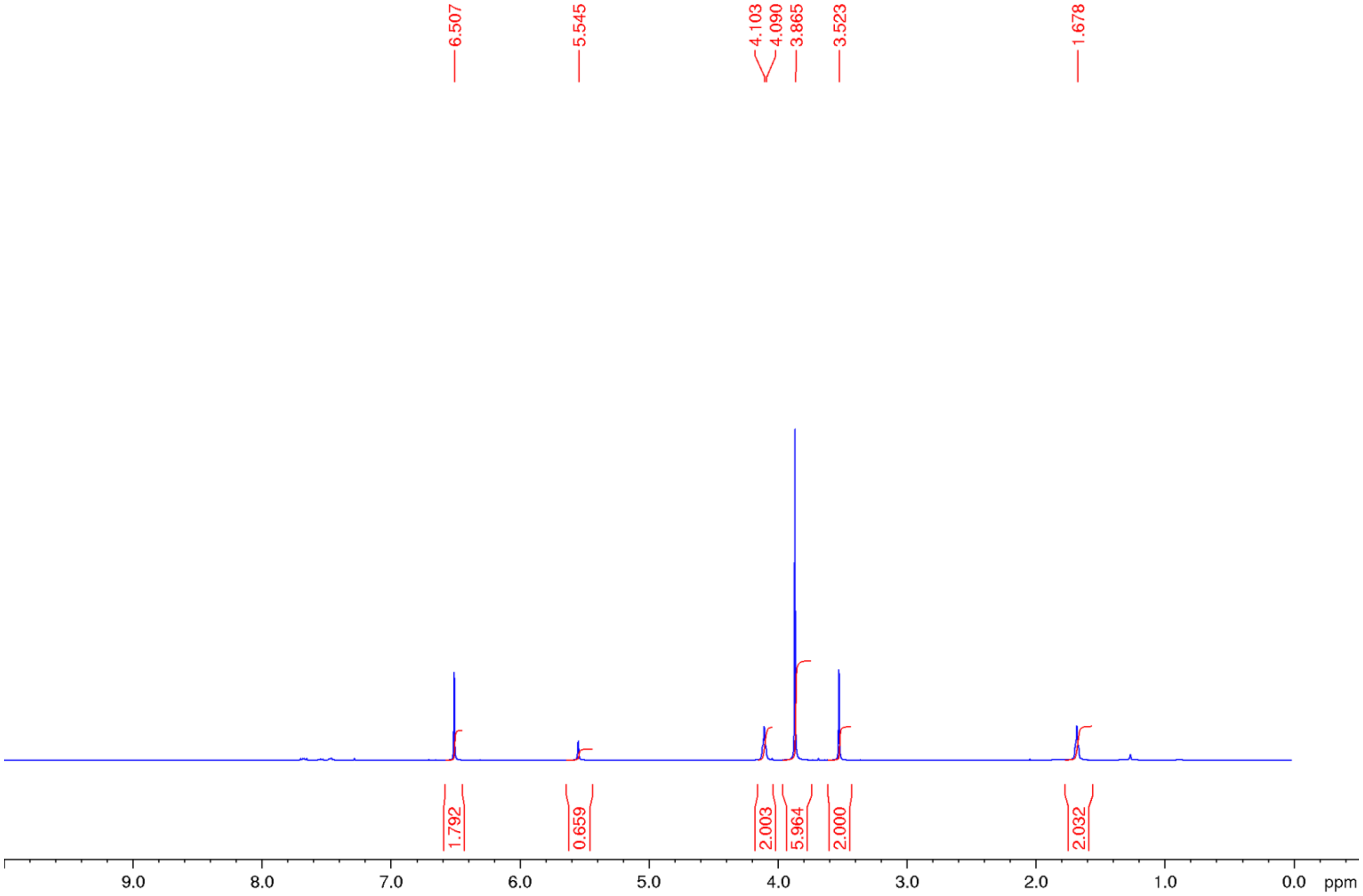

^{13}C NMR

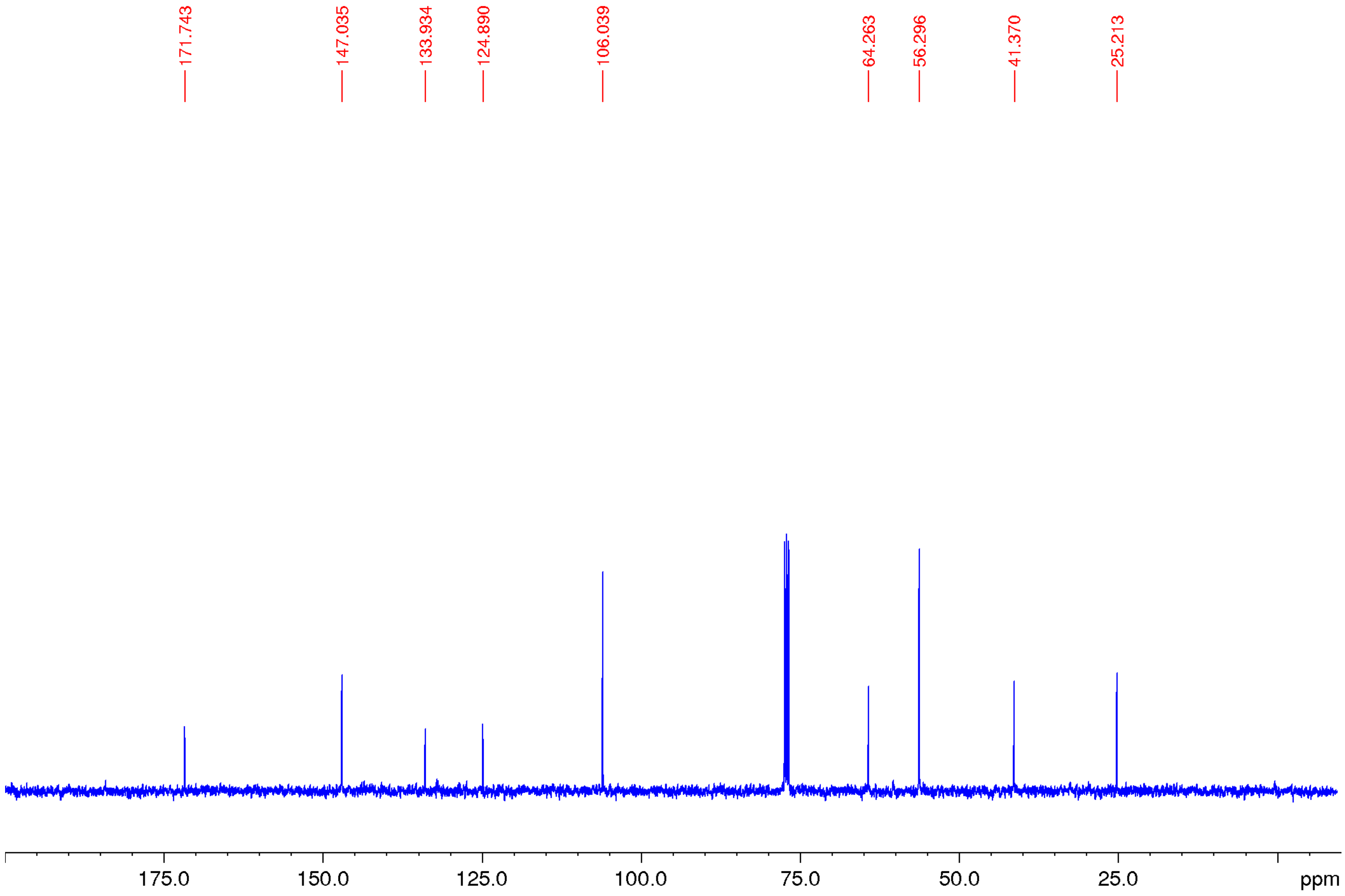
171.743
147.035
133.934
124.890
106.039
64.263
56.296
41.370
25.213
175.0
150.0
125.0
100.0
75.0
50.0
25.0
ppm

HRMS

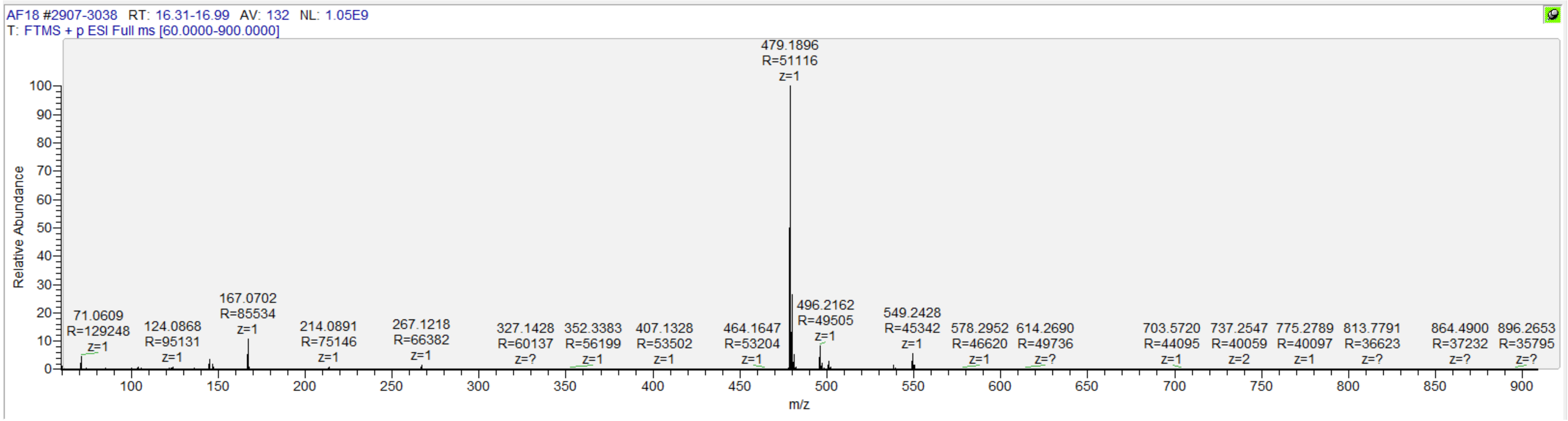
AF18 #2907-3038 RT: 16.31-16.99 AV: 132 NL: 1.05E9
T: FTMS + p ESI Full ms [60.0000-900.0000]
479.1896
R=51116
z=1
Relative Abundance
m/z
71.0609 R=129248 z=1
124.0868 R=95131 z=1
167.0702 R=85534 z=1
214.0891 R=75146 z=1
267.1218 R=66382 z=1
327.1428 R=60137 z=?
352.3383 R=56199 z=1
407.1328 R=53502 z=1
464.1647 R=53204 z=1
496.2162 R=49505 z=1
549.2428 R=45342 z=1
578.2952 R=46620 z=1
614.2690 R=49736 z=?
703.5720 R=44095 z=1
737.2547 R=40059 z=2
775.2789 R=40097 z=1
813.7791 R=36623 z=?
864.4900 R=37232 z=?
896.2653 R=35795 z=?

FTIR

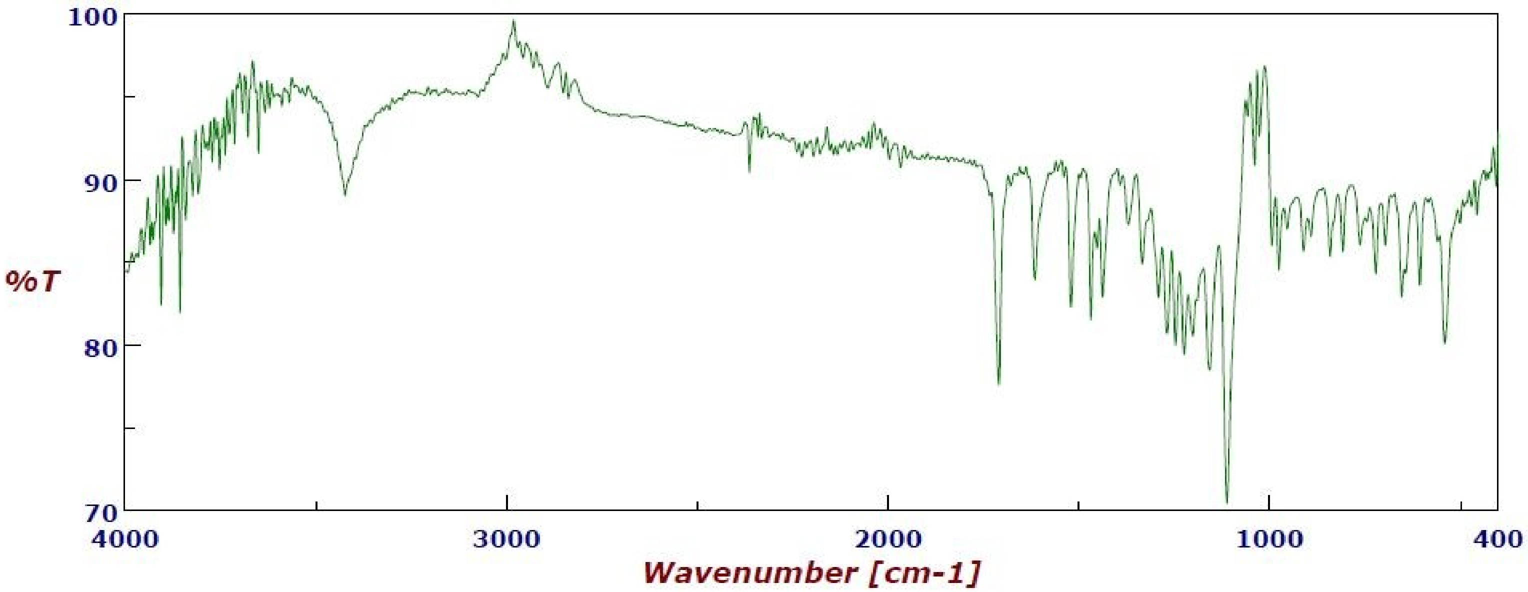
%T
Wavenumber [cm-1]
100
90
80
70
4000
3000
2000
1000
400
